# In Vitro and In Silico Evaluation of Cholinesterase Inhibition by Alkaloids Obtained from Branches of *Abuta panurensis* Eichler

**DOI:** 10.3390/molecules27103138

**Published:** 2022-05-13

**Authors:** Rochelly da Silva Mesquita, Andrii Kyrylchuk, Anton Cherednichenko, Ingrity Suelen Costa Sá, Lílian Macedo Bastos, Felipe Moura Araújo da Silva, Rita de Cássia Saraiva Nunomura, Andriy Grafov

**Affiliations:** 1 Analytical Central—Multidisciplinary Support Center—CAM, Federal University of Amazonas—UFAM, Manaus 69077-000, Amazonas, Brazil; rochellymesquita@gmail.com (R.d.S.M.); ingrity.scosta@gmail.com (I.S.C.S.); lilianbastos3@hotmail.com (L.M.B.); felipemas@ufam.edu.br (F.M.A.d.S.); ritasnunomura@gmail.com (R.d.C.S.N.); 2 Institute of Organic Chemistry, National Academy of Sciences—NAS, 5 Murmanska Str., 02660 Kyiv, Ukraine; iamkaant@gmail.com; 3 Chemspace LLC, Of. 1, 85 Chervonotkatska Str., 02094 Kyiv, Ukraine; 4 Latvian Institute of Organic Synthesis, Aizkraukles 21, 1006 Riga, Latvia; chanton1996@gmail.com; 5 Institute of High Technologies, T. Shevchenko National University, 4-g Prosp. Glushkova, 03022 Kyiv, Ukraine; 6 Department of Chemistry, Federal University of Amazonas-UFAM, Manaus 69077-000, Amazonas, Brazil; 7 Department of Chemistry, University of Helsinki, A.I. Virtasen Aukio 1, 00560 Helsinki, Finland

**Keywords:** *Abuta panurensis*, alkaloids, acetylcholinesterase, butyrylcholinesterase, inhibitory activity, molecular docking

## Abstract

Alkaloids are natural products known as ethnobotanicals that have attracted increasing attention due to a wide range of their pharmacological properties. In this study, cholinesterase inhibitors were obtained from branches of *Abuta panurensis* Eichler (Menispermaceae), an endemic species from the Amazonian rainforest. Five alkaloids were isolated, and their structure was elucidated by a combination of 1D and 2D ^1^H and ^13^C NMR spectroscopy, HPLC-MS, and high-resolution MS: Lindoldhamine isomer *m*/*z* 569.2674 (**1**), stepharine *m*/*z* 298.1461 (**2**), palmatine *m*/*z* 352.1616 (**3**), 5-*N*-methylmaytenine *m*/*z* 420.2669 (**4**) and the *N*-*trans*-feruloyltyramine *m*/*z* 314.1404 (**5**). The compounds **1**, **3**, and **5** were isolated from *A. panurensis* for the first time. Interaction of the above-mentioned alkaloids with acetylcholinesterase (AChE) and butyrylcholinesterase (BChE) enzymes was investigated in silico by molecular docking and molecular dynamics. The molecules under investigation were able to bind effectively with the active sites of the AChE and BChE enzymes. The compounds **1**–**4** demonstrated in vitro an inhibitory effect on acetylcholinesterase with IC_50_ values in the range of 19.55 µM to 61.24 µM. The data obtained in silico corroborate the results of AChE enzyme inhibition.

## 1. Introduction

The Menispermaceae family has a wide geographic distribution, mainly in tropical and subtropical regions of the world [1]. Some species have been used by indigenous people for a long time to prepare curare poison [2]. The *Abuta* genus is native to tropical Central and South America, where it is represented by more than 30 species, some of which have been used in traditional medicine [1,3].

From the chemical viewpoint, the Menispermaceae family is known as a source of a variety of alkaloids with different structures, such as benzylisoquinoline, benzyltetraisoquinoline, bisbenzyltetraisoquinoline, aporphine, and proaporphine derivatives; less prevalent ones include tropolone-isoquinoline, azafluoranthene, benzazepine, and amide alkaloids [4,5,6,7,8]. The *Abuta* genus is a promising source of alkaloids, particularly the isoquinoline derivatives. There are only a few phytochemical investigations regarding the alkaloid content on *A. panurensis* published so far. Namely, the isolation of bisbenzylisoquinoline alkaloids panurensine and norpanurensine was reported in mid-1970s [9], and recently we described the isolation of stepharine and 5-*N*-methylmaytenine, proaporphine and polyamine alkaloids, respectively [10]. Those alkaloids reveal a wide range of pharmacological properties including the inhibition of acetylcholinesterase (AChE) and butyrylcholinesterase (BChE) enzymes [11,12,13,14,15,16], cytotoxic [17,18,19,20], and immunomodulatory activity [10,21].

**Figure 1:**
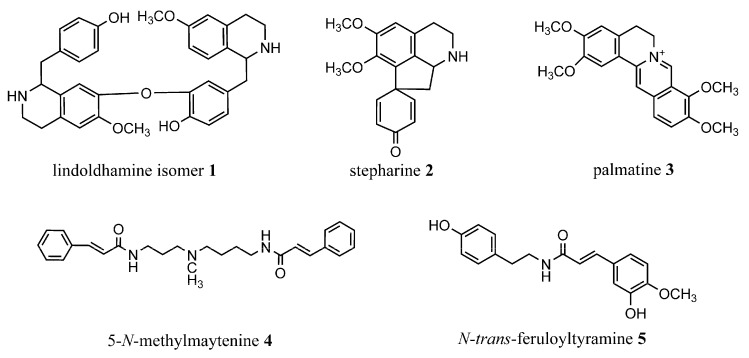
The structures of compounds **1–5**. (**1**) Lindoldhamine isomer, (**2**) stepharine, (**3**) palmatine, (**4**) 5-*N*-methylmaytenine, and the (**5**) *N-trans*-feruloyltyramine.

In the present study, we isolated the lindoldhamine isomer **1**, palmatine **3** and the *N*-*trans*-feruloyltyramine **5** for the first time from *A. panurensis*, while the other two alkaloids (stepharine **2** and 5-*N*-methylmaytenine **4**) were isolated by us earlier [10]. This is also the first report on the occurrence of **1** and **5** in the *Abuta* genus. The potential of alkaloids isolated as AChE inhibitors was demonstrated by in vitro studies. Interactions of the alkaloids in question with AChE and BChE enzymes were investigated in silico by molecular docking to investigate the affinity of the alkaloids at the active sites of AChE and BChE. The chemical structures of the isolated alkaloids are shown in the Figure 1.

Lindoldhamine was previously reported in Menispermaceae family in the genera *Triclisia* [12], *Abuta* [22], and *Albertisia* [23]. Lindoldhamine isomer **1** was first reported in arrow and dart poison of the Matis tribe [24]. Bisbenzyltetrahydroisoquinoline alkaloids represent a large and important class of natural products [1,25] possessing a variety of pharmacological properties, including the acetyl- and butyrylcholinesterase inhibitory activity [11,12,13,26,27].

Palmatine **3** is the most represented protoberberine alkaloid described in Menispermaceae family, being encountered in the *Stephania* [7,28,29,30], *Tinospora* [31,32,33,34,35,36], *Coscinium* [37], *Abuta* [38], and *Arcangelisia* [39] genera. Protoberberine alkaloids are well known for their neuroprotective, antioxidant, and cholinesterases (ChEs) inhibitory activities [40,41] among different other pharmacological properties [42,43].

An amide alkaloid *N-trans*-feruloyltyramine [1] was previously reported in *Tinospora* and *Cocculus* genera of Menispermaceae family [30,31,44,45,46]. This ferulic acid derived of an amide-alkaloid showed interesting anti-inflammatory properties [47,48], but the inhibitory activity toward AChE [49] and antioxidant effect [44] were quite low.

Therefore, all the alkaloids in question possess a common feature—they are inhibitors of predominant cholinesterases. The AChE and BChE are involved in the hydrolysis of a neurotransmitter, acetylcholine. The AChE is highly selective for the neurotransmitter hydrolysis, whereas the BChE can metabolize different substrates. In the brains of Alzheimer’s disease patients, the activity of AChE tends to decrease whereas that of BChE increases. Consequently, cholinesterase inhibitors that suppress both AChE and BChE may provide a better therapeutic response rather than AChE-only selective agents [50,51,52].

## 2. Results and Discussion

### 2.1. Spectral Data

The lindoldhamine isomer (**1**) was isolated as a light yellow amorphous solid (3.6 mg). ^1^H NMR (500 MHz, CD_3_OD, TMS): δ 4.59 (t, 1H, 7.1 Hz, 1-H), δ 3.40 (m, 2H, 3-H), δ 3.03 and 3.07 (m, 2H, 4-H), δ 6.77 (s, 1H, 5-H), δ 3.82 (s, 3H, 6-OCH_3_), δ 6.48 (s, 1H, 8-H), δ 7.25 (d, 1H, 8.5 Hz, 10-H), δ 6.93 (d, 1H, 8.5 Hz, 11-H), δ 6.93 (d, 1H, 8.5 Hz, 13-H), δ 7.5 (d, 1H, 8.5 Hz, 14-H), δ 3.05 (m, 1H, 15-H), δ 3.36 (m, 1H, 15-H), δ 4.55 (t, 1H, 7.1 Hz, 1′-H), δ 3.49 (m, 2H, 3′-H), δ 2.98 and 2.99 (m, 2H, 4′-H), δ 6.72 (s, 1H, 5′-H), δ 3.84 (s, 3H, 6′-OCH_3_), δ 6.59 (s, 1H, 8′-H), δ 6.76 (s, 1H, 10′-H), δ 6.98 (d, 1H, 8.5 Hz, 13′-H), δ 6.99 (dd, 1H, 8.5 and 2.0 Hz, 14′-H), δ 3.05 (m, 1H, 15′-H), δ 3.25 (m, 1H, 15′-H) ppm. ^13^C NMR by HSQC (125 MHz, CD_3_OD, TMS): δ 56.1; 39.3; 24.9; 124.5; 111.4; 148.2; 55.2; 145.6; 113.1; 123.1; 131.1; 130.6; 117.5; 157.8; 117.5; 126.0; 39.3; 56.4; 39.4; 24.8; 124.3; 111.3; 148.2; 55.1; 145.7; 112.9; 123.0; 127.7; 121.9; 144.5; 148.8; 117.6; 126.0; 39.3 ppm (Table S1). MS (APCI+) *m*/*z* 569 [M+H]^+^: 552, 178. HRMS *m*/*z* 569.2674 (calc. for C_34_H_37_N_2_O_6_ *m*/*z* 569.2646, ∆_*m*/*z*__theor._= •4.8 ppm).

Stepharine (**2**) was isolated as a light brownish amorphous solid (22.1mg). ^1^H NMR (500 MHz, CD_3_OD, TMS): δ 7.01 (dd, 1H, 3 and 10 Hz, 12-H), δ 7.16 (dd, 1H, 3 and 10 Hz, 8 H), δ 6.89 (s, 1H, 3-H), δ 6.41(dd, 1H, 1.8 and 10 Hz, 11-H), δ 6.29 (dd, 1H, 1.8 and 10 Hz, 9-H), δ 4.72 (m, 1H, 6a-H), δ 3.82 (s, 3H, 2-OCH_3_), δ 3.70 (ddd, 1H, 1.5, 6.3 and 13 Hz, 5-H), δ 3.61 (s, 3H, 1-OCH_3_), δ 2.52 (dd, 1H, 6.6 and 12 Hz, 7-H), δ 2.42 (dd, 1H, 10.5 and 12 Hz, 7‘-H), δ 3.44 (ddd, 1H, 6.3, 11 and 13 Hz, 5-H), δ 3,02 and δ 3,00 (m, 2H, 4-H), δ 1.95 (s, 1H, NH) ppm. ^13^C NMR by HSQC (125 MHz, CD3OD, TMS): δ 153.63; 150.28; 112.15; 127.81; 126.74; 56.48; 55.37; 43.71; 59.95, 44.90; 43.71; 23.5 ppm. (Table S2). MS (APCI+) *m*/*z* 298 [M+H]^+^: 281, 266, 250, 235, 161. HRMS *m*/*z* 298.1461 (calc. for C_18_H_20_NO_3_ *m*/*z* 298.1438, ∆_*m*/*z*__theor._= −7.9 ppm).

Palmatine (**3**) was isolated as a yellow amorphous solid (8.7mg). ^1^H NMR (500 MHz, CD_3_OD, TMS): δ 7.08 (s, 1H, 1-H), δ 4.02 (s, 3H, 2-OCH_3_), δ 3.97 (s, 3H, 3-OCH_3_), δ 7.69 (s, 1H, 4-H), δ 3.30 (m, 2H, 5-H), δ 4.96 (m, 2H, 6-H), δ 9.79 (s, 1H, 8-H), δ 4.24 (s, 3H, 9-OCH_3_), δ 4.14 (s, 3H, 10-OCH_3_), δ 8.14 (d, 1H, 9.3 Hz, 11-H), δ 8.04 (d, 1H, 8.8 Hz, 12-H), δ 8.82 (s, 1H, 13-H) ppm. ^13^C NMR by HSQC (125 MHz, CD_3_OD, TMS): δ 110.6; 152.2; 57.7; 155.1; 57.2; 112.8; 121.9; 29.1; 58.5; 146.9; 136.6; 147.1; 63.2; 153.3; 58.5; 128.7; 125.1; 122.7; 121.8; 141.2; 131.5 ppm (Table S3). MS (APCI+) *m*/*z* 352 [M+H]^+^: 336, 322, 308, 278, 250. HRMS *m*/*z* 352.1616 (calc. for C_14_H_26_NO_9_ *m*/*z* 352.1602, ∆_*m*/*z*__theor._= −3.9 ppm).

5-*N*-methylmaytenine (**4**) was isolated as a light yellow amorphous solid (11.2 mg). ^1^H NMR (500 MHz, DMSO d_6_, TMS): δ 8.12 (t; 2H; *J*= 5.5 Hz, 1 and 10-NH), δ 7.56 (m, 2H, 5’-H and 5´´-H or 9´-H and 9´´-H), δ 7.54 (m, 2H, 9´-H and 9´´-H or 5´-H and 5´´ H), δ 7.55 (m, 2H, 6´-H, 6´´-H, 8´-H, 8´´-H), δ 7.40 (m, 2H, 7´-H and 7´´-H), δ 7.42 (m, 1H, 3´-H or 3´´-H), δ 7.38 (m, 1H, 3´´-H or 3´-H), δ 6.63 (d, 1H, 2.4 Hz, 2´-H or 2´´-H), δ 6.60 (d, 1H, 2.4 Hz, 2´´-H or 2´-H), δ 3.20 (m, 2H, 2-H), δ 3.18 (m, 2H, 9-H), δ 2.35 (m, 2H, 4-H), δ 2.32 (m, 2H, 6-H), δ 2.16 (s, 3H, 5-NCH_3_), δ 1.61 (m, 2H, 3-H), δ 1.47 (m, 2H, 8-H), δ 1.45 (m, 2H, 7-H). ^13^C NMR (125 MHz, DMSO d_6_, TMS): δ 165.32; 165.27; 135.43; 127.98; 127.94, 129.39; 129.83; 138.85; 138.88; 122.79; 122.83; 55.26; 57.12; 42.09; 37.58; 39.07; 27.28; 24.53; 27.48 ppm. (Table S4). MS (APCI+) *m*/*z* 420 [M+H]^+^: 202, 188, and 131. HRMS *m*/*z* 420.2669 (calc. for C_26_H_34_N_3_O_2_ *m*/*z* 420.2646, ∆_*m*/*z*__theor._= −5.6 ppm).

*N-trans*-feruloyltyramine (**5**) was isolated as a yellow amorphous solid (3.0mg). 1H NMR (500 MHz, CD3OD, TMS): δ 7.11 (d, 1H, 1.8 Hz, 2-H), δ 3.88 (s. 3H, 4-OCH_3_), δ 6.70 (m, 1H, 5-H), δ 7.03 (dd, 1H, 1.8 and 8.5 Hz, 6-H), δ 7.44 (d, 1H, 16 Hz, 7-H), δ 6.40 (d, 1H, 16 Hz, 8-H), δ 7.06 (m, 1H, 2′-H), δ 6.72 (m, 2H, 3′-H), δ 6.72 (m, 2H, 5′-H), δ 7.06 (m, 1H, 6′-H), δ 2.75 (m, 2H, 7′-H), δ 3.46 (m, 2H, 8′-H) ppm. ^13^C NMR by HSQC (125 MHz, CD_3_OD, TMS): δ 123.4; 111.1; 148.8; 150.2; 56.0; 115.7; 122.7; 141.5; 118.4; 169.3; 130.9; 130.2; 115.7; 157.1; 115.7; 130.2; 35.4; 42.1 ppm (Table S5). MS (APCI+) *m*/*z* 314 [M+H]^+^: 177, 145, 121. HRMS *m*/*z* 314.1404 (calc. for C_18_H_20_NO_4_ *m*/*z* 314.1387, ∆_*m*/*z*__theor._= −6.3 ppm).

The MS and NMR spectra of the alkaloids are found in the Supplementary Materials.

The molecular formula of the compound **1** was determined by HRMS in the positive mode as C_34_H_37_N_2_O_6_, indicating the same molecular formula as that of lindoldhamine. The ^1^H NMR and HSQC data (Figures S1 and S3) revealed eleven aromatic methines (7.3–6.5 ppm); two aliphatic methines at δ_H_ 4.59 (t; 7.1 Hz; H-1) and δ_H_ 4.55 (t; 7.1 Hz; H-1′); six aliphatic methylene groups (3.5–2.0 ppm); two -OCH_3_ groups connected to the aromatic rings; and four aromatic proton singlets at δ_H_ 6.59 (s; H-8′), δ_H_ 6.48 (s; H-8), δ_H_ 6.72 (s; H-5′), and δ_H_ 6.77 (s; H-5) ppm. The NMR data featured a 1,2,4-trisubstituted and a 1,4-disubstituted benzene moieties [H-10′ (δ_H_ 6.76), H-13′ (δ_H_ 6.98), H-14′ (δ_H_ 6.99) and H-13 (δ_H_ 6.93), H-14 (δ_H_ 7.25) *J* = 8.1 Hz] of a AA′XX′ spin system.

The HMBC (Figure S5) spectrum shows six quaternary carbons. The presence of C7–O–C11′ bond was supported by the HMBC spectrum according to the characteristics observed in the chemical environment of the carbons in question δ_C_ 144.5 (C-11’) and δ_C_ 145.6 (C-7). The data suggest that the compound **1** is more likely an isomer of lindoldhamine with head-to-tail bonding.

The MS/MS (Figure S7) spectrum of the compound **1** is in agreement with the above NMR data. The ion *m*/*z* 569 showed a loss of 17 Da (*m*/*z* 552) attributed to that of a hydroxyl [M-OH]^+^ in a **C** ring of the bisbenzyltetrahydroisoquinoline skeleton. The peak at *m*/*z* 178 can be considered as a diagnostic ion in the skeleton of benzyldihydroisoquinoline alkaloids [12,22,53]. Therefore, the compound **1** was identified as an isomer of lindoldhamine.

The molecular formula of the compound **2** was determined by HRMS in a positive mode as C_18_H_20_NO_3_. The MS/MS spectrum of the *m*/*z* 298 ion showed sequential losses of 17 Da (*m*/*z* 281) and 15 Da (*m*/*z* 266), and a loss of 31 Da (*m*/*z* 281 → 250) (Figure S16); which are consistent with aporphine alkaloids containing adjacent methoxyls in the ring **A** and a non-substituted ring **D** [54,55].

The proposed structure was further confirmed by NMR spectroscopy. The ^1^H NMR spectrum of **2** exhibited signals typical for proaporphine alkaloids with three aliphatic methylenes at δ_H_ 2.52 (dd; 6.6 and 12 Hz; H-7 or H-7’), δ_H_ 2.42 (dd; 10.5 and 12 Hz; H-7’or H-7), δ_H_ 3.02 (m; H-4), δ_H_ 3.44 (ddd; 6.3, 11, and 13 Hz; H-5 or H-5’) and δ_H_ 3.70 (ddd; 1.5, 6.3, and 13 Hz; H-5’or H-5). In the range of aromatic proton resonances, the following signals were observed: δ_H_ 6.89 (s, H-3) corresponding to an *ortho*-substituted ring **A**; δ_H_ 7.16 (dd; 3 and 10 Hz; H-8), δ_H_ 6.29 (d; 1.8 and 10 Hz; H-9), δ_H_ 6.41 (dd; 1.8 and 10 Hz; H-11), and δ_H_ 7.01 (dd; 3 and 10 Hz; H-12) characteristic of the unsubstituted ring **D**; and two signals δ_H_ 3.61 (s, 3H) and δ_H_ 3.82 (s, 3H) of the methoxy-substituents in the ring **A** (Figure S9).

In agreement with the above, the HMBC experiments (Figure S14) demonstrated signals at δ_H_ 7.16 (dd; 3 and 10 Hz; H-8) and δ_H_ 7.01 (dd; 3 and 10 Hz; H 12) having a *J*^3^-coupling to the carbon at δ_C_ 186.6 (C-10). The proaporphine skeleton was also established following long-distance ^1^H-^13^C couplings of the signals at δ_H_ 6.89 (s, H-3) and those of the methoxyls at δ_H_ 3.61 (s, 3H) and δ_H_ 3.82 (s, 3H) with the carbons at δ_C_ 144.6 (C-1) and δ_C_ 154.7 (C-2), thus, confirming the existence of two substitutions in the **A** ring. Therefore, the compound (**2**) was elucidated as being the proaporphine alkaloid stepharine.

The molecular formula of the compound **3** was determined by HRMS in positive mode as C_14_H_26_NO_9_. The MS/MS spectrum of the *m*/*z* 352 ion showed the main loss of 16 Da (*m*/*z* 352→336) [M- CH_3_– H]^+^ characteristic of the protoberberine alkaloids [37,53]. The ^1^H NMR and HMBC spectra (Figures S18 and S22) of the compound **3** exhibited signals typical for protoberberine alkaloids: nine quaternary carbons; four aromatic proton singlets corresponding to a para substituted ring viz., δ_H_ 7.69 (s; H-4) and δ_H_ 7.08 (s; H-1) corresponding to the ring **A** and δ_H_ 9.79 (s; H-8) and δ_H_ 8.82 (s; H-13) to the ring C. The signals at δ_H_ 8.14 (d; 9.3 Hz; H-11) and δ_H_ 8.04 (d; *J*: 8.8 Hz; H-12) were assigned to the ortho substituted ring **D**. The structure also contained two aliphatic methylenes at δ_H_ 3.30 (m H-5) and δ_H_ 4.96 (m; H-6); four methoxy-groups connected to the aromatic rings δ_H_ 4.24 (s; 9-OCH_3_), δ_H_ 4.14 (s; 10-OCH_3_), δ_H_ 4.02 (s; 2-OCH_3_), and δ_H_ 3.97 (s; 3-OCH_3_). Thus, the compound **3** was identified as the protoberberine alkaloid palmatine.

The molecular formula of the compound **4** was determined by HRMS in the positive mode as C_26_H_34_N_3_O_2_. The ^1^H NMR data of the compound **4** revealed the presence of two dehydroxylated cinnamic acid moieties characterized by signals of unsubstituted aromatic rings at δ_H_ 7.40 (m, H-7’and H-7″) and δ_H_ 7.54–7.56 (m, H-5’; H-5″; H-6’; H-6″; H-8’; H-8″; H-9’; H 9″) as well as the signals of methylene protons at δ_H_ 7.38 (m, H-3″ or H-3’), δ_H_ 7.42 (m, H-3’or H 3″), δ_H_ 6.60 (d, H-2’or H-2″), and δ_H_ 6.63 (d, H-2″ or H-2’). Signals of proton resonances in the aliphatic region (δ_H_ 1.45–3.20 ppm) were also elucidated (Figure S26). In the aliphatic region of the ^13^C NMR spectra (Figure S28), the presence of a methyl (δ_C_ 42.09) and seven methylene carbons (δ_C_ 24–55 ppm) was confirmed by DEPT-135 (Figure S36). Additionally, the HMBC spectra (Figure S32) revealed two carbonyls in the structure and showed correlations of the -NH group (δ_H_ 8.12) with the above-mentioned carbonyl signals (δ_C_ 165.27 and δ_C_ 165.32), thus confirming the presence of two cinnamamide moieties. The methyl group (δ_C_ 42.09) directly linked to the nitrogen in the aliphatic chain can be confirmed in the HMBC with the long-distance proton couplings between the δ_H_ 2.16 (s, NCH_3_) and the methylene carbons at δ_C_ 55.26 (C-4) and δ_C_ 57.12 (C-6).

The MS/MS spectrum of the compound **4** is in agreement with the above NMR data. The ion *m*/*z* 420 showed sequential losses of 218 Da (*m*/*z* 202), 14 Da (*m*/*z* 188), and 57 Da (*m*/*z* 131) (Figure S38), typical for the fragmentation pattern of cinnamic acid amides [56,57]. Therefore, the compound was identified as 5-*N*-methylmaytenine or 1,10-di-E-cinnamamide of 5 *N*-methylspermidine.

The molecular formula of the compound **5** was determined by HRMS in the positive mode as C_18_H_20_NO_4_. In the MS/MS spectrum of the compound, the ion *m*/*z* 314 showed main losses of 137 Da (*m*/*z* 314→177), 32 Da (*m*/*z* 177→145), and 56 Da (*m*/*z* 145→121), typical for the fragmentation pattern of cinnamic acid amides [58,59] (Figure S46). 

The presence of two substituted-aromatic rings in the structure of **5** was suggested upon analysis of the ^1^H NMR spectrum. One of them gave rise to signals at δ_H_ 7.11 (d; 1.8 Hz; H-2), δ_H_ 7.03 (dd; 1.8 and 8.5 Hz; H-6), and δ_H_ 6.70 (m; H-5). The resonances at δ_H_ 7.06 (m; H-2′ and H-6′) and δ_H_ 6.72 (m; H-3′ and H-5′) correspond to the other aromatic ring. The signals at δ_H_ 7.44 (d; 16 Hz; H-7) and δ_H_ 6.40 (d; 16 Hz; H-8) indicated a presence of a *trans-* double bond. Besides that, two methylene groups at δ_H_ 2.75 (m; H-7′) and δ_H_ 3.46 (m; H-8′) and one methoxy-group at δ_H_ 3.88 (s; 4-OCH_3_) were also found (Figure S40). According to the HMBC experiment, δ_H_ 7.44 (d; 16Hz; H-7) correlated at two and three bonds with the carbon signals at δ_C_ 123.4 (C-1), δ_C_ 111.1 (C-2), and δ_C_ 169.3 (C-9) indicating the presence of a trans-feruloyl moiety (Figure S44). The signal of δ_H_ 2.75 (m; H-7’) correlated with the signals at δ_C_ 130.9 (C-1′) and δ_C_ 42.1 (C-8′) that is characteristic to the presence of ethylbenzene moieties. Additionally, the signal δ_H_ 3.46 (H-8’) was assigned to a proton of the imino-group in a tyramine moiety. Coupling of the H-8’ signal with those at δ_C_ 130.9 (C-1’) and δ_C_ 169.3 (C-9) observed in the HMBC enabled us to identify the compound as *N-trans*-feruloyltyramine.

All compounds were first isolated by us from *A. panurensis* either previously (**2**, **4**) [10] or in the present work (**1**, **3**, **5**). They were identified by a comparison of the obtained spectral results with the data reported in the literature. The isomer of lindoldhamine alkaloid **1**, and the *N*-*trans*-feruloyltyramine **5** were found for the first time in *Abuta* genus, while palmatine **3** was isolated for the first time from *A. panurensis*.

### 2.2. Acetylcholinesterase Inhibition Assay

The inhibitory activity of the lindoldhamine isomer, stepharine, palmatine, 5-*N*-methylmaytenine, and neostigmine (positive control) toward the AChE enzyme showed the inhibition percentage above 70%, whereas *N-trans*-feruloyltyramine was practically inactive (the inhibition percentage 19.88 ± 0.09%). The alkaloids isolated from *A. panurensis* revealed promising IC_50_ values of 19.55–61.24 µM, the neostigmine (positive control) is an efficient short-term reversible inhibitor of the AChE enzyme that demonstrated the IC_50_ of 3.72 µM (Table 1).

### 2.3. Binding with Acetylcholinesterase of Tetronarce californica (TcAChE)

The X-ray structure of the AChE from the electric ray *T. californica* was found in the RCSB Protein Data Bank under 6H12 code. The analysis showed that the majority of ligands were stable within the simulation and did not change their position with respect to their induced fit docking (IFD) pose.

**Figure 2:**
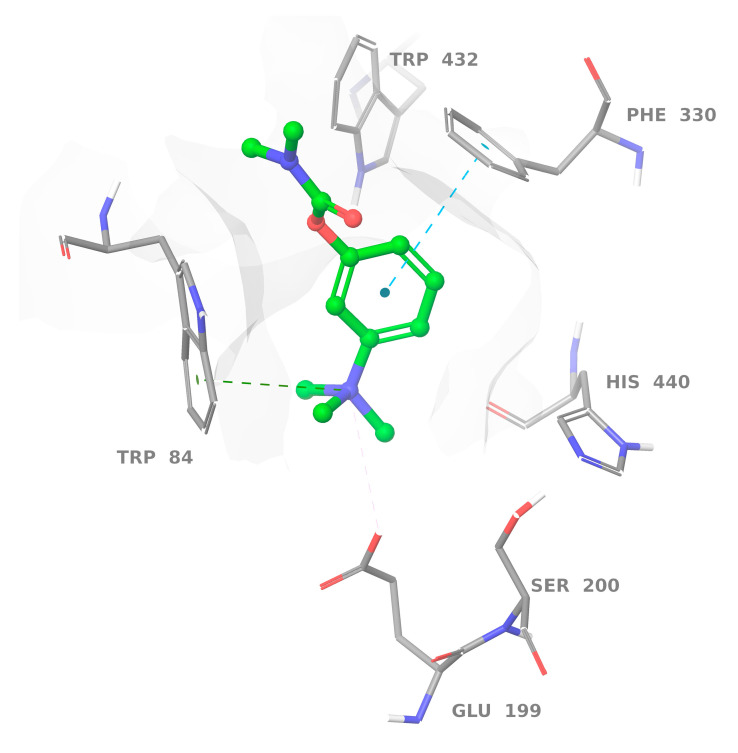
Protein-ligand interactions observed between acetylcholinesterase (AChE) and neostigmine in the molecular dynamics (MD) calculations. Interactions shown correspond to those occurring more than 30% of the simulation time in the trajectory.

Neostigmine was used as a positive control in the AChE binding assay. Results of molecular dynamics (MD) calculations for this ligand revealed a pose similar to that proposed previously by our docking studies [10]. The benzene ring of neostigmine forms a stacking interaction with Phe330, while an ammonia cation forms a salt bridge with the carboxyl group of Glu199 and π-cation interaction with Trp84 at the anionic site (Figure 2).

**Figure 3:**
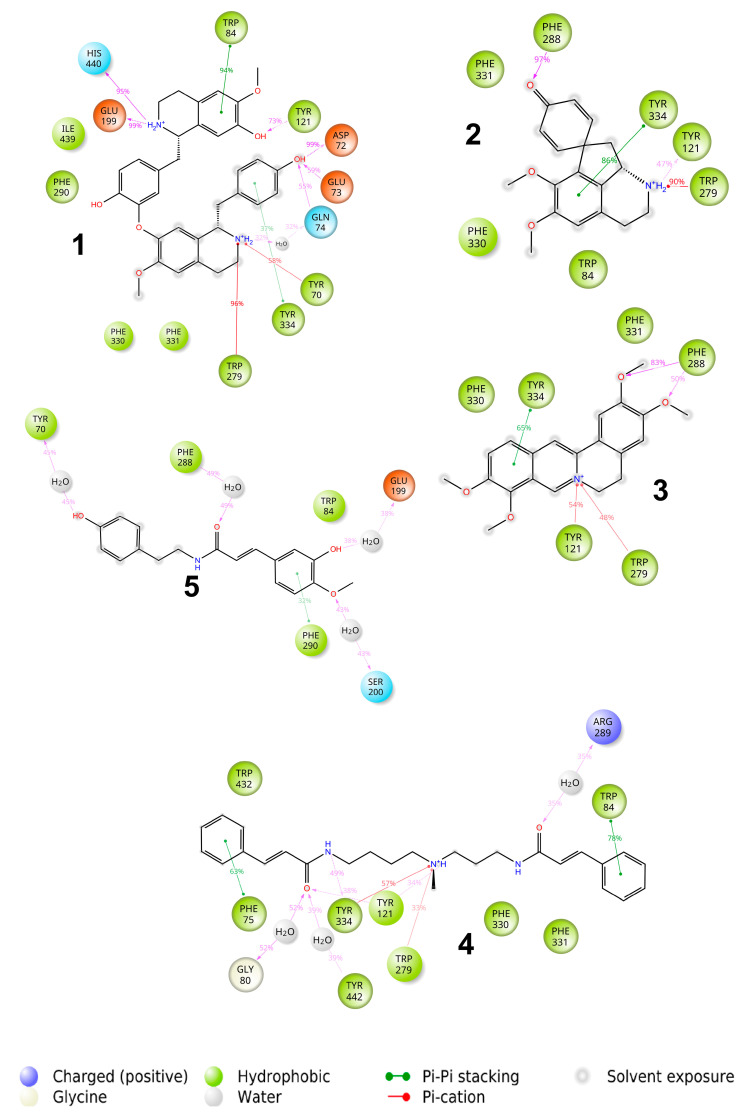
Protein–ligand interactions observed between acetylcholinesterase (AChE) and studied ligands in the molecular dynamics (MD) calculations. Interactions shown correspond to those occurring more than 30% of the simulation time in the trajectory. (**1**) Lindoldhamine isomer, (**2**) stepharine, (**3**) palmatine, (**4**) 5-*N*-methylmaytenine, and the (**5**) *N-trans*-feruloyltyramine.

The docking and further MD study revealed that all active ligands with positively charged ammonia nitrogen formed a stable π-cation and π–π interactions with one of the aromatic amino acids of the external part of the AChE binding pocket (see Figure 3).

The lindoldhamine isomer **1** (see Figure 3) has the highest binding affinity to AChE of −115 kcal/mol, whereas other ligands show similar affinities in the range of −70 to −80 kcal/mol (Table 1). This can be explained by the presence of 4 aromatic rings and two charged ammonia nitrogens in the molecule possessing quite high conformational mobility. Surprisingly, despite the overall aromatic character of the binding pocket, only one stacking interaction with Trp84 remained stable throughout the MD run. Instead, the π-cation interactions and hydrogen bonds with -OH groups were predominantly responsible for the binding of **1**. Moreover, the compound also formed a steady ionic contact with the Glu199 moiety of the AChE.

Stepharine **2** (see Figure 3) forms one stacking interaction with Tyr334, a π-cation interaction with Trp279, and hydrogen bonds with Phe288 and Tyr121. Quite similar interactions were formed between palmatine **3** and AChE viz., stacking with Tyr334, hydrogen bonds between -OMe groups and Phe288, and π-cation interactions with Tyr121 and Trp279. Rigidity of the structure did not allow it to attain a more favourable binding pose.

*N*-methylmaytenine **4** forms three stacking interactions with Phe75, Trp84, and Tyr334, a π-cation interaction with Tyr121, and several hydrogen bonds with protein residues and surrounding water molecules. The structure and conformational mobility of **4** led to the second-highest affinity in the series under investigation.

Only one phenyl ring of *N*-*trans*-feruloyltyramine **5** formed stacking interaction with Phe290, and hydrogen bonds were formed only with the surrounding water molecules leading to a relatively low binding affinity.

The molecules **1**, **2**, and **4** have substantial π–π contacts with buried Trp84 and Phe330 moieties. That fact corroborates with known binding modes of other AChE inhibitors, e.g., anti-TZ2-PA6 [60], MC1420 [61], and a 6-chlorotacrine-based triazole derivative [62].

In general, we observe better experimental IC_50_ values for more conformationally flexible molecules. 5-*N*-methylmaytenine **4** has the highest count of rotatable bonds among the series and the lowest IC_50_ value. The binding affinity of the less flexible lindoldhamine isomer **1** is slightly lower according to the experimental results. The most rigid ligand stepharine **2** has both the lowest molecular mechanics/generalized Born surface area continuum solvation method (MM/GBSA) energy and experimental activity. Despite possessing a quite rigid scaffold, palmatine **3** showed a good correlation of the predicted binding activity with the experimental IC_50_ values. Presumably, the aromatic character of the structure allows it to form favorable hydrophobic interactions, and that may be one of the reasons explaining the inactivity of *N*-*trans*-feruloyltyramine **5**. It lacks π-cation interaction with the AChE aromatic residues due to the absence of charged ammonia nitrogen in the structure. In line with the described trend, neostigmine has low predicted binding affinity. Nevertheless, it shows the strongest acetylcholinesterase inhibition. MM/GBSA is known to successfully rank binding affinities [63]; however, a limited number of alkaloids isolated from *A. panurensis* along with close values of the predicted energies for some complexes might affect the ranking accuracy.

### 2.4. Binding with Human Butyrylcholinesterase (BChE)

**Figure 4:**
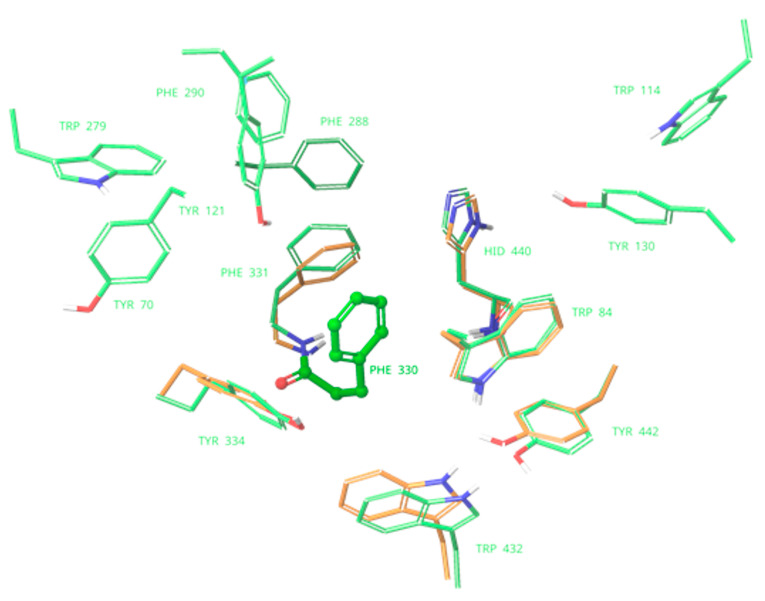
Comparison of the binding pockets of acetylcholinesterase of *Tetronarce californica* (TcAChE) (green) and butyrylcholinesterase (BChE) (red). Only aromatic residues are shown. Labels are provided solely for the residues of acetylcholinesterase. A greater number of aromatic residues in TcAChE binding site is easily observed.

The X-ray structure of human BChE was retrieved from the RCSB Protein Data Bank under 6EP4 code [64]. The binding site of the BChE contains only four aromatic residues compared to ten in the binding pocket of AChE. (Figure 4). Therefore, interactions of aromatic molecules with the BChE binding site could be slightly weaker (Table 1).

**Figure 5:**
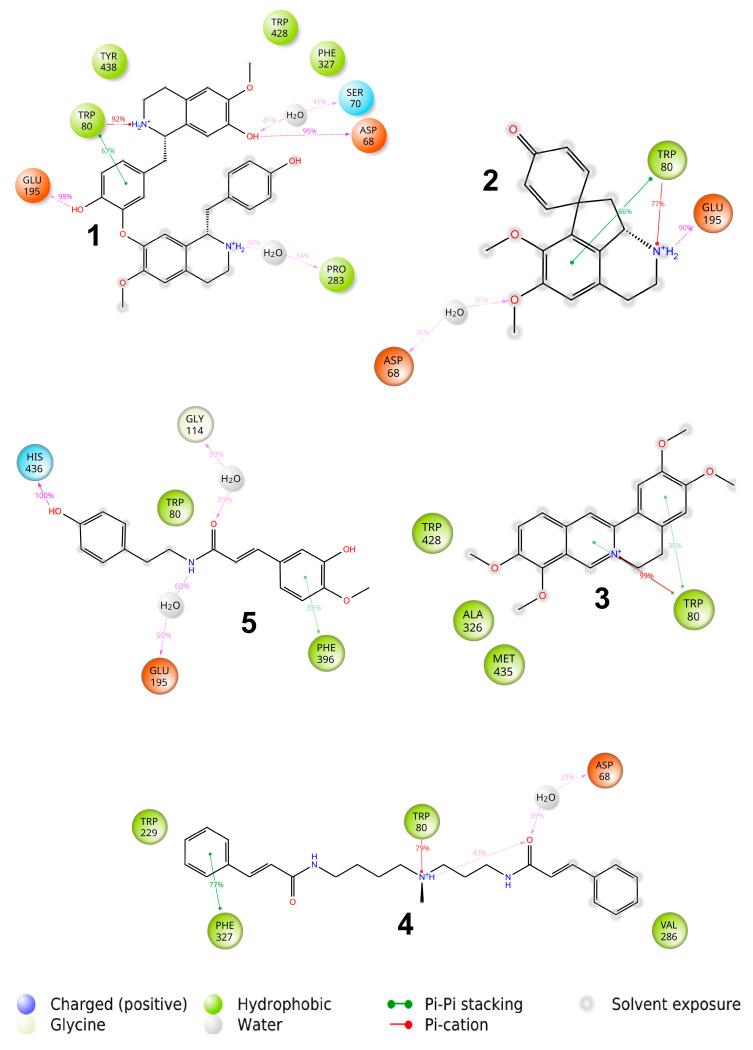
Protein–ligand interactions observed between butyrylcholinesterase (BChE) and studied ligands in the molecular dynamics (MD) calculations. Interactions shown correspond to those occurring more than 30% of the simulation time in the trajectory. (**1**) Lindoldhamine isomer, (**2**) stepharine, (**3**) palmatine, (**4**) 5-*N*-methylmaytenine, and the (**5**) *N-trans*-feruloyltyramine.

The majority of compounds under investigation form stacking and π-cation interactions with Trp80 at the choline-binding pocket of the acylation site as well as hydrogen bonds with deeply buried and highly conserved Glu195 residue (see Figure 5). At the peripheral site, the Asp68 forms hydrogen bonds with some of the alkaloids in question, either directly (lindoldhamine isomer **1**) or through a water molecule (stepharine **2** and 5-*N*-methylmaytenine **4**). The *N*-feruloyltyramine **5** is the only ligand with a different interaction pattern: it stretches between the acyl- and choline-binding pockets and forms a weak π-stacking with Phe396 and a hydrogen bond between OH-group and a His436 moiety. The larger predicted binding energy of palmatine **3** with BChE compared to AChE can be explained by the different strength of the π-stacking interactions in both complexes: the one with the Trp80 moiety of BChE was preserved during the MD run, whereas stacking with the Tyr334 in the AChE complex was more labile, according to the MD simulation.

Despite fewer aromatic residues in the binding pocket of BChE compared to AChE, calculated binding affinities do not differ substantially. Results of the MD calculations show that polar interactions, including hydrogen bonds and π-cation interactions, survive more readily than hydrophobic ones. As there is no clear difference in the number and strength of polar interactions formed with BChE compared to AChE, the binding affinities to those receptors have close values.

Currently, natural products constitute one of the main sources of the AChE inhibitors used as active compounds to treat central and peripheral nervous system damages as well as to alleviate symptoms of neurodegenerative diseases [14,65,66]. The alkaloids isolated by us from *A. panurensis* belong to several different groups, such as bisbenzyltetrahydroisoquinoline (**1**), proaporphine (**2**), protoberberine (**3**), polyamine (**4**), and amide alkaloids (**5**). According to the literature, bisbenzyltetrahydroisoquinoline and protoberberine alkaloids exhibit a moderate AChE enzyme inhibition potential with IC_50_ values in the range of 34.66 µM to 78.22 µM [11] and 36.6 µM to 141.8 µM [41], respectively. Whereas aporphine and proaporphine alkaloids demonstrate better AChE inhibitory activity with the IC_50_ values ranging from 2.98 µM to 20.4 µM and this effect is often related to different substituents in their structure [15]. Polyamine alkaloids such as putrescine, spermidine, spermine, cadaverine, and their derivatives are present ubiquitously in all living cells [67]. In particular, they can act on receptors of the central nervous system related to neurodegenerative processes [68,69]. For example, spermidine decreases significantly the AChE activity, oxidative stress, and neuroinflammation in a cerebral hippocampus [70]. According to a previous report [49], *N-trans*-feruloiltyramine was inactive towards the AChE enzyme, the results of the present study confirm those findings, the alkaloid did not reach 20% of the enzyme inhibition. Taking into a consideration the values of IC_50_ determined for the alkaloids under investigation together with a comparison of those with the IC_50_ values reported in the literature for the same groups of alkaloids, the compound **4** appears to be the most promising, because it showed the lowest IC_50_ value.

Beyond a regulation of synaptic acetylcholine levels, BChE also plays an important role in neurodegenerative disease progression [51,71]. With respect to the BChE inhibition, the bisbenzylisoquinoline [11,72,73] and protoberberine [74,75] alkaloids are usually more potent inhibitors of BChE than AChE. This fact is in a good agreement with the Gibbs free energies of binding in protein–ligand complexes found here by MD simulations for **1** and **3**. The aporphine alkaloids may inhibit both AChE and BChE; preferential activity toward one enzyme or another strongly depends on a particular alkaloid structure [76,77].

## 3. Materials and Methods 

### 3.1. Chemicals

Reagents and HPLC-grade solvents were purchased from Tedia Company (Fairfield, OH, USA) and Merck KGaA (Darmstadt, Germany) and used as supplied. P.A. (nuclear)-grade solvents were purified by standard procedures used in natural products chemistry. An ultrahigh-purity water was obtained by Milli Q system (Millipore, Bedford, MA, USA).

### 3.2. Plant Material

The authors declare that a specific permission from the National Institute of Amazonian Research (INPA, Manaus, AM, Brazil) was required to collect the plant material. The authors got the permission No. 35/12 of 2 December 2017 and confirm that the study did not involve endangered or protected species. The plant material of *A. panurensis* was collected at Adolpho Ducke Forest Reserve, 26 km along the AM-010 highway from the city of Manaus, the State of Amazonas, Brazil. The species were identified by the taxonomist L. S. Mergulhão. The voucher specimens were deposited in the INPA Herbarium under the voucher number 279373. The access to genetic heritage was registered at the National System of Genetic Heritage and Associated Traditional Knowledge Management (SisGen) under the code number A9CC956.

The branches collected were dried at room temperature (ca. 20 °C) for 10 days. Subsequently, the vegetal material (1.4 kg of branches) was crushed in a knife mill and stored in a refrigerator until use.

### 3.3. Extraction

Dried and crushed plant material was subjected to an acid-base extraction [54]. The crushed branches (300 g) were macerated with a mixture of 10% solution of NH_4_OH (2 L) and CH_2_Cl_2_ (2 L) at room temperature (20 °C) for 72 h. The material was stirred every 24 h. The organic phase (1.5 L) was transferred to a separatory funnel with a 10% solution of acetic acid (2 L) and shacked manually. Then, the acidic aqueous phase was transferred to another vessel and the pH was adjusted to 10.0 by using the NH_4_OH solution, and extracted with CH_2_Cl_2_ (2 × 300 mL). The organic phase was separated, concentrated on a rotary evaporator under reduced pressure, and dried with anhydrous sodium sulfate, resulting in the alkaloid fraction (280 mg).

### 3.4. HPLC-APCI-MS Analysis

HPLC-APCI-MS analyses were performed on an Acella chromatograph (Thermo Scientific, Waltham, MA, USA) coupled to a triple-quadrupole mass spectrometer model TSQ Quantum Acess^®^ (Thermo Scientific), equipped with an atmospheric pressure chemical ionization (APCI) source, and operated in positive mode with monitoring in the range of *m*/*z* 100–800. The instrument was equipped with a Surveyor LC Pump Plus, a Surveyor Autosampler Plus, a Rheodyne injection valve (25 μL), and a Luna C18 column (150 × 4.60 mm, 5 μm) (Phenomenex, Torrance, Torrance, CA, USA), and the mass spectrometer was operated simultaneously with a Surveyor PDA Plus diode array detector (DAD). The mobile phase was composed of B (methanol) and A (formic acid 1% *v*/*v* in H_2_O) with a linear elution gradient (**1**): 0–14 min 20–80% B, 20–30 min 80% B, and a linear elution gradient (**2**): 0–20 min 20–80% B, 20–35 min 80% B, 35–45 min 20–80% B. The flow rate of the mobile phase was 1 mL/min and the injection volume was 10 μL. The DAD detector was set up for monitoring between 200 and 400 nm. The spectra were processed by using Xcalibur software (version 2.2).

### 3.5. Semi-Preparative HPLC Analysis

Isolation of the alkaloids was performed on a semi-preparative scale on a Shimadzu chromatograph composed of a CBM-20A communication module, an SPD-20AV UV detector, a DGU-20A5 degasser, an LC-6AD pump, a 200-μL Rheodyne injection valve, and a Luna C18 column (250 × 15.00 mm, 5 μm) (Phenomenex, Torrance, CA, USA) with a flow rate of 3 mL/min. The mobile phase was composed of B (methanol) and A (formic acid 1% *v/v* in H_2_O) with a linear elution gradient (**1**): 0–14 min 20–80% B, 20–30 min 80% B, and a linear elution gradient (**2**): 0–20 min 20–80% B, 20–35 min 80% B, 35–45 min 20–80% B. The UV detector was set to monitoring at 260 nm and 280 nm. The gradient (**1**): fractions containing lindoldhamine isomer (3.6 mg), stepharine (22.1 mg), and palmatine (8.7 mg); the gradient (**2**): fractions containing 5-*N*-methylmaytenine (11.2 mg) and *N-trans*-feruloyltyramine (3.0 mg) were collected and analyzed by high-resolution mass spectrometry (HRMS) and NMR spectroscopy.

### 3.6. High-Resolution Mass Spectrometry

HRMS analyses were performed on a Shimadzu chromatograph composed of a CBM-20A communication module, an SPD-20AV UV detector, an LC-20AD pump, a SIL-20A HT autosampler (200 μL), a CTO-20A oven, and a Luna PFP column (150 × 2 mm, 100 A) coupled to a Bruker microTOF-QII mass spectrometer, equipped with an APCI source, operating in a positive mode. The instrument parameters were as follows: capillary voltage, 4500 V; nebulizer pressure (N_2_), 4.0 bar; dry gas flow (N_2_), 8 L/min; dry heater temperature, 200 °C; with a monitoring in the range of *m*/*z* 100–800. The mobile phase was composed of B (formic acid 0.1% *v/v* in methanol) and A (formic acid 0.1% *v/v* in H_2_O) with a linear elution gradient as follows: 0–2 min 20–80% B; 2–42 min 100% B. The flow rate of the mobile phase was set to 0.2 mL/min and the injection volume was 10 μL. The UV detector was set up for monitoring between 254 nm and 330 nm. The spectra were processed by using Bruker Compass Data Analysis software (version 4.2).

### 3.7. 1D and 2D NMR Spectroscopy

NMR spectra were recorded on a Bruker Avance IIIHD spectrometer operated at a magnetic field strength of 11.7 Tesla at 500.13 MHz for ^1^H and 125.0 MHz for ^13^C, equipped with a 5-mm direct detection PA BBO BBF HD-05-Z SP Intelligent probe incorporating a Z-axis gradient coil capable of providing gradient amplitudes up to 50 G/cm. Shigemi’s 5.0-mm NMR tubes were used. For structural elucidation, the samples of lindoldhamine isomer, stepharine, palmatine, and *N-trans*-feruloyltyramine were solubilized in 600 µL of CD_3_OD (δ_H_ 3.34, δ_C_ 49.8); 5-*N*-methylmaytenine was solubilized in 600 µL of DMSO-d_6_ (δ_H_ 2.50, δ_C_ 39.9). The acquisition of ^1^H, ^13^C, DEPT 135, COSY, HSQC, and HMBC spectra was performed by using standard Bruker pulse sequences. The analysis based on ^1^H NMR data were performed by solubilizing an amount of the samples in 550 µL of CD_3_OD or DMSO-d_6_ with 50 µL of TMS (0.5 mM, 98%, Tokyo Chemical Industry, Tokyo, Japan) at 25 °C. Acquisition of the spectra was performed by using the zg30 pulse sequence with water suppression signal, data points of the 64 kB time domain, 10 kHz spectral width, 1.00 s relaxation delay (D1), 3.27 s acquisition time (AQ), 32 (lindoldhamine isomer, stepharine, 5-*N*-methylmaytenine and *N-trans*-feruloyltyramine) and 16 (palmatine) scan numbers with DS of 2, decomposition resolution of 0.31 Hz, a constant receiver gain at 114 (palmatine), 161 (5-*N*-methylmaytenine and *N-trans*-feruloyltyramine), 181 (stepharine) and 203 (lindoldhamine isomer) with displacement frequency set at 2425.23 Hz, PLW1 of 20.3 W. The calibration pulse (P1 9.400 µs to 5-*N*-methylmaytenine and P1 10.300 µs to lindoldhamine isomer, stepharine, palmatine, and *N-trans*-feruloyltyramine) with PLW9 were of 7.183 × 10^−5^ W (5-*N*-methylmaytenine) and 8.6243 × 10^−5^ W (lindoldhamine isomer, stepharine, palmatine and *N-trans*-feruloyltyramine). The data obtained were processed by using Bruker^®^ Topspin 4.0.6 software.

### 3.8. Docking and MD Procedures

Missing residues Ala536-Phe565 in the published structure of AChE (PDB ID: 6H12) [60] and the the residues Val377-Gln380 in the published structure of BchE (PDB ID: 6EP4) [64] were modelled by using Modeller web service [78]. Then, completed AChE and BchE structures were prepared by employing the Protein Preparation Wizard of the Schrödinger package [79,80,81,82], and all the water and other small molecules and ions were removed. Conformations of the alkaloids **1–5** and their ionization states were determined by using LigPrep [83] at pH 7.4 by using Epik [80] force field OPLS3e [84].

**Figure 6:**
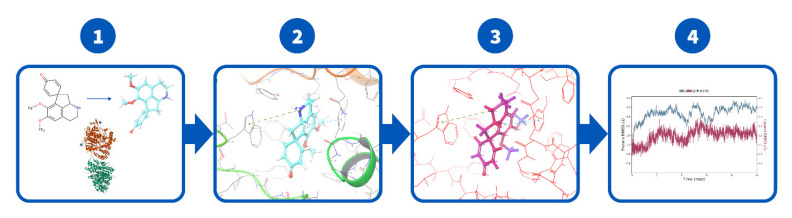
Schematic road map of the modeling study. **1st step**—modeling and preparation of proteins, 2D to 3D. **2nd step**—Induced Fit Docking (IFD) at the active site. **3rd step**—rescoring with molecular mechanics generalized Born surface area (MM/GBSA). **4th step**—molecular dynamics simulation of the highest scored poses and trajectory analysis.

The calculations proceeded as shown in Figure 6. First, the ligands were docked by using the Induced Fit Docking (IFD) protocol of the Schrödinger software package [82,85]. In contrast to the standard docking protocols that assume a rigid receptor structure, the IFD tailors both a receptor (including a backbone) and a ligand structure to allow for the best binding affinity [82,85,86,87]. The top five poses for each ligand were further analyzed for binding free-energy perturbations by the molecular mechanics method by using Prime MM/GBSA (molecular mechanics energies combined with the generalized Born and surface area continuum solvation) [82]. The poses with higher MM/GBSA binding energy were chosen; these are referred to as binding affinities throughout the text.

Finally, to verify the stability of the binding poses, we conducted 10 ns molecular dynamic simulations by employing a Desmond package of the Schrodinger one [88,89]. Simulation conditions: explicit water TIP4PEw, 0.15M NaCl, T = 300 K, forcefield OPLS4. The binding poses and interactions of the protein–ligand complexes were analyses using the MD calculation results. The MD simulation reports generated by Schrodinger are available in the supporting information.

### 3.9. Acetylcholinesterase Inhibition Assay

An acetylcholinesterase enzyme from an electric ray *Tetronarce californica* (Sigma-Aldrich, St. Louis, MO, USA) was used for the experiments. The in vitro AChE inhibition assay was performed in 96-well microplates according to the methodology proposed by Ellman et al. [90] and Senol et al. [91] with some modifications. The alkaloid fraction and alkaloids were tested at concentrations of 2.8; 5.6; 11.2; 22.5; 45.0 and 90.0 µg/mL. Initially, 20 µL of each sample from the stock solution (1 mg/mL) were added and serial dilutions were performed. Then, 150 µL of a sodium phosphate buffer pH = 8 (0.1 mM), 20 µL of 5,5’-dithio-bis(2- nitrobenzoic)acid (DTNB, 0.0025 M), and 20 µL of the acetylcholinesterase enzyme (1 U/mL) were added subsequently to each well at 25 °C and left for 15 min. The reaction was initiated by the addition of 10 µL of acetylcholine iodide (AChI) (0.1 M). Neostigmine (0.28–9.0 μg/mL) was used as a positive control.

A thiocholine formed by the enzymatic hydrolysis of the AChI, reacts with the DTNB giving rise to a yellow 5-mercapto-2-nitrobenzoate anion. Concentration of the latter in each well was measured as absorbance at 405 nm by using a 96-well microplate reader ELx800^TM^ (Bio Tek Instruments, Inc., Winooski, VT, USA). The transformation was monitored for 30 min at 5-min intervals. The inhibition curve was plotted as the inhibition percent vs. concentration. All assays were performed in triplicate. Statistical analysis was performed with GraphPad Prism 7.0^®^ software.

## 4. Conclusions

This is the first report on the isolation of the lindoldhamine isomer **1**, palmatine **3**, and the *N-trans*-feruloyltyramine **5** from *A. panurensis*, and two other alkaloids, stepharine **2** and 5-*N*-methylmaytenine **4**, that we isolated earlier from the same species. Inhibitory activity of those alkaloids towards the AChE enzyme was evaluated by spectrophotometry and a molecular docking study. The compounds in question bind effectively to the enzyme active site and demonstrate promising inhibitory potential, except the compound **5** which was inactive. Alkaloids **1–4** were capable of interacting with both anionic and peripheral subsites, thus demonstrating higher AChE inhibition potential. Our results suggest that the alkaloids **1–4** could be used as reversible AChE inhibitors in the treatment of neurological disorder manifestations. However, more research is necessary to better investigate the complete pharmacological potential and toxicological profile of these compounds. Gibbs free binding energies in the BChE-alkaloid complexes assessed by MD simulations suggest that lindoldhamine isomer (**1**) and palmatine (**3**) are the most promising BChE inhibitors among the series investigated.

## Figures and Tables

**Table 1:** Gibbs free binding energies of enzyme-alkaloid complexes, kcal/mol (MM/GBSA—molecular mechanics/generalized Born surface area continuum solvation method) and IC_50_ values for the acetylcholinesterase (AChE) inhibition assay (µM).

Compounds	IC_50_ (µM)	ΔG(AChE)	ΔG(BChE)
lindoldhamine isomer **1**	39.38 ± 0.08	−114.60	−110.15
stepharine **2**	61.24 ± 0.03	−68.87	−66.01
palmatine **3**	35.25 ± 0.04	−74.87	−82.64
5-*N*-methylmaytenine **4**	19.55 ± 0.09	−82.01	−68.49
*N-trans*-feruloyltyramine **5**	not active	−68.43	−67.20
neostigmine (positive control)	3.72 ± 0.03	−61.71	−32.18

## Data Availability

Data are available upon reasonable request.

## Supplementary Materials

The following are available online at https://www.mdpi.com/article/10.3390/molecules27103138/s1, Figures S1–S8: NMR, MS/MS and HRMS spectra of lindoldhamine isomer, Figures S9–S17: NMR, MS/MS and HRMS spectra of stepharine, Figures S18–S25: NMR, MS/MS and HRMS spectra of palmatine, Figures S26–S39: NMR, MS/MS and HRMS spectra of 5-*N*-methylmaytenine, Figures S40–S47: NMR, MS/MS and HRMS spectra of *N-trans*-feruloyltyramine. Table S1: NMR chemical shifts of lindoldhamine isomer Table S2: NMR chemical shifts of stepharine, Table S3: NMR chemical shifts of palmatine, Table S4: NMR chemical shifts of 5-N-methylmaitenine, Table S5: NMR chemical shifts of *N-trans*-feruloyltyramine, and MD simulation reports generated by Schrödinger software on AChE and BChE interactions with alkaloids.

## Sample Availability

Samples of the compounds **1–5** are available from the authors.
