# Supplementary material for: In Vitro and In Silico Evaluation of Cholinesterase Inhibition by Alkaloids Obtained from Branches of Abuta panurensis Eichler
Source: Molecules. 2022 May 13;27(10):3138. doi: 10.3390/molecules27103138 (PMC9144276; doi:10.3390/molecules27103138)
Supplement: Supplementary file 1 [file molecules-27-03138-s001.zip › molecules-1713251-supplementary.pdf]

Supplementary Material

In vitro and *in silico* Evaluation of Cholinesterase Inhibition by Alkaloids Obtained from Branches of *Abuta panurensis* Eichler

by

Rochelly da S. Mesquita, Andrii Kyrylchuk, Anton Cherednichenko, Ingrity S. C. Sá,
Lílian M. Bastos, Felipe M. A. da Silva, Rita de C. S. Nunomura, and Andriy Grafov*

Table of Contents

Item	Description	Pages
**Figure S1**	**^{1}H NMR spectrum (500 MHz, CD_3OD d_6, TMS) of lindoldhamine isomer.**	**5**
**Figure S2**	**Expanded low-field regions of the 1H NMR spectrum (500 MHz, CD_3OD d_6, TMS) of lindoldhamine isomer.**	**6**
**Figure S3**	**HSQC spectrum (125 MHz, CD_3OD, TMS) of lindoldhamine isomer.**	**7**
**Figure S4**	**Expanded regions of the HSQC spectrum (125 MHz, CD_3OD, TMS) of lindoldhamine isomer.**	**8**
**Figure S5**	**HMBC spectrum (125 MHz, CD_3OD, TMS) of lindoldhamine isomer.**	**9**
**Figure S6**	**Expanded regions of the HMBC spectrum (125 MHz, CD_3OD, TMS) of lindoldhamine isomer.**	**10**
**Table S1**	**NMR chemical shifts of lindoldhamine isomer.** The experiments were realized in CD_3OD at magnetic field strength of 11.7 T; 500 MHz for ^{1}H NMR.	**11**
**Figure S7**	**Mass spectrum (MS/MS) of lindoldhamine isomer with chemical structure and identification of the main fragment ions.**	**12**
**Figure S8**	**Hight resolution mass-spectrum of lindoldhamine isomer with chemical structure.**	**13**
**Figure S9**	**^{1}H NMR spectrum (500 MHz, CD_3OD, TMS) of stepharine.**	**14**
**Figure S10**	**Expanded regions of the 1H NMR spectrum (500 MHz, CD_3OD, TMS) of stepharine.**	**15**
**Figure S11**	**Expanded regions of the 1H NMR spectrum (500 MHz, CD_3OD, TMS) of stepharine.**	**16**
**Figure S12**	**HSQC spectrum (125 MHz, CD_3OD, TMS) of stepharine.**	**17**
**Figure S13**	**Expanded regions of the HSQC spectrum (125 MHz, CD_3OD, TMS) of stepharine.**	**18**
**Figure S14**	**HMBC spectrum (125 MHz, CD_3OD, TMS) of stepharine.**	**19**
**Figure S15**	**Expanded regions of the HMBC spectrum (125 MHz, CD_3OD, TMS) of stepharine.**	**20**
**Table S2**	**NMR chemical shifts of stepharine.** The experiments were realized in CD_3OD at magnetic field strength of 11.7 T; 500 MHz for ^{1}H NMR.	**21**
**Figure S16**	**Mass spectrum (MS/MS) of stepharine with chemical structure and identification of the main fragment ions.**	**22**
**Figure S17**	**Hight resolution mass spectrum of stepharine with chemical structure.**	**23**
**Figure S18**	**^{1}H NMR spectrum (500 MHz, CD_3OD d_6, TMS) of palmatine.**	**24**
**Figure S19**	**Expanded low-field region of the ^{1}H NMR spectrum (500 MHz, CD_3OD d_6, TMS) of palmatine.**	**25**
**Figure S20**	**HSQC spectrum (125 MHz, CD_3OD, TMS) of palmatine.**	**26**
**Figure S21**	**Expanded regions of the HSQC spectrum (125 MHz, CD_3OD, TMS) of palmatine.**	**27**
**Figure S22**	**HMBC spectrum (125 MHz, CD_3OD, TMS) of palmatine.**	**28**
**Figure S23**	**Expanded regions of the HMBC spectrum (125 MHz, CD_3OD, TMS) of palmatine.**	**29**
**Table S3**	**NMR chemical shifts of palmatine.** The experiments were realized in CD_3OD at magnetic field strength of 11.7 T; 500 MHz for ^{1}H NMR.	**30**

Table of Contents (continuation)

Item	Description	Pages
**Figure S24**	**Mass spectrum (MS/MS) of palmatine with chemical structure and identification of the main fragment ions.**	**31**
**Figure S25**	**Hight resolution mass-spectrum of palmatine with chemical structure.**	**32**
**Figure S26**	**^{1}H NMR spectrum (500 MHz, DMSO d_6, TMS) of 5-*N*-methylmaytenine.**	**33**
**Figure S27**	**Expanded low-field region of the ^{1}H NMR spectrum (500 MHz, DMSO d_6, TMS) of 5-*N*-methylmaytenine.**	**34**
**Figure S28**	**^{13}C NMR spectrum (125 MHz, DMSO d_6, TMS) of 5-*N*-methylmaytenine.**	**35**
**Figure S29**	**Expanded regions of the^{13}C NMR spectrum (125 MHz, DMSO d_6, TMS) of 5-*N*-methylmaytenine.**	**36**
**Figure S30**	**Expanded regions of the ^{13}C NMR spectrum (125 MHz, DMSO d_6, TMS) of 5-*N*-methylmaytenine.**	**37**
**Figure S31**	**Expanded region of the ^{13}C NMR spectrum (125 MHz, DMSO d_6, TMS) of 5-*N*-methylmaytenine.**	**38**
**Figure S32**	**HMBC spectrum (125 MHz, DMSO d_6, TMS) of 5-N-methylmaytenine.**	**39**
**Figure S33**	**Expanded regions of the HMBC spectrum (125 MHz, DMSO d_6, TMS) of 5-*N*-methylmaytenine.**	**40**
**Figure S34**	**HSQC spectrum (125 MHz, DMSO d_6, TMS) of 5-N-methylmaytenine.**	**41**
**Figure S35**	**Expanded regions of the HSQC spectrum (125 MHz, DMSO d_6, TMS) of 5-*N*-methylmaytenine.**	**42**
**Figure S36**	**DEPT-135º spectrum (125 MHz, DMSO d_6, TMS) of 5-N-methylmaytenine.**	**43**
**Figure S37**	**Expanded regions of the DEPT-135º spectrum (125 MHz, DMSO d_6, TMS) of 5-*N*-methylmaytenine.**	**44**
**Table S4**	**NMR chemical shifts of 5-*N*-methylmaitenine.** The experiments were realized in DMSO d_6 at magnetic field strength of 11.7 T; 500 MHz for ^{1}H and 125 MHz for ^{13}C NMR.	**45**
**Figure S38**	**Mass spectrum (MS/MS) of 5-*N*-methylmaytenine with chemical structure and identification of the main fragment ions.**	**46**
**Figure S39**	**Hight resolution mass-spectrum of 5-*N*-methylmaytenine with chemical structure.**	**47**
**Figure S40**	**^{1}H NMR spectrum (500 MHz, CD3OD d_6, TMS) of *N*-*trans*-feruloyltyramine.**	**48**
**Figure S41**	**Expanded low-field region of the ^{1}H NMR spectrum (500 MHz, CD_3OD d_6, TMS) *N*-*trans*-feruloyltyramine.**	**49**
**Figure S42**	**HSQC spectrum (125 MHz, CD_3OD, TMS) *N*-*trans*-feruloyltyramine.**	**50**
**Figure S43**	**Expanded regions of the HSQC spectrum (125 MHz, CD_3OD, TMS) *N*-*trans*-feruloyltyramine.**	**51**
**Figure S44**	**HMBC spectrum (125 MHz, CD_3OD, TMS) of *N*-*trans*-feruloyltyramine.**	**52**
**Figure S45**	**Expanded regions of the HMBC spectrum (125 MHz, CD_3OD, TMS) of *N*-*trans*-feruloyltyramine.**	**53**
**Table S5**	**NMR chemical shifts of *N*-*trans*-feruloyltyramine**. The experiments were realized in CD_3OD at magnetic field strength of 11.7 T; 500 MHz for ^{1}H NMR.	**54**

Table of Contents (continuation)

Item	Description	Pages
**Figure S46**	**Mass spectrum (MS/MS) of *N-trans*-feruloyltyramine with chemical structure and identification of the main fragment ions.**	**55**
**Figure S47**	**Hight resolution mass-spectrum of *N-trans*-feruloyltyramine with chemical structure.**	**56**
**Report S1**	**MD Simulation Report on AChE - Neostigmine Interactions.**	**57-67**
**Report S2**	**MD Simulation Report on AChE - Lindoldhamine Isomer Interactions.**	**68-79**
**Report S3**	**MD Simulation Report on AChE - Stepharine Interactions.**	**80-90**
**Report S4**	**MD Simulation Report on AChE - Palmatine Interactions.**	**91-101**
**Report S5**	**MD Simulation Report on AChE - 5-*N*-Methylmaytenine Interactions.**	**102-112**
**Report S6**	**MD Simulation Report on AChE - *N-trans*-Feruloyltyramine Interactions.**	**113-122**
**Report S7**	**MD Simulation Report on BChE - Neostigmine Interactions.**	**123-132**
**Report S8**	**MD Simulation Report on BChE - Lindoldhamine Isomer Interactions.**	**133-144**
**Report S9**	**MD Simulation Report on BChE - Stepharine Interactions.**	**145-156**
**Report S10**	**MD Simulation Report on BChE - Palmatine Interactions.**	**157-167**
**Report S11**	**MD Simulation Report on BChE - 5-*N*-Methylmaytenine Interactions.**	**168-178**
**Report S12**	**MD Simulation Report on BChE - *N-trans*-Feruloyltyramine Interactions.**	**179-188**

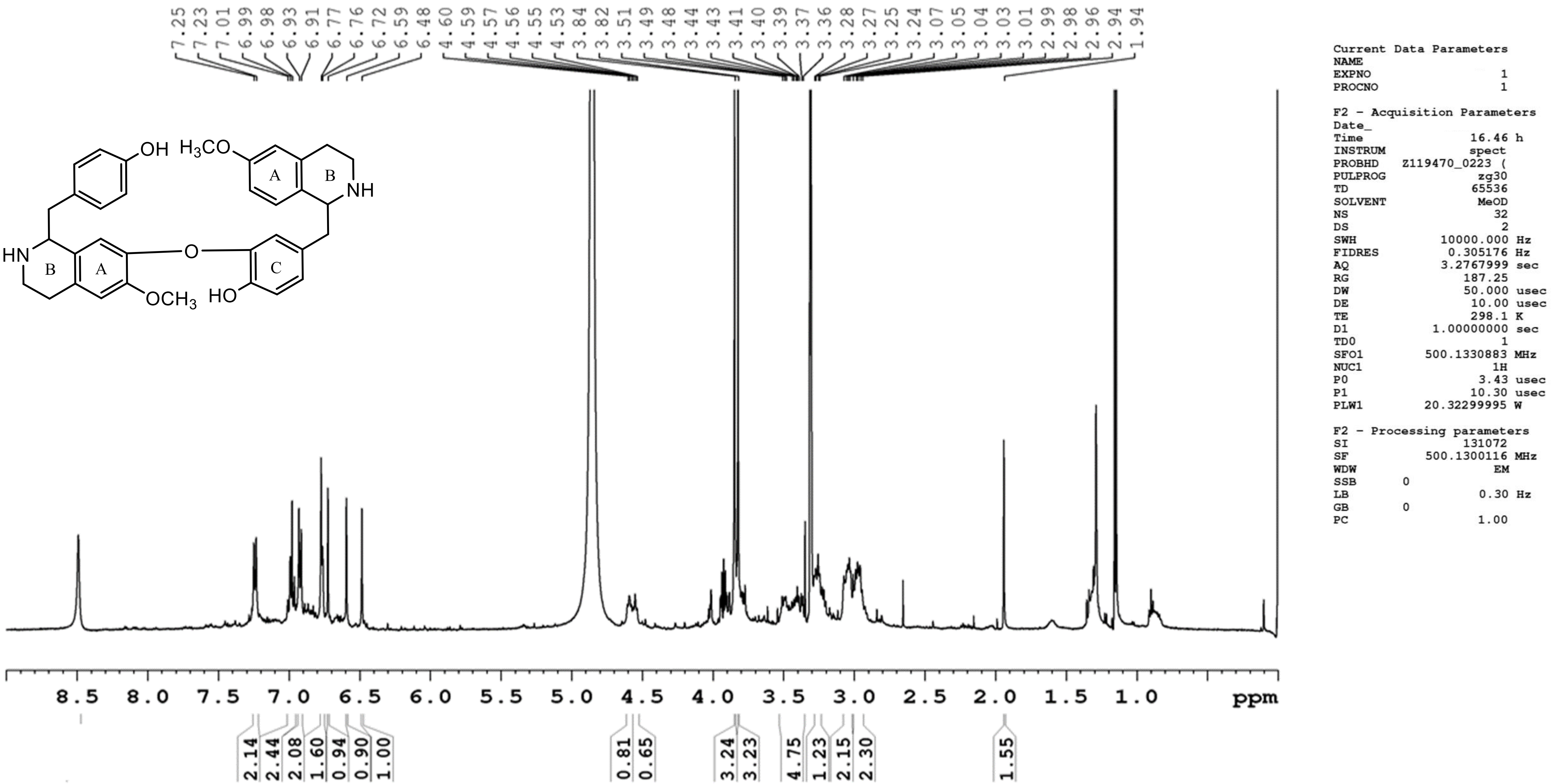

Figure S1: ^{1}H NMR spectrum (500 MHz, CD_3OD d_6, TMS) of lindoldhamine isomer.

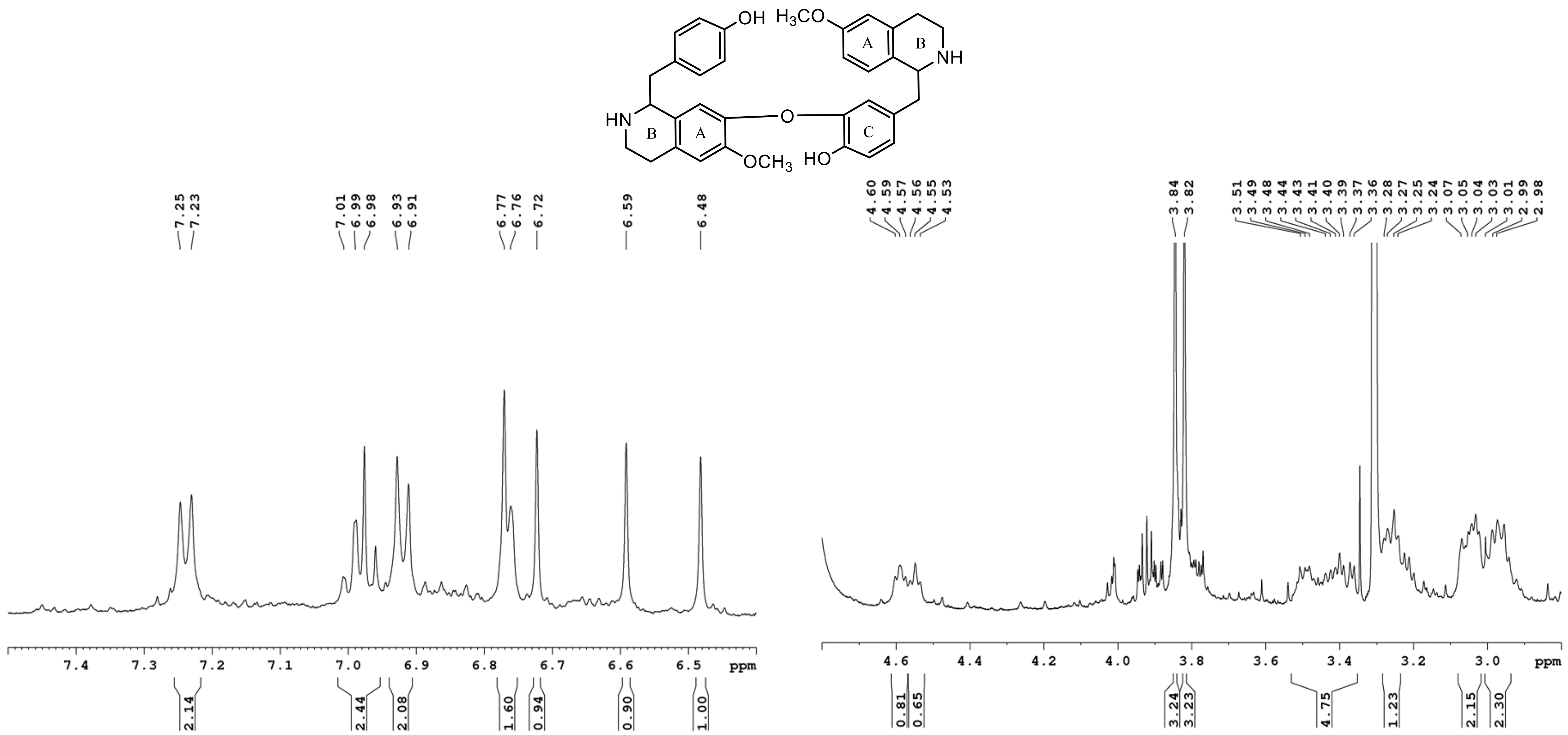

Figure S2: Expanded low-field regions of the ^{1}H NMR spectrum (500 MHz, CD_3OD d_6, TMS) of lindoldhamine isomer.

```
Current Data Parameters
NAME
EXPNO                    200
PROCNO                     1

F2 - Acquisition Parameters
Date_
Time                   21.13 h
INSTRUM                spect
PROBHD   Z119470_0223 (
PULPROG  hsqcetgpsisp2.2
TD                      2048
SOLVENT                 MeOD
NS                        64
DS                        16
SWH                 5804.954 Hz
FIDRES              2.834450 Hz
AQ                 0.1764011 sec
RG                    187.25
DW                    86.133 usec
DE                     10.00 usec
TE                     298.2 K
CNST2            145.0000000
CNST17            -0.5000000
D0                0.00000300 sec
D1                1.00000000 sec
D4                0.00172414 sec
D11               0.03000000 sec
D16               0.00020000 sec
D24               0.00089000 sec
IN0               0.00001850 sec
TDav                       1
SFO1             500.1322610 MHz
NUC1                      1H
P1                      9.40 usec
P2                     18.80 usec
P28                  1000.00 usec
PLW1             20.32299995 W
SFO2             125.7703643 MHz
NUC2                     13C
CPDPRG[2                garp
P3                     10.00 usec
P14                   500.00 usec
P24                  2000.00 usec
PCPD2                  70.00 usec
PLW0       0 W
PLW2             88.00000000 W
PLW12             1.78668594 W
SPNAM[3] Crp60,0.5,20.1
SPOAL3                 0.500
SPOFFS3  0 Hz
SPW3             13.44499969 W
SPNAM[7]      Crp60comp.4
SPOAL7                 0.500
SPOFFS7  0 Hz
SPW7             13.44499969 W
GPNAM[1]        SMSQ10.100
GPZ1                   80.00 %
GPNAM[2]        SMSQ10.100
GPZ2                   20.10 %
GPNAM[3]        SMSQ10.100
GPZ3                   11.00 %
GPNAM[4]        SMSQ10.100
GPZ4                   -5.00 %
P16                  1000.00 usec
P19                   600.00 usec

F1 - Acquisition parameters
TD                       256
SFO1                125.7704 MHz
FIDRES            211.148651 Hz
SW                   214.892 ppm
FnMODE      Echo-Antiecho

F2 - Processing parameters
SI                      4096
SF               500.1300109 MHz
WDW                    QSINE
SSB                        2
LB         0 Hz
GB         0
PC                      1.40

F1 - Processing parameters
SI                      1024
MC2           echo-antiecho
SF               125.7577790 MHz
WDW                    QSINE
SSB                        2
LB         0 Hz
GB         0
```

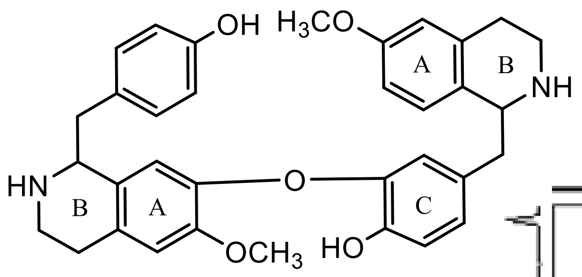

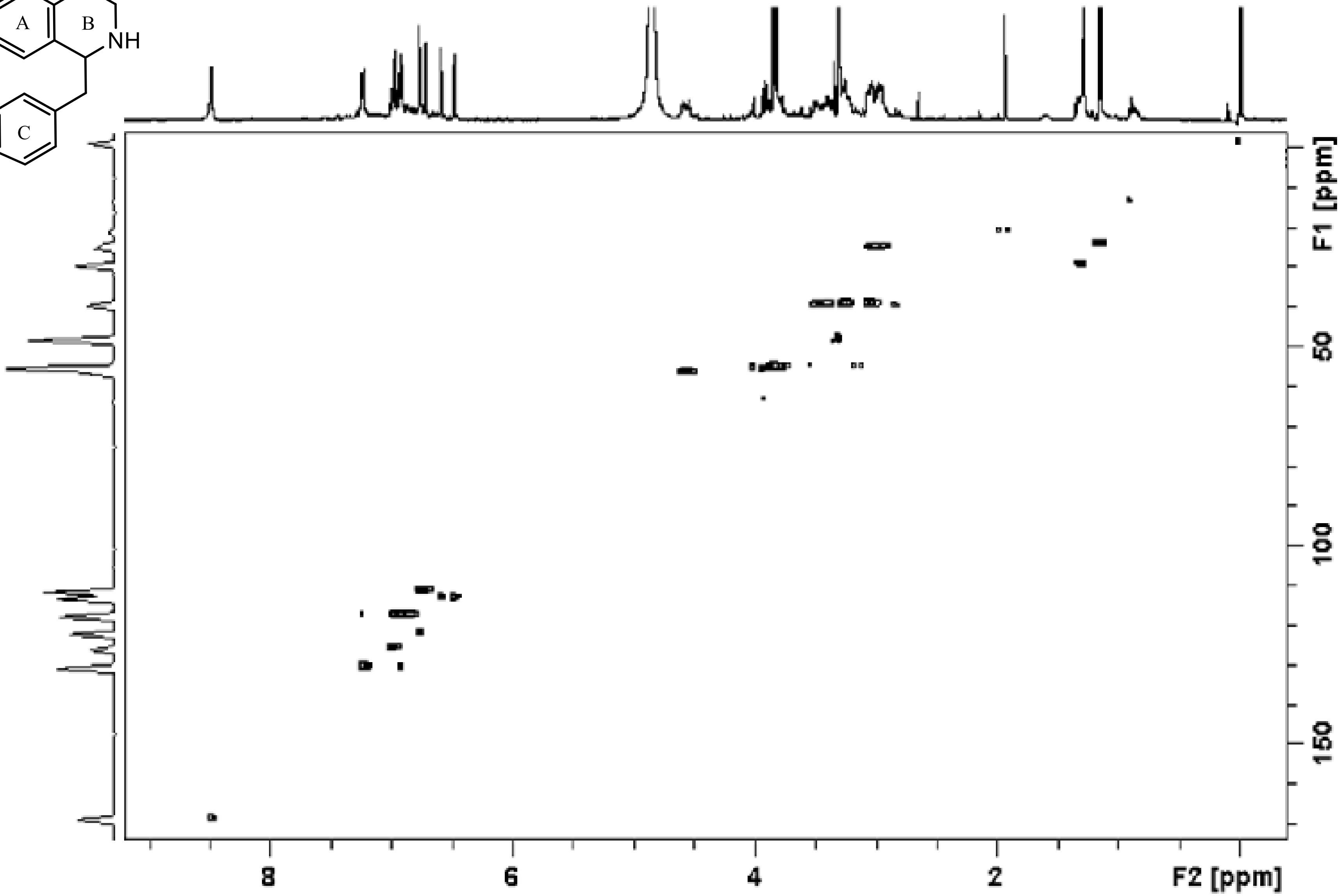

Figure S3: HSQC spectrum (125 MHz, CD_3OD, TMS) of lindoldhamine isomer.

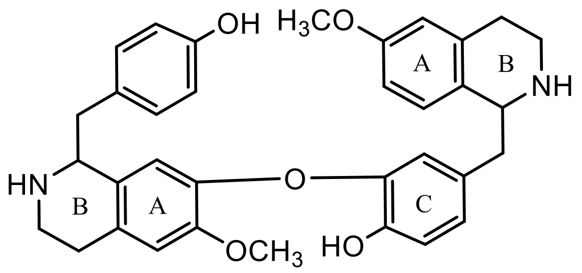

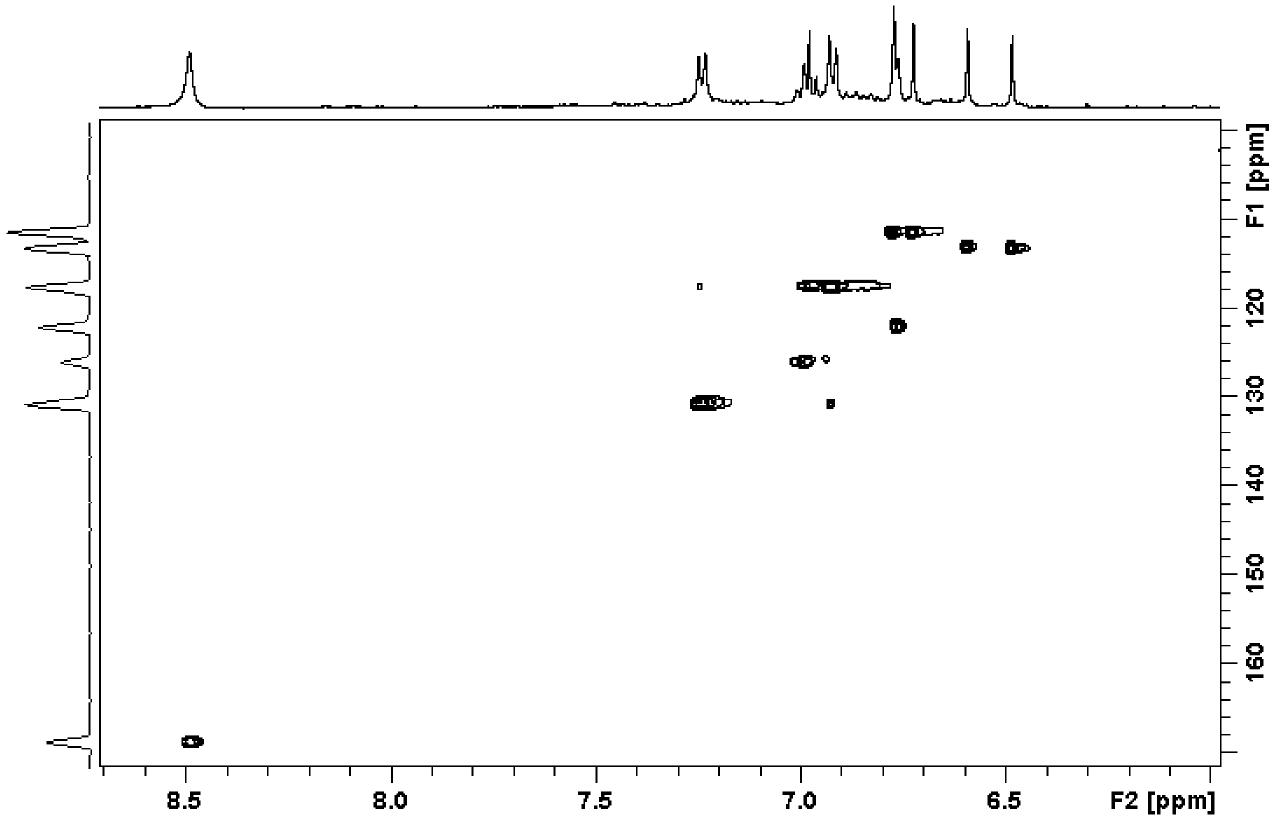

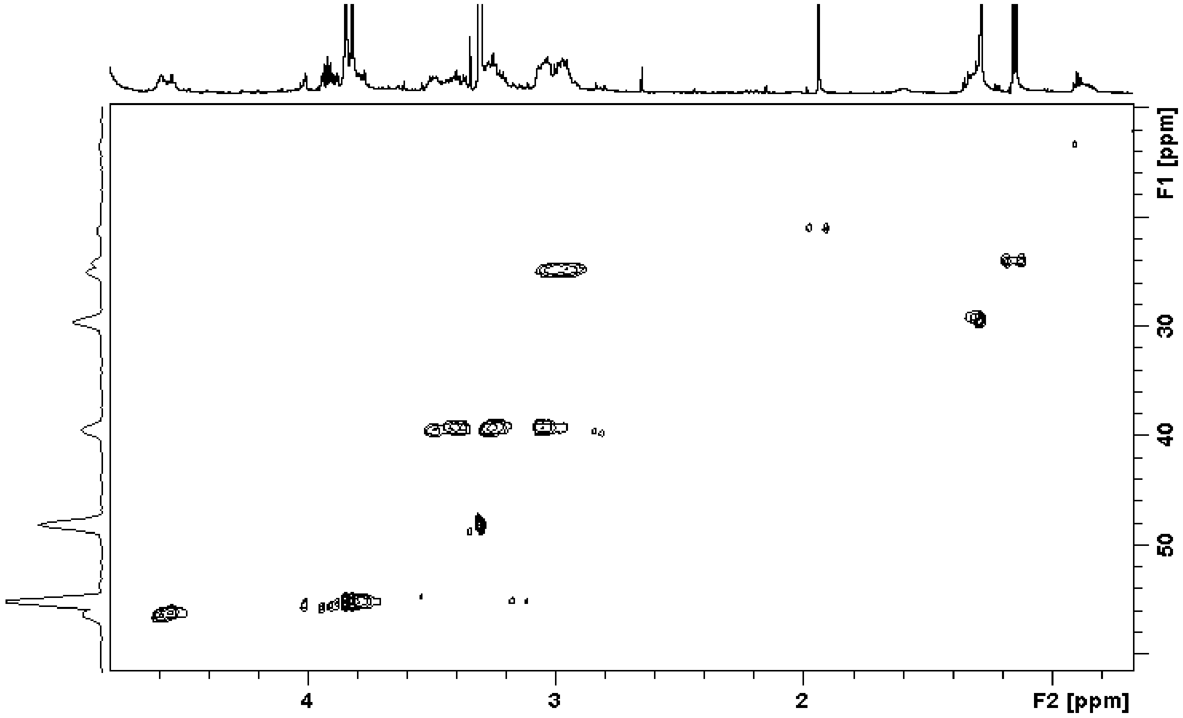

Figure S4: Expanded regions of the HSQC spectrum (125 MHz, CD_3OD, TMS) of lindoldhamine isomer.

**Current Data Parameters**		
NAME		
EXPNO	300	
PROCNO	1	
**F2 - Acquisition Parameters**		
Date_		
Time	2.44	h
INSTRUM	spect	
PROBHD	Z119470_0223 (	
PULPROG	hmbcgplpndqf	
TD	2048	
SOLVENT	MeOD	
NS	184	
DS	16	
SWH	5804.954	Hz
FIDRES	2.834450	Hz
AQ	0.1764011	sec
RG	187.25	
DW	86.133	usec
DE	10.00	usec
TE	298.1	K
CNST2	145.0000000	
CNST13	8.0000000	
D0	0.00000300	sec
D1	1.00000000	sec
D2	0.00344828	sec
D6	0.06250000	sec
D16	0.00020000	sec
IN0	0.00001660	sec
TDav	1	
SFO1	500.1322610	MHz
NUC1	1H	
P1	9.40	usec
P2	18.80	usec
PLW1	20.32299995	W
SFO2	125.7716219	MHz
NUC2	13C	
P3	10.00	usec
PLW2	88.00000000	W
GPNAM[1]	SMSQ10.100	
GPZ1	50.00	%
GPNAM[2]	SMSQ10.100	
GPZ2	30.00	%
GPNAM[3]	SMSQ10.100	
GPZ3	40.10	%
P16	1000.00	usec
**F1 - Acquisition parameters**		
TD	294	
SFO1	125.7716	MHz
FIDRES	204.901245	Hz
SW	239.486	ppm
FnMODE	QF	
**F2 - Processing parameters**		
SI	4096	
SF	500.1300121	MHz
WDW	SINE	
SSB	0	
LB	0	Hz
GB	0	
PC	1.40	
**F1 - Processing parameters**		
SI	1024	

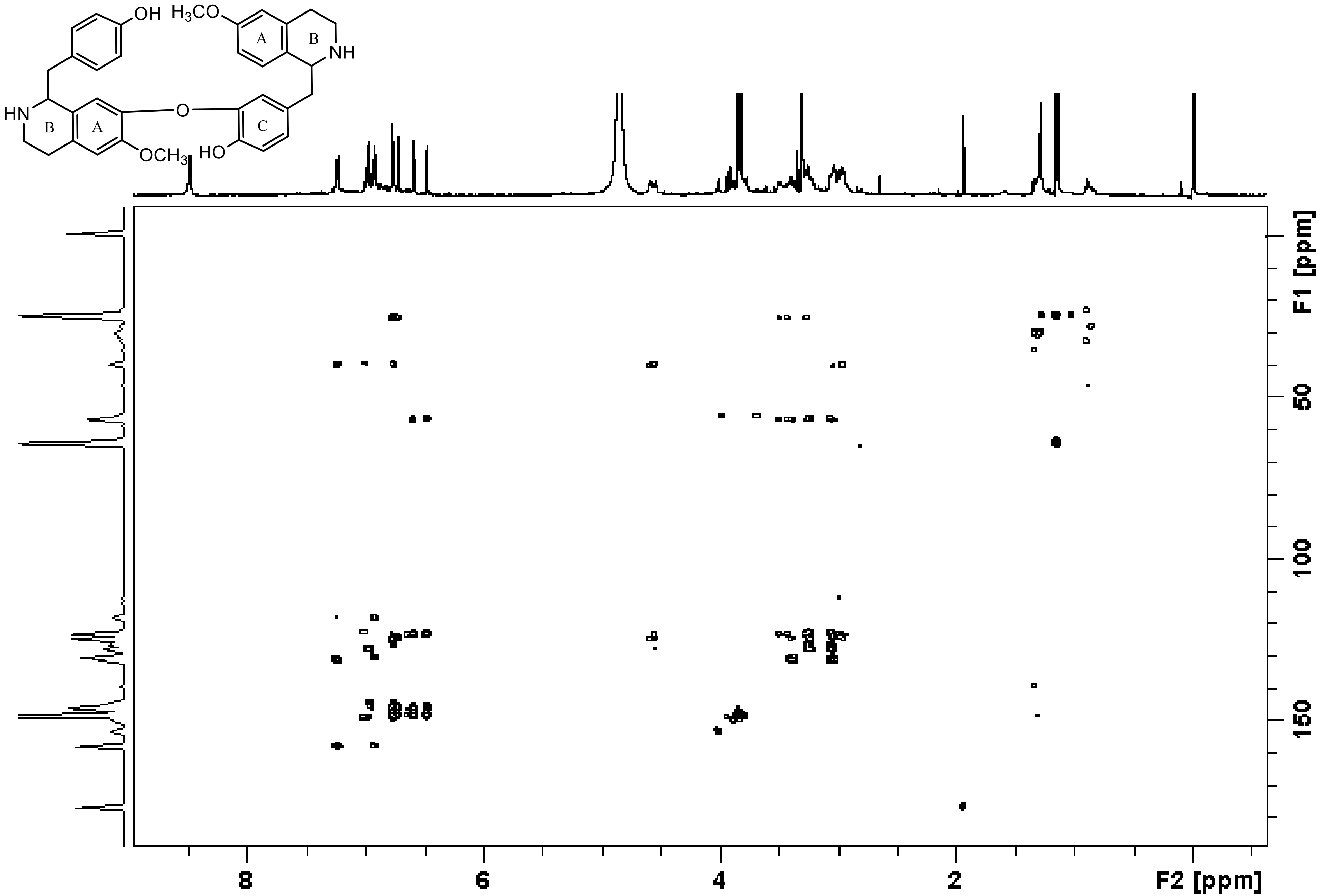

Figure S5: HMBC spectrum (125 MHz, CD_3OD, TMS) of lindoldhamine isomer.

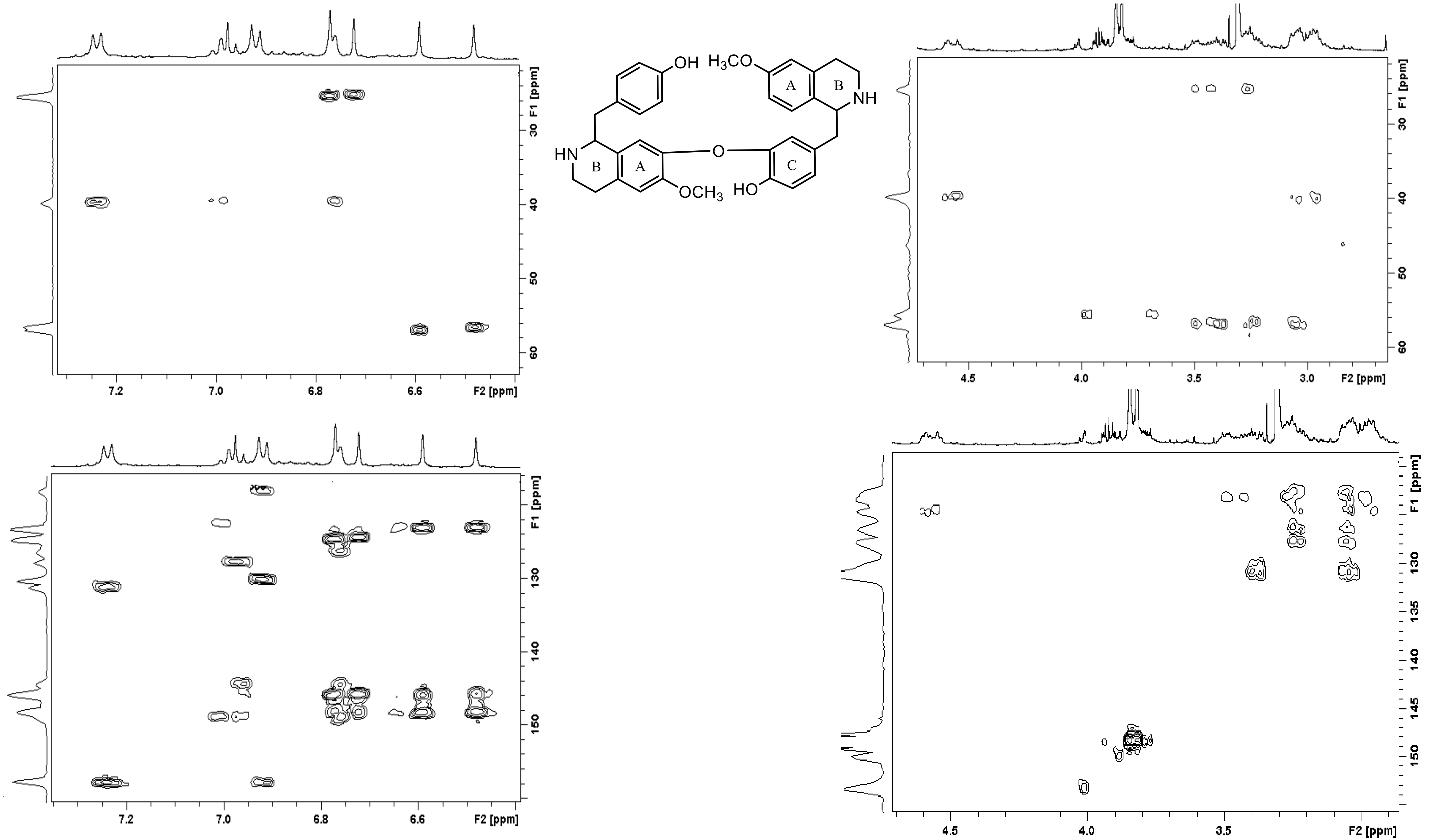

Figure S6: Expanded regions of the HMBC spectrum (125 MHz, CD_3OD, TMS) of lindoldhamine isomer.

Table S1: NMR chemical shifts of lindoldhamine isomer. The experiments were realized in CD_3OD at magnetic field strength of 11.7 T; 500 MHz for ^1H NMR.

s: singlet; m: multiplet; dd: doublet of doublets; ddd: doublet of doublets of doublets.

Position	^1H δ_H, ppm (multiplicity; number of H; J, Hz)	^{13}C δ_C (HSQC), ppm [a]	HMBC, ppm [a]
1	4.59 (t;1H; 7.1 Hz)	56.1	C-15; C-4a
3	3.40 (m; 2H)	39.3	C-4; C-1; C-8a
4	3.03 and 3.07 (m; 2H)	24.9	C-3; C-1
4a	-	124.5	-
5	6.77 (s; 1H)	111.4	C-4;C-8; C-8a; C-4a; C-7; C-6
6	-	148.2	-
6-OCH_3	3.82 (s; 3H)	55.2	C-7; C-6
7	-	145.6	-
8	6.48 (s; 1H)	113.1	C-6 OCH_3; C-8a; C-7; C-6
8a	-	123.1	-
9	-	131.1	-
10	7.25 (d;1H; 8.5 Hz)	130.6	C-15; C-11; C-9; C-12; C-14
11	6.93 (d; 1H; 8.5 Hz)	117.5	C-13; C-9; C-12
12	-	157.8	-
13	6.93 (d;1H; 8.5 Hz)	117.5	C-11; C-9; C-12
14	7.25 (d;1H; 8.5 Hz)	126.0	C-15; C-13; C-10; C-9; C-12
15	3.05 (m; 1H)<br>3.36 (m; 1H)	39.3	C-1; C-8a; C-14; C-10; C-9
1’	4.55 (t; 1H; 7.1 Hz)	56.4	C-15’; C-4a’
3’	3.49 (m; 2H)	39.4	C-4’; C-1’; C-8a’
4’	2.98 and 2.99 (m; 2H)	24.8	C-3’; C-1’; C-8a’; C-4a’
4a’	-	124.3	-
5’	6.72 (s; 1H)	111.3	C-4’; C-8’; C-4a’; C-7’; C-6’
6’	-	148.2	-
6’-OCH_3	3.84 (s; 3H)	55.1	C-7’; C-6’
7’	-	145.7	-
8’	6.59 (s; 1H)	112.9	C-8a’; C-1’; C-7’; C-6’
8a’	-	123.0	-
9’	-	127.7	-
10’	6.76 (s; 1H)	121.9	C-15’; C-14’; C-12’
11’	-	144.5	-
12’	-	148.8	-
13’	6.98 (d;1H; 8.5 Hz)	117.6	C-15’; C-9’; C-11’; C-12’
14’	6.99 (dd;1H; 8.5 and 2.0 Hz)	126.0	C-15’; C-10’; C-12’
15’	3.05 (m; 1H)<br>3.25 (m; 1H)	39.3	C-1’; C-10’; C-8a’; C-14’; C-9’

[a] ^{13}C NMR signals were assigned using the HSQC and HMBC data.

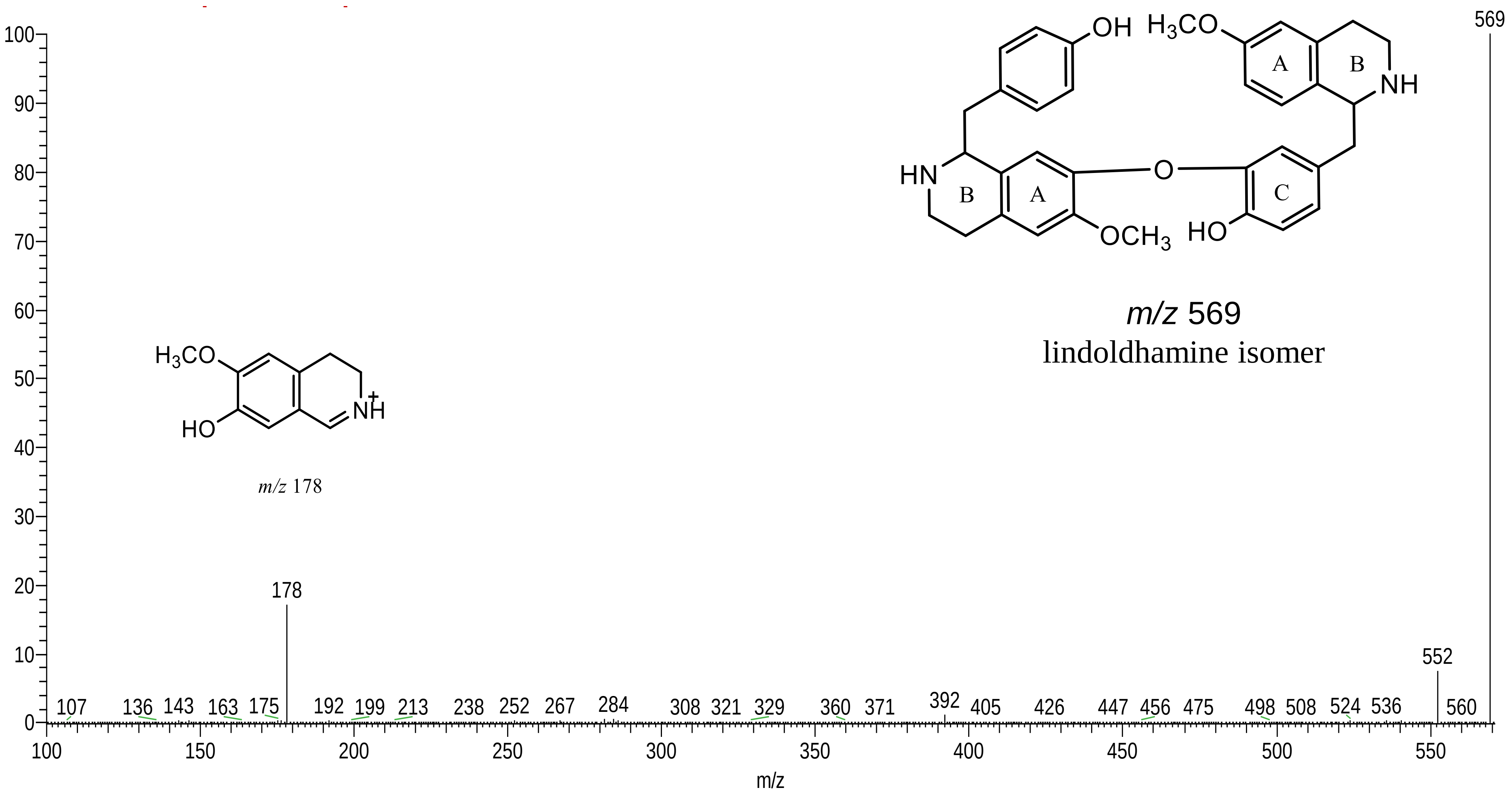

Figure S7: Mass spectrum (MS/MS) of lindoldhamine isomer with chemical structure and identification of the main fragment ions.

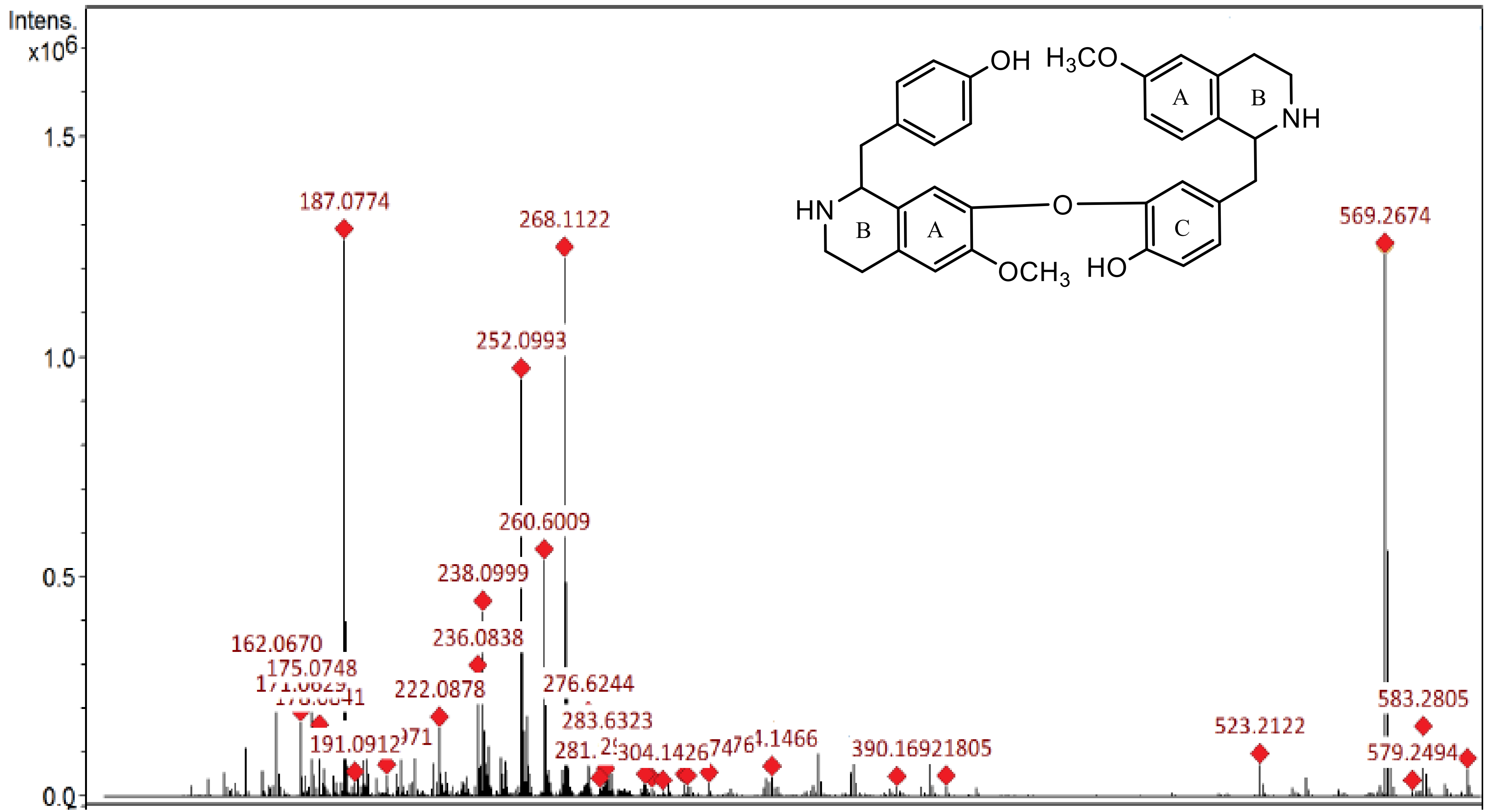

Figure S8: Hight resolution mass-spectrum of lindoldhamine isomer with chemical structure.

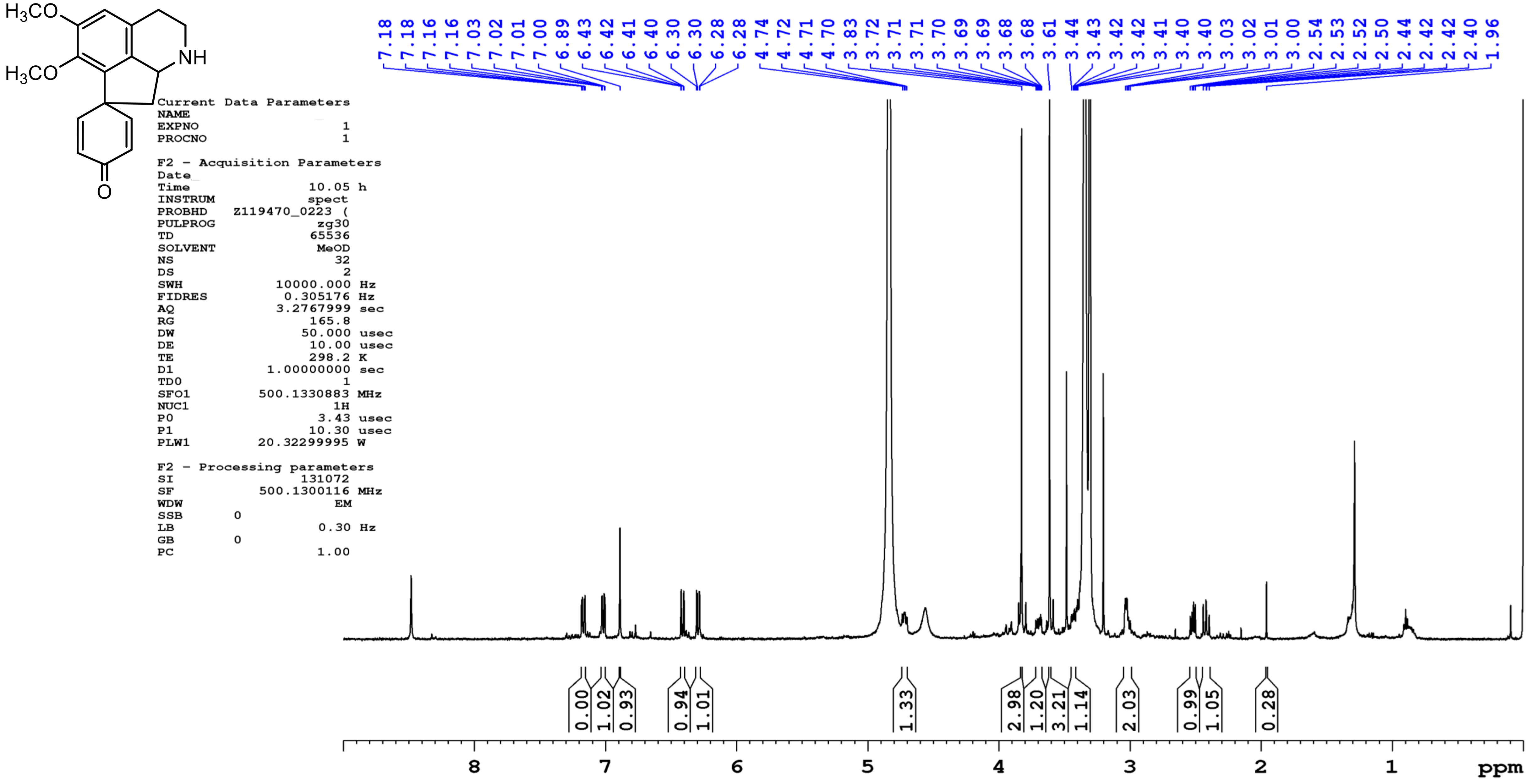

Figure S9: ^1H NMR spectrum (500 MHz, CD_3OD, TMS) of stepharine.

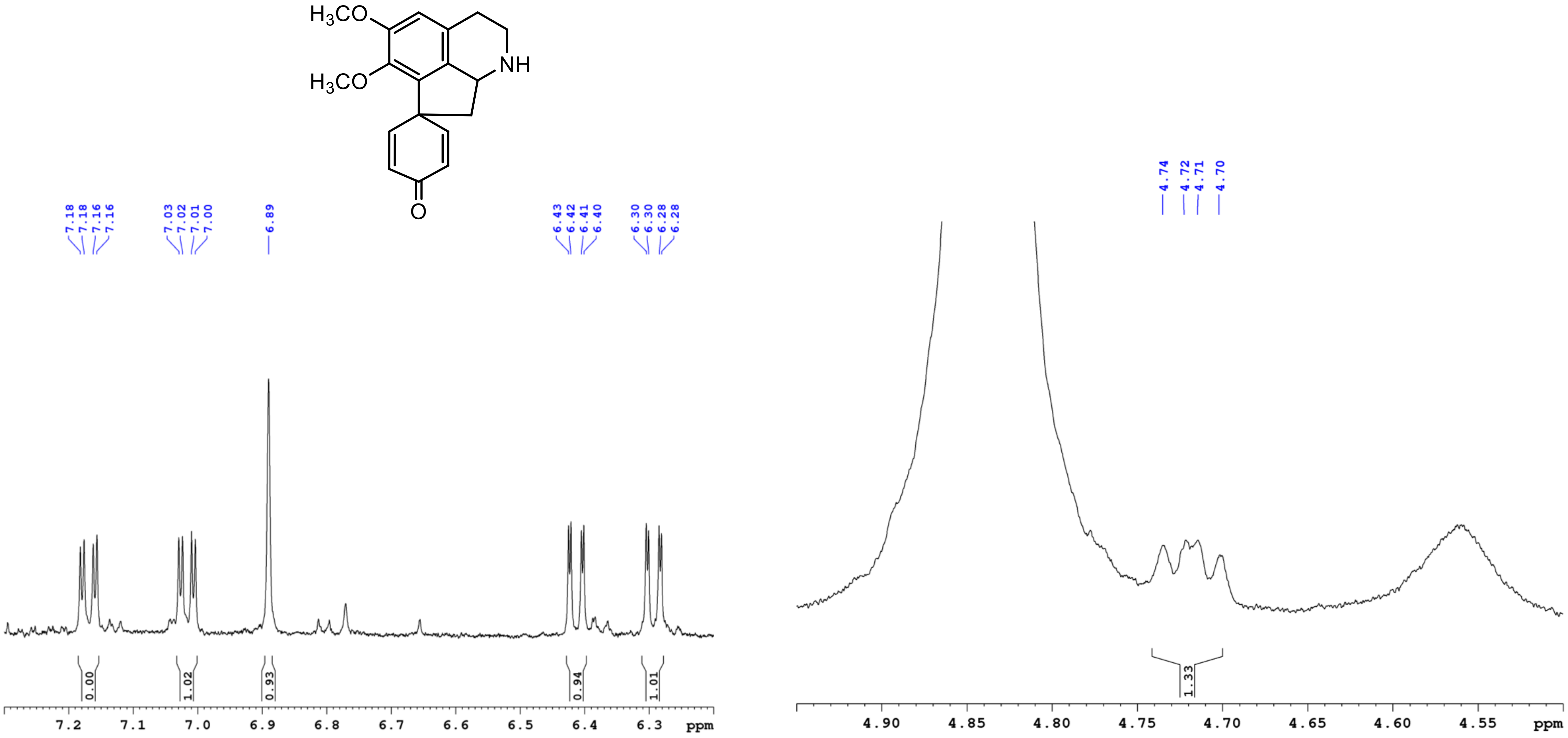

Figure S10: Expanded regions of the ^{1}H NMR spectrum (500 MHz, CD_3OD, TMS) of stepharine.

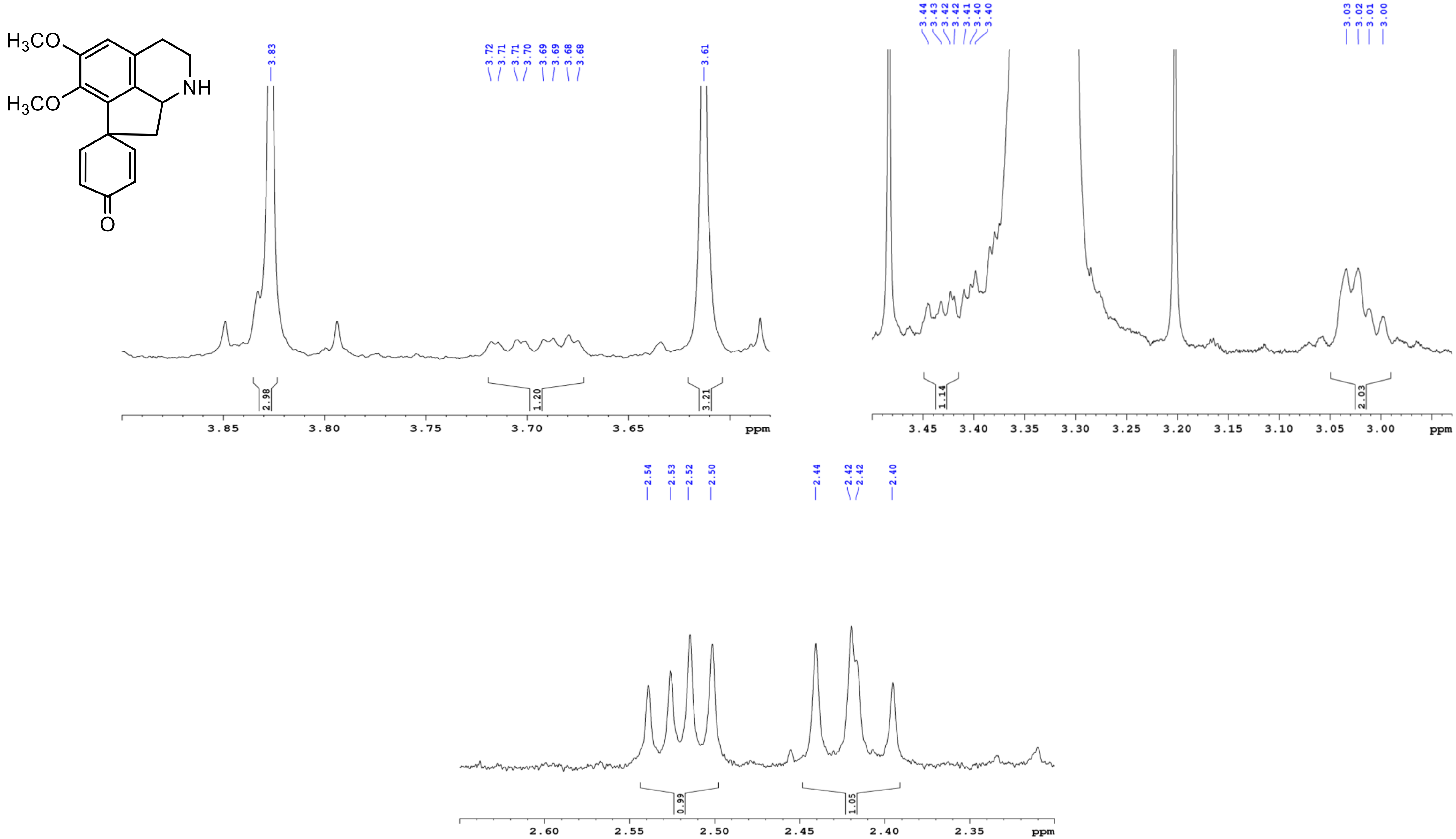

Figure S11: Expanded regions of the ^{1}H NMR spectrum (500 MHz, CD_3OD, TMS) of stepharine.

Current Data Parameters
NAME
EXPNO 200
PROCNO 1

F2 - Acquisition Parameters
Date_
Time 18.13 h
INSTRUM spect
PROBHD Z119470_0223 (
PULPROG hsqcetgpsisp2.2
TD 2048
SOLVENT MeOD
NS 64
DS 16
SWH 6421.233 Hz
FIDRES 3.135368 Hz
AQ 0.1594709 sec
RG 187.25
DW 77.867 usec
DE 10.00 usec
TE 298.2 K
CNST2 145.0000000
CNST17 -0.5000000
D0 0.00000300 sec
D1 1.00000000 sec
D4 0.00172414 sec
D11 0.03000000 sec
D16 0.00020000 sec
D24 0.00089000 sec
IN0 0.00001850 sec
TDav 1
SFO1 500.1322062 MHz
NUC1 1H
P1 9.40 usec
P2 18.80 usec
P28 1000.00 usec
PLW1 20.32299995 W
SFO2 125.7703643 MHz
NUC2 13C
CPDPRG[2 garp
P3 10.00 usec
P14 500.00 usec
P24 2000.00 usec
PCPD2 70.00 usec
PLW0 0 W
PLW2 88.00000000 W
PLW12 1.78668594 W
SPNAM[3] Crp60,0.5,20.1
SPOAL3 0.500
SPOFFS3 0 Hz
SPW3 13.44499969 W
SPNAM[7] Crp60comp.4
SPOAL7 0.500
SPOFFS7 0 Hz
SPW7 13.44499969 W
GPNAM[1] SMSQ10.100
GPZ1 80.00 %
GPNAM[2] SMSQ10.100
GPZ2 20.10 %
GPNAM[3] SMSQ10.100
GPZ3 11.00 %
GPNAM[4] SMSQ10.100
GPZ4 -5.00 %
P16 1000.00 usec
P19 600.00 usec

F1 - Acquisition parameters
TD 256
SFO1 125.7704 MHz
FIDRES 211.148651 Hz
SW 214.892 ppm
FnMODE Echo-Antiecho

F2 - Processing parameters
SI 4096
SF 500.1300093 MHz
WDW QSINE
SSB 2
LB 0 Hz
GB 0
PC 1.40

F1 - Processing parameters
SI 1024
MC2 echo-antiecho
SF 125.7577936 MHz
WDW QSINE
SSB 2
LB 0 Hz
GB 0

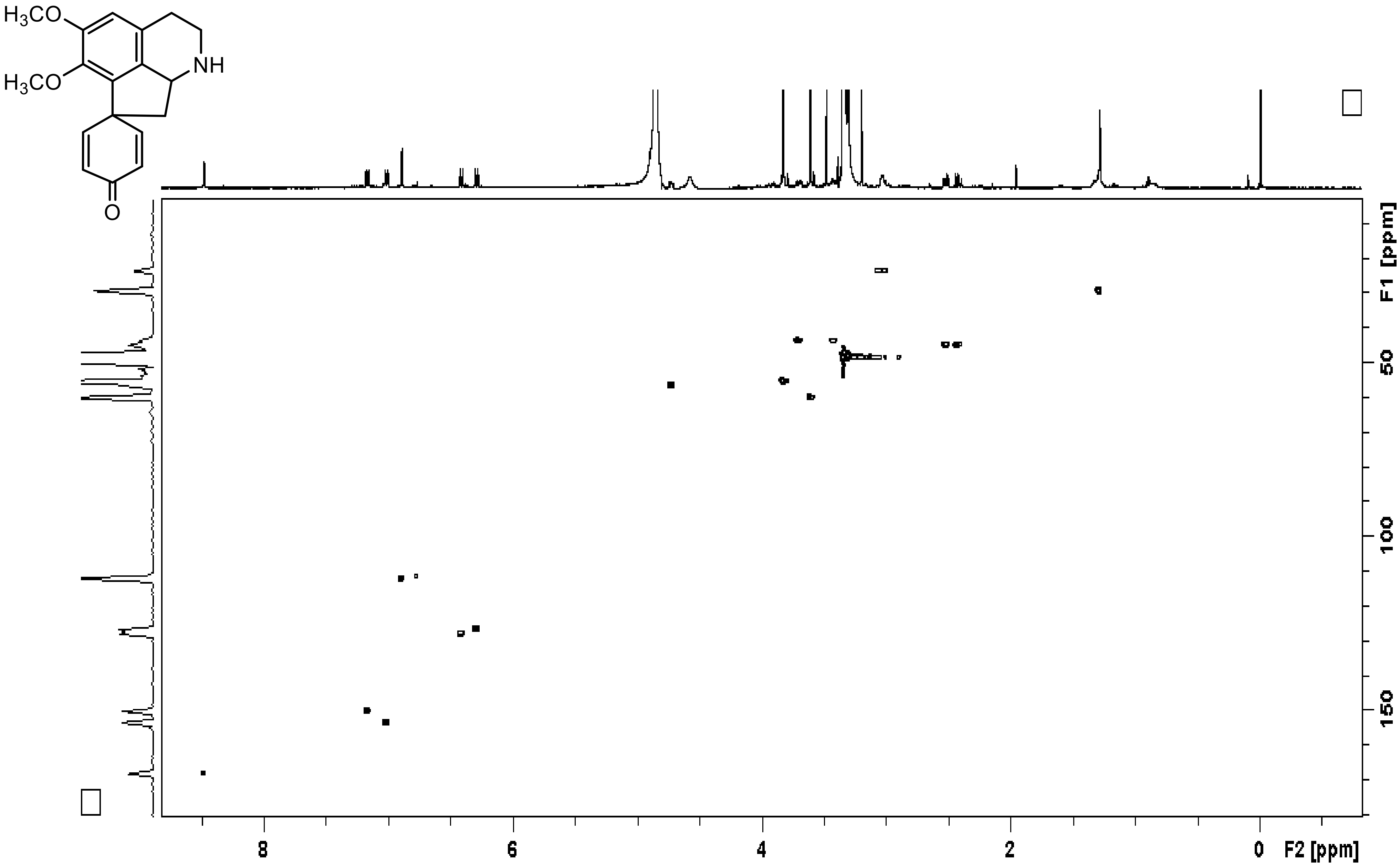

Figure S12: HSQC spectrum (125 MHz, CD_3OD, TMS) of stepharine.

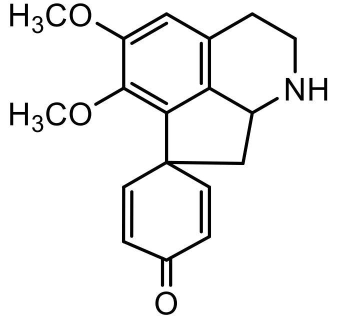

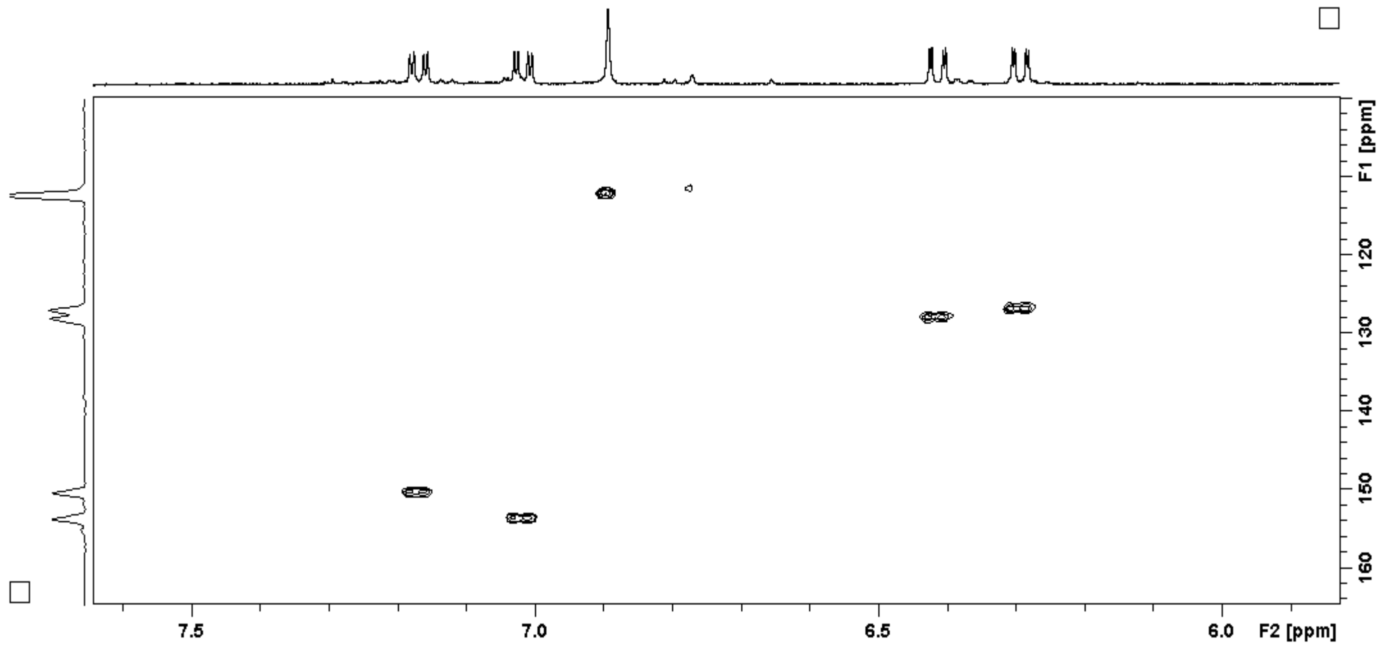

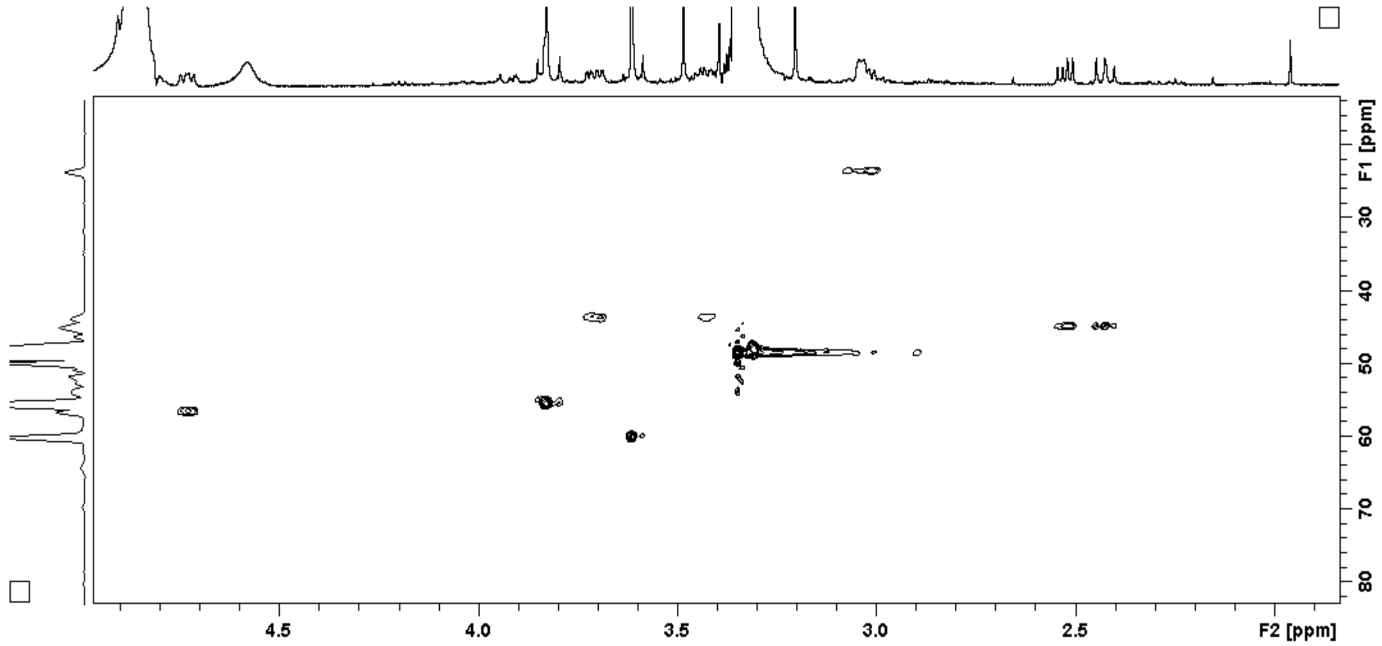

Figure S13: Expanded regions of the HSQC spectrum (125 MHz, CD_3OD, TMS) of stepharine.

```
Current Data Parameters
NAME
EXPNO                 300
PROCNO                  1

F2 - Acquisition Parameters
Date
Time                23.39 h
INSTRUM             spect
PROBHD    Z119470_0223 (
PULPROG     hmbcgplpndqf
TD                   2048
SOLVENT              MeOD
NS                    186
DS                     16
SWH              6421.233 Hz
FIDRES           3.135368 Hz
AQ              0.1594709 sec
RG                 187.25
DW                 77.867 usec
DE                  10.00 usec
TE                  298.2 K
CNST2         145.0000000
CNST13          8.0000000
D0             0.00000300 sec
D1             1.00000000 sec
D2             0.00344828 sec
D6             0.06250000 sec
D16            0.00020000 sec
IN0            0.00001660 sec
TDav                    1
SFO1          500.1322062 MHz
NUC1                   1H
P1                   9.40 usec
P2                  18.80 usec
PLW1          20.32299995 W
SFO2          125.7716219 MHz
NUC2                  13C
P3                  10.00 usec
PLW2          88.00000000 W
GPNAM[1]       SMSQ10.100
GPZ1                50.00 %
GPNAM[2]       SMSQ10.100
GPZ2                30.00 %
GPNAM[3]       SMSQ10.100
GPZ3                40.10 %
P16               1000.00 usec

F1 - Acquisition parameters
TD                    240
SFO1             125.7716 MHz
FIDRES         251.004013 Hz
SW                239.486 ppm
FnMODE                 QF

F2 - Processing parameters
SI                   4096
SF            500.1300128 MHz
WDW                  SINE
SSB      0
LB       0 Hz
GB       0
PC                   1.40

F1 - Processing parameters
SI                   1024
MC2                    QF
SF            125.7578149 MHz
WDW                  SINE
SSB      0
LB       0 Hz
GB       0
```

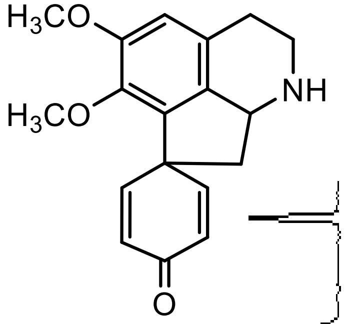

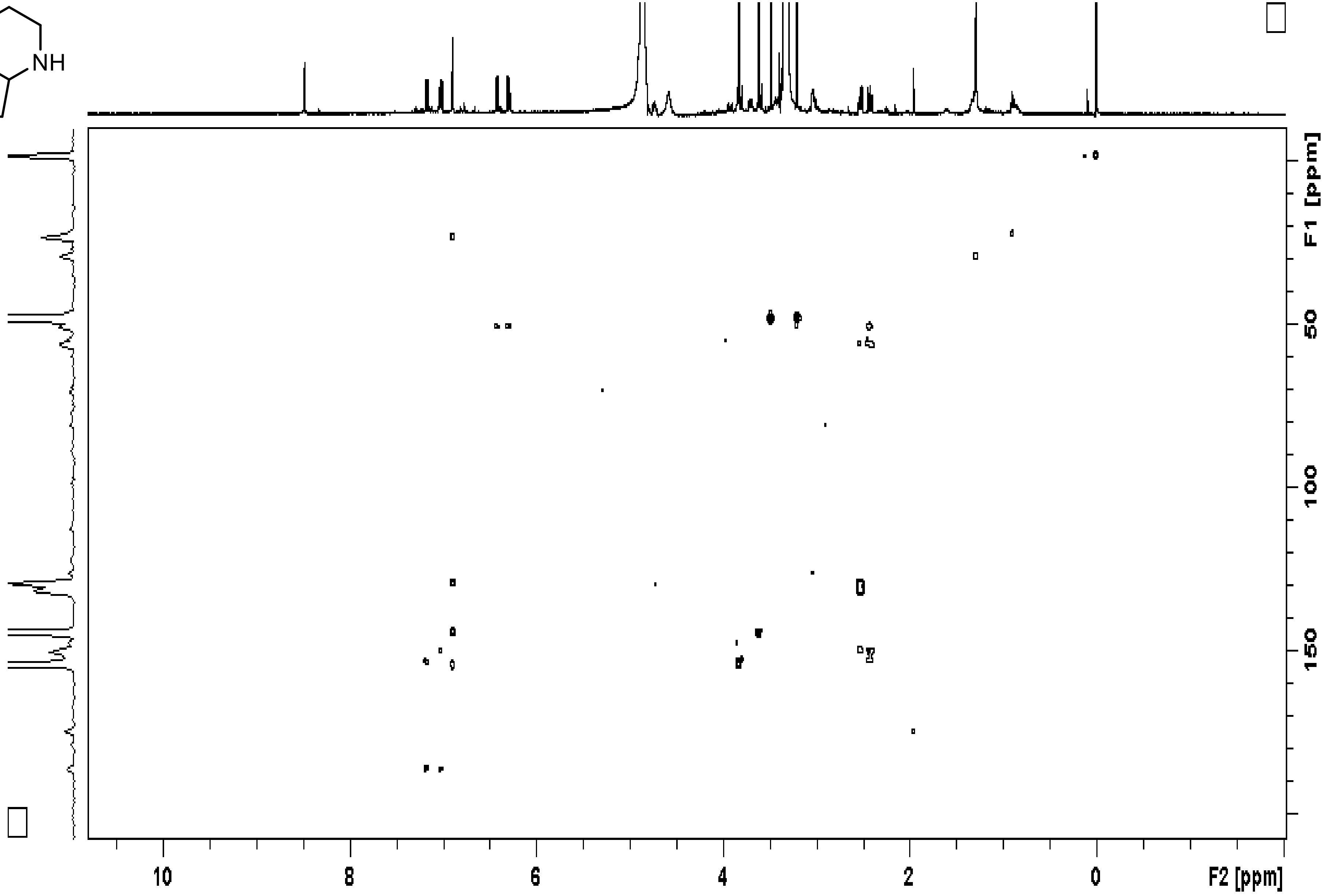

Figure S14: HMBC spectrum (125 MHz, CD_3OD, TMS) of stepharine.

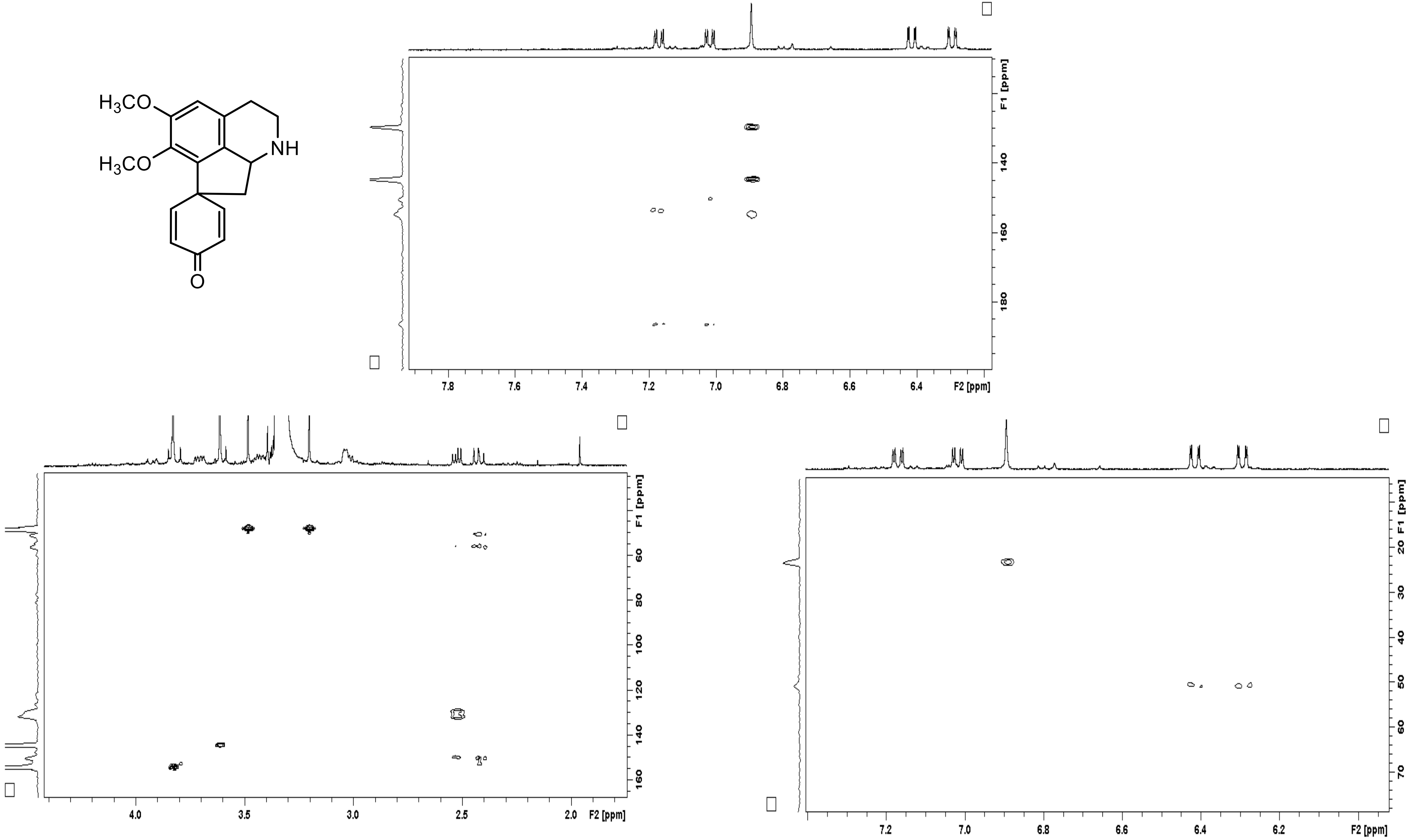

Figure S15: Expanded regions of the HMBC spectrum (125 MHz, CD_3OD, TMS) of stepharine.

Table S2: NMR chemical shifts of stepharine. The experiments were realized in CD_3OD at magnetic field strength of 11.7 T; 500 MHz for ^1H NMR.

s: singlet; m: multiplet; dd: doublet of doublets; ddd: doublet of doublets of doublets.

Position	^1H δ_H, ppm (multiplicity; number of H; J, Hz)	^{13}C δ_C (HSQC), ppm [a]	HMBC, ppm [a]
1	-	144.6	-
1 -OCH_3	3.61 (s; 3H)	59.9	C-1
1a	-	133.8	-
2	-	154.7	-
2 -OCH_3	3.82 (s ; 3H)	55.4	C-2
3	6.89 (s ; 1H)	112.1	C-1; C-2; C-3b; C-4
3a	-	131.5	-
3b	-	129.3	-
4	3.00 (m; 2H)	23.5	-
5	3.44 (m; 1H)<br>3.69 (ddd; 1H; 13, 5.9, and 2.3 Hz)	43.7	-
6 -NH	1.95 (s)	-	-
6a	4.72 (m; 1H)	56.5	C-1a
7	2.42 (dd; 1H; 12 and 10.5 Hz)<br>2.52 (dd; 1H; 12 and 6.5 Hz)	44.9	C-1a; C-3b; C-6a; C-7a; C-8; C-12
7a	-	50.7	-
8	7.16 (dd; 1H; 10 and 3 Hz)	150.3	C-7; C-7a; C-10
9	6.29 (dd; 1H; 10 and 1.9 Hz)	126.7	C-7a
10	-	186.5	-
11	6.41 (dd; 1H; 10 and 1.9 Hz)	127.8	C-7a
12	7.02 (dd; 1H; 10 and 3 Hz)	153.6	C-10

[a] ^{13}C NMR signals were assigned using the HSQC and HMBC data.

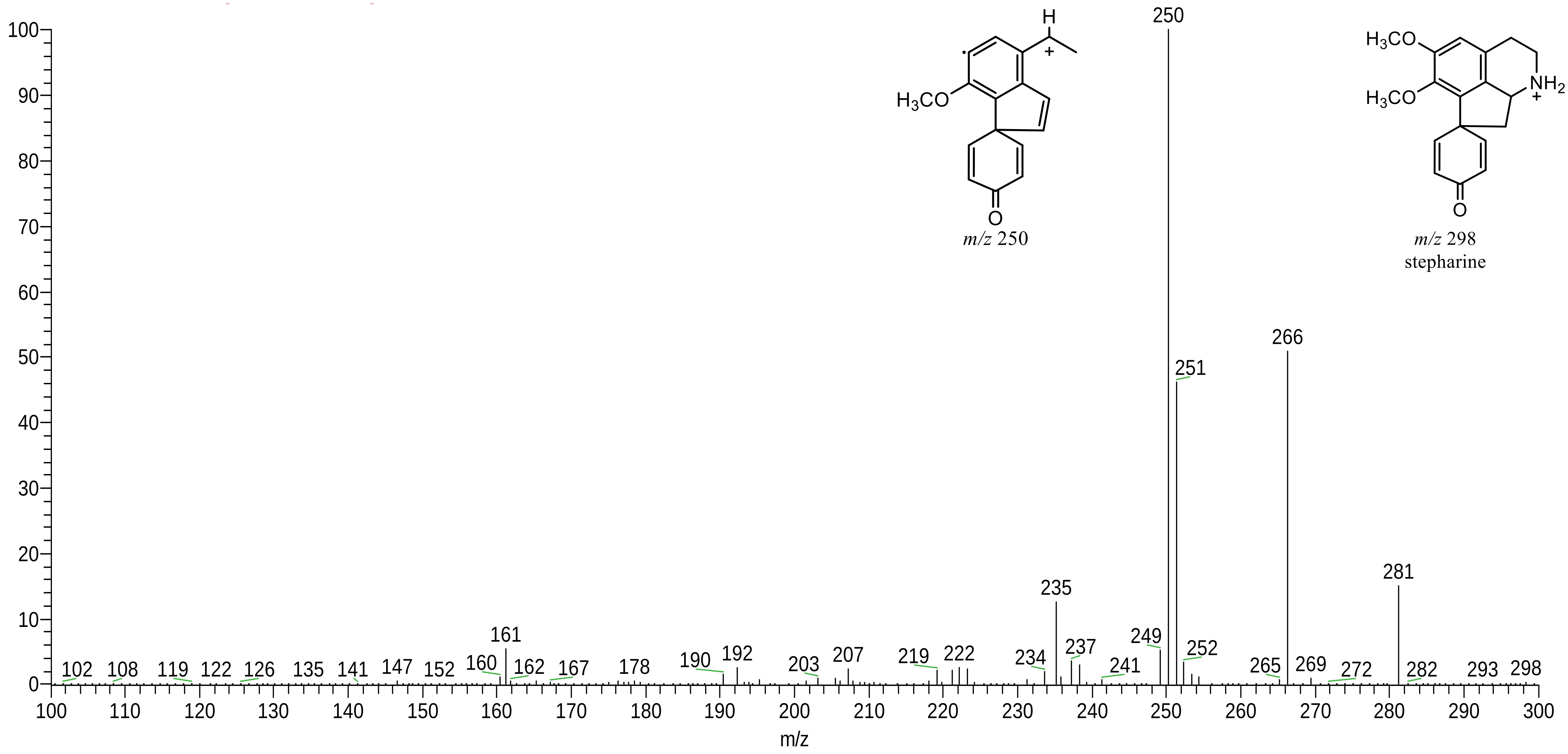

Figure S16: Mass spectrum (MS/MS) of stepharine with chemical structure and identification of the main fragment ions.

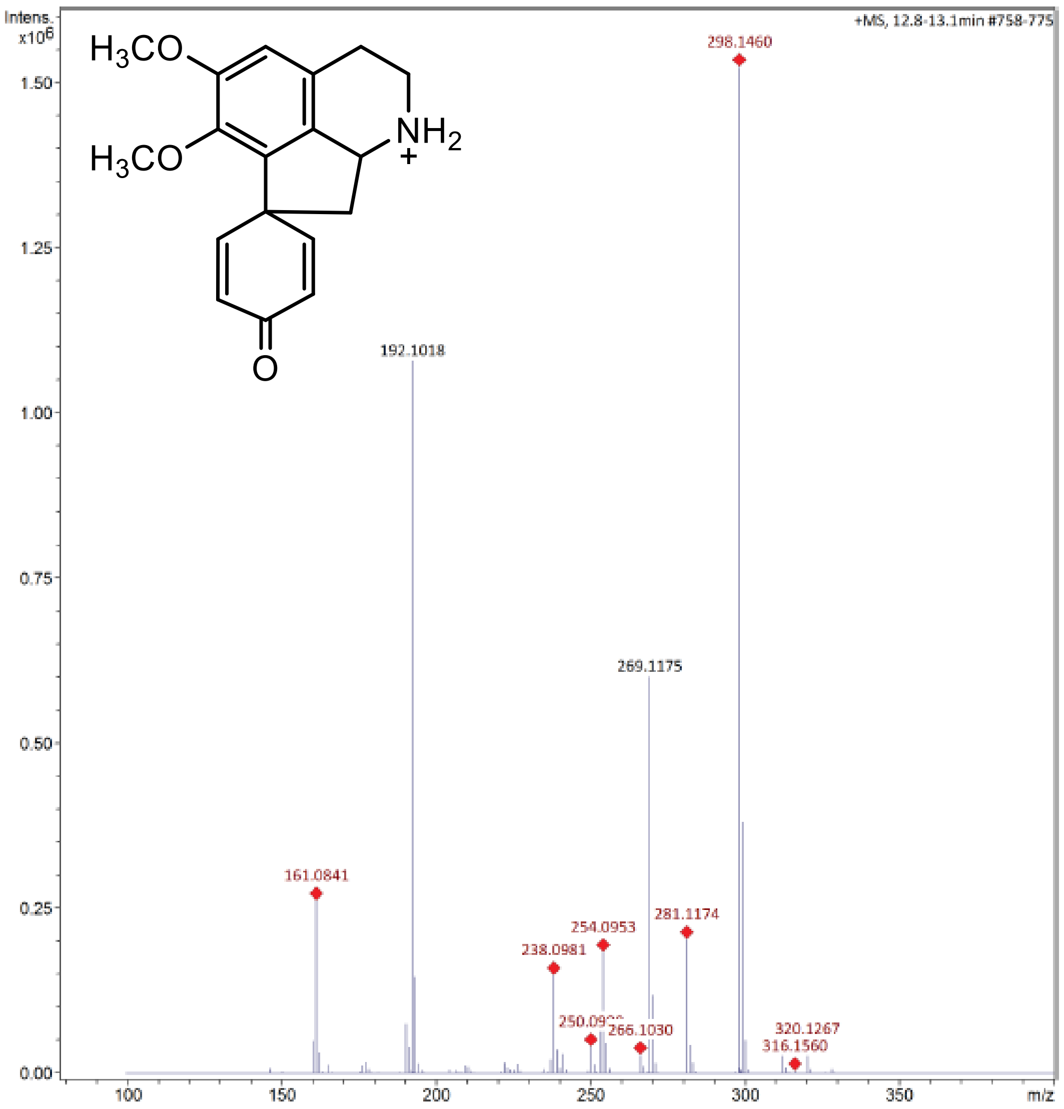

Figure S17: Hight resolution mass spectrum of stepharine with chemical structure.

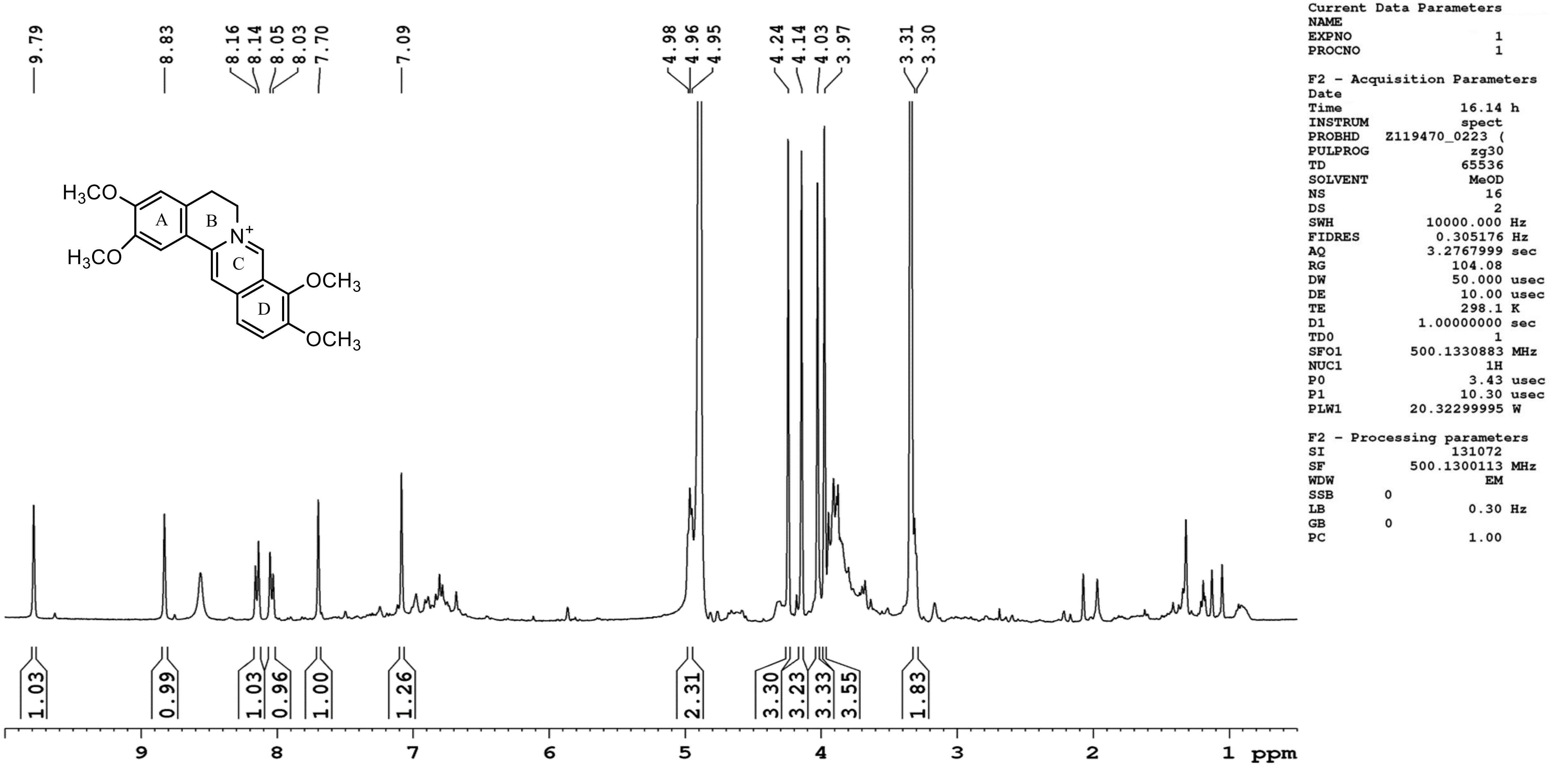

Figure S18: ^{1}H NMR spectrum (500 MHz, CD_3OD d_6, TMS) of palmatine.

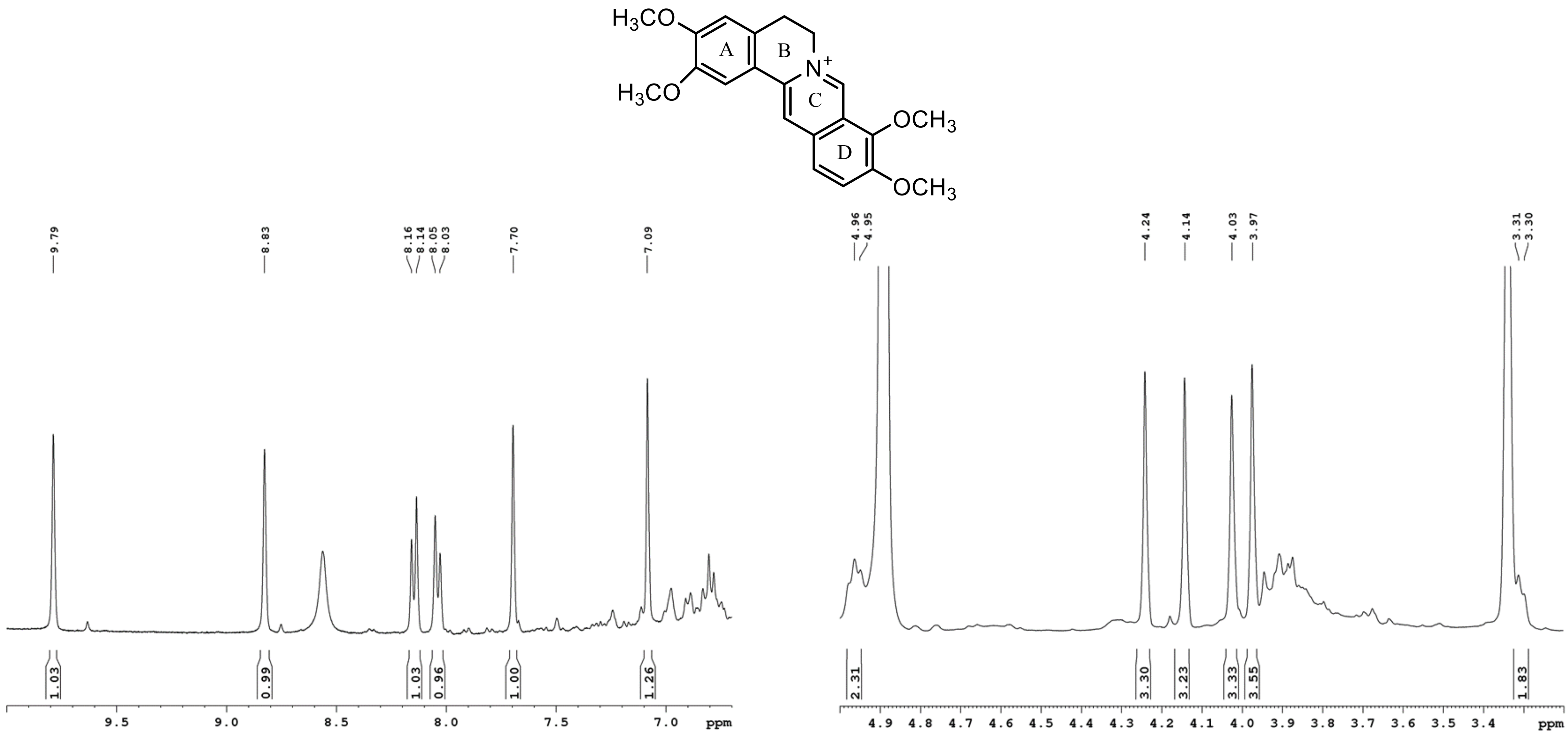

Figure S19: Expanded low-field region of the ^{1}H NMR spectrum (500 MHz, CD_3OD d_6, TMS) of palmatine.

```
Current Data Parameters
NAME
EXPNO                200
PROCNO                 1

F2 - Acquisition Parameters
Date
Time               23.25 h
INSTRUM            spect
PROBHD   Z119470_0223 (
PULPROG  hsqcetgpsisp2.2
TD                  2048
SOLVENT             MeOD
NS                    64
DS                    16
SWH             6996.269 Hz
FIDRES          3.416147 Hz
AQ             0.1463637 sec
RG                187.25
DW                71.467 usec
DE                 10.00 usec
TE                 298.2 K
CNST2        145.0000000
CNST17        -0.5000000
D0            0.00000300 sec
D1            1.00000000 sec
D4            0.00172414 sec
D11           0.03000000 sec
D16           0.00020000 sec
D24           0.00089000 sec
IN0           0.00001850 sec
TDav                   1
SFO1         500.1325097 MHz
NUC1                  1H
P1                  9.40 usec
P2                 18.80 usec
P28              1000.00 usec
PLW1         20.32299995 W
SFO2         125.7703643 MHz
NUC2                 13C
CPDPRG[2            garp
P3                 10.00 usec
P14               500.00 usec
P24              2000.00 usec
PCPD2              70.00 usec
PLW0     0 W
PLW2         88.00000000 W
PLW12         1.78668594 W
SPNAM[3] Crp60,0.5,20.1
SPOAL3             0.500
SPOFFS3  0 Hz
SPW3         13.44499969 W
SPNAM[7]     Crp60comp.4
SPOAL7             0.500
SPOFFS7  0 Hz
SPW7         13.44499969 W
GPNAM[1]      SMSQ10.100
GPZ1               80.00 %
GPNAM[2]      SMSQ10.100
GPZ2               20.10 %
GPNAM[3]      SMSQ10.100
GPZ3               11.00 %
GPNAM[4]      SMSQ10.100
GPZ4               -5.00 %
P16              1000.00 usec
P19               600.00 usec

F1 - Acquisition parameters
TD                   256
SFO1         125.7704 MHz
FIDRES     211.148651 Hz
SW            214.892 ppm
FnMODE   Echo-Antiecho

F2 - Processing parameters
SI                  4096
SF           500.1300115 MHz
WDW                QSINE
SSB                    2
LB       0 Hz
GB       0
PC                  1.40

F1 - Processing parameters
SI                  1024
MC2        echo-antiecho
SF           125.7577885 MHz
WDW                QSINE
SSB                    2
LB       0 Hz
GB       0
```

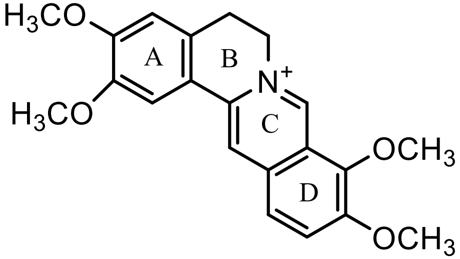

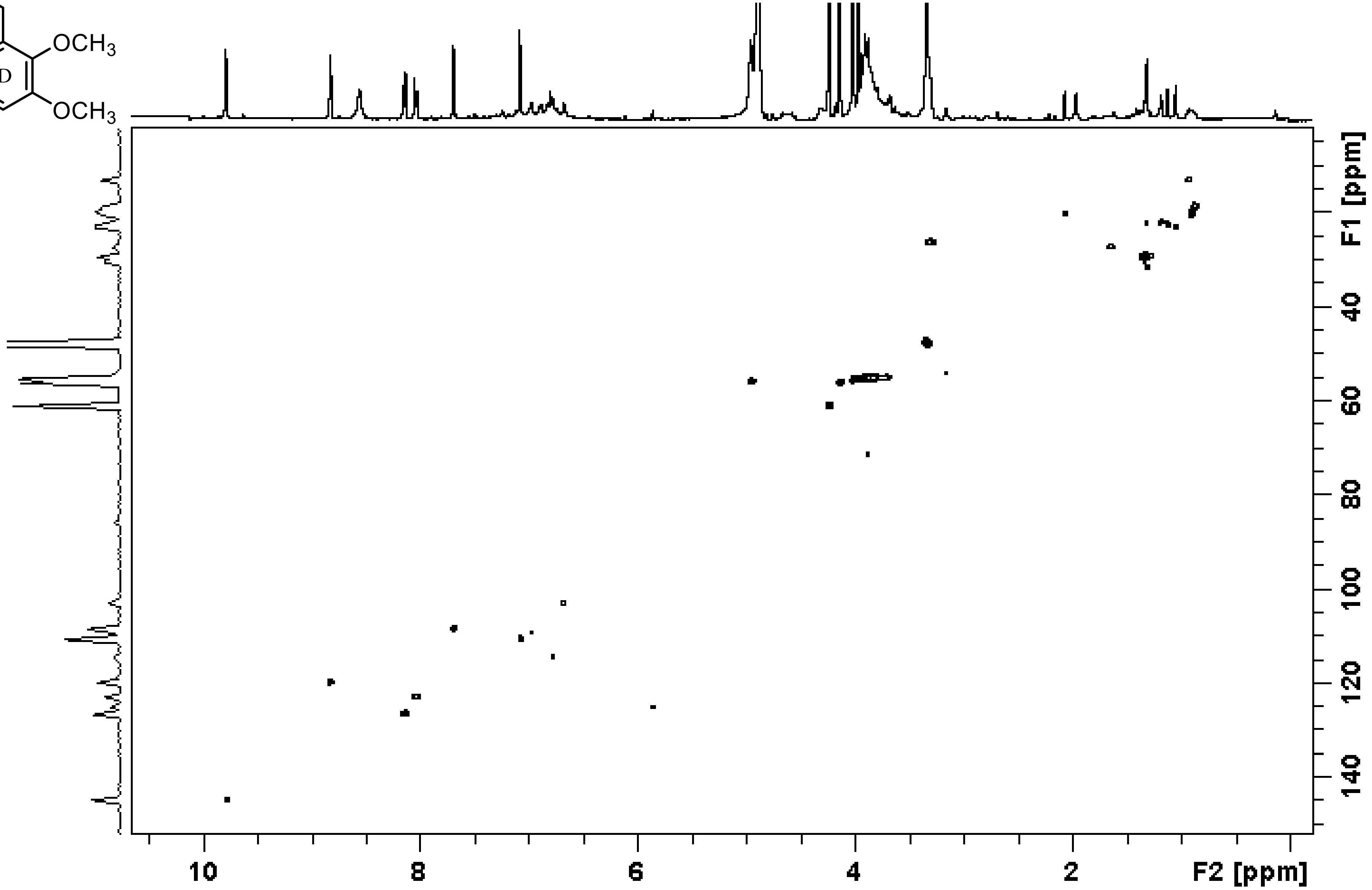

Figure S20: HSQC spectrum (125 MHz, CD_3OD, TMS) of palmatine.

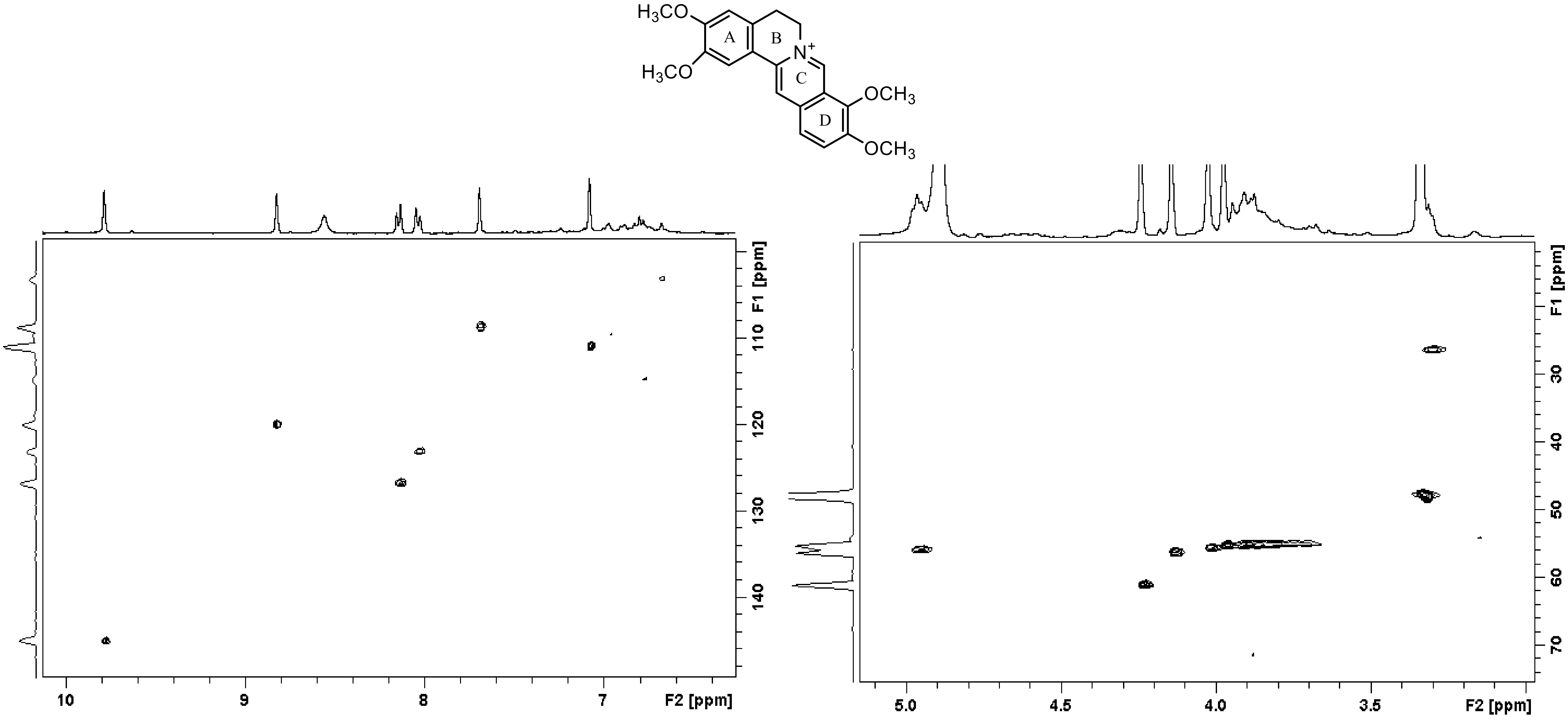

Figure S21: Expanded regions of the HSQC spectrum (125 MHz, CD_3OD, TMS) of palmatine.

```
Current Data Parameters
NAME
EXPNO                    300
PROCNO                     1

F2 - Acquisition Parameters
Date
Time                    4.46 h
INSTRUM                spect
PROBHD   Z119470_0223 (
PULPROG     hmbcgplpndqf
TD                      2048
SOLVENT                 MeOD
NS                        98
DS                        16
SWH                 7002.801 Hz
FIDRES              3.419337 Hz
AQ                 0.1462272 sec
RG                    187.25
DW                    71.400 usec
DE                     10.00 usec
TE                     298.2 K
CNST2            145.0000000
CNST13             8.0000000
D0                0.00000300 sec
D1                1.00000000 sec
D2                0.00344828 sec
D6                0.06250000 sec
D16               0.00020000 sec
IN0               0.00001660 sec
TDav                       1
SFO1             500.1325097 MHz
NUC1                      1H
P1                      9.40 usec
P2                     18.80 usec
PLW1             20.32299995 W
SFO2             125.7716219 MHz
NUC2                     13C
P3                     10.00 usec
PLW2             88.00000000 W
GPNAM[1]           SMSQ10.100
GPZ1                   50.00 %
GPNAM[2]           SMSQ10.100
GPZ2                   30.00 %
GPNAM[3]           SMSQ10.100
GPZ3                   40.10 %
P16                  1000.00 usec

F1 - Acquisition parameters
TD                       312
SFO1                125.7716 MHz
FIDRES            193.080017 Hz
SW                   239.486 ppm
FnMODE                    QF

F2 - Processing parameters
SI                      4096
SF               500.1300115 MHz
WDW                     SINE
SSB       0
LB        0 Hz
GB        0
PC                      1.40

F1 - Processing parameters
SI                      1024
MC2                       QF
SF               125.7577885 MHz
WDW                     SINE
SSB       0
LB        0 Hz
GB        0
```

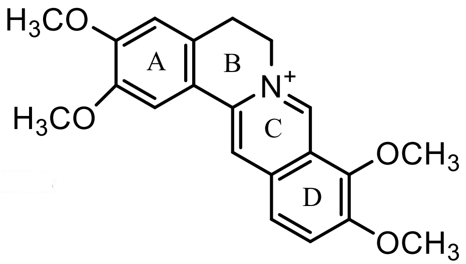

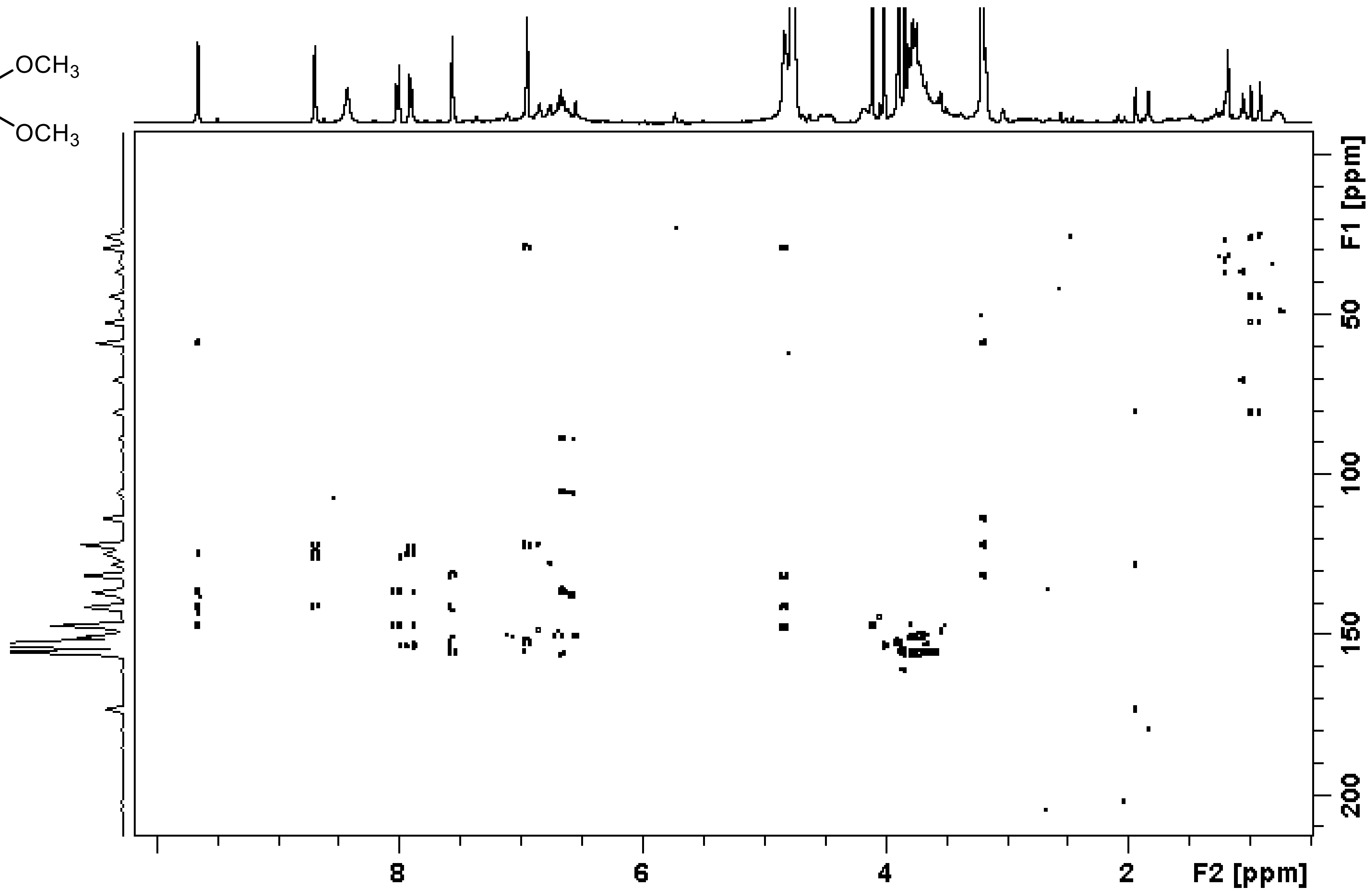

Figure S22: HMBC spectrum (125 MHz, CD_3OD, TMS) of palmatine.

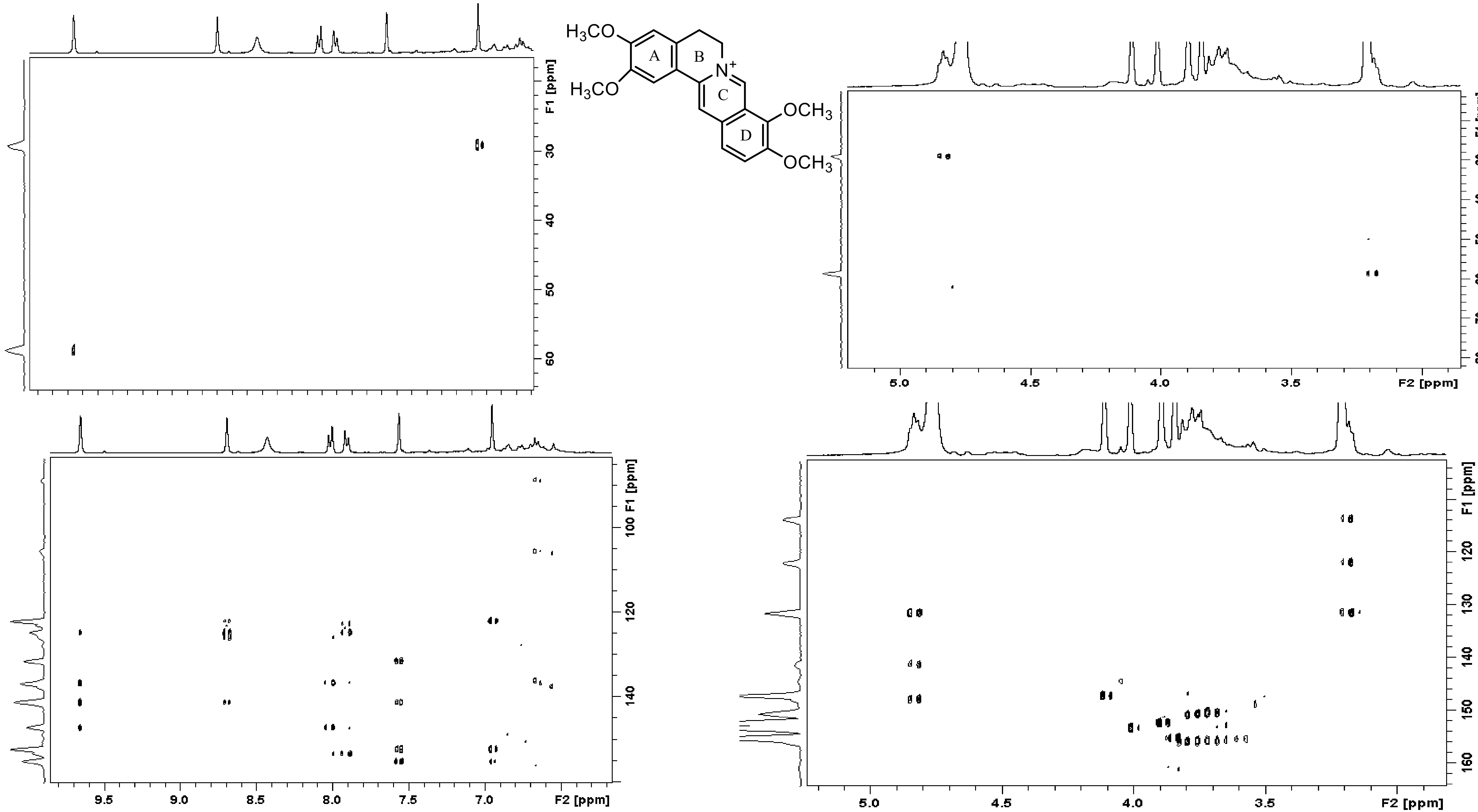

Figure S23: Expanded regions of the HMBC spectrum (125 MHz, CD_3OD, TMS) of palmatine.

Table S3: NMR chemical shifts of palmatine. The experiments were realized in CD_3OD at magnetic field strength of 11.7 T; 500 MHz for ^1H NMR.

s: singlet; m: multiplet; dd: doublet of doublets; ddd: doublet of doublets of doublets.

Position	^1H δ_H, ppm (multiplicity; number of H; J, Hz)	^{13}C δ_C (HSQC), ppm [a]	HMBC, ppm [a]
1	7.08 (s; 1H)	110.6	C-5; C-4a; C-2; C-3
2	-	152.2	-
2-OCH_3	4.02 (s; 3H)	57.7	C-2
3	-	155.1	-
3-OCH_3	3.97 (s; 3H)	57.2	C-3
4	7.69 (s; 1H)	112.8	C-13b; C-13a; C-2; C-3
4a	-	121.9	-
5	3.30 (m; 2H)	29.1	C-6; C-1; C-4; C-4a; C-13b
6	4.96 (m; 2H)	58.5	C-5; C-13b; C-13a; C-8
7	-	-	-
8	9.79 (s; 1H)	146.9	C-6; C-13a; C-12; C-8a; C-9
8a	-	136.6	-
9	-	147.1	-
9-OCH_3	4.24 (s; 3H)	63.2	C-9
10	-	153.3	-
10-OCH_3	4.14 (s; 3H)	58.5	C-10
11	8.14 (d;1H; 9.3 Hz)	128.7	C-12;C-8a; C-9; C-10
12	8.04 (d; 1H; 8.8 Hz)	125.1	C-12a; C-8a; C-9; C-10
12a	-	122.7	-
13	8.82 (s; 1H)	121.8	C-12; C-13a
13a	-	141.2	-
13b	-	131.5	-

[a] ^{13}C NMR signals were assigned using the HSQC and HMBC data.

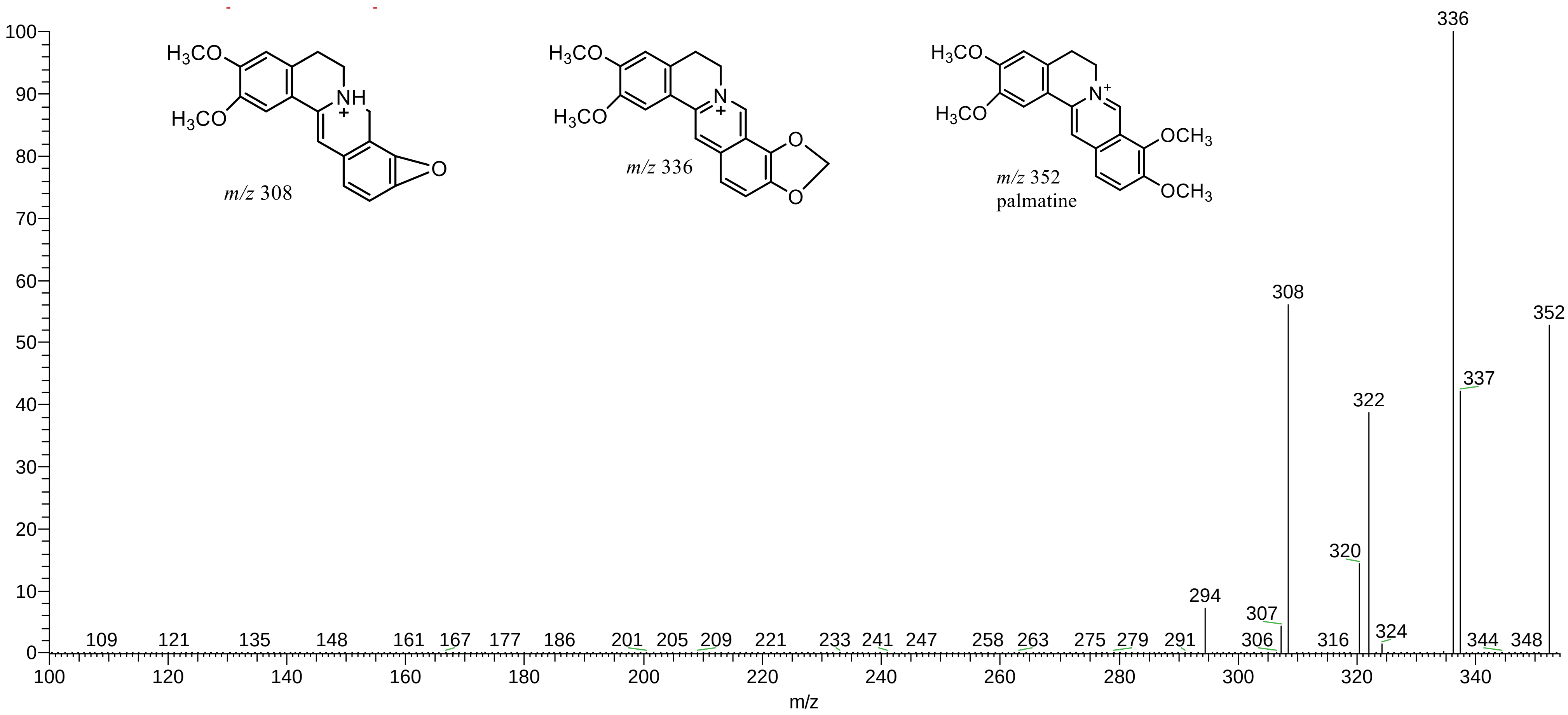

Figure S24: Mass spectrum (MS/MS) of palmatine with chemical structure and identification of the main fragment ions.

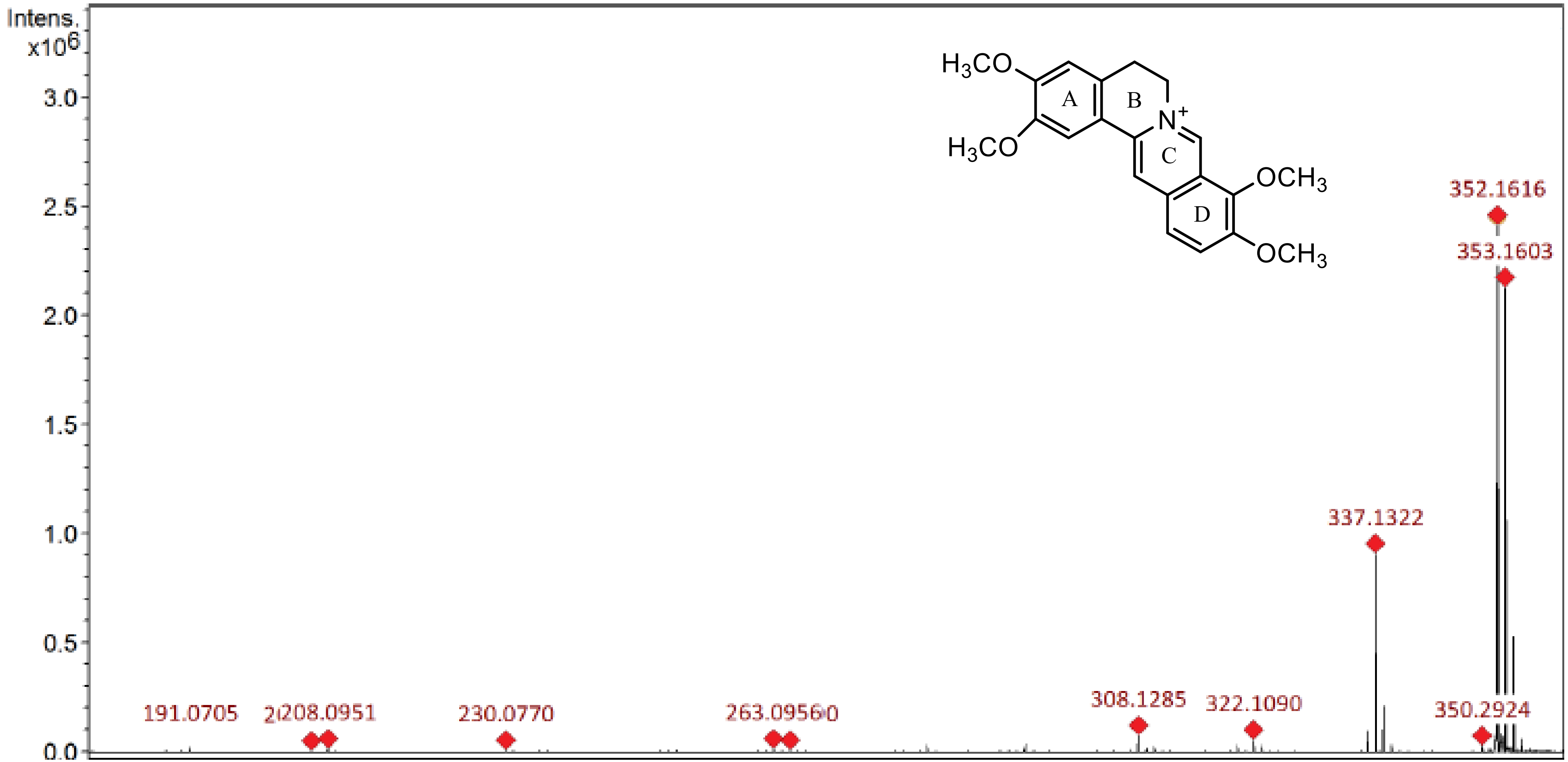

Figure S25: Hight resolution mass-spectrum of palmatine with chemical structure.

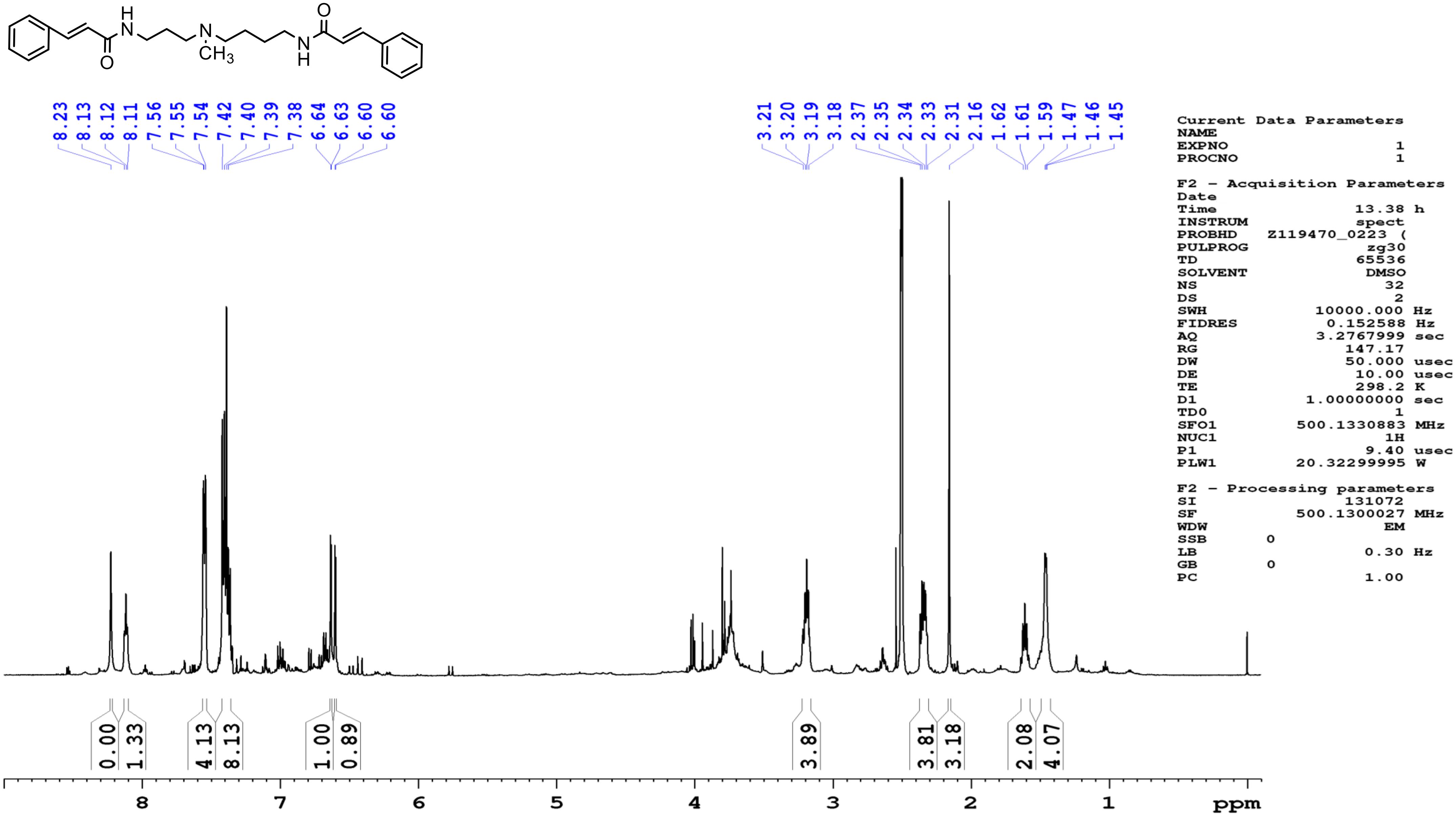

Figure S26: ^{1}H NMR spectrum (500 MHz, DMSO d_6, TMS) of 5-*N*-methylmaytenine.

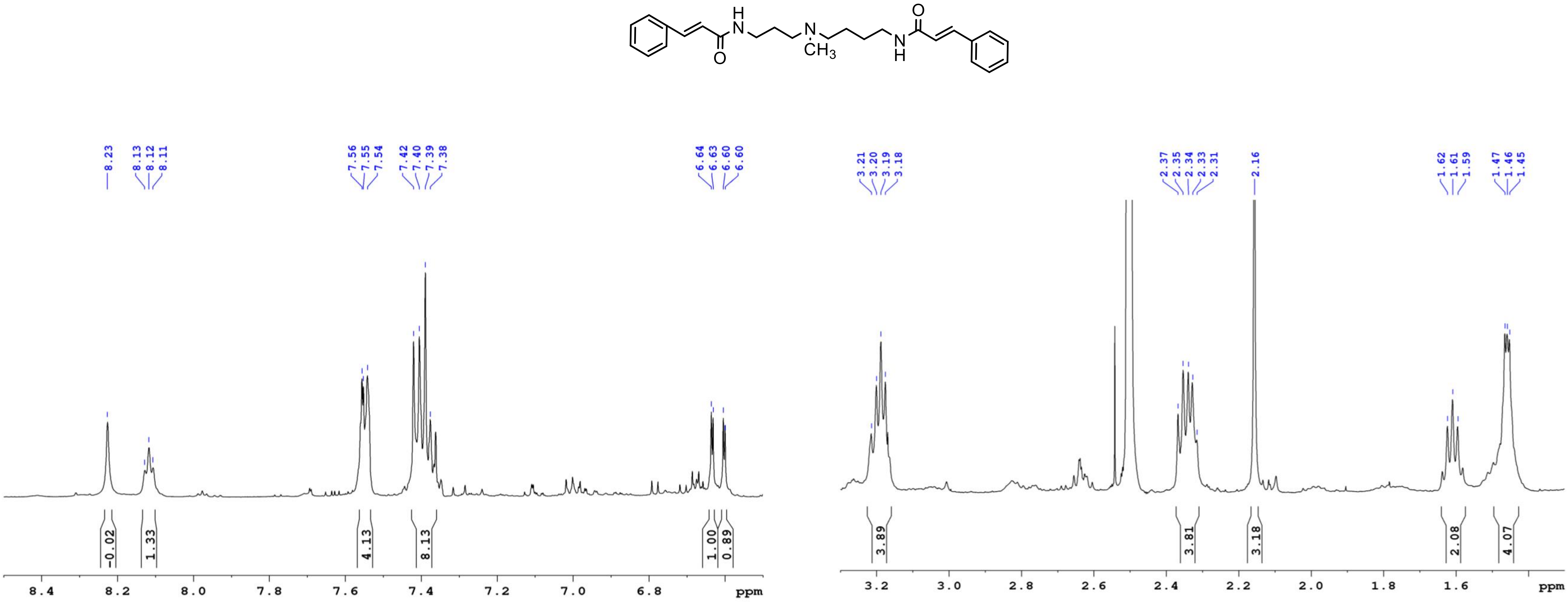

Figure S27: Expanded low-field region of the ^{1}H NMR spectrum (500 MHz, DMSO d_6, TMS) of 5-*N*-methylmaytenine.

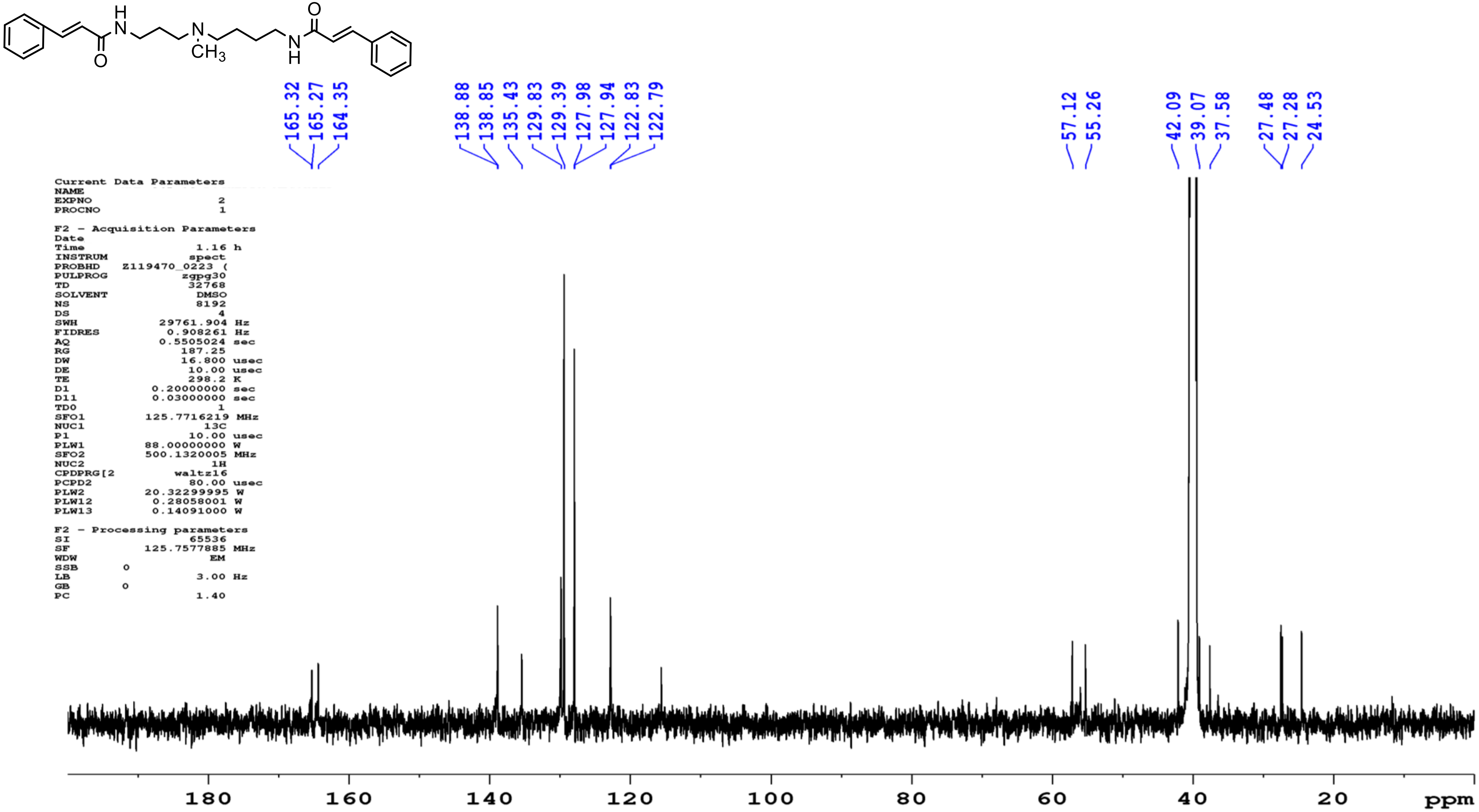

Figure S28: ^{13}C NMR spectrum (125 MHz, DMSO d_6, TMS) of 5-*N*-methylmaytenine.

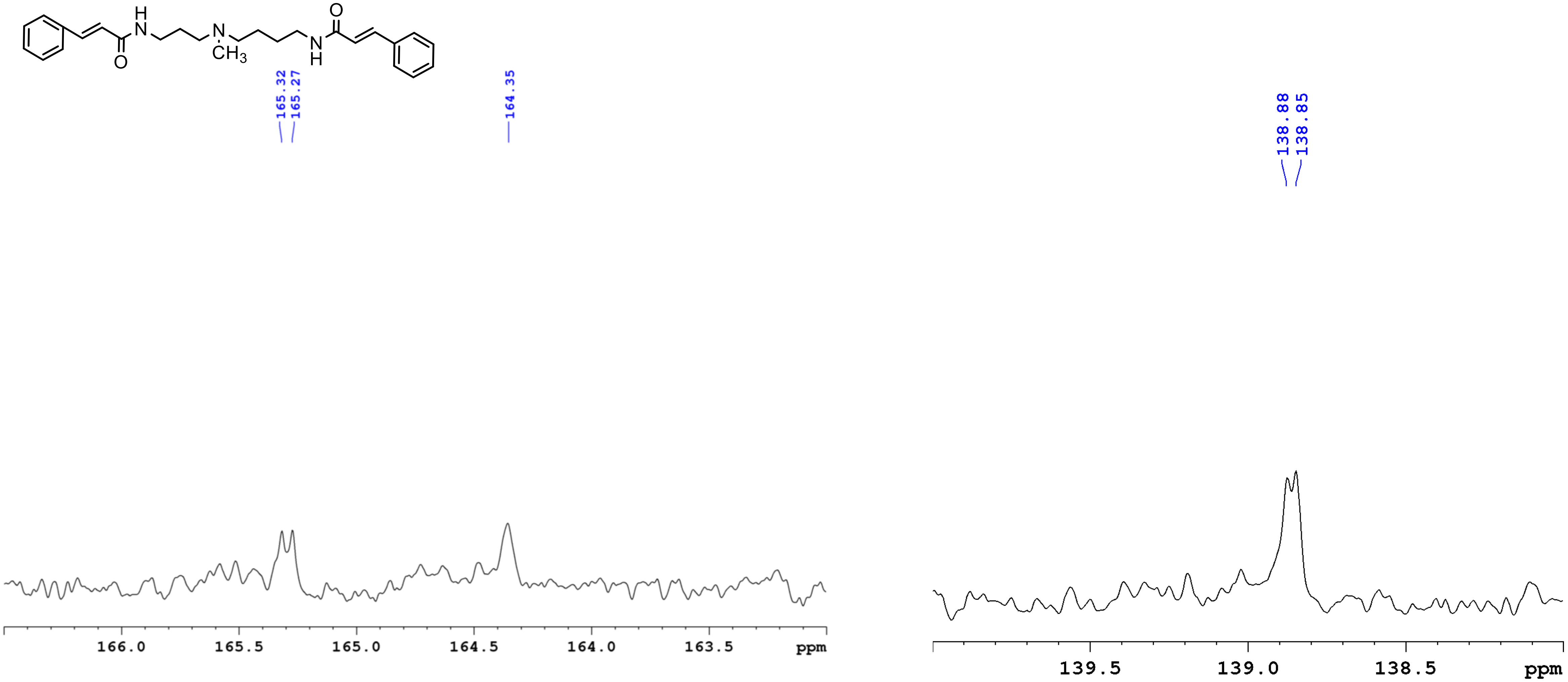

Figure S29: Expanded regions of the^{13}C NMR spectrum (125 MHz, DMSO d_6, TMS) of 5-*N*-methylmaytenine.

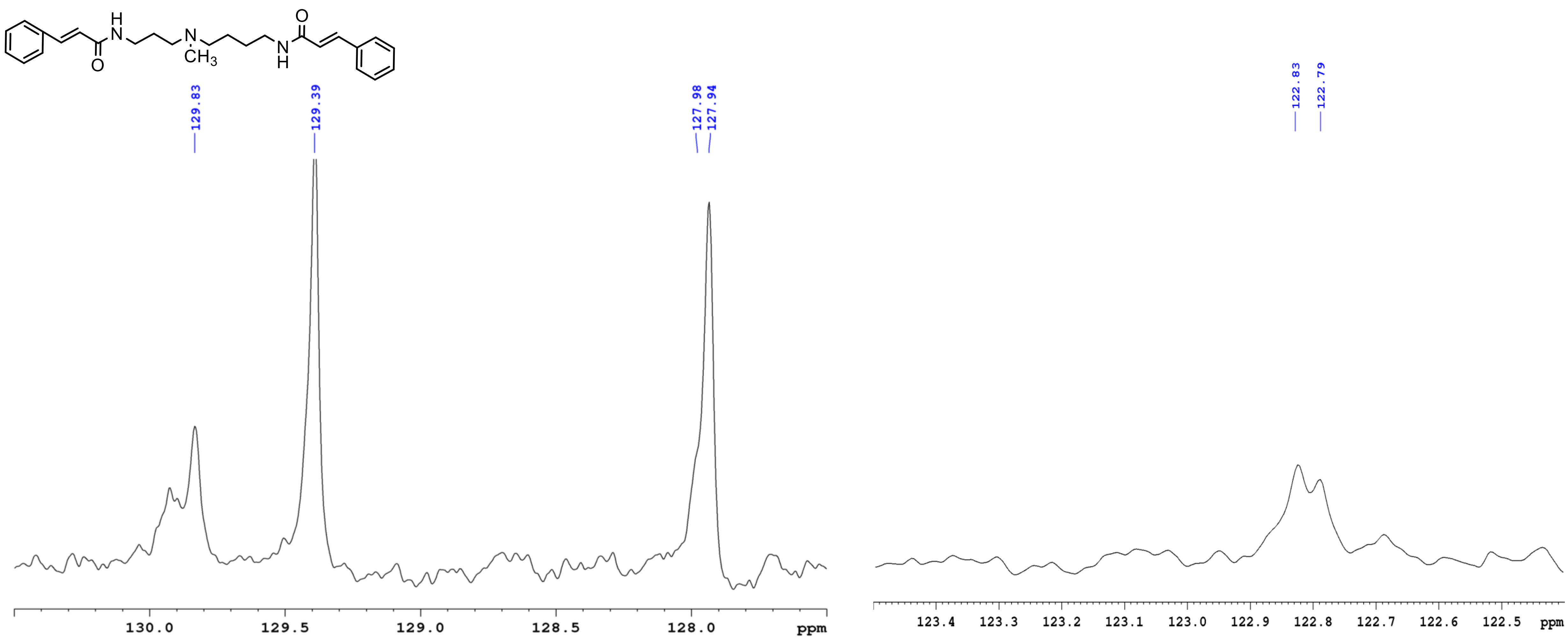

Figure S30: Expanded regions of the ^{13}C NMR spectrum (125 MHz, DMSO d_6, TMS) of 5-*N*-methylmaytenine.

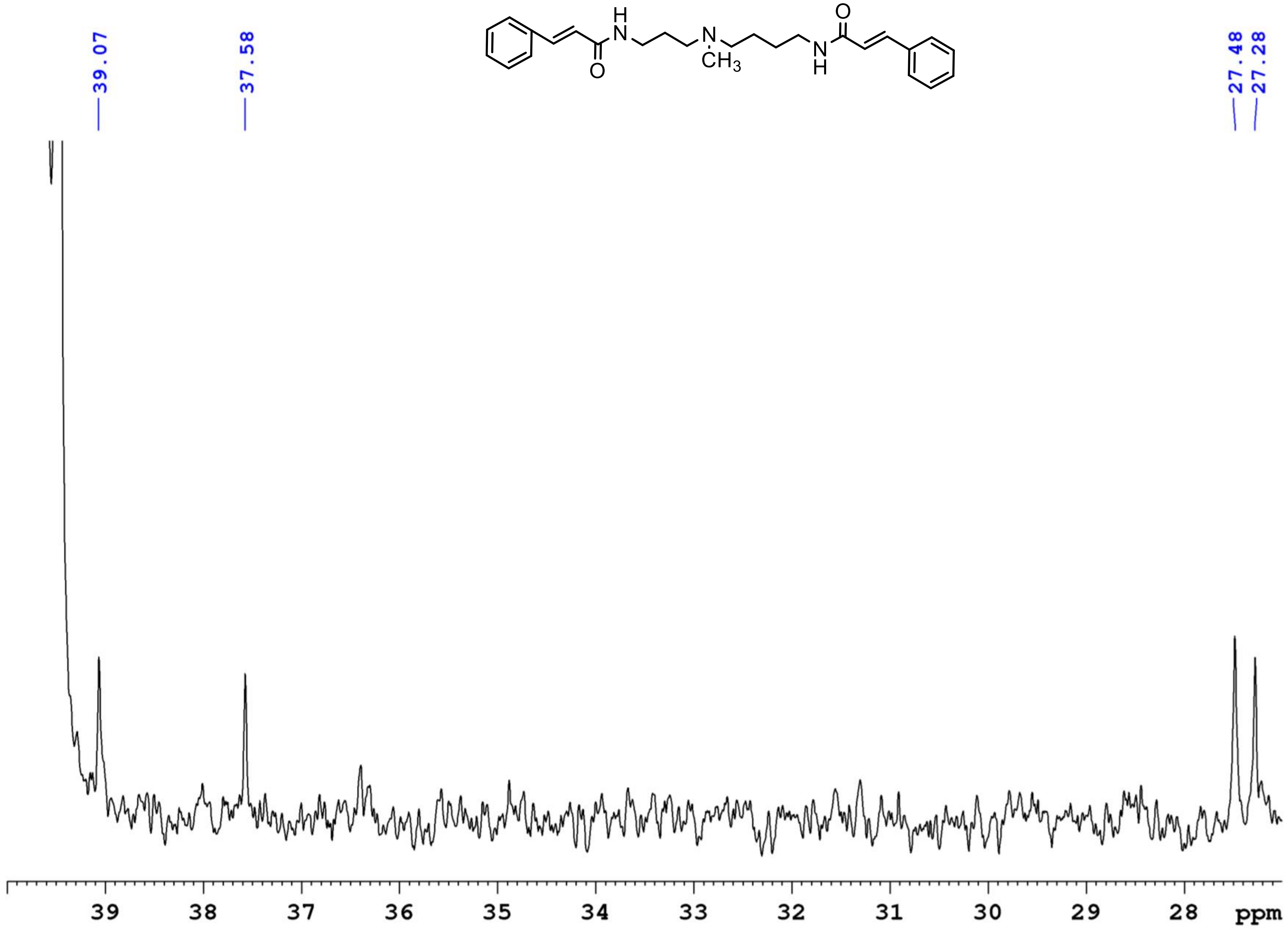

Figure S31: Expanded region of the ^{13}C NMR spectrum (125 MHz, DMSO d_6, TMS) of 5-*N*-methylmaytenine.

```
Current Data Parameters
NAME
EXPNO                  300
PROCNO                   1

F2 - Acquisition Parameters
Date
Time                  5.23 h
INSTRUM              spect
PROBHD    Z119470_0223 (
PULPROG     hmbcgplpndqf
TD                    2048
SOLVENT               DMSO
NS                      52
DS                      16
SWH               5980.861 Hz
FIDRES            2.920342 Hz
AQ               0.1712128 sec
RG                  187.25
DW                  83.600 usec
DE                   10.00 usec
TE                   298.1 K
CNST2          145.0000000
CNST13           8.0000000
D0              0.00000300 sec
D1              1.00000000 sec
D2              0.00344828 sec
D6              0.06250000 sec
D16             0.00020000 sec
IN0             0.00001660 sec
TDav                     1
SFO1           500.1325072 MHz
NUC1                    1H
P1                    9.40 usec
P2                   18.80 usec
PLW1           20.32299995 W
SFO2           125.7716219 MHz
NUC2                   13C
P3                   10.00 usec
PLW2           88.00000000 W
GPNAM[1]        SMSQ10.100
GPZ1                 50.00 %
GPNAM[2]        SMSQ10.100
GPZ2                 30.00 %
GPNAM[3]        SMSQ10.100
GPZ3                 40.10 %
P16                1000.00 usec

F1 - Acquisition parameters
TD                     235
SFO1              125.7716 MHz
FIDRES          256.344513 Hz
SW                 239.486 ppm
FnMODE                  QF

F2 - Processing parameters
SI                    4096
SF             500.1300000 MHz
WDW                   SINE
SSB       0
LB        0 Hz
GB        0
PC                    1.40

F1 - Processing parameters
SI                    1024
MC2                     QF
SF             125.7577885 MHz
WDW                   SINE
SSB       0
LB        0 Hz
GB        0
```

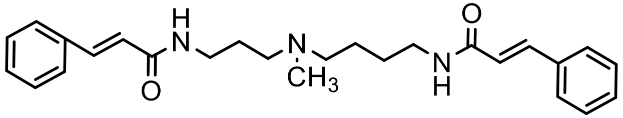

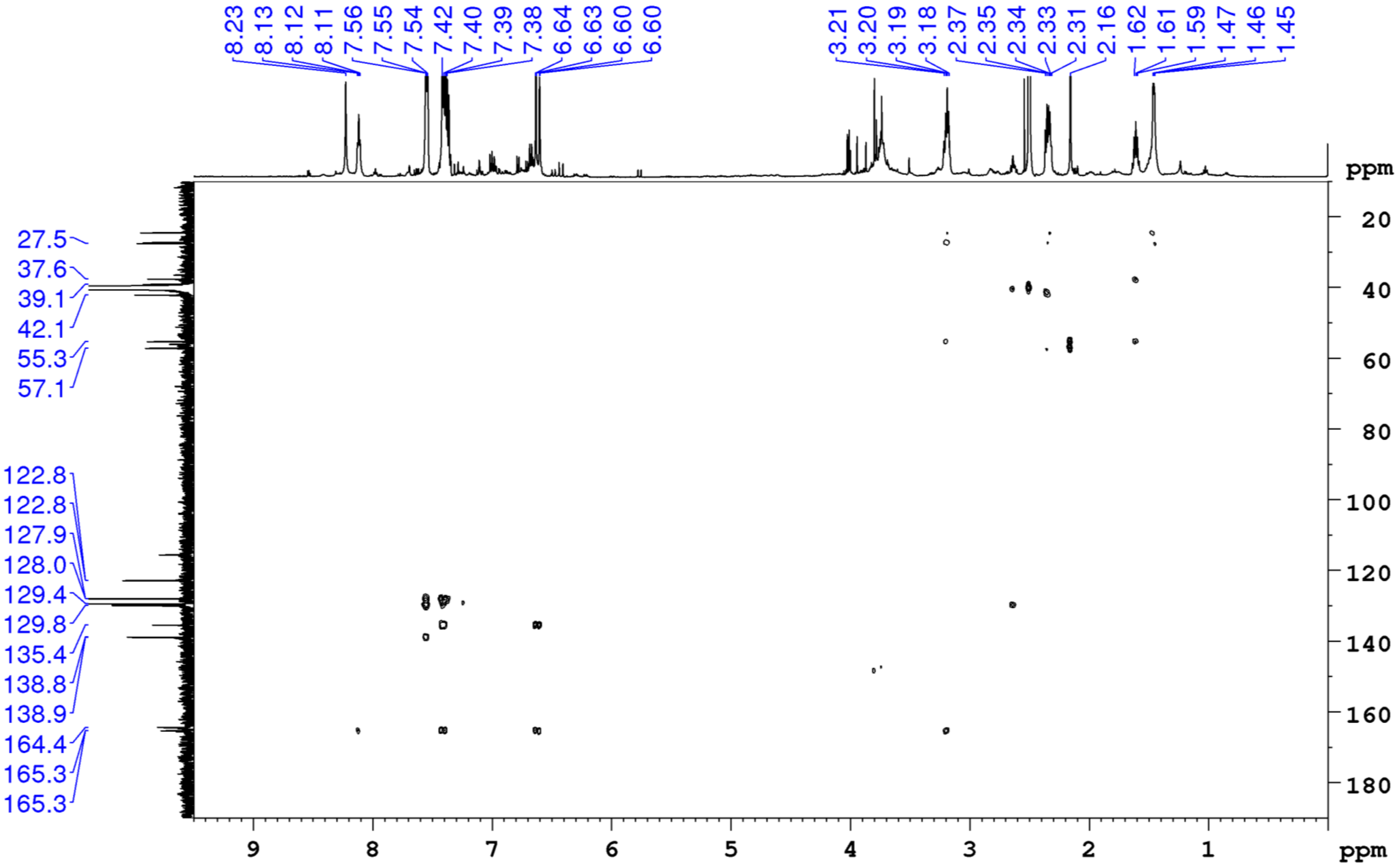

Figure S32: HMBC spectrum (125 MHz, DMSO d_6, TMS) of 5-*N*-methylmaytenine.

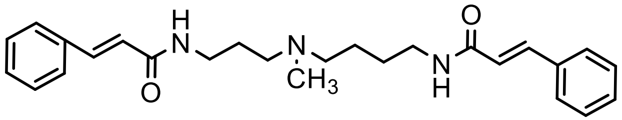

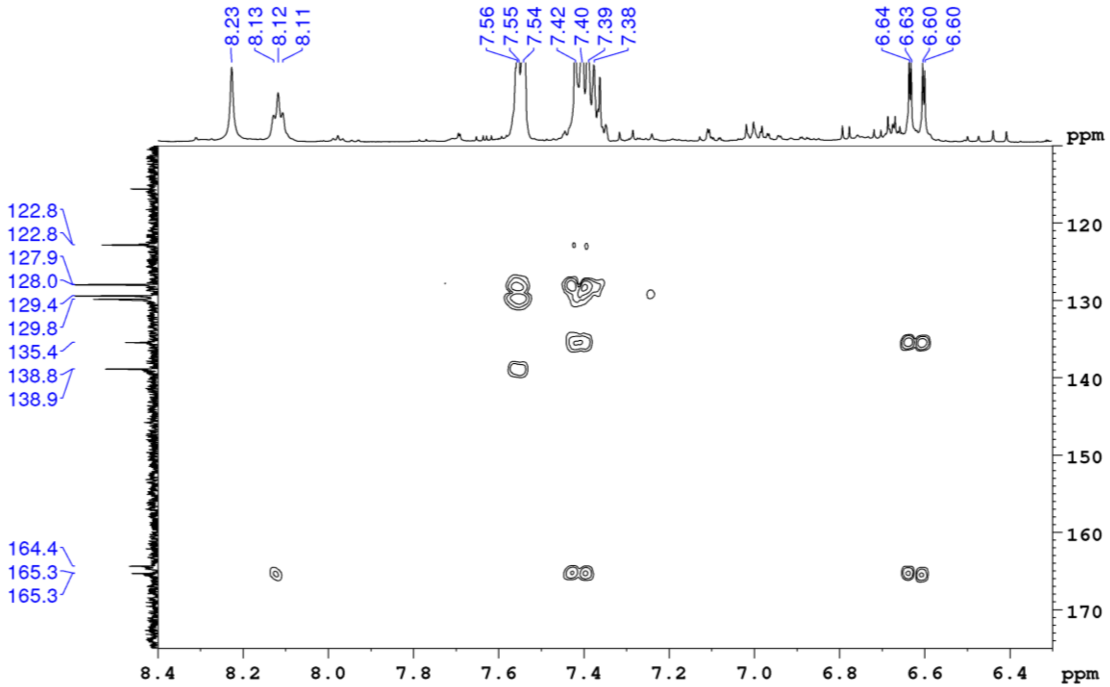

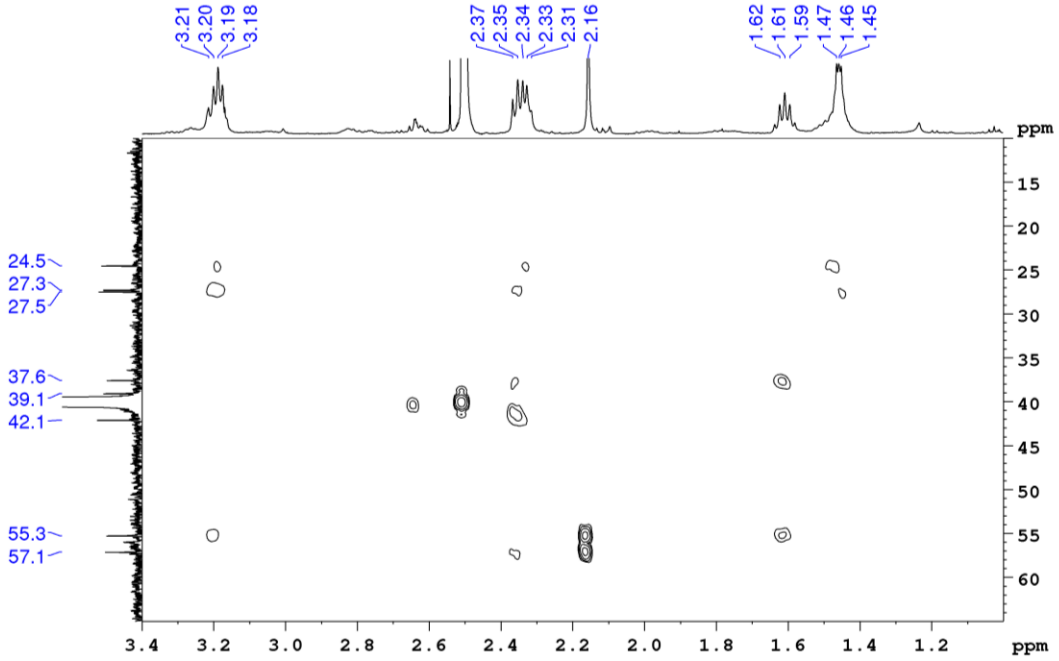

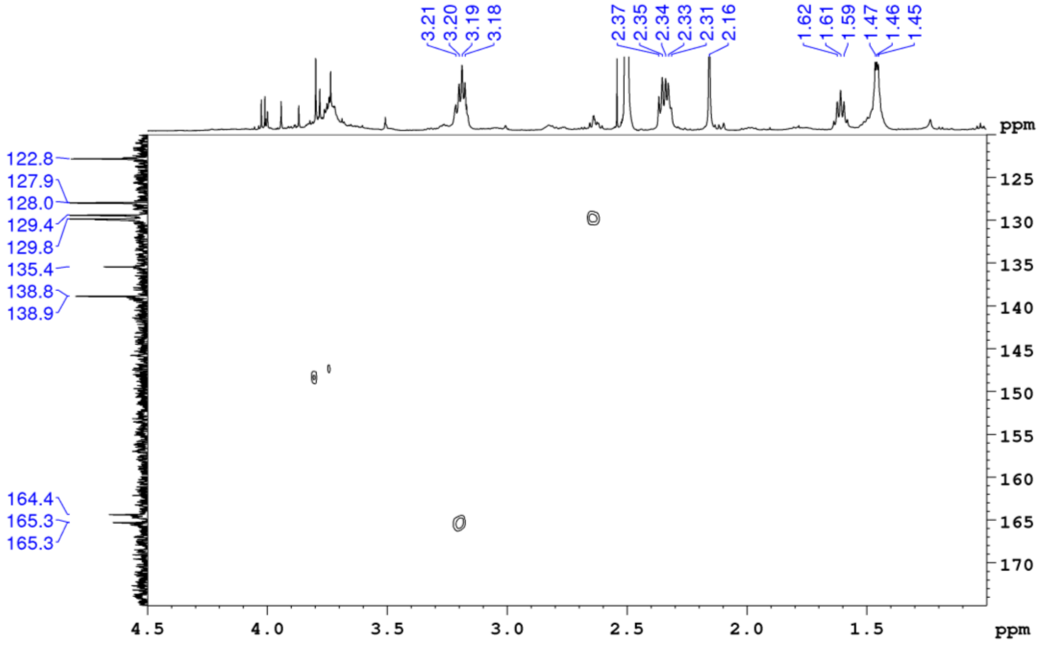

Figure S33: Expanded regions of the HMBC spectrum (125 MHz, DMSO d$_6$, TMS) of 5-*N*-methylmaytenine.

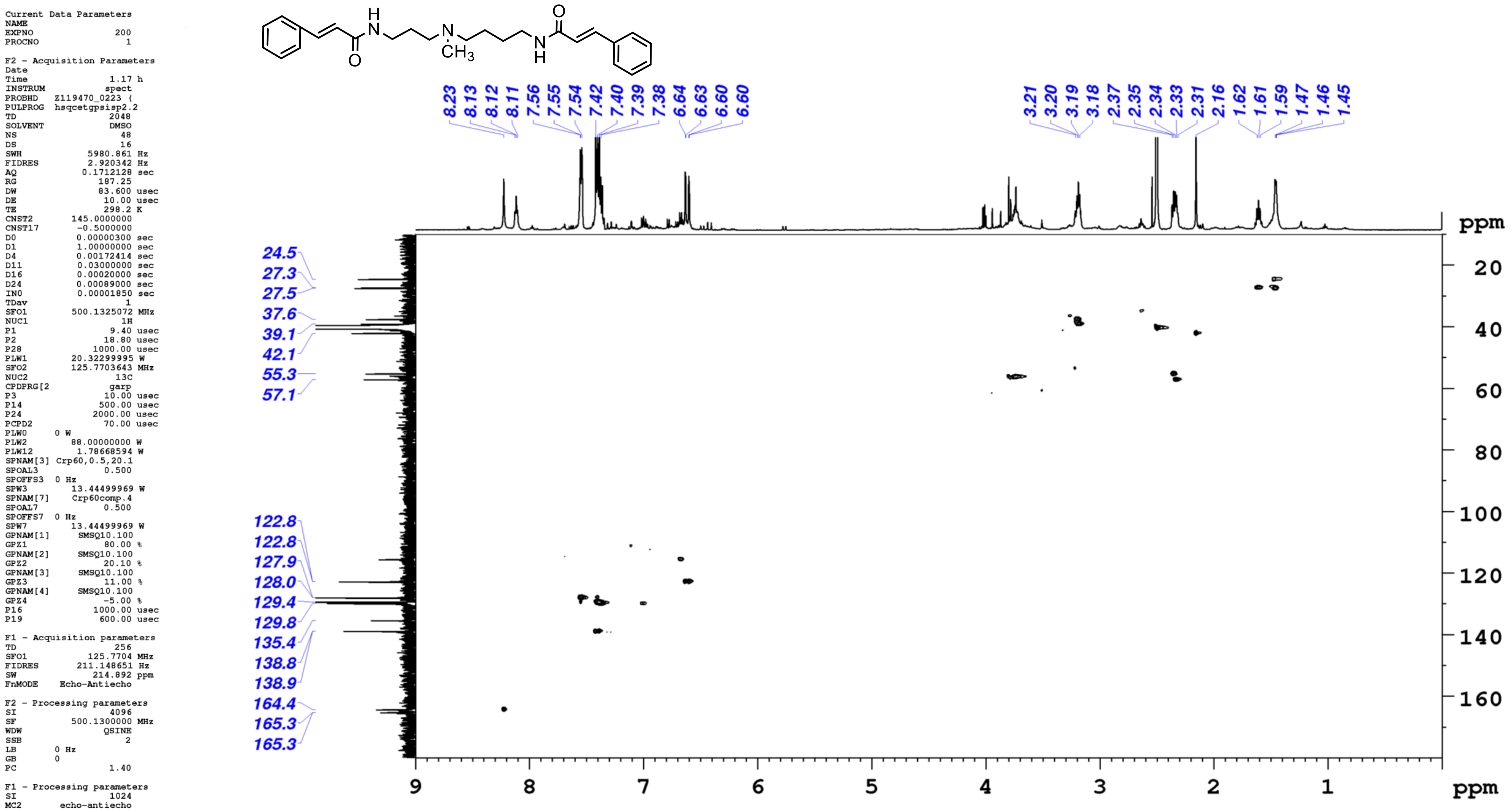

Figure S34: HSQC spectrum (125 MHz, DMSO d_6, TMS) of 5-*N*-methylmaytenine.

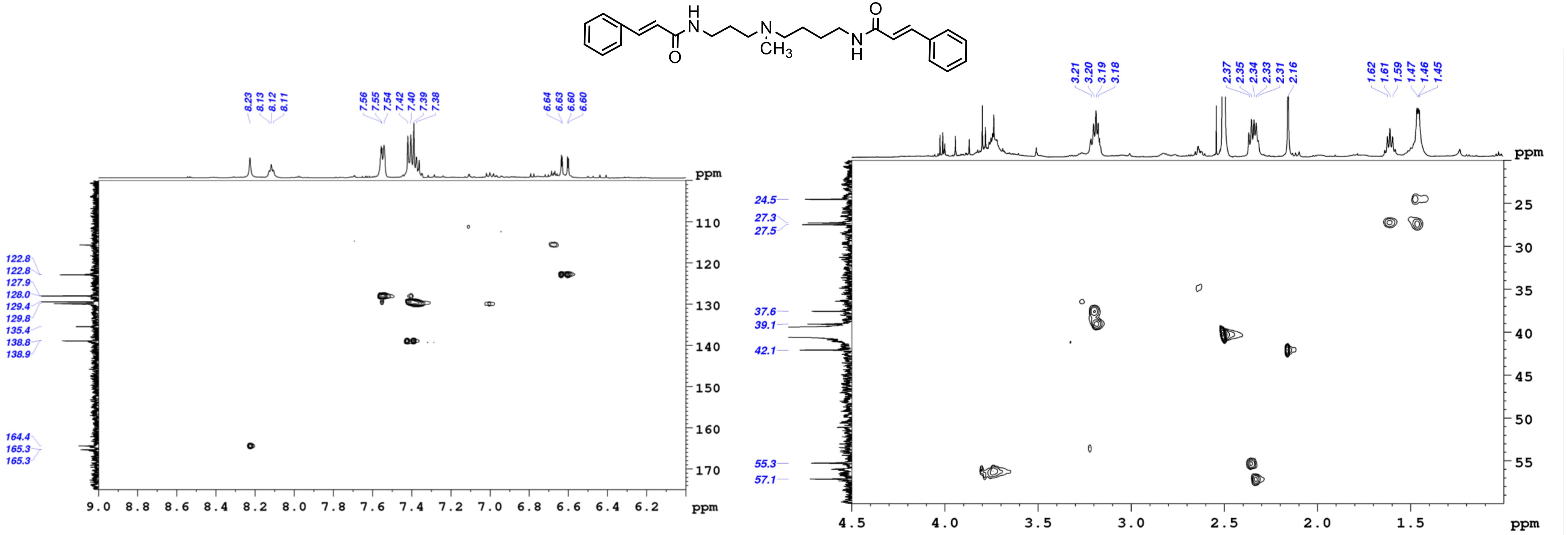

Figure S35: Expanded regions of the HSQC spectrum (125 MHz, DMSO d_6, TMS) of 5-*N*-methylmaytenine.

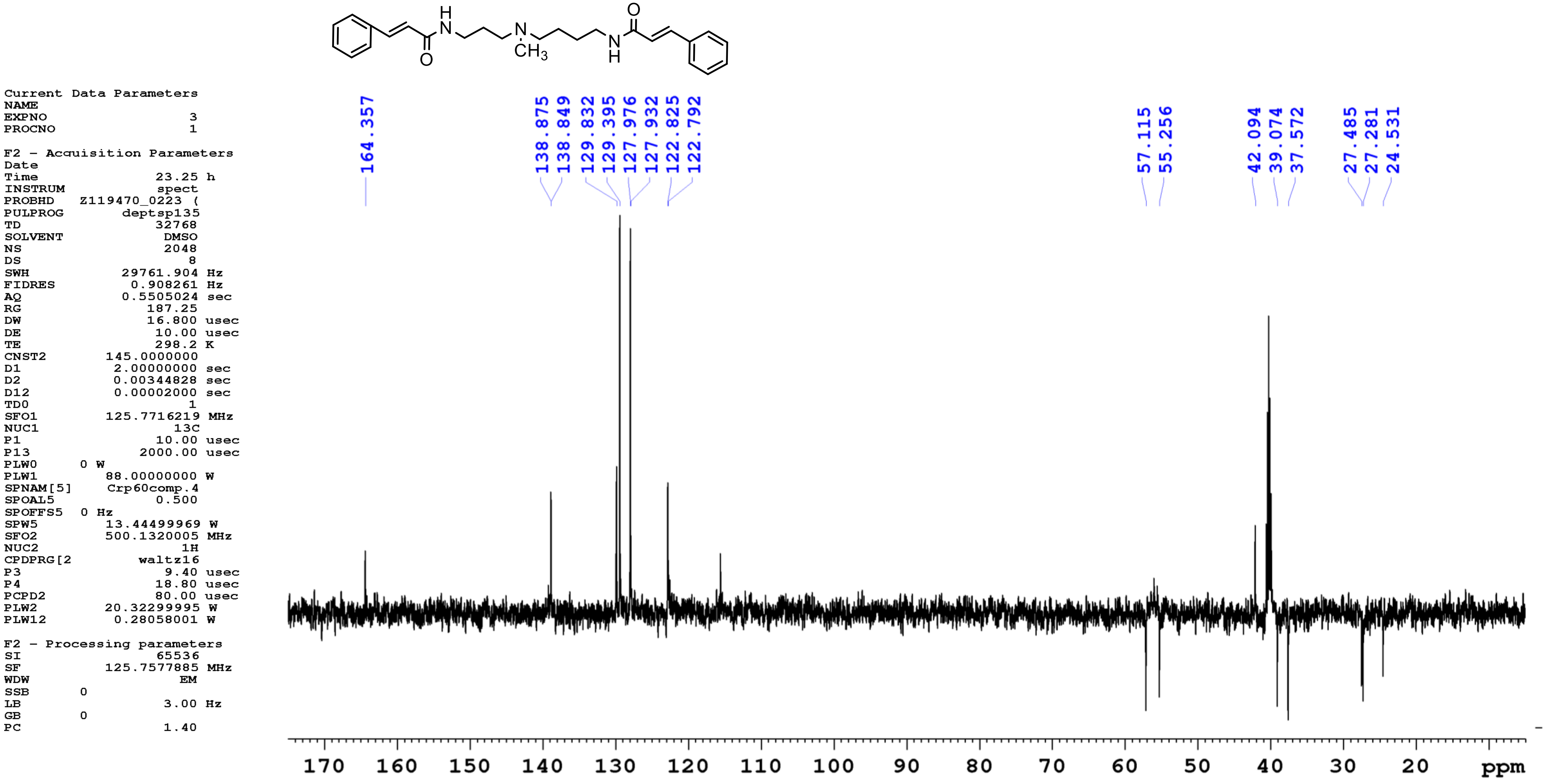

Figure S36: DEPT-135º spectrum (125 MHz, DMSO d_6, TMS) of 5-*N*-methylmaytenine.

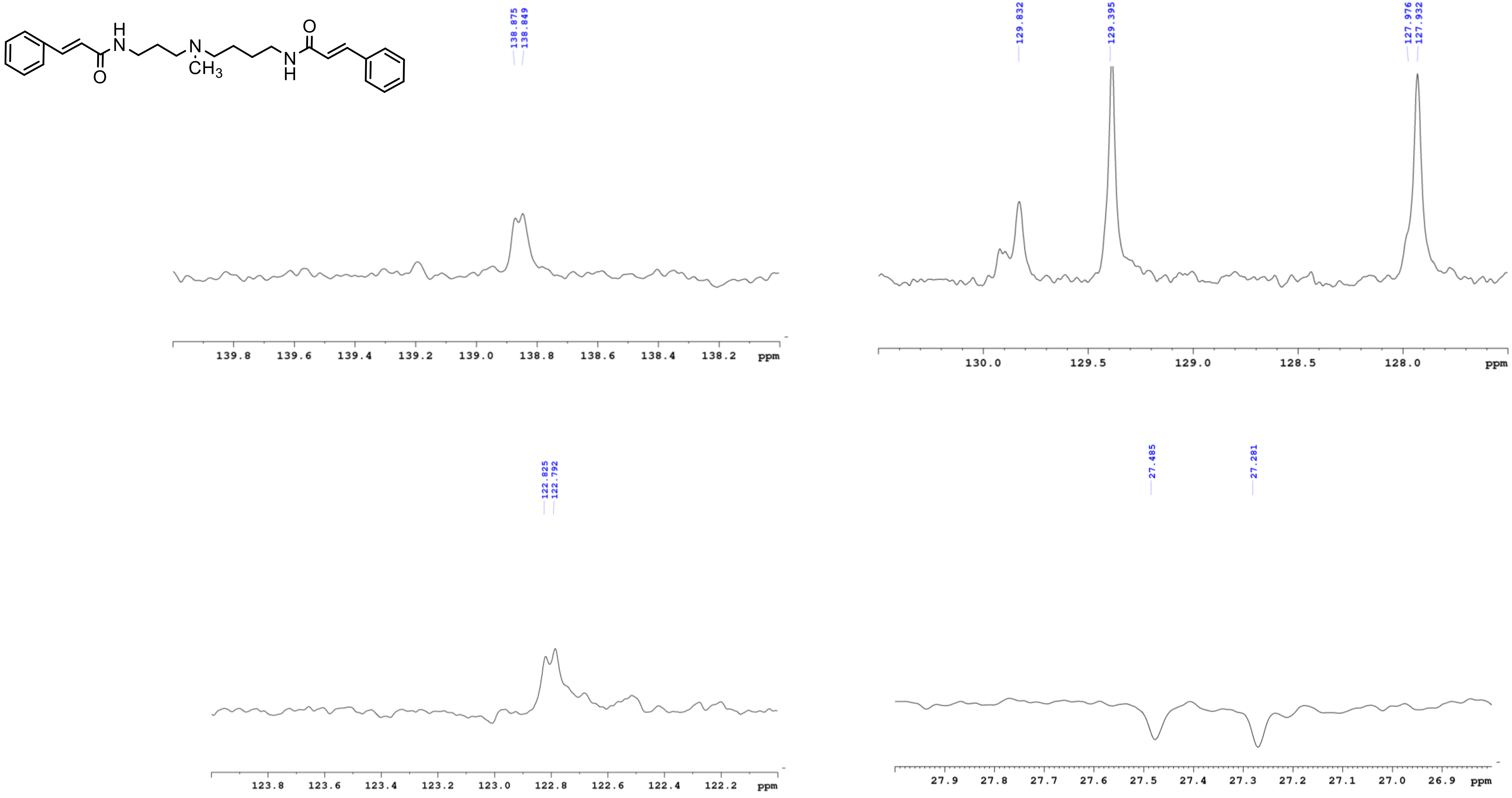

Figure S37: Expanded regions of the DEPT-135º spectrum (125 MHz, DMSO d_6, TMS) of 5-*N*-methylmaytenine.

Table S4: NMR chemical shifts of 5-*N*-methylmaitenine. The experiments were realized in DMSO d_6 at magnetic field strength of 11.7 T; 500 MHz for ^{1}H and 125 MHz for ^{13}C NMR;

s: singlet; d: doublet; t: triplet; m: multiplet.

Position	^{1}H δ$_H$, ppm (multiplicity; number of H; J, Hz)	COSY, ppm	^{13}C δ$_C$, ppm	HMBC, ppm
4´ and 4´´	-	-	135.43	-
1´ or 1´´	-	-	165.27	-
1´´ or 1´	-	-	165.32	-
5´ and 5´´ or 9´ and 9´´	7.56 – 7.54 (m; 2H)	H-3’; H-7’; H-3”; H-7”	127.98	C-3’; C-3”; C-7’; C-7”; C-9’; C-9” or C-5’; C-5”
9´ and 9´´ or 5´ and 5´´	7.56 – 7.54 (m; 2H)	H-3’; H-3”; H-7’; H-7”	127.94	C-3’; C-3”; C-7’; C-7”; C-5’; C-5” or C-9’; C-9”
6´, 6´´, 8´and 8´´	7.56 – 7.54 (m; 4H)	H-3’; H-3”; H-7’; H-7”	129.39	C-3’; C-3”; C-7’; C-7”
7´ and 7´´	7.40 (m; 2H)	H-5’; H-5” H-6’; H-6” H-9’; H-9”	129.83	C-5’; C-5”; C-9’; C-9”
3´or 3´´	7.42 (m; 1H)	H-2’; H-2” H-5’; H-5” H-6’; H-6” H-9’; H-9”	138.85	C-1’; C-1”; C-2’; C-2”; C-4’; C-4”; C-5’; C-5”; C-9’; C-9”
3´´or 3´	7.38 (m; 1H)	H-2’; H-2” H-5’; H-5” H-6’; H-6” H-9’; H-9”	138.88	C-1’; C-1”; C-2’; C-2”; C-4’; C-4”; C-5’; C-5”; C-9’; C-9”
2´or 2´´	6.63 (d; 1H; 16 Hz)	H-3’; H-3”	122.79	C-1’; C-1”; C-4’; C-4”
2´´ or 2´	6.60 (d; 1H; 16 Hz)	H-3’; H-3”	122.83	C-1’; C-1”; C-4’; C-4”
1 and 10 N-H	8.12 (t; 5.5 Hz)	H-2; H-9	-	C-1’; C-1”
2	3.20 (m; 2H)	H-3; -NH	37.58	C-1’; C-1”; C-3; C-4
9	3.18 (m; 2H)	H-8; -NH	39.07	C-1’; C-1”; C-8
4	2.35 (m; 2H)	H-3	55.26	C-2; C-3; C-5; C-6
6	2.32 (m; 2H)	H-7	57.12	C-4; C-8
5 N-CH_3	2.16 (s; 3H)	-	42.09	C-4; C-6
3	1.61 (m; 2H)	H-2; H-4	27.28	C-4; C-2
8	1.47 (m; 2H)	H-9	24.53	C-7
7	1.45 (m; 2H)	H-6	27.48	C-8

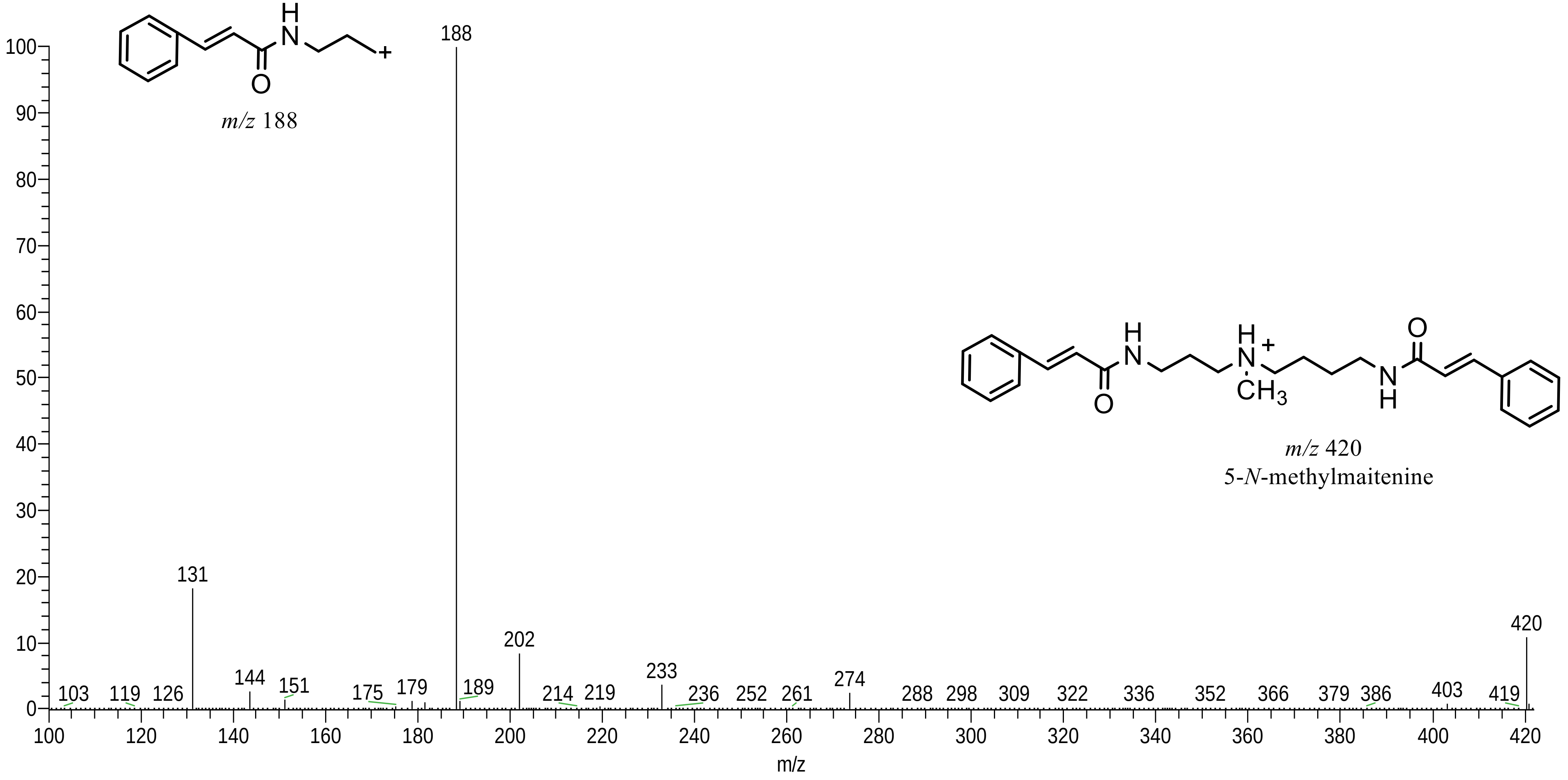

Figure S38: Mass spectrum (MS/MS) of 5-*N*-methylmaytenine with chemical structure and identification of the main fragment ions.

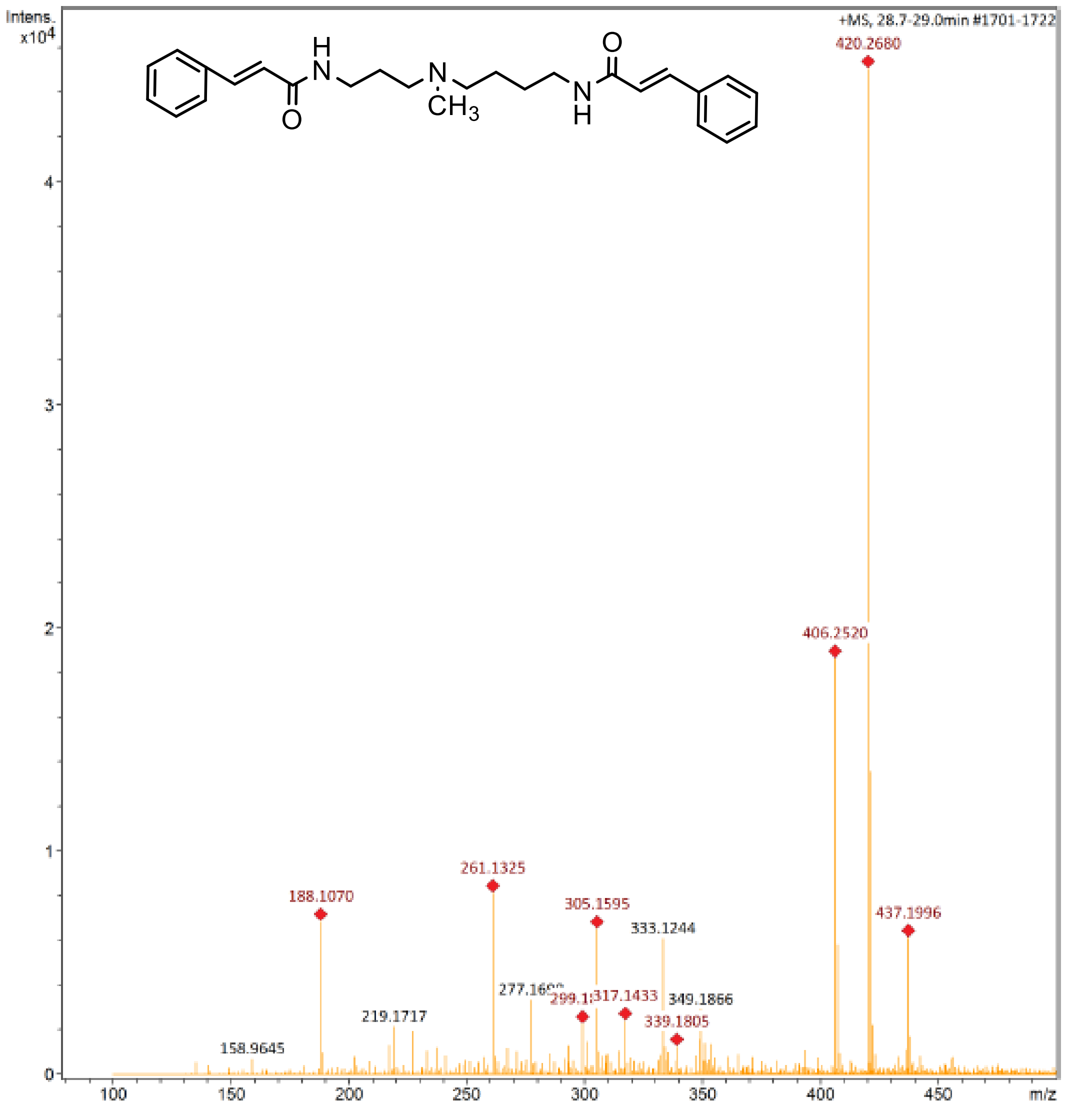

Figure S39: Hight resolution mass-spectrum of 5-*N*-methylmaytenine with chemical structure.

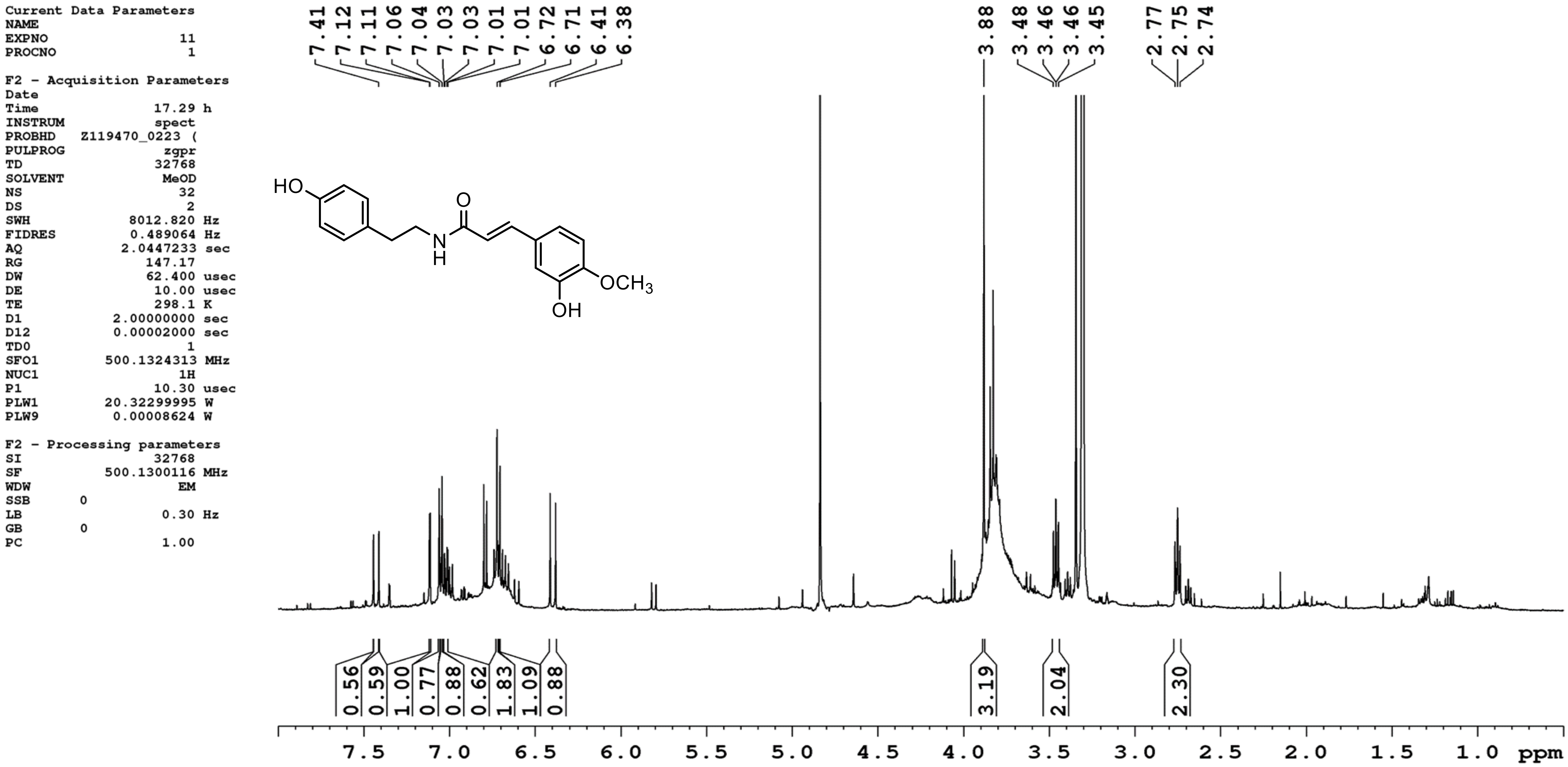

Figure S40: ^{1}H NMR spectrum (500 MHz, CD_3OD d_6, TMS) of *N-trans*-feruloyltyramine.

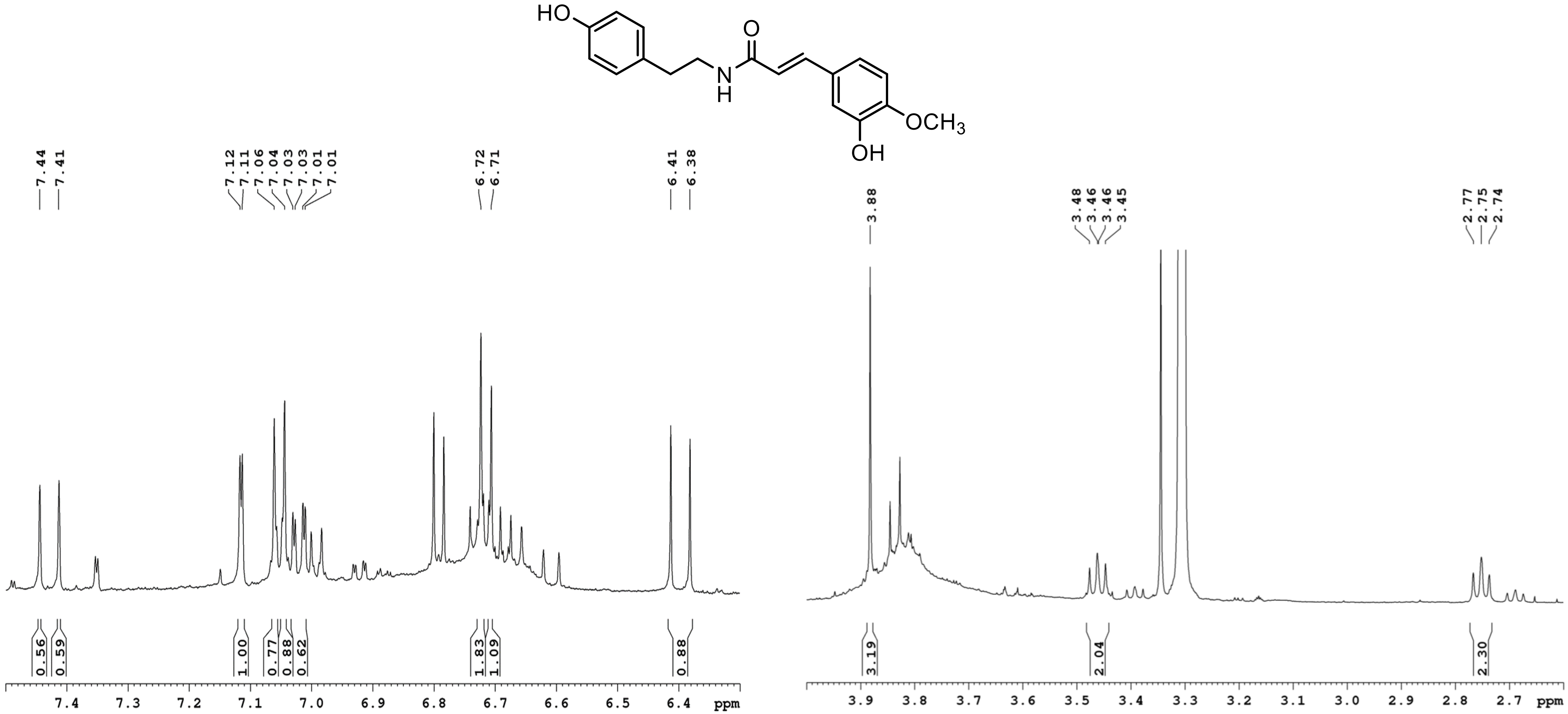

Figure S41: Expanded low-field region of the ^{1}H NMR spectrum (500 MHz, CD_3OD d_6, TMS) *N-trans*-feruloyltyramine.

```
Current Data Parameters
NAME
EXPNO                200
PROCNO                 1

F2 - Acquisition Parameters
Date
Time                1.50 h
INSTRUM            spect
PROBHD   Z119470_0223 (
PULPROG  hsqcetgpsisp2.2
TD                  2048
SOLVENT             MeOD
NS                    64
DS                    16
SWH            10000.000 Hz
FIDRES          4.882813 Hz
AQ             0.1024000 sec
RG                187.25
DW                50.000 usec
DE                 10.00 usec
TE                 298.2 K
CNST2        145.0000000
CNST17        -0.5000000
D0            0.00000300 sec
D1            1.00000000 sec
D4            0.00172414 sec
D11           0.03000000 sec
D16           0.00020000 sec
D24           0.00089000 sec
IN0           0.00001850 sec
TDav                   1
SFO1         500.1330883 MHz
NUC1                  1H
P1                  9.40 usec
P2                 18.80 usec
P28              1000.00 usec
PLW1         20.32299995 W
SFO2         125.7703643 MHz
NUC2                 13C
CPDPRG[2            garp
P3                 10.00 usec
P14               500.00 usec
P24              2000.00 usec
PCPD2              70.00 usec
PLW0       0 W
PLW2         88.00000000 W
PLW12         1.78668594 W
SPNAM[3] Crp60,0.5,20.1
SPOAL3             0.500
SPOFFS3    0 Hz
SPW3         13.44499969 W
SPNAM[7]     Crp60comp.4
SPOAL7             0.500
SPOFFS7    0 Hz
SPW7         13.44499969 W
GPNAM[1]      SMSQ10.100
GPZ1               80.00 %
GPNAM[2]      SMSQ10.100
GPZ2               20.10 %
GPNAM[3]      SMSQ10.100
GPZ3               11.00 %
GPNAM[4]      SMSQ10.100
GPZ4               -5.00 %
P16              1000.00 usec
P19               600.00 usec

F1 - Acquisition parameters
TD                   256
SFO1            125.7704 MHz
FIDRES        211.148651 Hz
SW               214.892 ppm
FnMODE     Echo-Antiecho

F2 - Processing parameters
SI                  4096
SF           500.1300105 MHz
WDW                QSINE
SSB                    2
LB         0 Hz
GB         0
PC                  1.40

F1 - Processing parameters
SI                  1024
MC2       echo-antiecho
SF           125.7576744 MHz
WDW                QSINE
SSB                    2
LB         0 Hz
GB         0
```

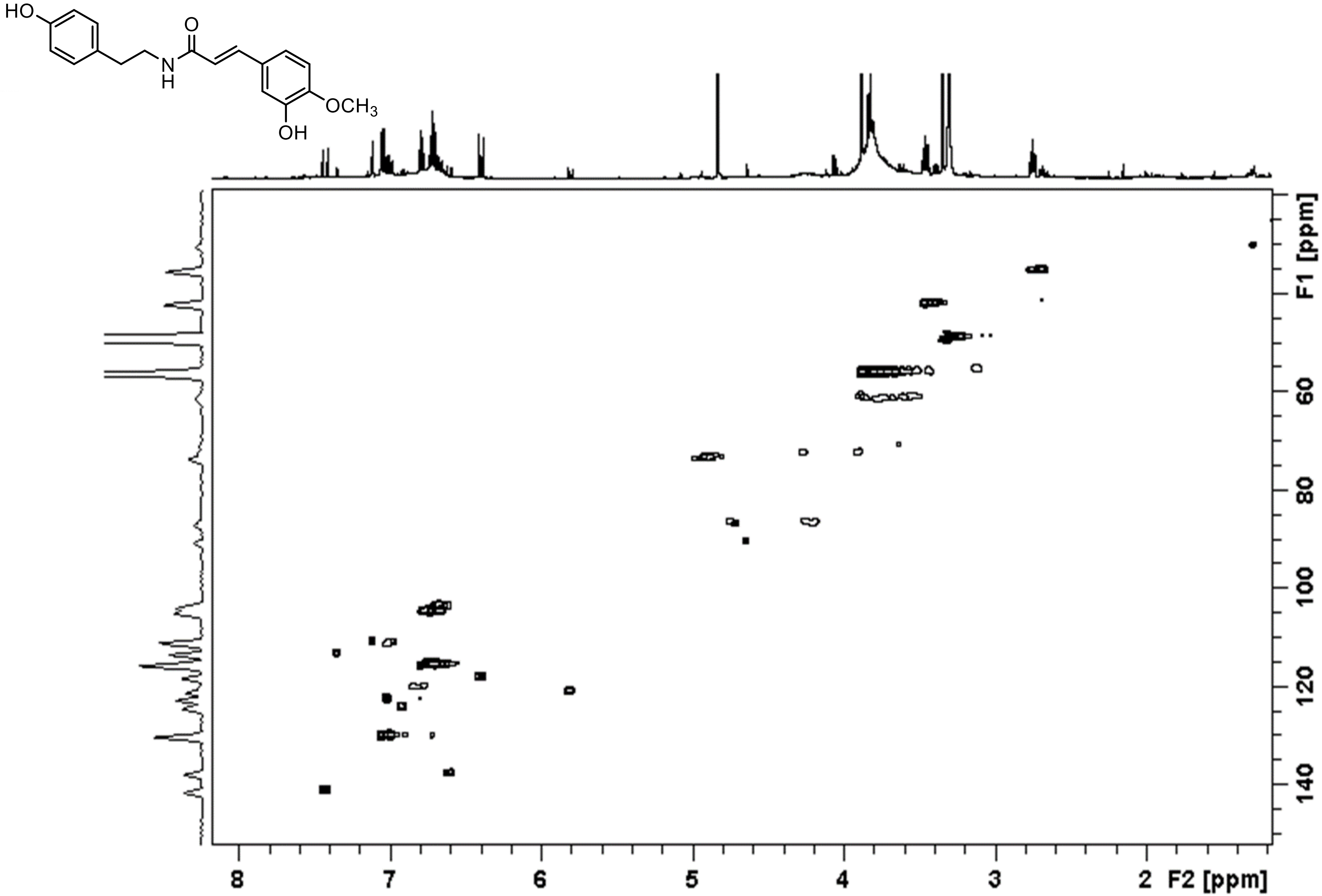

Figure S42: HSQC spectrum (125 MHz, CD_3OD, TMS) *N-trans*-feruloyltyramine.

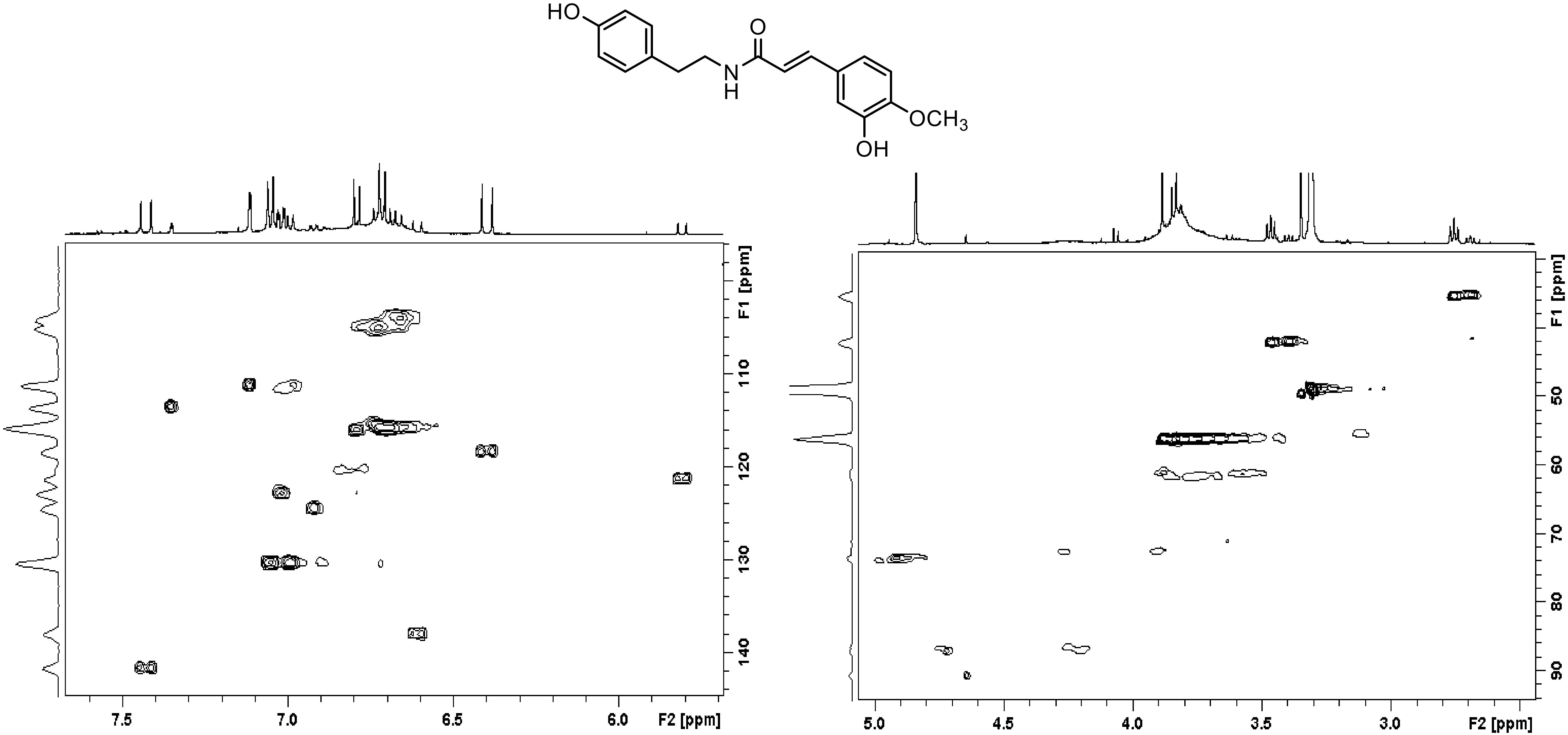

Figure S43: Expanded regions of the HSQC spectrum (125 MHz, CD_3OD, TMS) *N-trans*-feruloyltyramine.

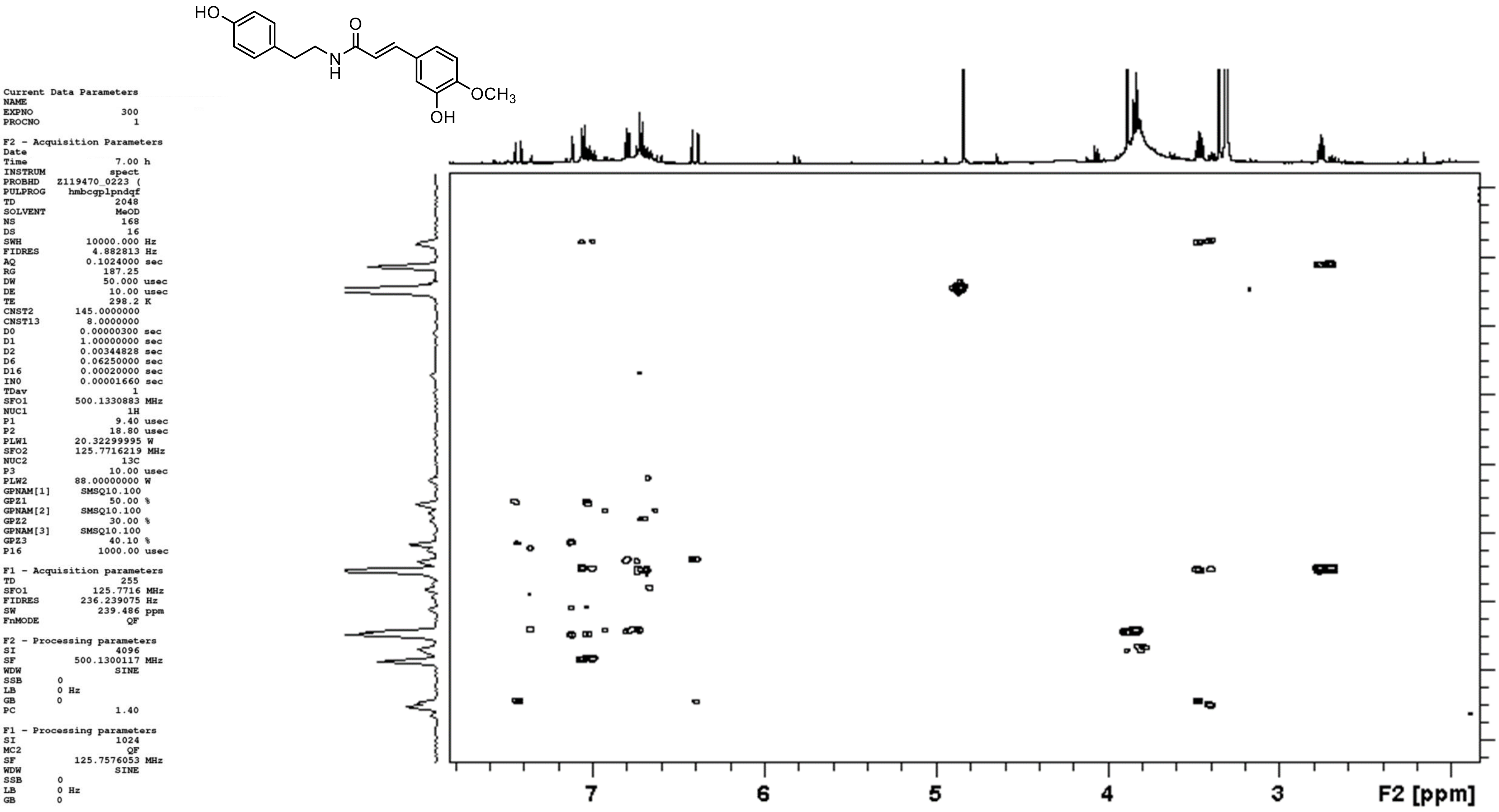

Figure S44: HMBC spectrum (125 MHz, CD_3OD, TMS) of *N-trans*-feruloyltyramine.

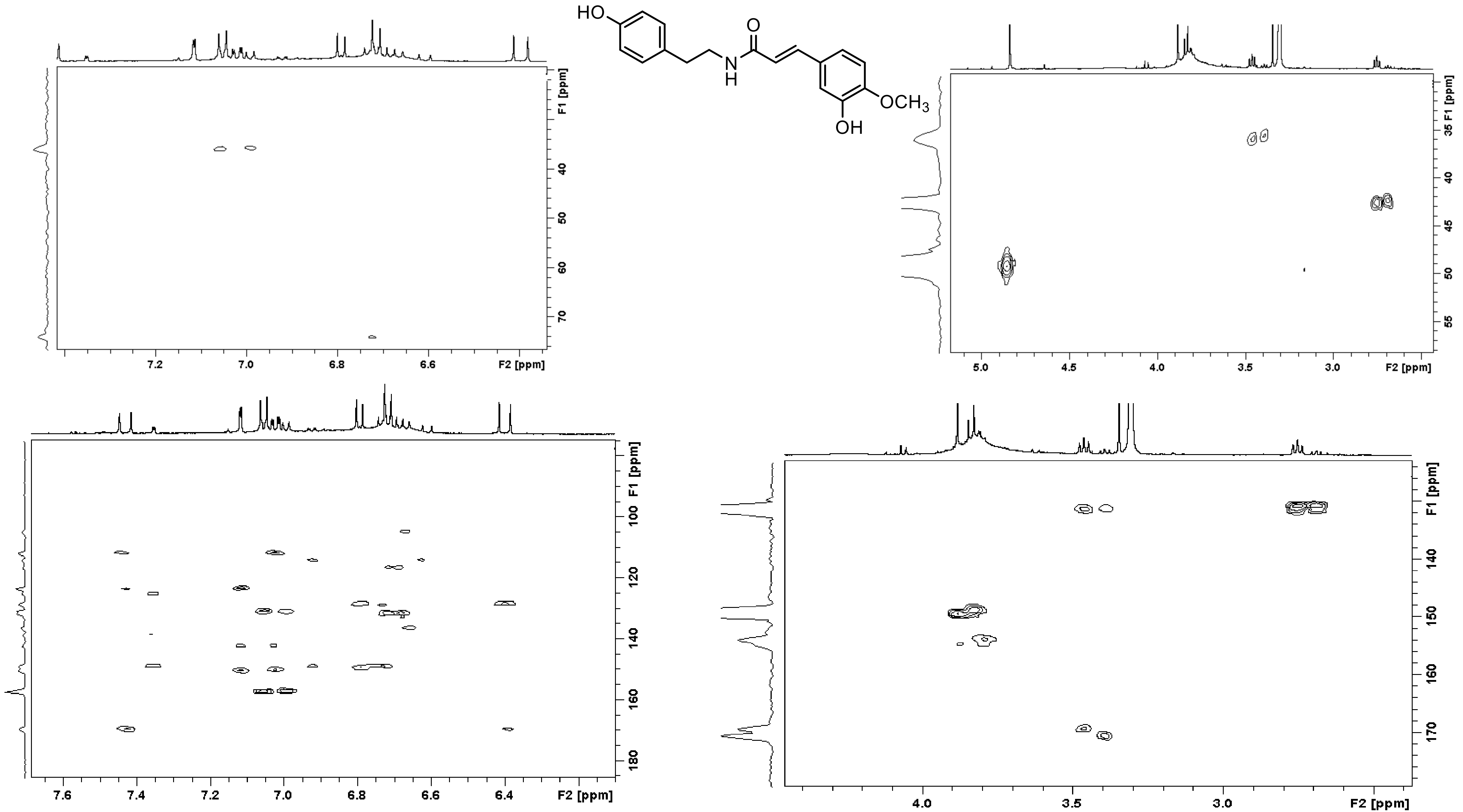

Figure S45: Expanded regions of the HMBC spectrum (125 MHz, CD_3OD, TMS) of *N-trans*-feruloyltyramine.

Table S5: NMR chemical shifts of *N-trans*-feruloyltyramine. The experiments were realized in CD_3OD at magnetic field strength of 11.7 T; 500 MHz for ^1H NMR.

s: singlet; m: multiplet; dd: doublet of doublets; ddd: doublet of doublets of doublets.

Position	^1H	^{13}C	
	δ_H, ppm (multiplicity; number of H; J, Hz)	δ_C (HSQC), ppm [a]	HMBC, ppm [a]
1	-	123.4	-
2	7.11 (d; 1H; 1.8 Hz)	111.1	C-1; C-7; C-4
3	-	148.8	-
4	-	150.2	-
4-OCH_3	3.88 (s; 3H)	56.0	C-4
5	6.70 (m; 1H)	115.7	C-3
6	7.03 (dd; 1H; 1.8 and 8.5 Hz)	122.7	C-2; C-7; C-4
7	7.44 (d; 1H; 16 Hz)	141.5	C-2; C-1; C-9
8	6.40 (d; 1H; 16 Hz)	118.4	C-9
9	-	169.3	-
1’	-	130.9	-
2’	7.06 (m; 1H)	130.2	C-7’; C-1’; C-4’
3’	6.72 (m; 2H)	115.7	C-1’; C-4’
4’	-	157.1	-
5’	6.72 (m; 2H)	115.7	C-1’; C-4’
6’	7.06 (m; 1H)	130.2	C-7’; C-1’; C-4’
7’	2.75 (m; 2H)	35.4	C-8’; C-1’
8’	3.46 (m; 2H)	42.1	C-7’; C-1’; C-9

[a] ^{13}C NMR signals were assigned using the HSQC and HMBC data.

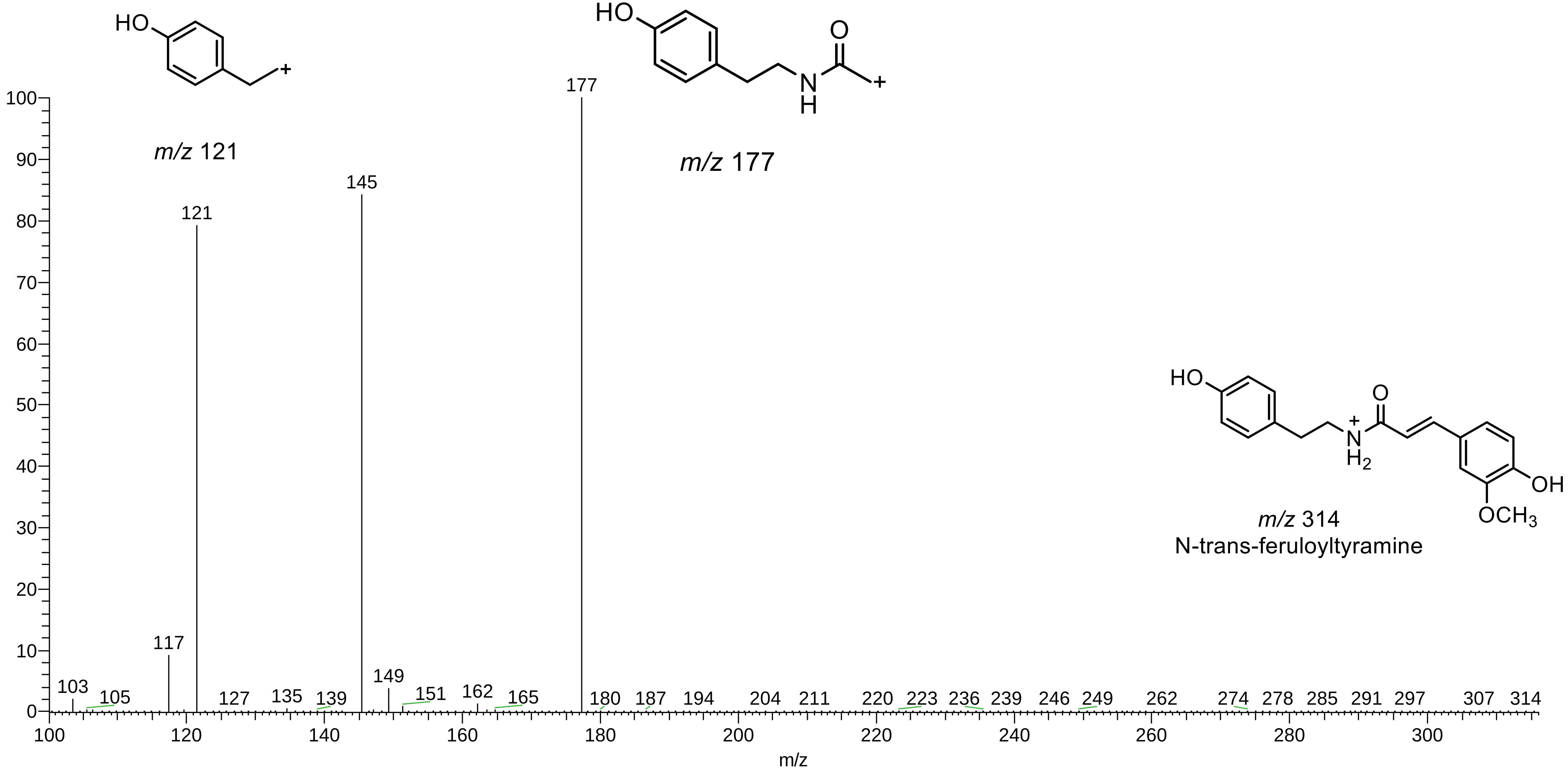

Figure S46: Mass spectrum (MS/MS) of *N-trans*-feruloyltyramine with chemical structure and identification of the main fragment ions.

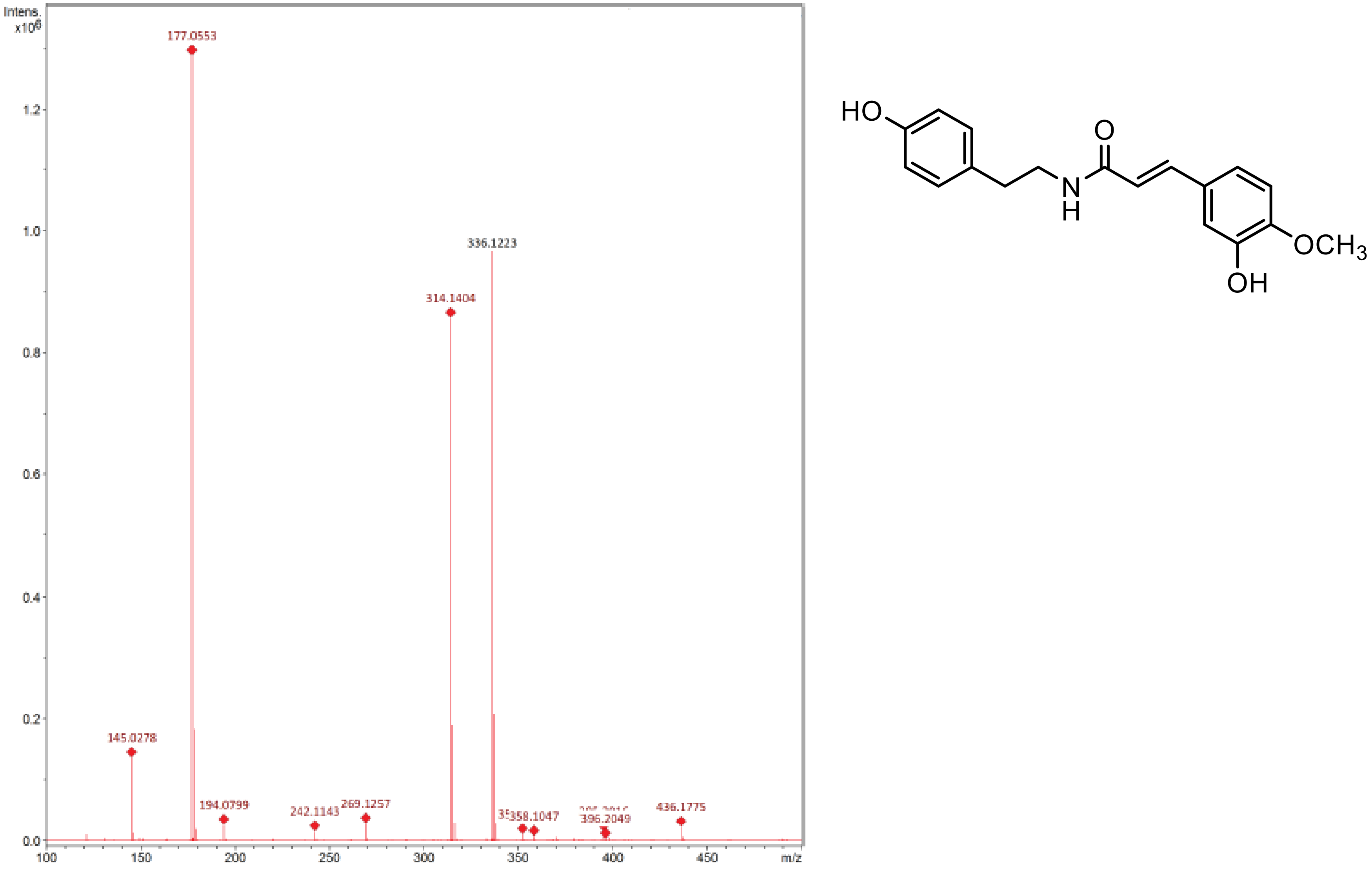

Figure S47: Hight resolution mass-spectrum of *N-trans*-feruloyltyramine with chemical structure.

Report S1
MD Simulation Report on AChE - Neostigmine Interactions

Simulation Details

Jobname: md_job_6H12_NEO-3_best_10ns_TIP4PEw
Entry title: Setup_6H12_NEO-3-best_mmgbsa

CPU #	Job Type	Ensemble	Temp. [K]	Sim. Time [ns]	# Atoms	# Waters	Charge
1	mdsim	NPT	300.0	10.005	62798	17952	0

Protein Information

Tot. Residues	Prot. Chain(s)	Res. in Chain(s)	# Atoms	# Heavy Atoms	Charge
562	'NoChainId'	ict_values([562]	8798	4471	-10

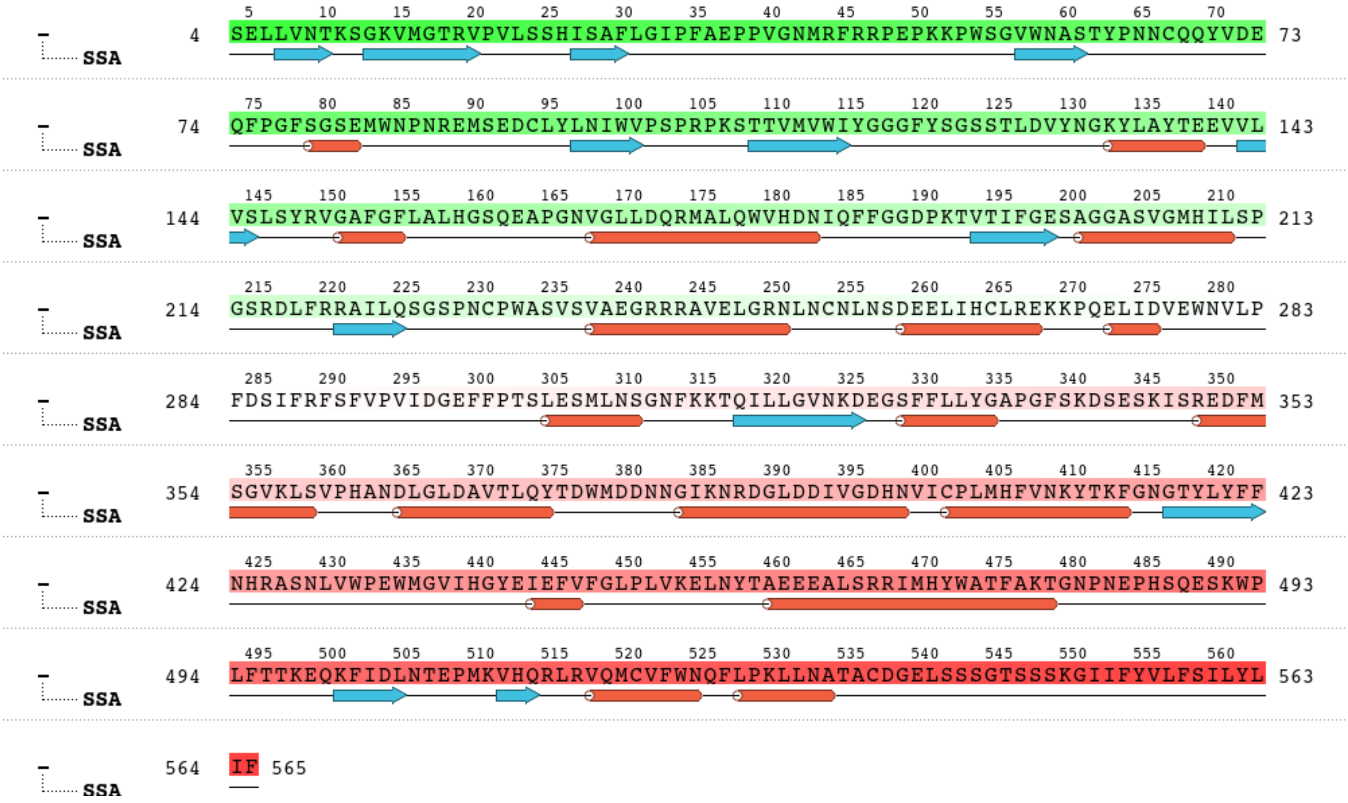

Ligand Information

SMILES	CN(C)C(=O)Oc(ccc1)cc1[N+](C)(C)C
PDB Name	'UNK'
Num. of Atoms	35 (total) 16 (heavy)
Atomic Mass	223.297 au
Charge	+1
Mol. Formula	C12H19N2O2
Num. of Fragments	5
Num. of Rot. Bonds	4

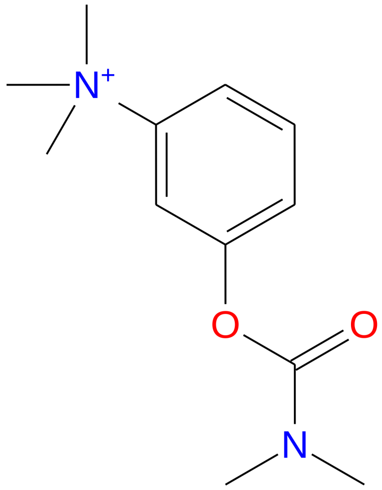

Counter Ion/Salt Information

Type	Num.	Concentration [mM]	Total Charge
Na	59	59.755	+59
Cl	50	50.640	-50

Protein-Ligand RMSD

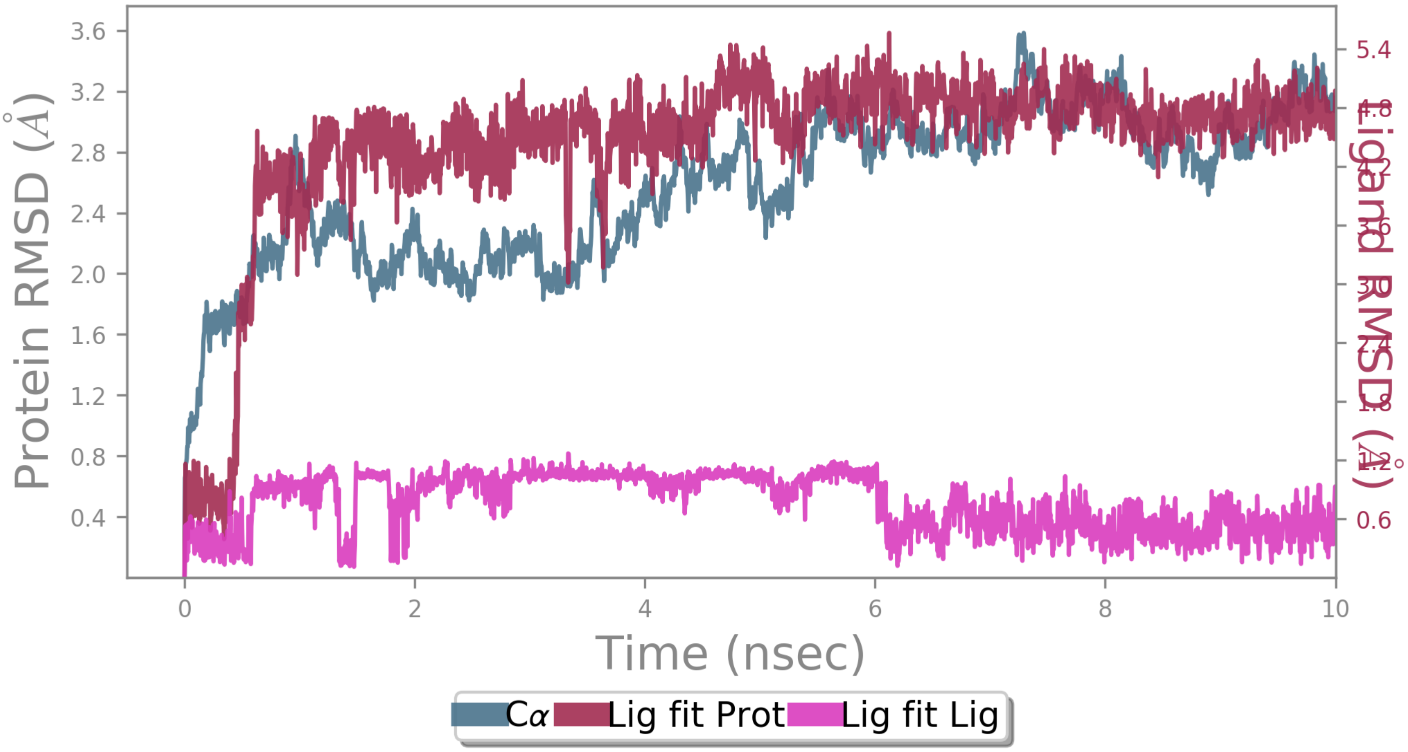

The Root Mean Square Deviation (RMSD) is used to measure the average change in displacement of a selection of atoms for a particular frame with respect to a reference frame. It is calculated for all frames in the trajectory. The RMSD for frame *x* is:

$$RMSD_x = \sqrt{\frac{1}{N}\sum_{i=1}^{N}(r'_i(t_x)) - r_i(t_{ref}))^2}$$

where *N* is the number of atoms in the atom selection; t_{ref} is the reference time, (typically the first frame is used as the reference and it is regarded as time *t*=0); and *r'* is the position of the selected atoms in frame *x* after superimposing on the reference frame, where frame *x* is recorded at time t_x. The procedure is repeated for every frame in the simulation trajectory.

Protein RMSD: The above plot shows the RMSD evolution of a protein (left *Y*-axis). All protein frames are first aligned on the reference frame backbone, and then the RMSD is calculated based on the atom selection. Monitoring the RMSD of the protein can give insights into its structural conformation throughout the simulation. RMSD analysis can indicate if the simulation has equilibrated — its fluctuations towards the end of the simulation are around some thermal average structure. Changes of the order of 1-3 Å are perfectly acceptable for small, globular proteins. Changes much larger than that, however, indicate that the protein is undergoing a large conformational change during the simulation. It is also important that your simulation converges — the RMSD values stabilize around a fixed value. If the RMSD of the protein is still increasing or decreasing on average at the end of the simulation, then your system has not equilibrated, and your simulation may not be long enough for rigorous analysis.

Ligand RMSD: Ligand RMSD (right *Y*-axis) indicates how stable the ligand is with respect to the protein and its binding pocket. In the above plot, 'Lig fit Prot' shows the RMSD of a ligand when the protein-ligand complex is first aligned on the protein backbone of the reference and then the RMSD of the ligand heavy atoms is measured. If the values observed are significantly larger than the RMSD of the protein, then it is likely that the ligand has diffused away from its initial binding site. 'Lig fit Lig' shows the RMSD of a ligand that is aligned and measured just on its reference conformation. This RMSD value measures the internal fluctuations of the ligand atoms.

Protein RMSF

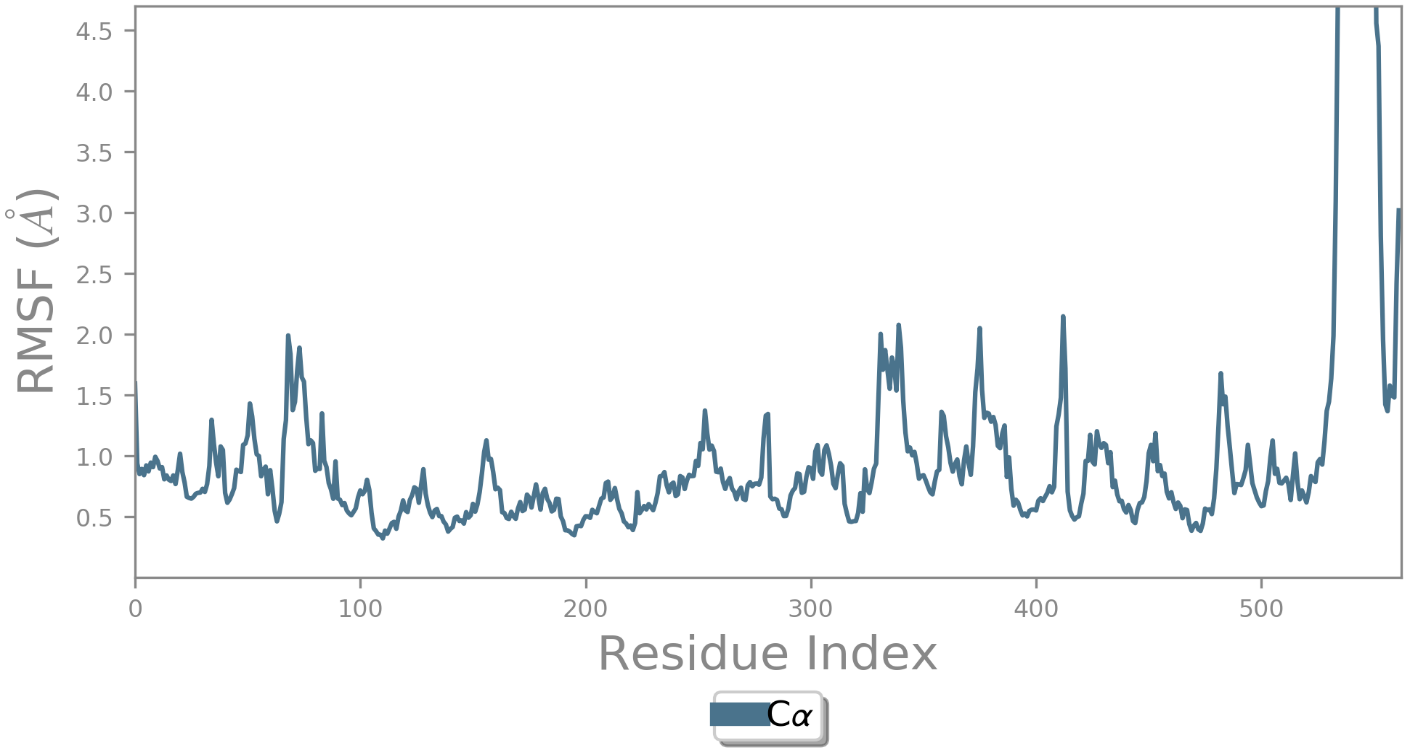

The Root Mean Square Fluctuation (RMSF) is useful for characterizing local changes along the protein chain. The RMSF for residue *i* is:

$$RMSF_i = \sqrt{\frac{1}{T}\sum_{t=1}^{T} < (r_i'(t)) - r_i(t_{ref}))^2 >}$$

where T is the trajectory time over which the RMSF is calculated, t_{ref} is the reference time, r_i is the position of residue *i*; *r'* is the position of atoms in residue *i* after superposition on the reference, and the angle brackets indicate that the average of the square distance is taken over the selection of atoms in the residue.

On this plot, peaks indicate areas of the protein that fluctuate the most during the simulation. Typically you will observe that the tails (*N*- and *C*-terminal) fluctuate more than any other part of the protein. Secondary structure elements like alpha helices and beta strands are usually more rigid than the unstructured part of the protein, and thus fluctuate less than the loop regions.

Protein Secondary Structure

% Helix	% Strand	% Total SSE
29.17	12.85	42.02

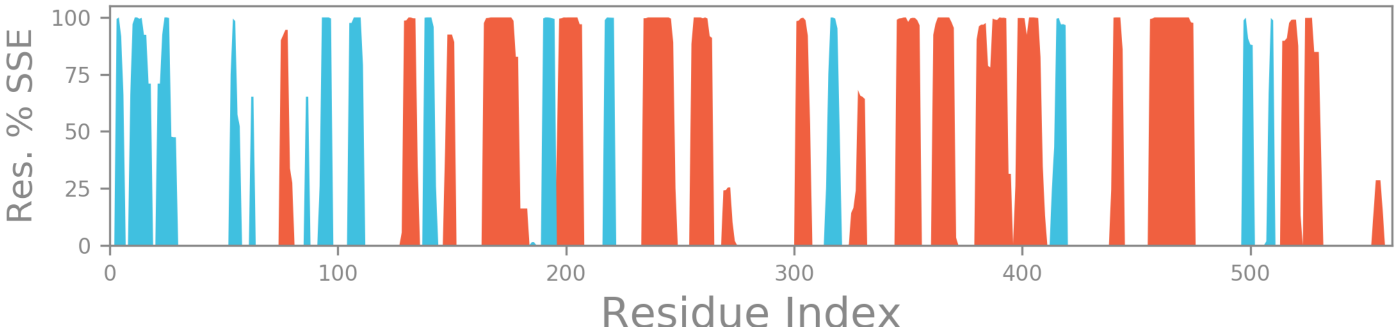

Protein secondary structure elements (SSE) like alpha-helices and beta-strands are monitored throughout the simulation. The plot above reports SSE distribution by residue index throughout the protein structure. The plot below summarizes the SSE composition for each trajectory frame over the course of the simulation, and the plot at the bottom monitors each residue and its SSE assignment over time.

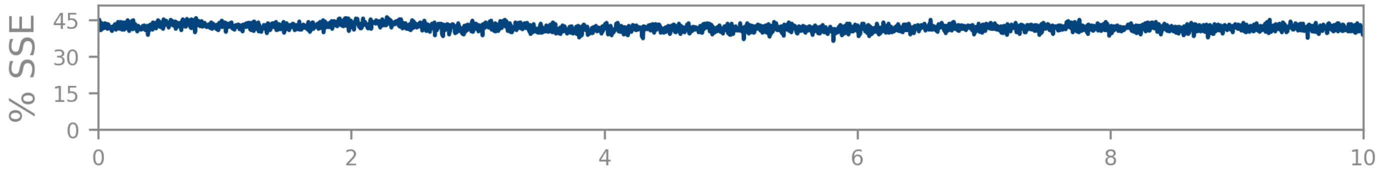

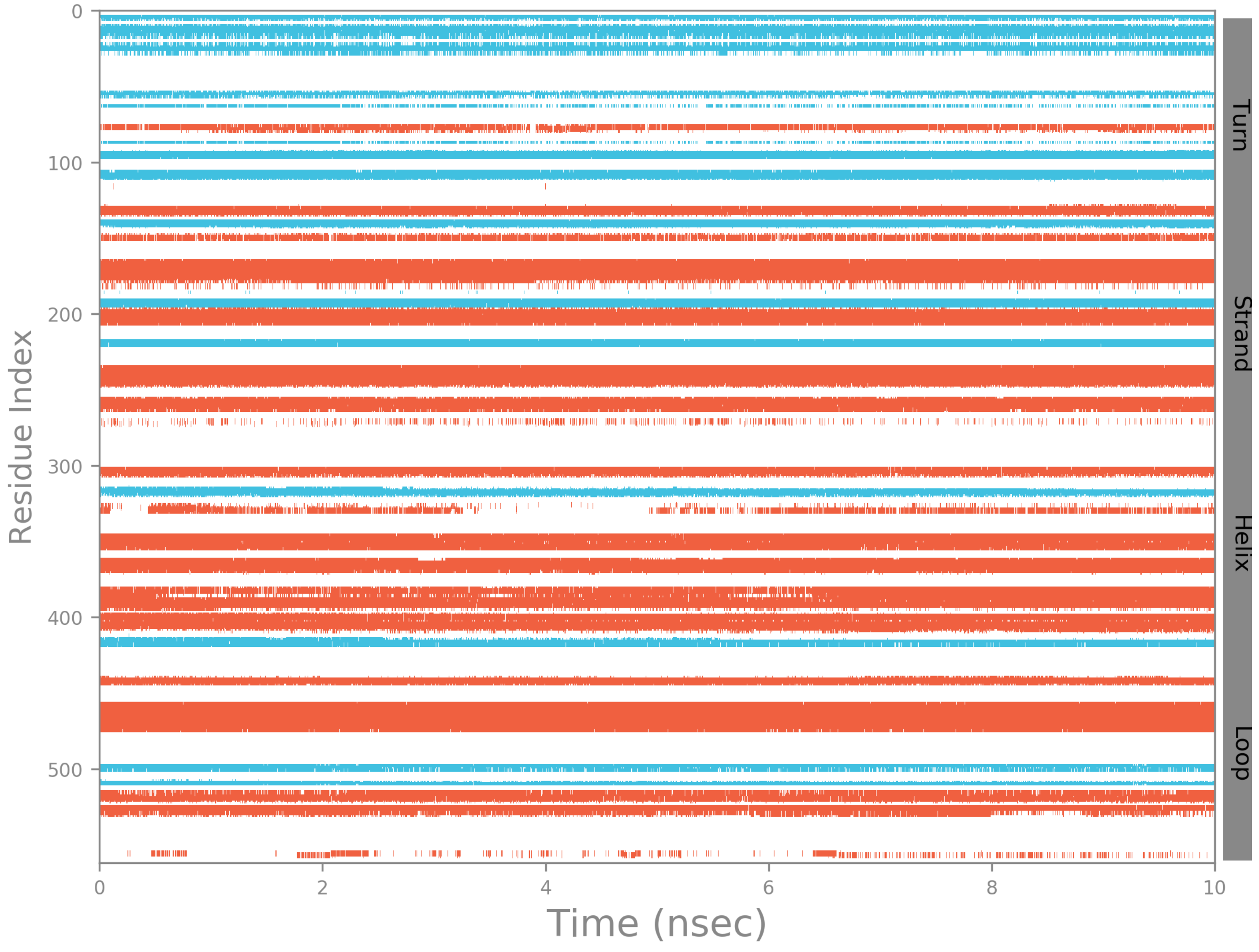

Ligand RMSF

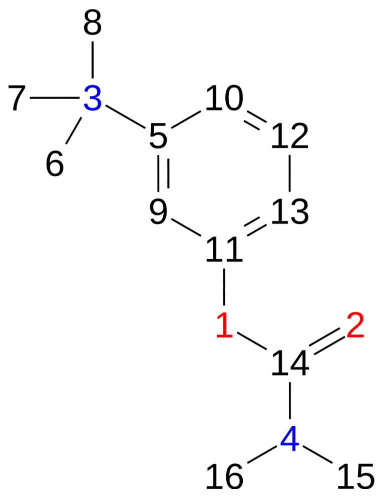

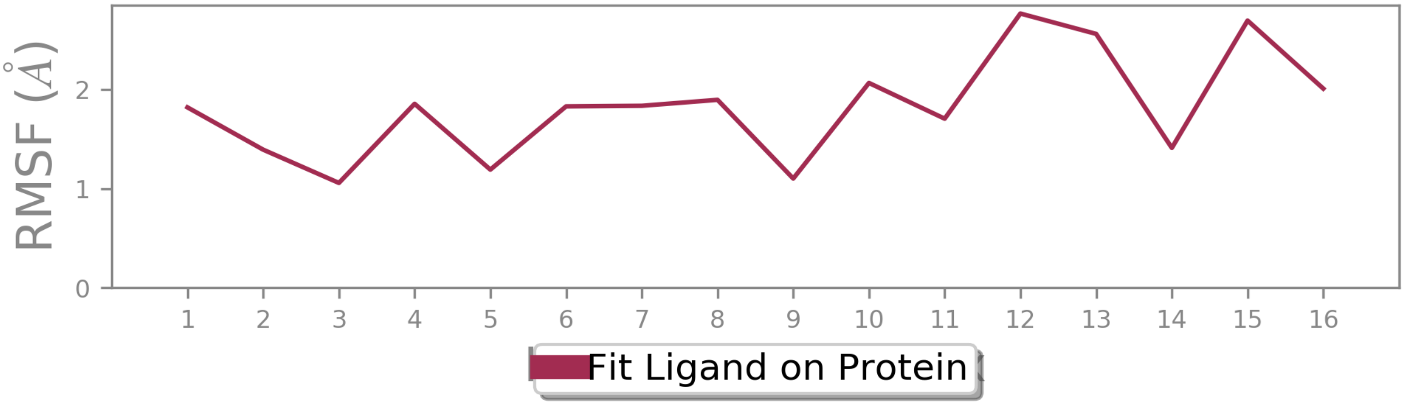

The Ligand Root Mean Square Fluctuation (L-RMSF) is useful for characterizing changes in the ligand atom positions. The RMSF for atom *i* is:

$$RMSF_i = \sqrt{\frac{1}{T}\sum_{t=1}^{T}(r_i'(t)) - r_i(t_{ref}))^2}$$

where *T* is the trajectory time over which the RMSF is calculated, t_{ref} is the reference time (usually for the first frame, and is regarded as the *zero* of time); *r* is the position of atom *i* in the reference at time t_{ref}, and *r'* is the position of atom *i* at time *t* after superposition on the reference frame.

Ligand RMSF shows the ligand's fluctuations broken down by atom, corresponding to the 2D structure in the top panel. The ligand RMSF may give you insights on how ligand fragments interact with the protein and their entropic role in the binding event. In the bottom panel, the 'Fit Ligand on Protein' line shows the ligand fluctuations, with respect to the protein. The protein-ligand complex is first aligned on the protein backbone and then the ligand RMSF is measured on the ligand heavy atoms.

Protein-Ligand Contacts

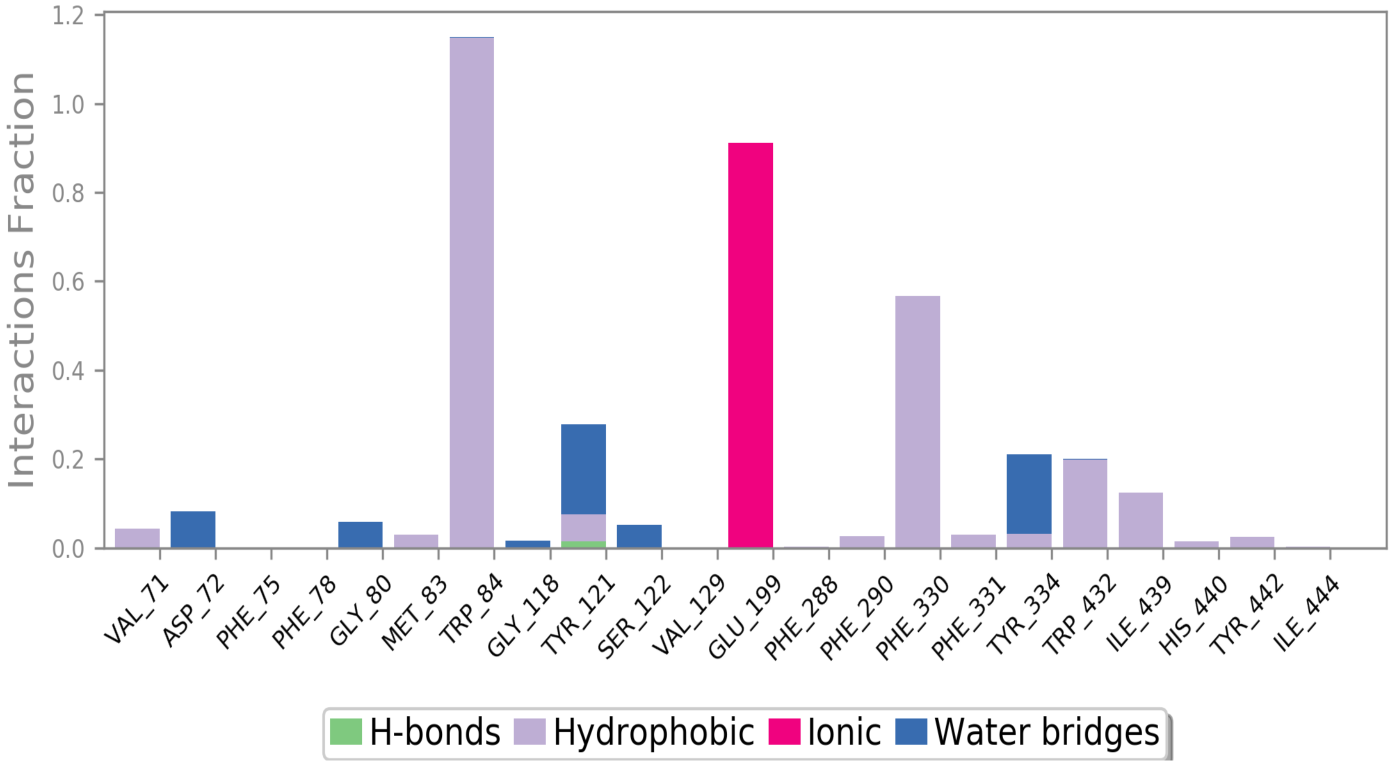

Protein interactions with the ligand can be monitored throughout the simulation. These interactions can be categorized by type and summarized, as shown in the plot above. Protein-ligand interactions (or 'contacts') are categorized into four types: Hydrogen Bonds, Hydrophobic, Ionic and Water Bridges. Each interaction type contains more specific subtypes, which can be explored through the 'Simulation Interactions Diagram' panel. The stacked bar charts are normalized over the course of the trajectory: for example, a value of 0.7 suggests that 70% of the simulation time the specific interaction is maintained. Values over 1.0 are possible as some protein residue may make multiple contacts of same subtype with the ligand.

Hydrogen Bonds: (H-bonds) play a significant role in ligand binding. Consideration of hydrogen-bonding properties in drug design is important because of their strong influence on drug specificity, metabolization and adsorption. Hydrogen bonds between a protein and a ligand can be further broken down into four subtypes: backbone acceptor; backbone donor; side-chain acceptor; side-chain donor.
The current geometric criteria for protein-ligand H-bond is: distance of 2.5 Å between the donor and acceptor atoms (D—H···A); a donor angle of ≥120° between the donor-hydrogen-acceptor atoms (D—H···A); and an acceptor angle of ≥90° between the hydrogen-acceptor-bonded_atom atoms (H···A—X).

Hydrophobic contacts: fall into three subtypes: π-Cation; π-π; and Other, non-specific interactions. Generally these type of interactions involve a hydrophobic amino acid and an aromatic or aliphatic group on the ligand, but we have extended this category to also include π-Cation interactions.
The current geometric criteria for hydrophobic interactions is as follows: π-Cation — Aromatic and charged groups within 4.5 Å; π-π — Two aromatic groups stacked face-to-face or face-to-edge; Other — A non-specific hydrophobic sidechain within 3.6 Å of a ligand's aromatic or aliphatic carbons.

Ionic interactions: or polar interactions, are between two oppositely charged atoms that are within 3.7 Å of each other and do not involve a hydrogen bond. We also monitor Protein-Metal-Ligand interactions, which are defined by a metal ion coordinated within 3.4 Å of protein's and ligand's heavy atoms (except carbon). All ionic interactions are broken down into two subtypes: those mediated by a protein backbone or side chains.

Water Bridges: are hydrogen-bonded protein-ligand interactions mediated by a water molecule. The hydrogen-bond geometry is slightly relaxed from the standard H-bond definition.
The current geometric criteria for a protein-water or water-ligand H-bond are: a distance of 2.8 Å between the donor and acceptor atoms (D—H···A); a donor angle of ≥110° between the donor-hydrogen-acceptor atoms (D—H···A); and an acceptor angle of ≥90° between the hydrogen-acceptor-bonded_atom atoms (H···A—X).

Protein-Ligand Contacts (cont.)

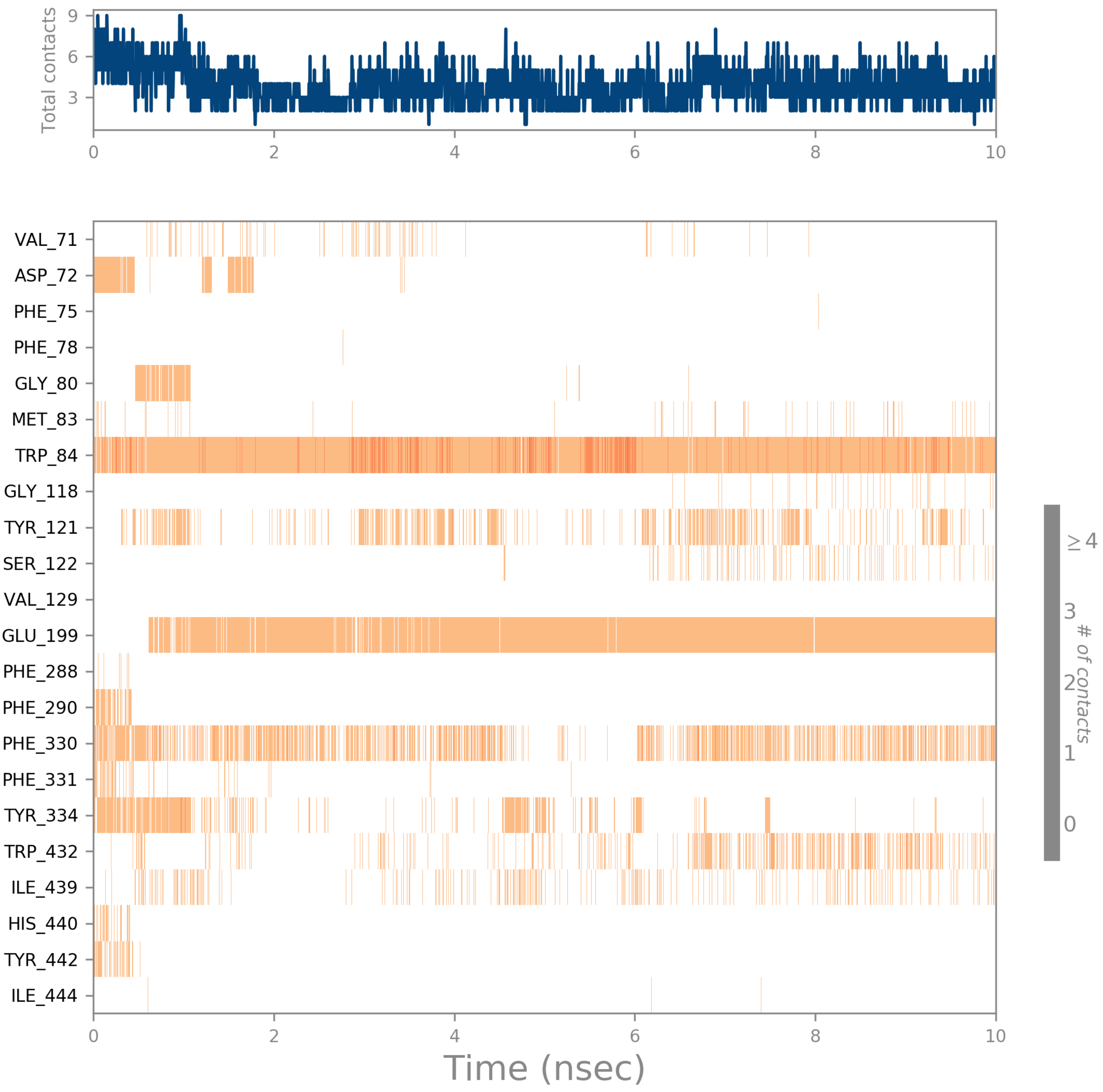

A timeline representation of the interactions and contacts (**H-bonds**, **Hydrophobic**, **Ionic**, **Water bridges**) summarized in the previous page. The top panel shows the total number of specific contacts the protein makes with the ligand over the course of the trajectory. The bottom panel shows which residues interact with the ligand in each trajectory frame. Some residues make more than one specific contact with the ligand, which is represented by a darker shade of orange, according to the scale to the right of the plot.

Ligand-Protein Contacts

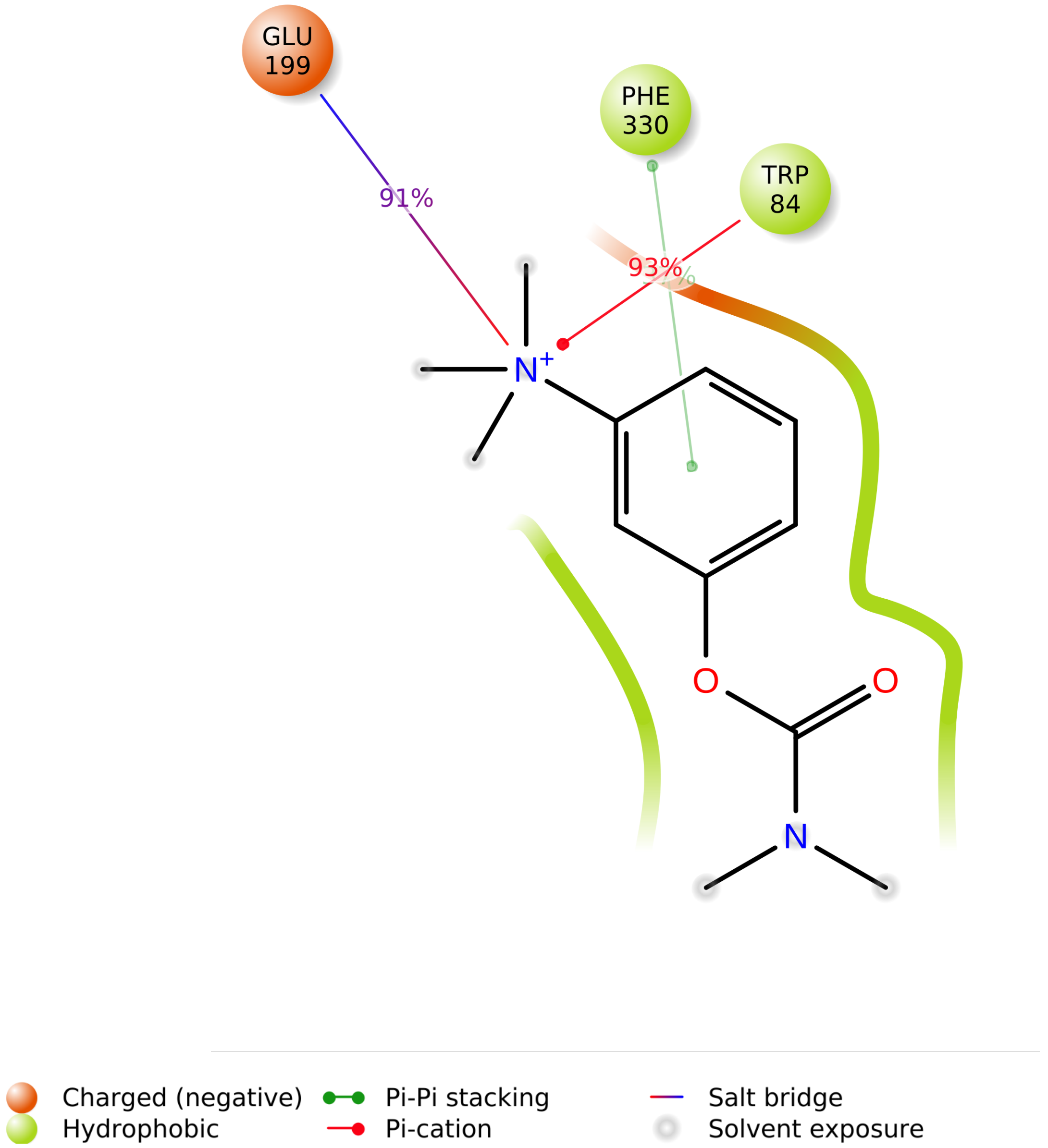

A schematic of detailed ligand atom interactions with the protein residues. Interactions that occur more than **30.0%** of the simulation time in the selected trajectory (0.00 through 10.00 nsec), are shown.
Note: it is possible to have interactions with >100% as some residues may have multiple interactions of a single type with the same ligand atom. For example, the ARG side chain has four H-bond donors that can all hydrogen-bond to a single H-bond acceptor.

Ligand Torsion Profile

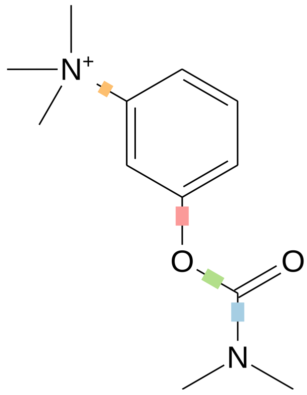

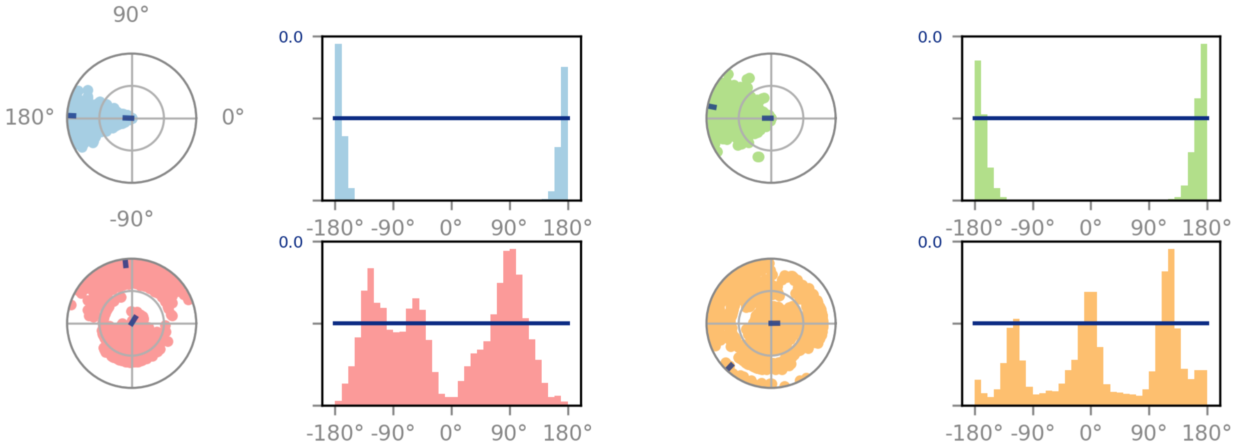

The ligand torsions plot summarizes the conformational evolution of every rotatable bond (RB) in the ligand throughout the simulation trajectory (0.00 through 10.00 nsec). The top panel shows the 2d schematic of a ligand with color-coded rotatable bonds. Each rotatable bond torsion is accompanied by a dial plot and bar plots of the same color.

Dial (or radial) plots describe the conformation of the torsion throughout the course of the simulation. The beginning of the simulation is in the center of the radial plot and the time evolution is plotted radially outwards.

The bar plots summarize the data on the dial plots, by showing the probability density of the torsion. If torsional potential information is available, the plot also shows the potential of the rotatable bond (by summing the potential of the related torsions). The values of the potential are on the left *Y*-axis of the chart, and are expressed in *kcal/mol*. Looking at the histogram and torsion potential relationships may give insights into the conformational strain the ligand undergoes to maintain a protein-bound conformation.

Ligand Properties

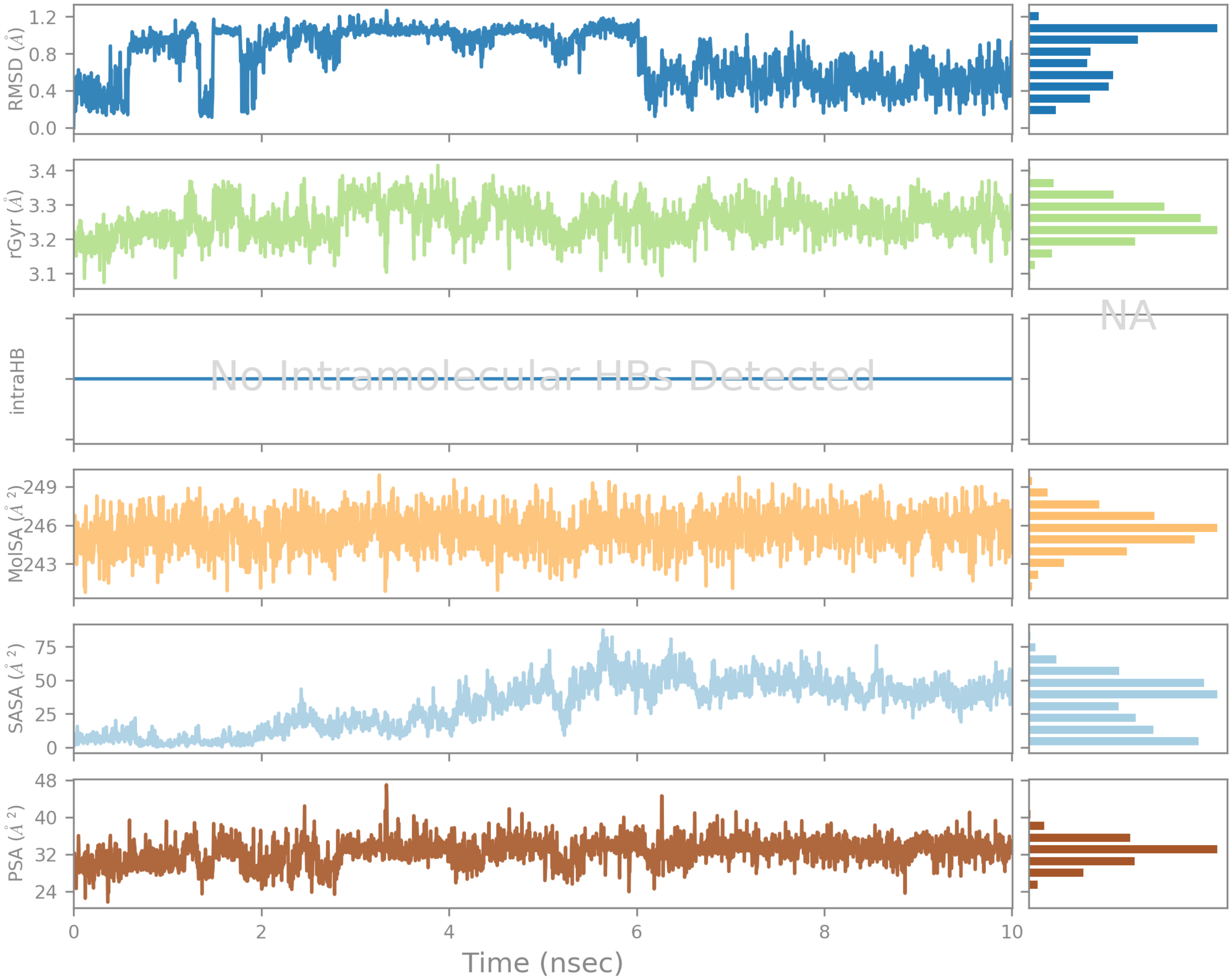

Ligand RMSD: Root mean square deviation of a ligand with respect to the reference conformation (typically the first frame is used as the reference and it is regarded as time *t*=0).

Radius of Gyration (rGyr): Measures the 'extendedness' of a ligand, and is equivalent to its principal moment of inertia.

Intramolecular Hydrogen Bonds (intraHB): Number of internal hydrogen bonds (HB) within a ligand molecule.

Molecular Surface Area (MolSA): Molecular surface calculation with 1.4 Å probe radius. This value is equivalent to a van der Waals surface area.

Solvent Accessible Surface Area (SASA): Surface area of a molecule accessible by a water molecule.

Polar Surface Area (PSA): Solvent accessible surface area in a molecule contributed only by oxygen and nitrogen atoms.

Report S2
MD Simulation Report on AChE - Lindholdhamine Isomer Interactions

Simulation Details

Jobname: md_job_6H12_1-dock-3
Entry title: 1-dock-3-rec

CPU #	Job Type	Ensemble	Temp. [K]	Sim. Time [ns]	# Atoms	# Waters	Charge
1	mdsim	NPT	300.0	10.005	62848	17954	0

Protein Information

Tot. Residues	Prot. Chain(s)	Res. in Chain(s)	# Atoms	# Heavy Atoms	Charge
562	'NoChainId'	ict_values([562]	8798	4471	-10

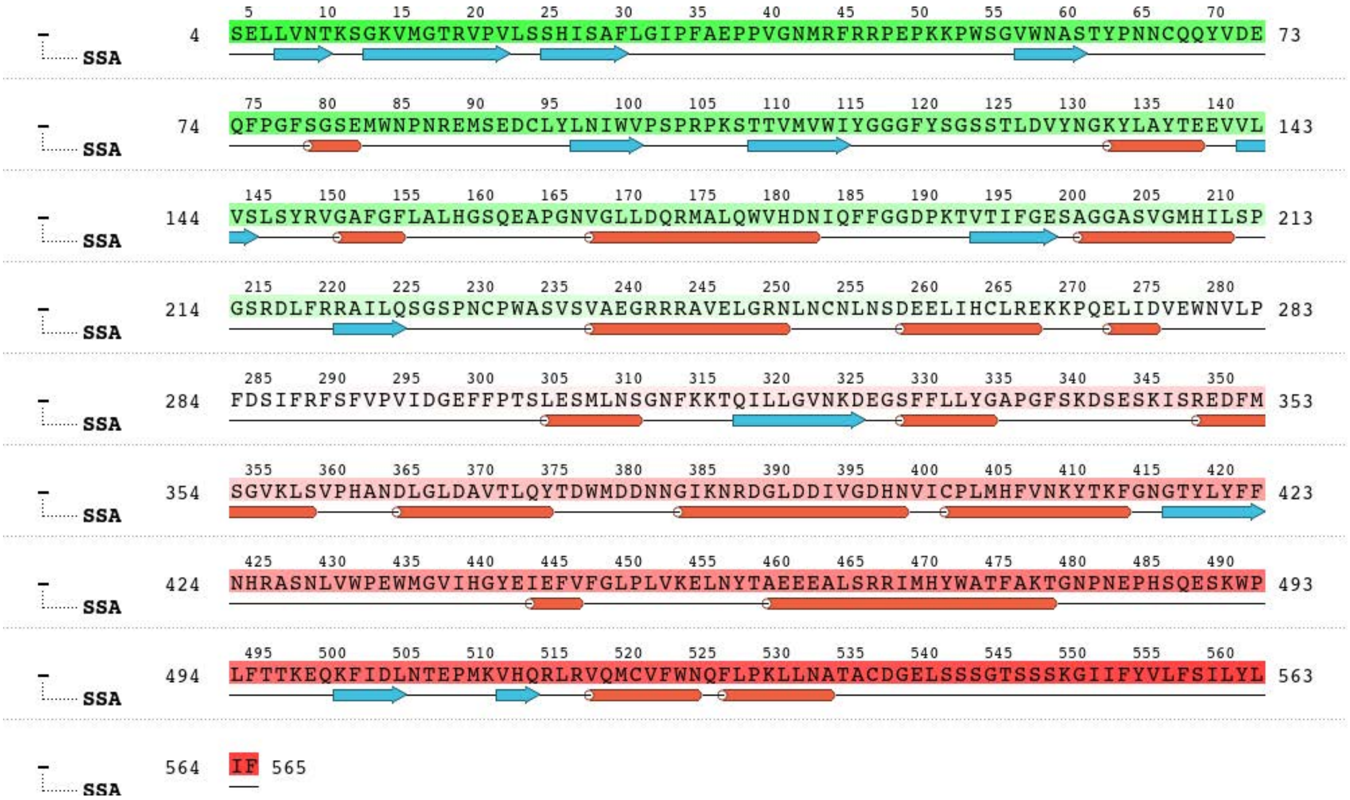

Ligand Information

SMILES	COc(c(c1)O)cc(c12)CC[NH2+][C@H]2Cc3cc(c(O)cc3)Oc(c4)c(OC)cc(c45)CC[NH2+][C@H]5Cc6ccc(O)cc6
PDB Name	'UNK'
Num. of Atoms	80 (total) 42 (heavy)
Atomic Mass	570.692 au
Charge	+2
Mol. Formula	C34H38N2O6
Num. of Fragments	3
Num. of Rot. Bonds	11

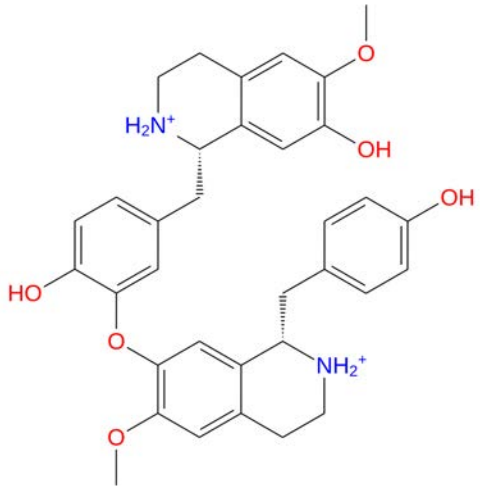

Counter Ion/Salt Information

Type	Num.	Concentration [mM]	Total Charge
Na	58	58.736	+58
Cl	50	50.634	-50

Protein-Ligand RMSD

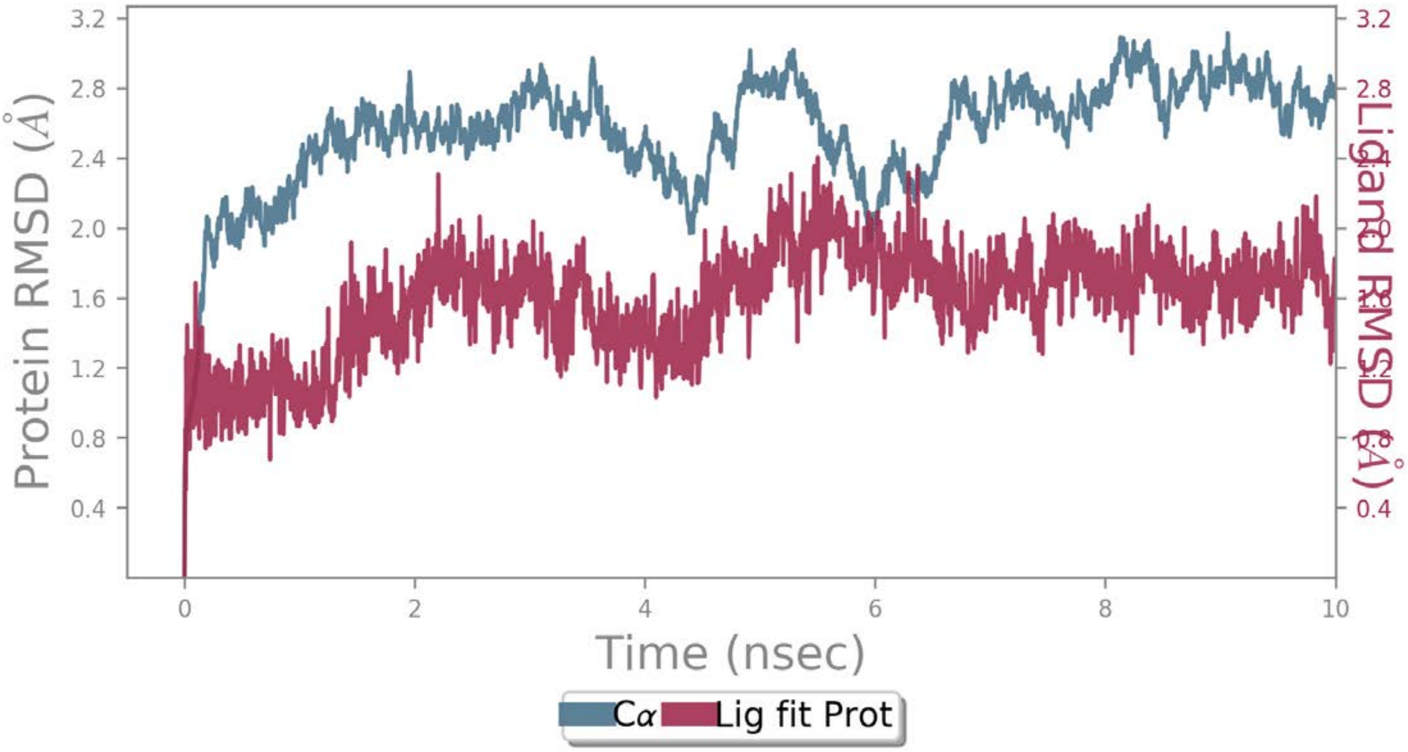

The Root Mean Square Deviation (RMSD) is used to measure the average change in displacement of a selection of atoms for a particular frame with respect to a reference frame. It is calculated for all frames in the trajectory. The RMSD for frame *x* is:

$$RMSD_x = \sqrt{\frac{1}{N}\sum_{i=1}^{N}(r'_i(t_x)) - r_i(t_{ref}))^2}$$

where *N* is the number of atoms in the atom selection; t_{ref} is the reference time, (typically the first frame is used as the reference and it is regarded as time *t*=0); and *r'* is the position of the selected atoms in frame *x* after superimposing on the reference frame, where frame *x* is recorded at time t_x. The procedure is repeated for every frame in the simulation trajectory.

Protein RMSD: The above plot shows the RMSD evolution of a protein (left *Y*-axis). All protein frames are first aligned on the reference frame backbone, and then the RMSD is calculated based on the atom selection. Monitoring the RMSD of the protein can give insights into its structural conformation throughout the simulation. RMSD analysis can indicate if the simulation has equilibrated — its fluctuations towards the end of the simulation are around some thermal average structure. Changes of the order of 1-3 Å are perfectly acceptable for small, globular proteins. Changes much larger than that, however, indicate that the protein is undergoing a large conformational change during the simulation. It is also important that your simulation converges — the RMSD values stabilize around a fixed value. If the RMSD of the protein is still increasing or decreasing on average at the end of the simulation, then your system has not equilibrated, and your simulation may not be long enough for rigorous analysis.

Ligand RMSD: Ligand RMSD (right *Y*-axis) indicates how stable the ligand is with respect to the protein and its binding pocket. In the above plot, 'Lig fit Prot' shows the RMSD of a ligand when the protein-ligand complex is first aligned on the protein backbone of the reference and then the RMSD of the ligand heavy atoms is measured. If the values observed are significantly larger than the RMSD of the protein, then it is likely that the ligand has diffused away from its initial binding site.

Protein RMSF

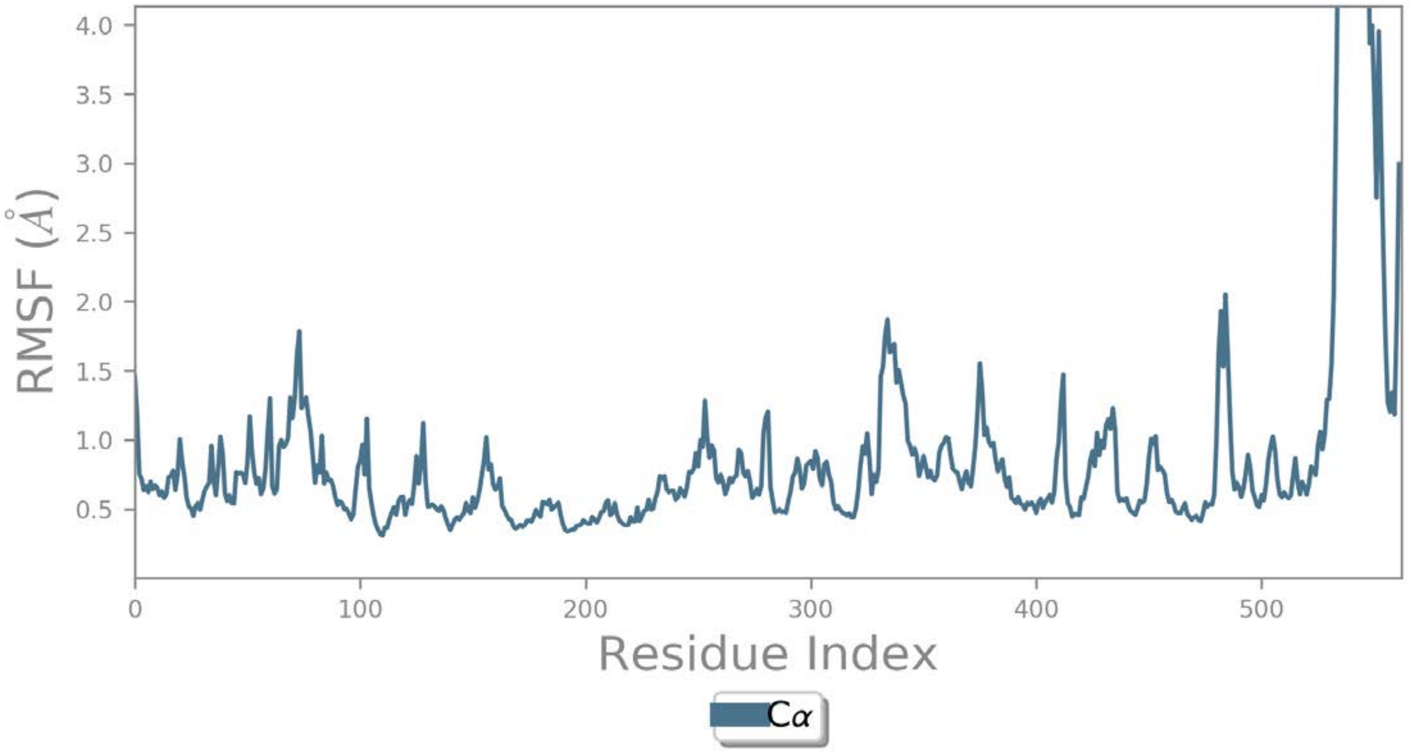

The Root Mean Square Fluctuation (RMSF) is useful for characterizing local changes along the protein chain. The RMSF for residue *i* is:

$$RMSF_i = \sqrt{\frac{1}{T}\sum_{t=1}^{T} < (r_i'(t)) - r_i(t_{ref}))^2 >}$$

where T is the trajectory time over which the RMSF is calculated, t_{ref} is the reference time, r_i is the position of residue *i*; *r'* is the position of atoms in residue *i* after superposition on the reference, and the angle brackets indicate that the average of the square distance is taken over the selection of atoms in the residue.

On this plot, peaks indicate areas of the protein that fluctuate the most during the simulation. Typically you will observe that the tails (*N*- and *C*-terminal) fluctuate more than any other part of the protein. Secondary structure elements like alpha helices and beta strands are usually more rigid than the unstructured part of the protein, and thus fluctuate less than the loop regions.

SCHRÖDINGER®

Protein Secondary Structure

% Helix	% Strand	% Total SSE
28.53	13.37	41.90

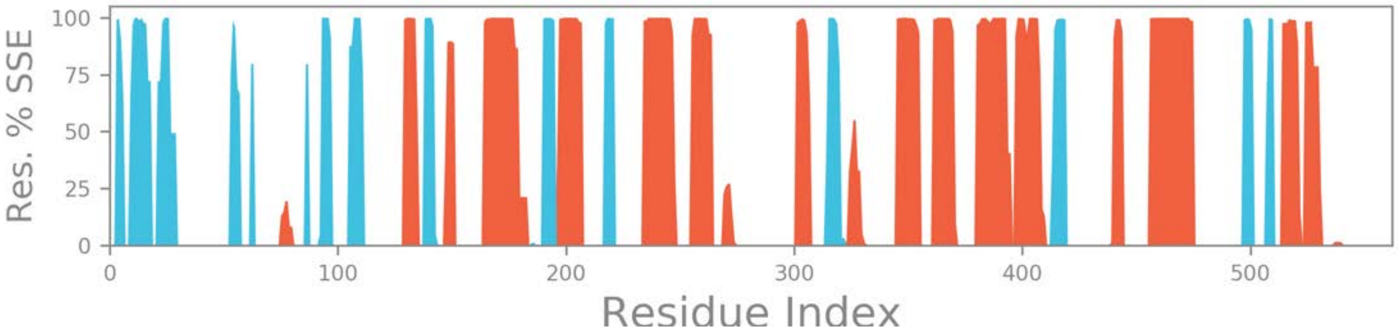

Protein secondary structure elements (SSE) like alpha-helices and beta-strands are monitored throughout the simulation. The plot above reports SSE distribution by residue index throughout the protein structure. The plot below summarizes the SSE composition for each trajectory frame over the course of the simulation, and the plot at the bottom monitors each residue and its SSE assignment over time.

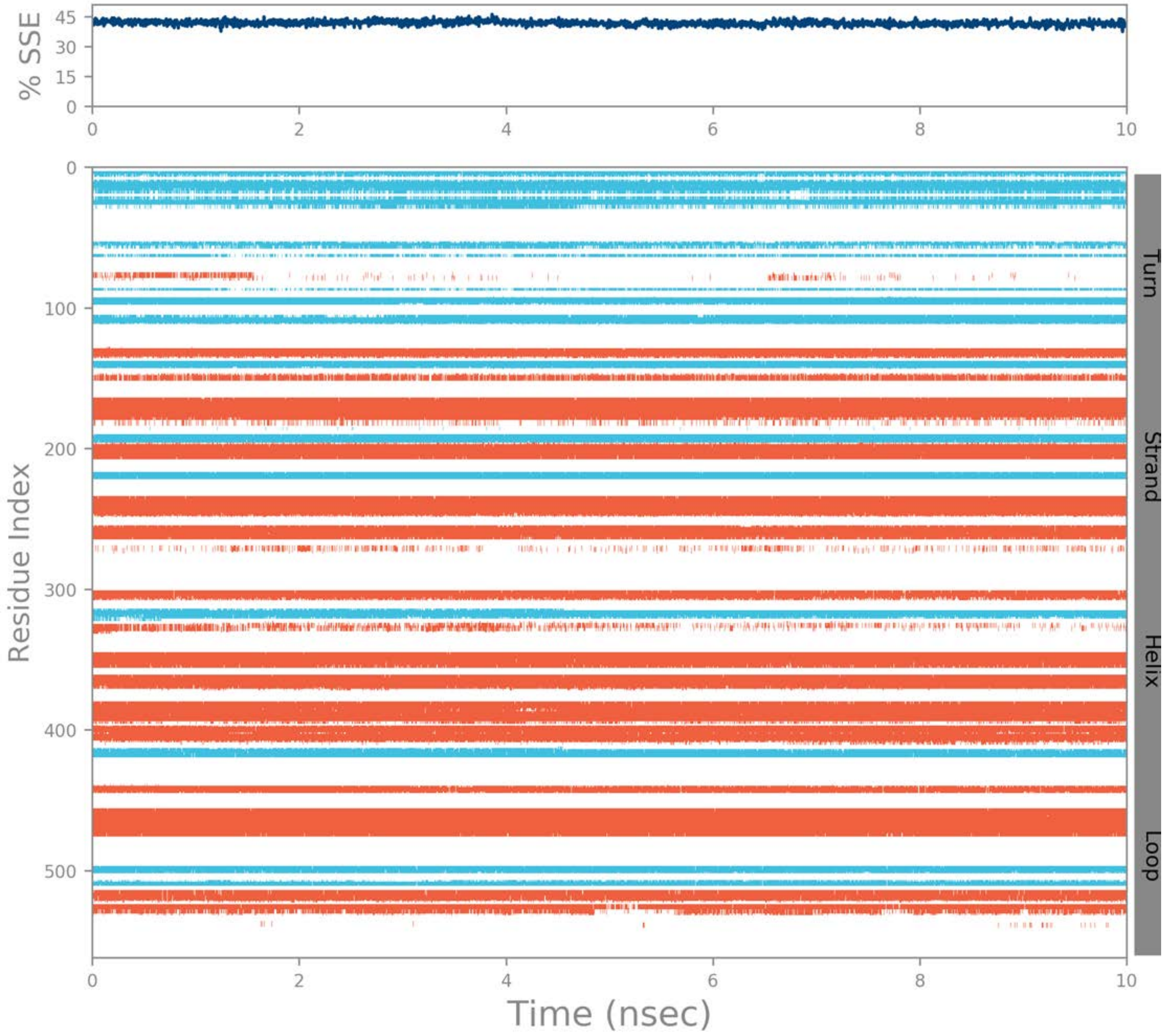

RMSF of Lindholdamine Isomer Ligand

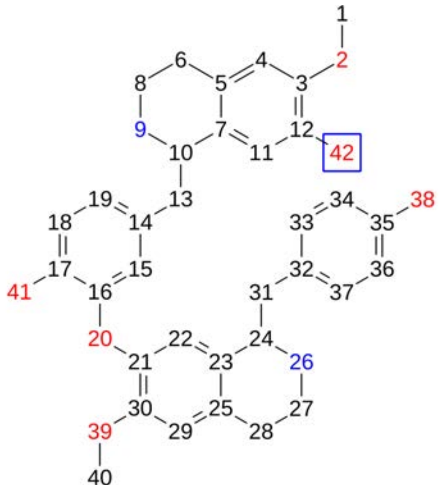

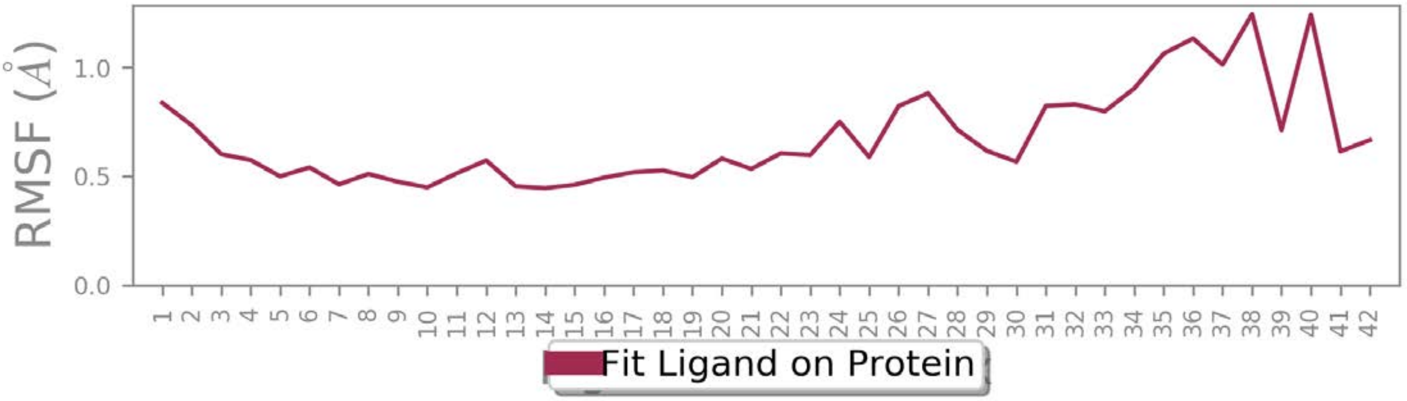

The Ligand Root Mean Square Fluctuation (L-RMSF) is useful for characterizing changes in the ligand atom positions. The RMSF for atom *i* is:

$$RMSF_i = \sqrt{\frac{1}{T}\sum_{t=1}^{T}(r'_i(t)) - r_i(t_{ref}))^2}$$

where *T* is the trajectory time over which the RMSF is calculated, t_{ref} is the reference time (usually for the first frame, and is regarded as the *zero* of time); *r* is the position of atom *i* in the reference at time t_{ref}, and *r'* is the position of atom *i* at time *t* after superposition on the reference frame.

Ligand RMSF shows the ligand's fluctuations broken down by atom, corresponding to the 2D structure in the top panel. The ligand RMSF may give you insights on how ligand fragments interact with the protein and their entropic role in the binding event. In the bottom panel, the 'Fit Ligand on Protein' line shows the ligand fluctuations, with respect to the protein. The protein-ligand complex is first aligned on the protein backbone and then the ligand RMSF is measured on the ligand heavy atoms.

Protein-Ligand Contacts

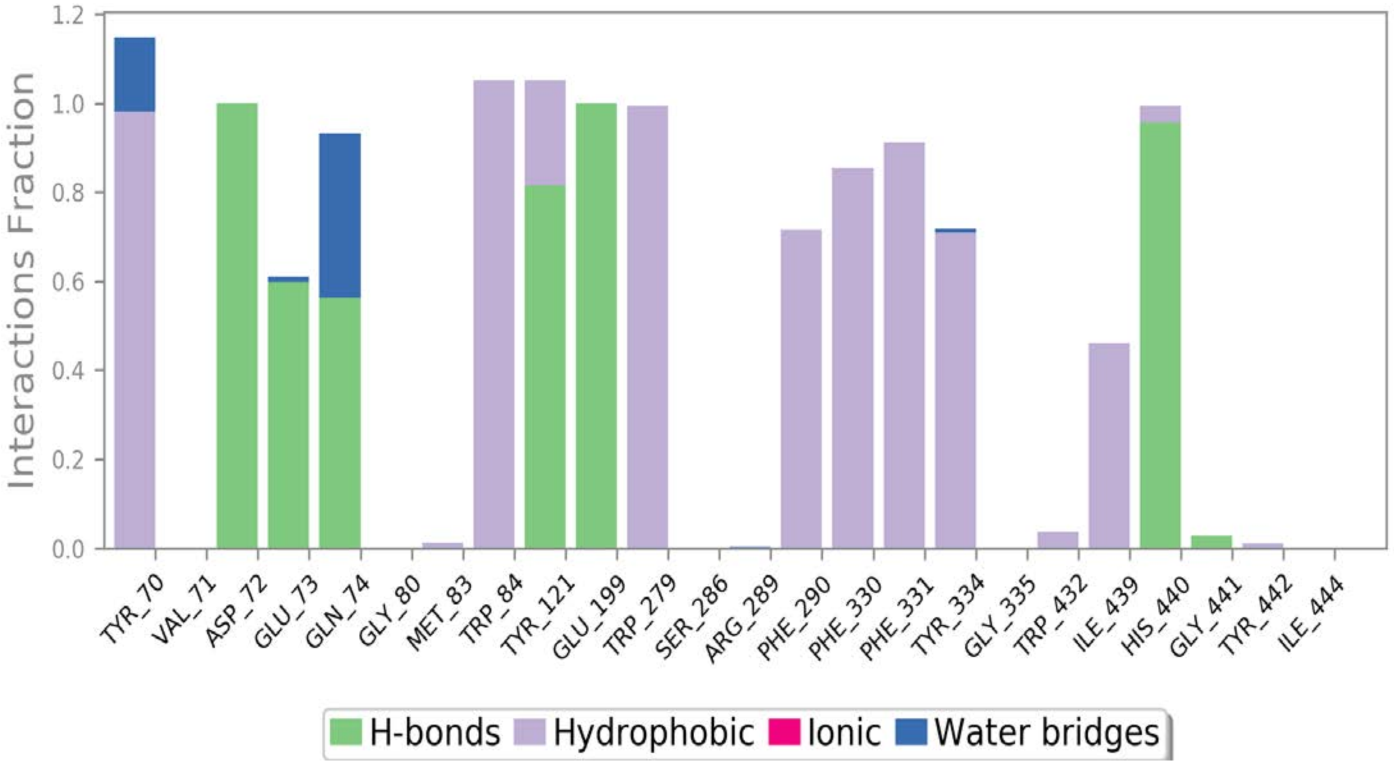

Protein interactions with the ligand can be monitored throughout the simulation. These interactions can be categorized by type and summarized, as shown in the plot above. Protein-ligand interactions (or 'contacts') are categorized into four types: Hydrogen Bonds, Hydrophobic, Ionic and Water Bridges. Each interaction type contains more specific subtypes, which can be explored through the 'Simulation Interactions Diagram' panel. The stacked bar charts are normalized over the course of the trajectory: for example, a value of 0.7 suggests that 70% of the simulation time the specific interaction is maintained. Values over 1.0 are possible as some protein residue may make multiple contacts of same subtype with the ligand.

Hydrogen Bonds: (H-bonds) play a significant role in ligand binding. Consideration of hydrogen-bonding properties in drug design is important because of their strong influence on drug specificity, metabolization and adsorption. Hydrogen bonds between a protein and a ligand can be further broken down into four subtypes: backbone acceptor; backbone donor; side-chain acceptor; side-chain donor.
The current geometric criteria for protein-ligand H-bond is: distance of 2.5 Å between the donor and acceptor atoms (D—H···A); a donor angle of ≥120° between the donor-hydrogen-acceptor atoms (D—H···A); and an acceptor angle of ≥90° between the hydrogen-acceptor-bonded_atom atoms (H···A—X).

Hydrophobic contacts: fall into three subtypes: π-Cation; π-π; and Other, non-specific interactions. Generally these type of interactions involve a hydrophobic amino acid and an aromatic or aliphatic group on the ligand, but we have extended this category to also include π-Cation interactions.
The current geometric criteria for hydrophobic interactions is as follows: π-Cation — Aromatic and charged groups within 4.5 Å; π-π — Two aromatic groups stacked face-to-face or face-to-edge; Other — A non-specific hydrophobic sidechain within 3.6 Å of a ligand's aromatic or aliphatic carbons.

Ionic interactions: or polar interactions, are between two oppositely charged atoms that are within 3.7 Å of each other and do not involve a hydrogen bond. We also monitor Protein-Metal-Ligand interactions, which are defined by a metal ion coordinated within 3.4 Å of protein's and ligand's heavy atoms (except carbon). All ionic interactions are broken down into two subtypes: those mediated by a protein backbone or side chains.

Water Bridges: are hydrogen-bonded protein-ligand interactions mediated by a water molecule. The hydrogen-bond geometry is slightly relaxed from the standard H-bond definition.
The current geometric criteria for a protein-water or water-ligand H-bond are: a distance of 2.8 Å between the donor and acceptor atoms (D—H···A); a donor angle of ≥110° between the donor-hydrogen-acceptor atoms (D—H···A); and an acceptor angle of ≥90° between the hydrogen-acceptor-bonded_atom atoms (H···A—X).

Protein-Ligand Contacts (cont.)

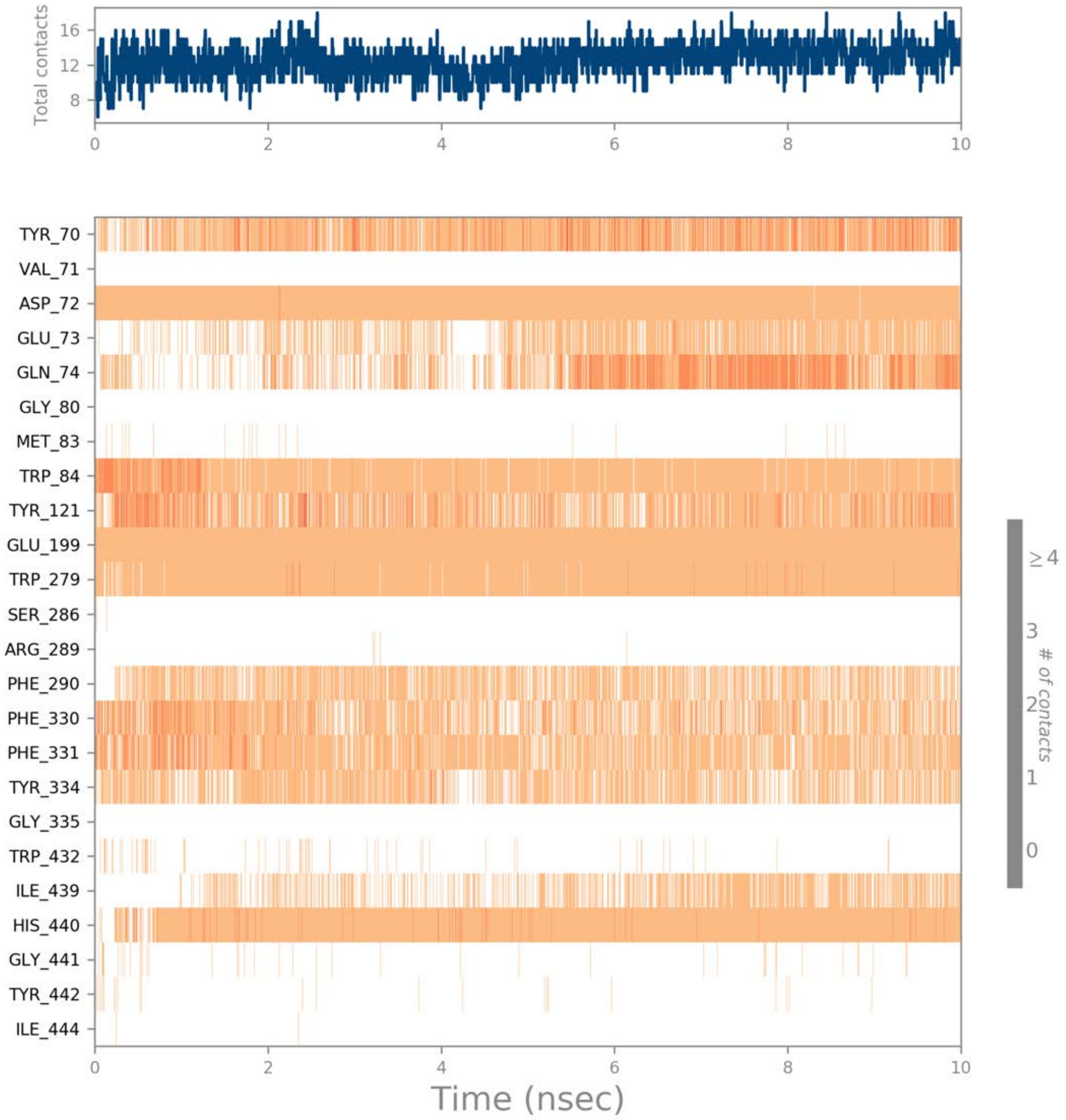

A timeline representation of the interactions and contacts (**H-bonds**, **Hydrophobic**, **Ionic**, **Water bridges**) summarized in the previous page. The top panel shows the total number of specific contacts the protein makes with the ligand over the course of the trajectory. The bottom panel shows which residues interact with the ligand in each trajectory frame. Some residues make more than one specific contact with the ligand, which is represented by a darker shade of orange, according to the scale to the right of the plot.

Ligand-Protein Contacts

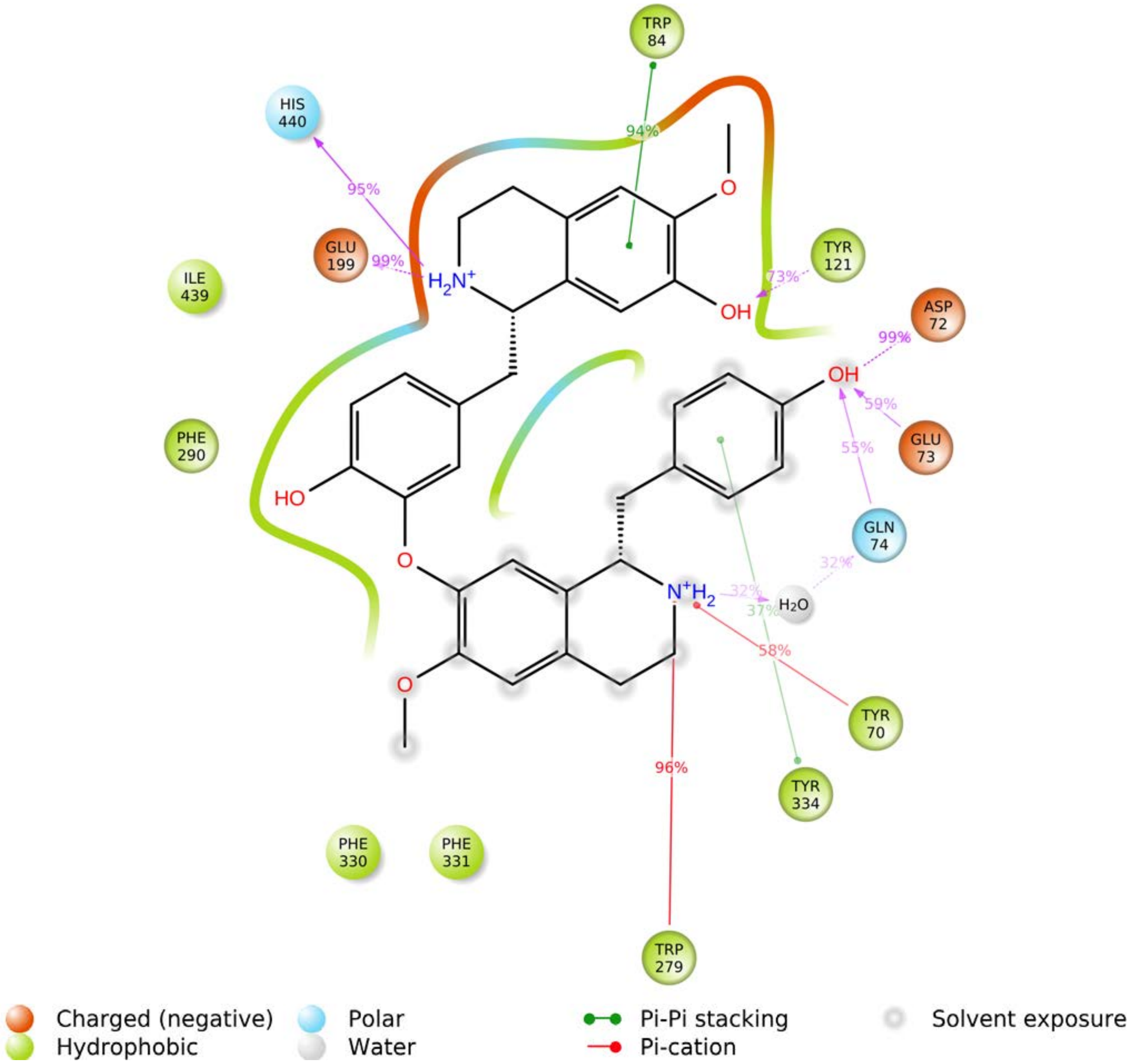

A schematic of detailed ligand atom interactions with the protein residues. Interactions that occur more than **30.0%** of the simulation time in the selected trajectory (0.00 through 10.00 nsec), are shown.
Note: it is possible to have interactions with >100% as some residues may have multiple interactions of a single type with the same ligand atom. For example, the ARG side chain has four H-bond donors that can all hydrogen-bond to a single H-bond acceptor.

SCHRÖDINGER®

Ligand Torsion Profile

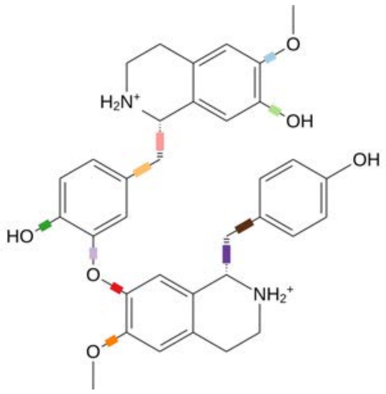

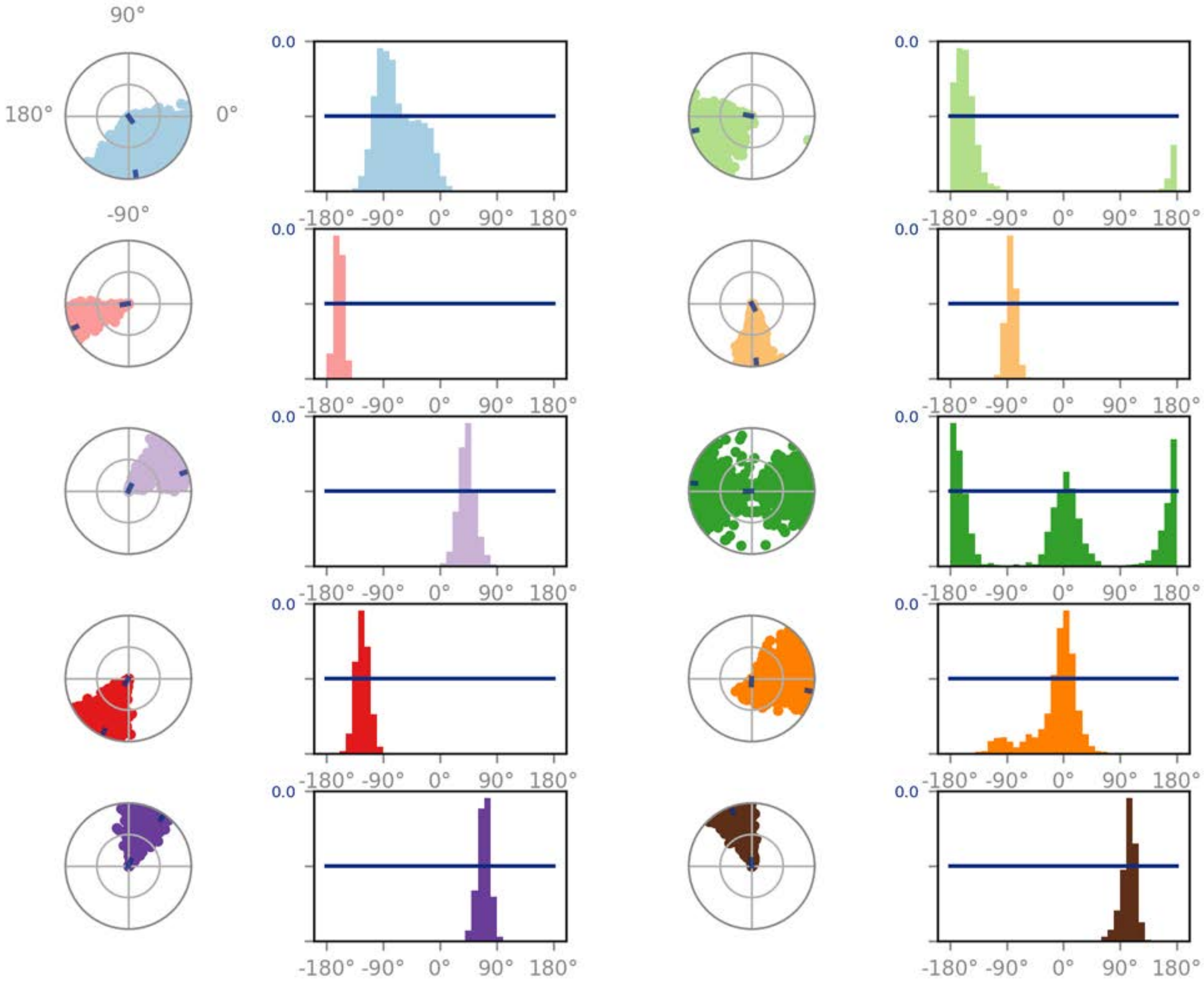

Ligand Torsion Profile (cont.)

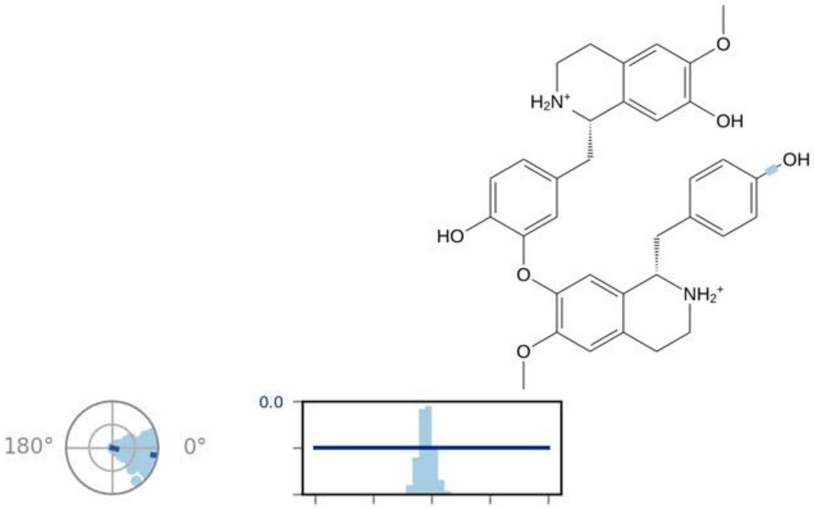

The ligand torsions plot summarizes the conformational evolution of every rotatable bond (RB) in the ligand throughout the simulation trajectory (0.00 through 10.00 nsec). The top panel shows the 2d schematic of a ligand with color-coded rotatable bonds. Each rotatable bond torsion is accompanied by a dial plot and bar plots of the same color.

Dial (or radial) plots describe the conformation of the torsion throughout the course of the simulation. The beginning of the simulation is in the center of the radial plot and the time evolution is plotted radially outwards.

The bar plots summarize the data on the dial plots, by showing the probability density of the torsion. If torsional potential information is available, the plot also shows the potential of the rotatable bond (by summing the potential of the related torsions). The values of the potential are on the left *Y*-axis of the chart, and are expressed in *kcal/mol*. Looking at the histogram and torsion potential relationships may give insights into the conformational strain the ligand undergoes to maintain a protein-bound conformation.

Ligand Properties

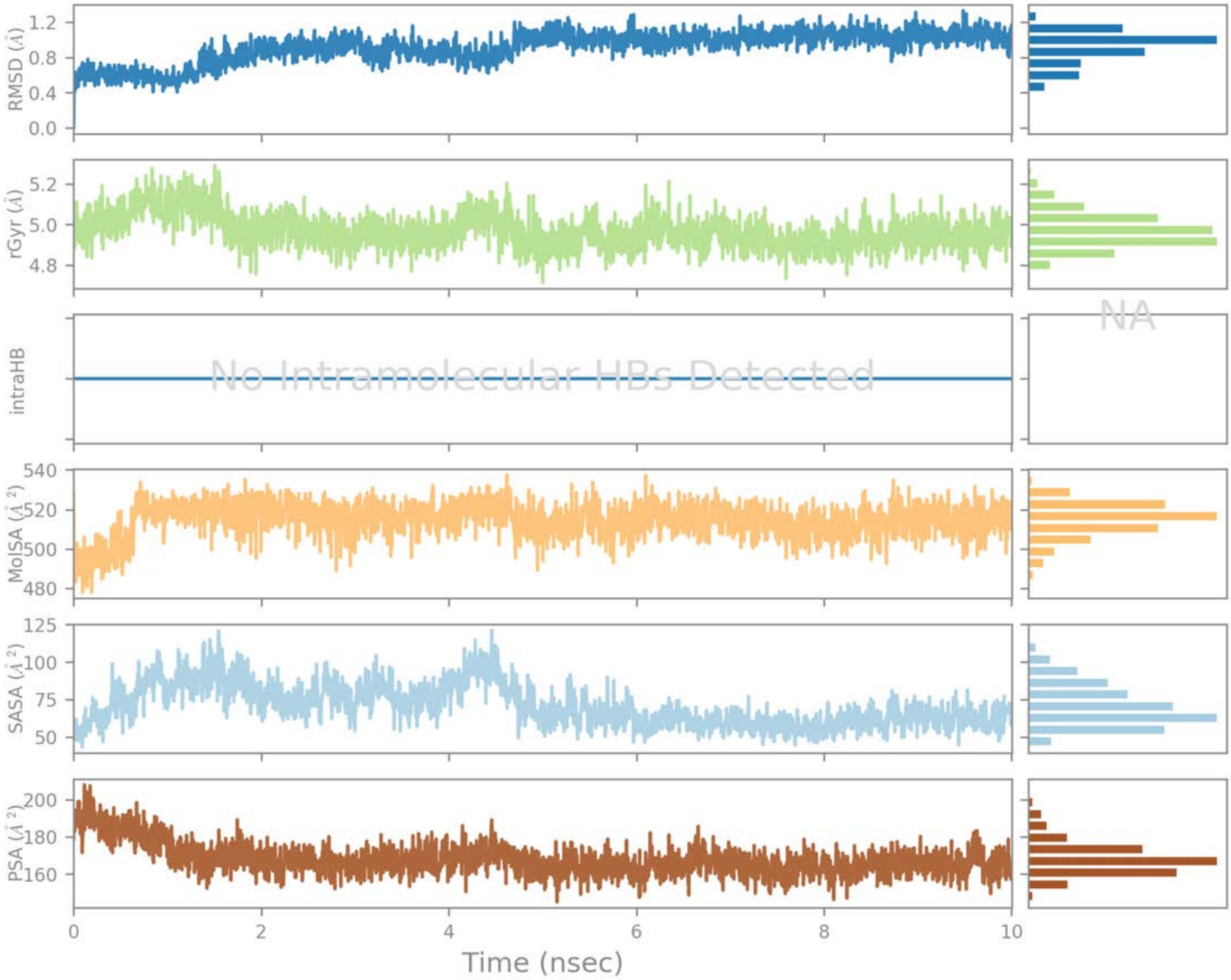

Ligand RMSD: Root mean square deviation of a ligand with respect to the reference conformation (typically the first frame is used as the reference and it is regarded as time *t*=0).

Radius of Gyration (rGyr): Measures the 'extendedness' of a ligand, and is equivalent to its principal moment of inertia.

Intramolecular Hydrogen Bonds (intraHB): Number of internal hydrogen bonds (HB) within a ligand molecule.

Molecular Surface Area (MolSA): Molecular surface calculation with 1.4 Å probe radius. This value is equivalent to a van der Waals surface area.

Solvent Accessible Surface Area (SASA): Surface area of a molecule accessible by a water molecule.

Polar Surface Area (PSA): Solvent accessible surface area in a molecule contributed only by oxygen and nitrogen atoms.

Report S3
MD Simulation Report on AChE - Stepharine Interactions

Simulation Details

Jobname: md_job_6H12_2-dock-2
Entry title: 2-dock-2

CPU #	Job Type	Ensemble	Temp. [K]	Sim. Time [ns]	# Atoms	# Waters	Charge
1	mdsim	NPT	300.0	10.005	62796	17949	0

Protein Information

Tot. Residues	Prot. Chain(s)	Res. in Chain(s)	# Atoms	# Heavy Atoms	Charge
562	'NoChainId'	ict_values([562]	8798	4471	-10

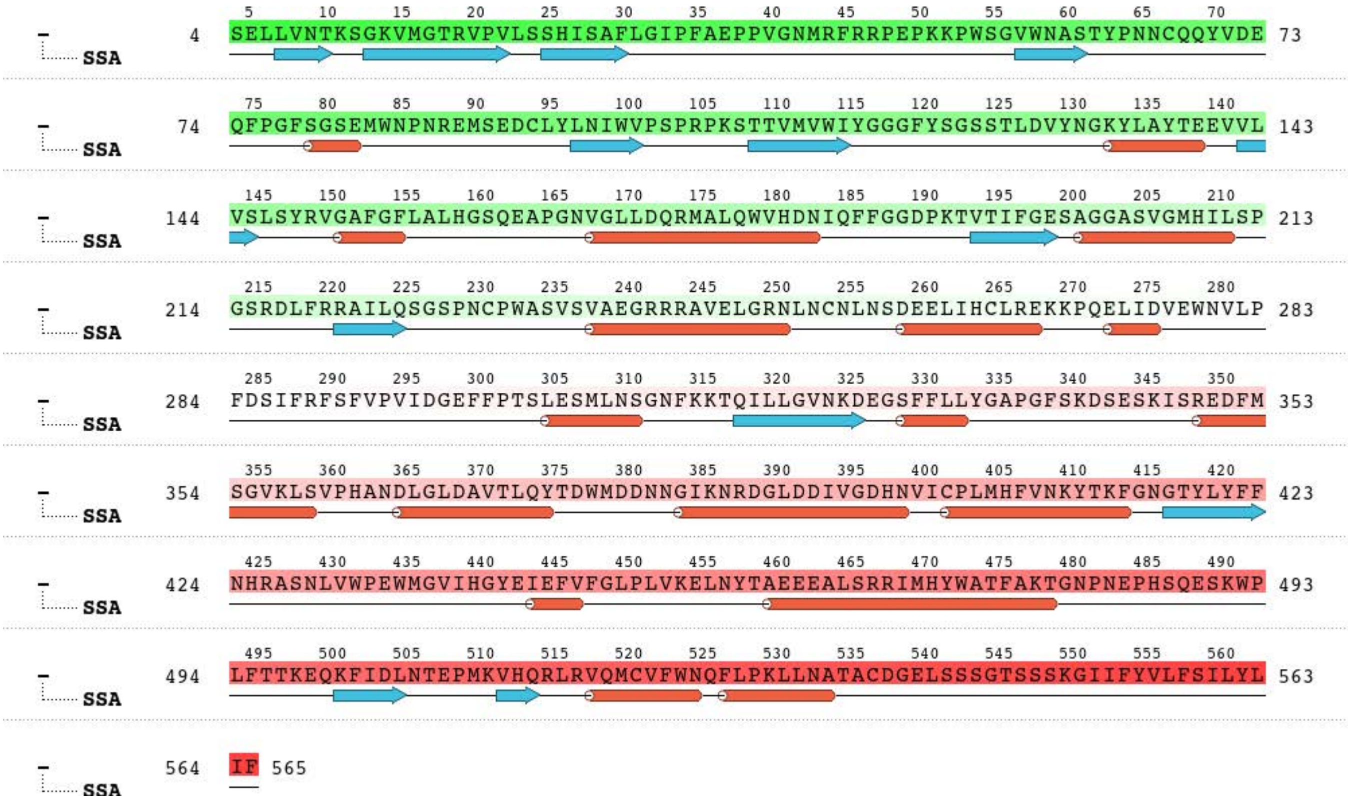

Ligand Information

SMILES	COc1c(OC)cc(CC[NH2+][C@H]2C3)c2c1C34C=CC(=O)C=C4
PDB Name	'UNK'
Num. of Atoms	42 (total) 22 (heavy)
Atomic Mass	298.365 au
Charge	+1
Mol. Formula	C18H20NO3
Num. of Fragments	1
Num. of Rot. Bonds	2

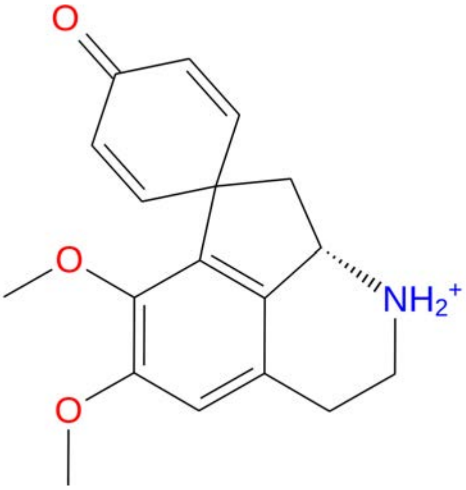

Counter Ion/Salt Information

Type	Num.	Concentration [mM]	Total Charge
Na	59	59.765	+59
Cl	50	50.649	-50

Protein-Ligand RMSD

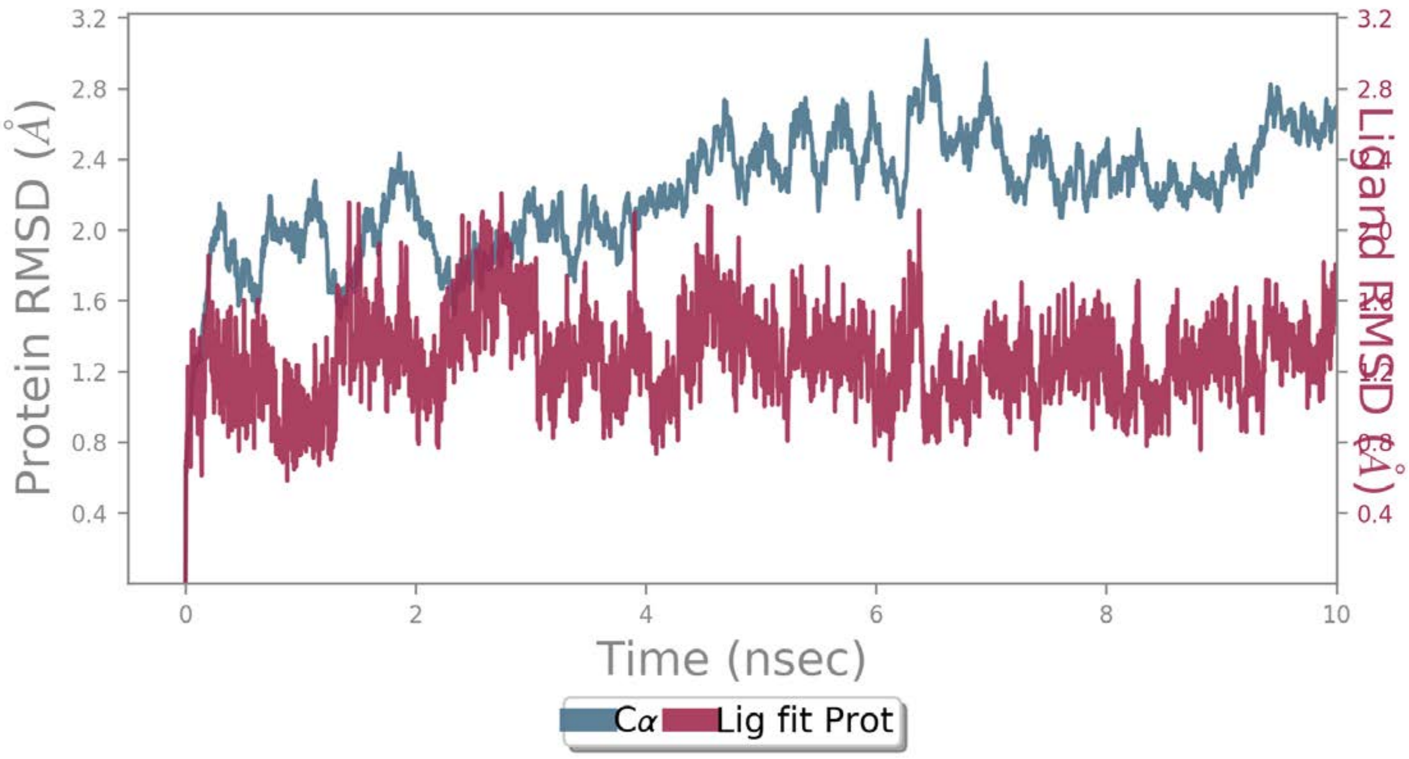

The Root Mean Square Deviation (RMSD) is used to measure the average change in displacement of a selection of atoms for a particular frame with respect to a reference frame. It is calculated for all frames in the trajectory. The RMSD for frame *x* is:

$$RMSD_x = \sqrt{\frac{1}{N}\sum_{i=1}^{N}(r'_i(t_x)) - r_i(t_{ref}))^2}$$

where *N* is the number of atoms in the atom selection; t_{ref} is the reference time, (typically the first frame is used as the reference and it is regarded as time *t*=0); and *r'* is the position of the selected atoms in frame *x* after superimposing on the reference frame, where frame *x* is recorded at time t_x. The procedure is repeated for every frame in the simulation trajectory.

Protein RMSD: The above plot shows the RMSD evolution of a protein (left *Y*-axis). All protein frames are first aligned on the reference frame backbone, and then the RMSD is calculated based on the atom selection. Monitoring the RMSD of the protein can give insights into its structural conformation throughout the simulation. RMSD analysis can indicate if the simulation has equilibrated — its fluctuations towards the end of the simulation are around some thermal average structure. Changes of the order of 1-3 Å are perfectly acceptable for small, globular proteins. Changes much larger than that, however, indicate that the protein is undergoing a large conformational change during the simulation. It is also important that your simulation converges — the RMSD values stabilize around a fixed value. If the RMSD of the protein is still increasing or decreasing on average at the end of the simulation, then your system has not equilibrated, and your simulation may not be long enough for rigorous analysis.

Ligand RMSD: Ligand RMSD (right *Y*-axis) indicates how stable the ligand is with respect to the protein and its binding pocket. In the above plot, 'Lig fit Prot' shows the RMSD of a ligand when the protein-ligand complex is first aligned on the protein backbone of the reference and then the RMSD of the ligand heavy atoms is measured. If the values observed are significantly larger than the RMSD of the protein, then it is likely that the ligand has diffused away from its initial binding site.

Protein RMSF

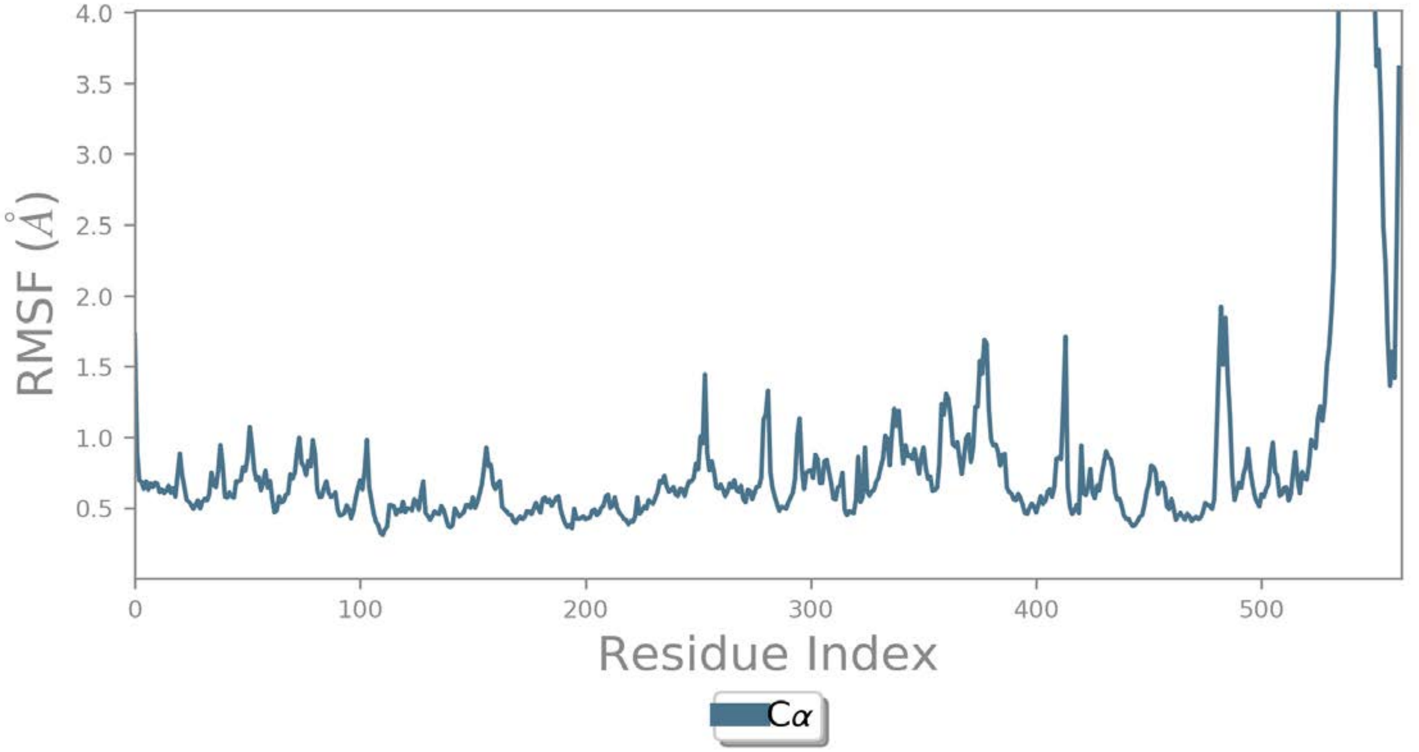

The Root Mean Square Fluctuation (RMSF) is useful for characterizing local changes along the protein chain. The RMSF for residue *i* is:

$$RMSF_i = \sqrt{\frac{1}{T}\sum_{t=1}^{T} < (r_i'(t)) - r_i(t_{ref}))^2 >}$$

where T is the trajectory time over which the RMSF is calculated, t_{ref} is the reference time, r_i is the position of residue *i*; *r'* is the position of atoms in residue *i* after superposition on the reference, and the angle brackets indicate that the average of the square distance is taken over the selection of atoms in the residue.

On this plot, peaks indicate areas of the protein that fluctuate the most during the simulation. Typically you will observe that the tails (*N*- and *C*-terminal) fluctuate more than any other part of the protein. Secondary structure elements like alpha helices and beta strands are usually more rigid than the unstructured part of the protein, and thus fluctuate less than the loop regions.

Protein Secondary Structure

% Helix	% Strand	% Total SSE
28.80	13.08	41.88

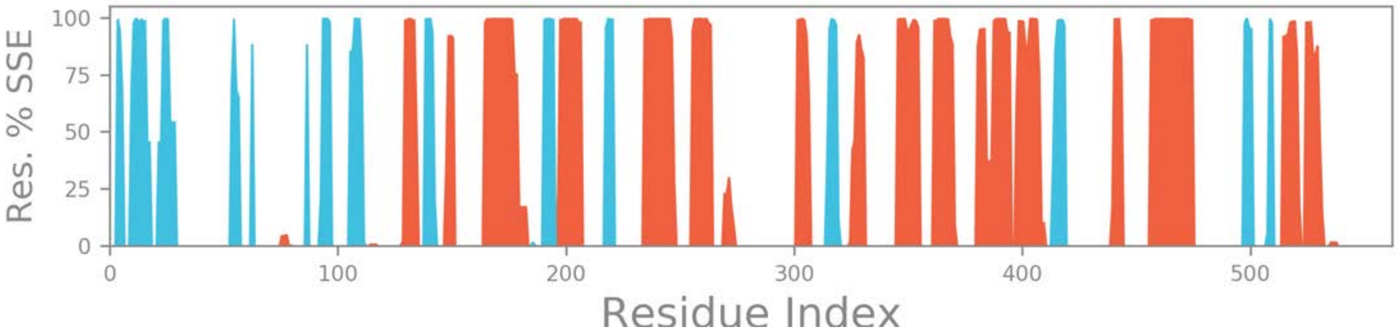

Protein secondary structure elements (SSE) like alpha-helices and beta-strands are monitored throughout the simulation. The plot above reports SSE distribution by residue index throughout the protein structure. The plot below summarizes the SSE composition for each trajectory frame over the course of the simulation, and the plot at the bottom monitors each residue and its SSE assignment over time.

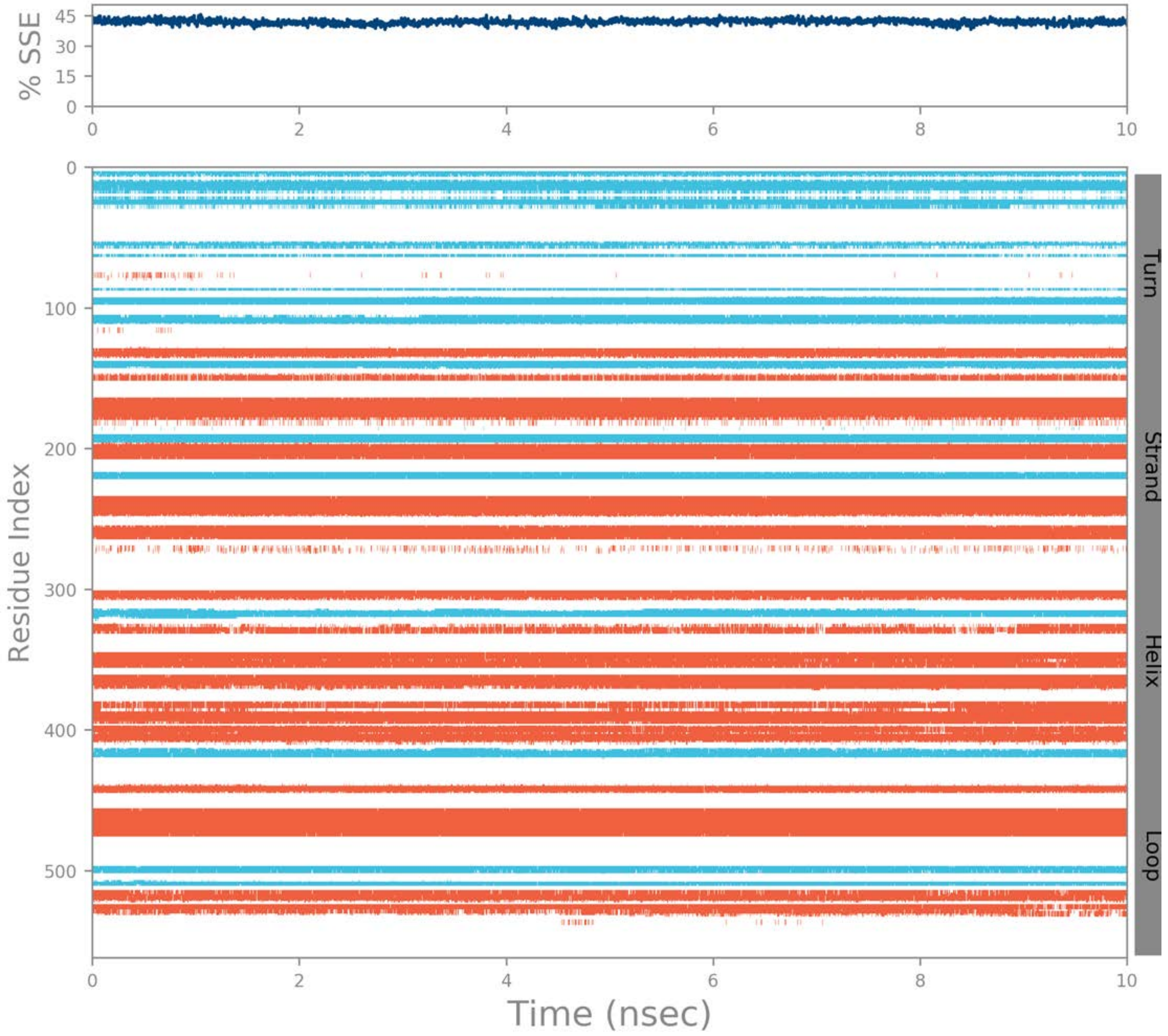

Stepharine Ligand RMSF

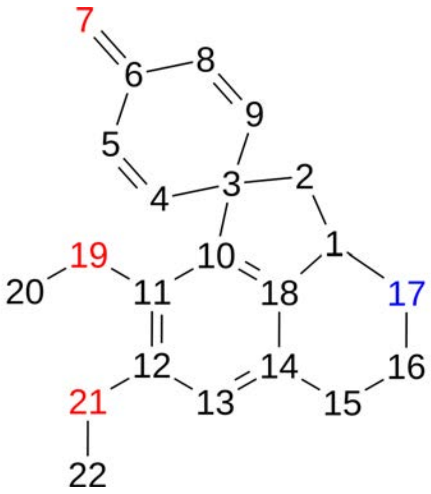

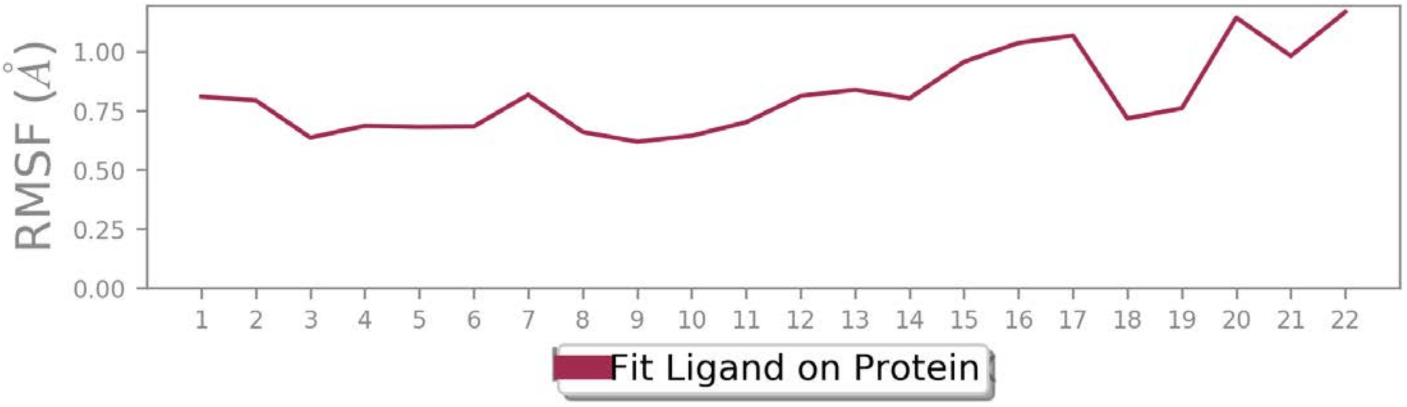

The Ligand Root Mean Square Fluctuation (L-RMSF) is useful for characterizing changes in the ligand atom positions. The RMSF for atom *i* is:

$$RMSF_i = \sqrt{\frac{1}{T}\sum_{t=1}^{T}(r_i'(t)) - r_i(t_{ref}))^2}$$

where T is the trajectory time over which the RMSF is calculated, t_{ref} is the reference time (usually for the first frame, and is regarded as the *zero* of time); r is the position of atom i in the reference at time t_{ref}, and r' is the position of atom i at time t after superposition on the reference frame.

Ligand RMSF shows the ligand's fluctuations broken down by atom, corresponding to the 2D structure in the top panel. The ligand RMSF may give you insights on how ligand fragments interact with the protein and their entropic role in the binding event. In the bottom panel, the 'Fit Ligand on Protein' line shows the ligand fluctuations, with respect to the protein. The protein-ligand complex is first aligned on the protein backbone and then the ligand RMSF is measured on the ligand heavy atoms.

Protein-Ligand Contacts

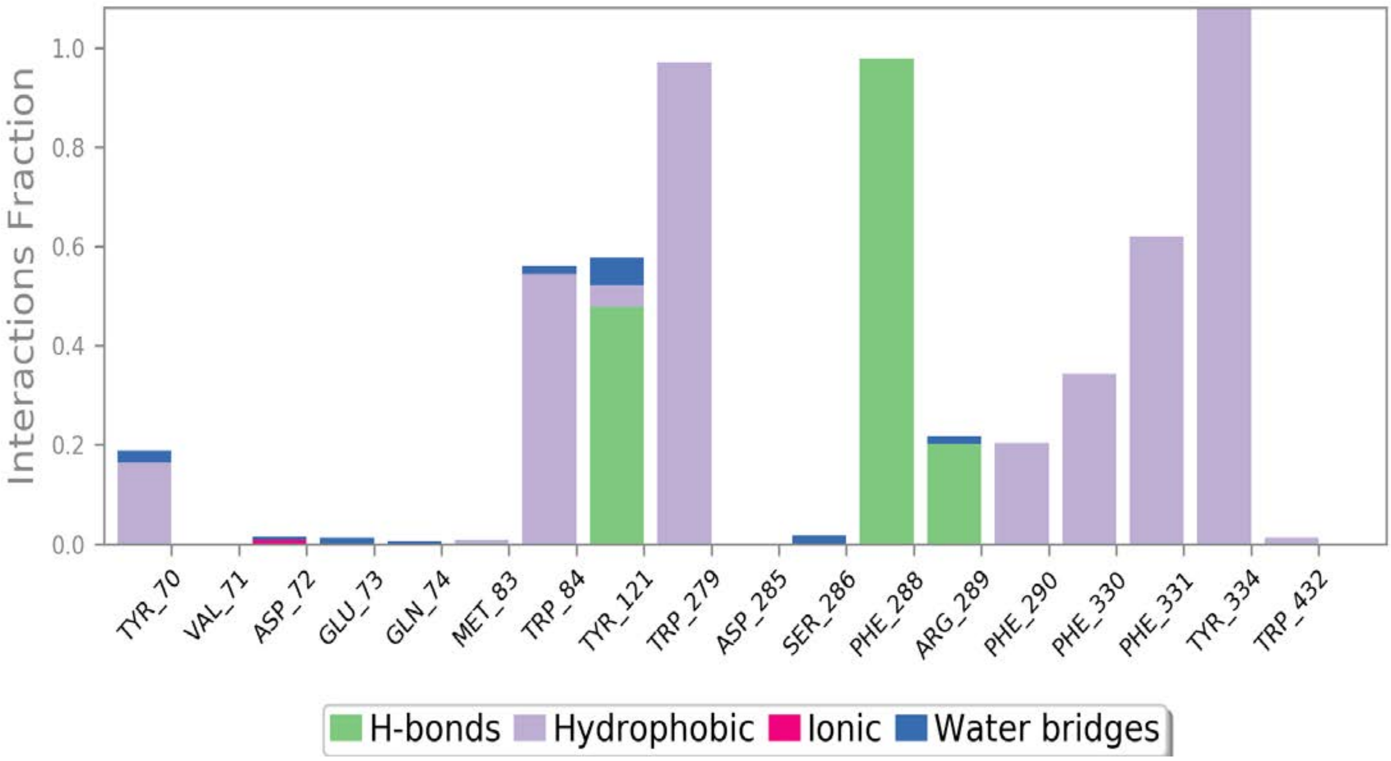

Protein interactions with the ligand can be monitored throughout the simulation. These interactions can be categorized by type and summarized, as shown in the plot above. Protein-ligand interactions (or 'contacts') are categorized into four types: Hydrogen Bonds, Hydrophobic, Ionic and Water Bridges. Each interaction type contains more specific subtypes, which can be explored through the 'Simulation Interactions Diagram' panel. The stacked bar charts are normalized over the course of the trajectory: for example, a value of 0.7 suggests that 70% of the simulation time the specific interaction is maintained. Values over 1.0 are possible as some protein residue may make multiple contacts of same subtype with the ligand.

Hydrogen Bonds: (H-bonds) play a significant role in ligand binding. Consideration of hydrogen-bonding properties in drug design is important because of their strong influence on drug specificity, metabolization and adsorption. Hydrogen bonds between a protein and a ligand can be further broken down into four subtypes: backbone acceptor; backbone donor; side-chain acceptor; side-chain donor.
The current geometric criteria for protein-ligand H-bond is: distance of 2.5 Å between the donor and acceptor atoms (D—H···A); a donor angle of ≥120° between the donor-hydrogen-acceptor atoms (D—H···A); and an acceptor angle of ≥90° between the hydrogen-acceptor-bonded_atom atoms (H···A—X).

Hydrophobic contacts: fall into three subtypes: π-Cation; π-π; and Other, non-specific interactions. Generally these type of interactions involve a hydrophobic amino acid and an aromatic or aliphatic group on the ligand, but we have extended this category to also include π-Cation interactions.
The current geometric criteria for hydrophobic interactions is as follows: π-Cation — Aromatic and charged groups within 4.5 Å; π-π — Two aromatic groups stacked face-to-face or face-to-edge; Other — A non-specific hydrophobic sidechain within 3.6 Å of a ligand's aromatic or aliphatic carbons.

Ionic interactions: or polar interactions, are between two oppositely charged atoms that are within 3.7 Å of each other and do not involve a hydrogen bond. We also monitor Protein-Metal-Ligand interactions, which are defined by a metal ion coordinated within 3.4 Å of protein's and ligand's heavy atoms (except carbon). All ionic interactions are broken down into two subtypes: those mediated by a protein backbone or side chains.

Water Bridges: are hydrogen-bonded protein-ligand interactions mediated by a water molecule. The hydrogen-bond geometry is slightly relaxed from the standard H-bond definition.
The current geometric criteria for a protein-water or water-ligand H-bond are: a distance of 2.8 Å between the donor and acceptor atoms (D—H···A); a donor angle of ≥110° between the donor-hydrogen-acceptor atoms (D—H···A); and an acceptor angle of ≥90° between the hydrogen-acceptor-bonded_atom atoms (H···A—X).

Protein-Ligand Contacts (cont.)

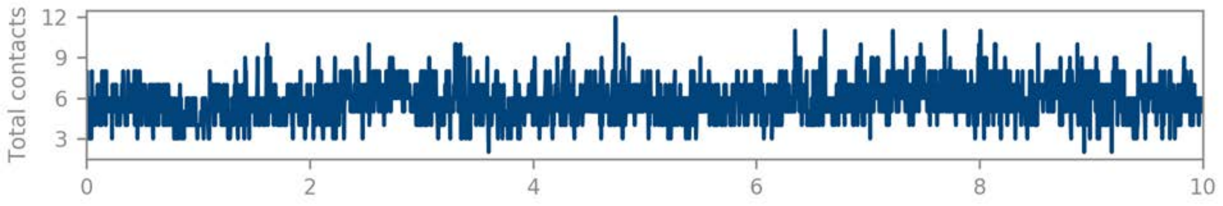

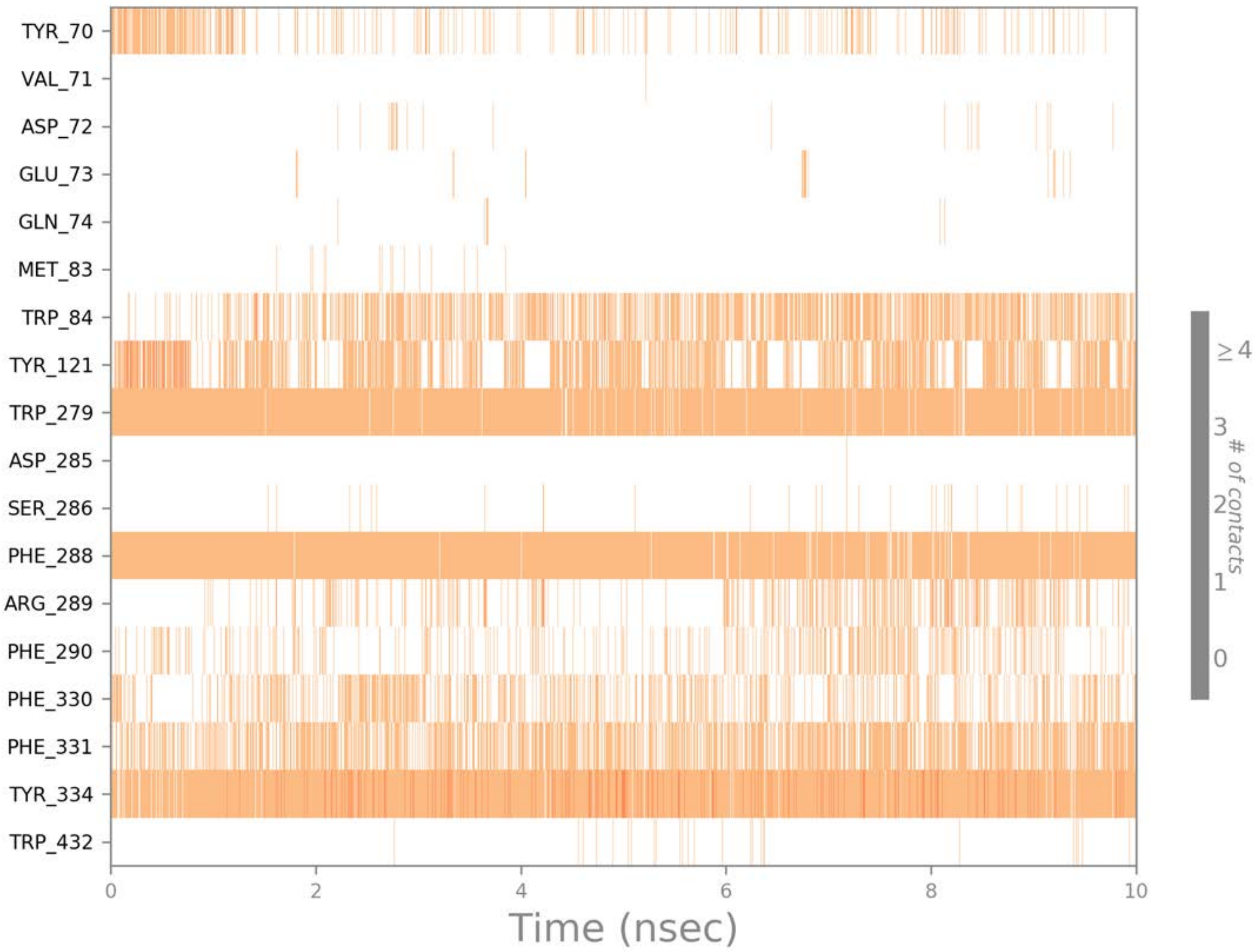

A timeline representation of the interactions and contacts (**H-bonds**, **Hydrophobic**, **Ionic**, **Water bridges**) summarized in the previous page. The top panel shows the total number of specific contacts the protein makes with the ligand over the course of the trajectory. The bottom panel shows which residues interact with the ligand in each trajectory frame. Some residues make more than one specific contact with the ligand, which is represented by a darker shade of orange, according to the scale to the right of the plot.

Ligand-Protein Contacts

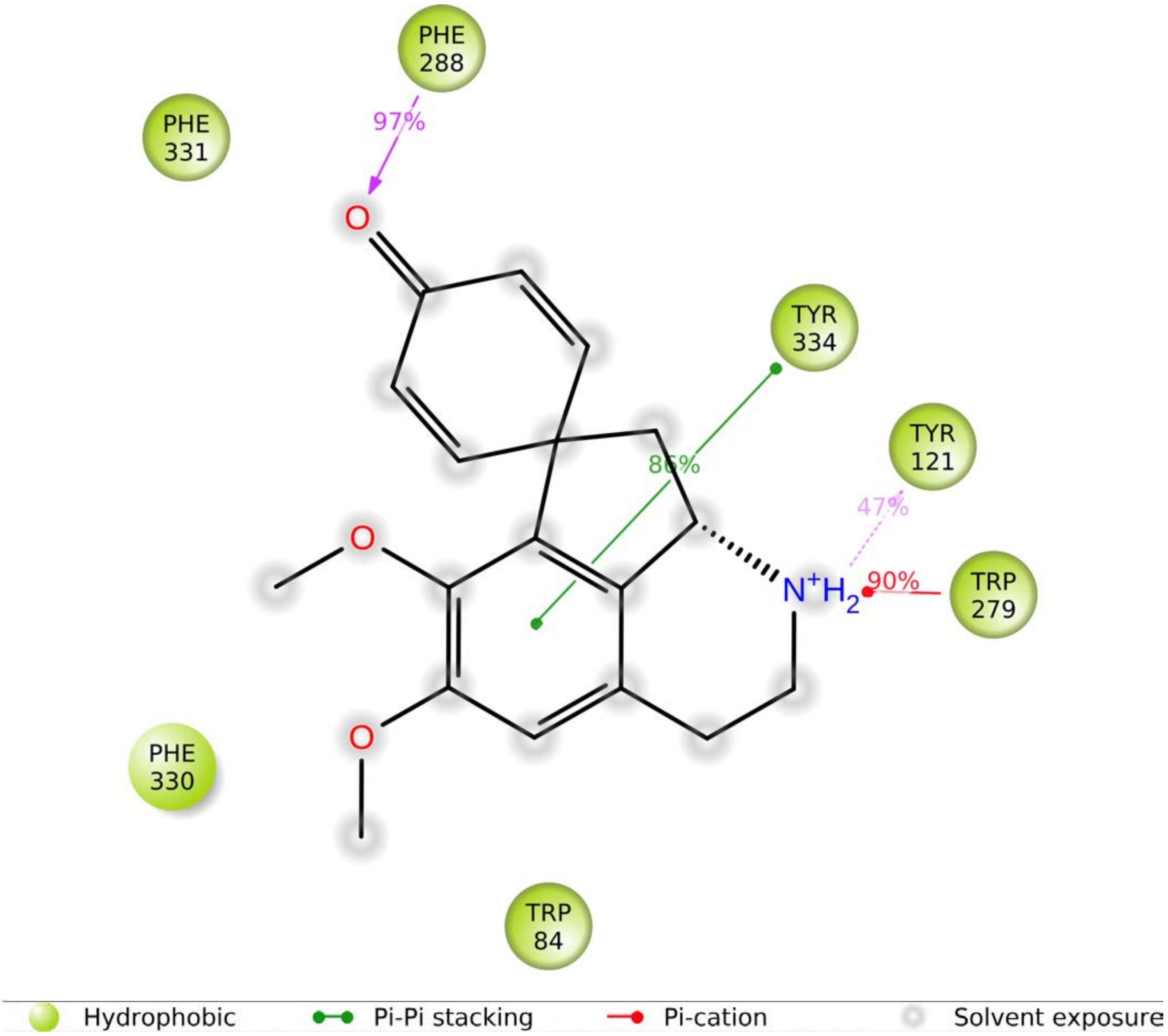

A schematic of detailed ligand atom interactions with the protein residues. Interactions that occur more than **30.0%** of the simulation time in the selected trajectory (0.00 through 10.00 nsec), are shown.
Note: it is possible to have interactions with >100% as some residues may have multiple interactions of a single type with the same ligand atom. For example, the ARG side chain has four H-bond donors that can all hydrogen-bond to a single H-bond acceptor.

Ligand Torsion Profile

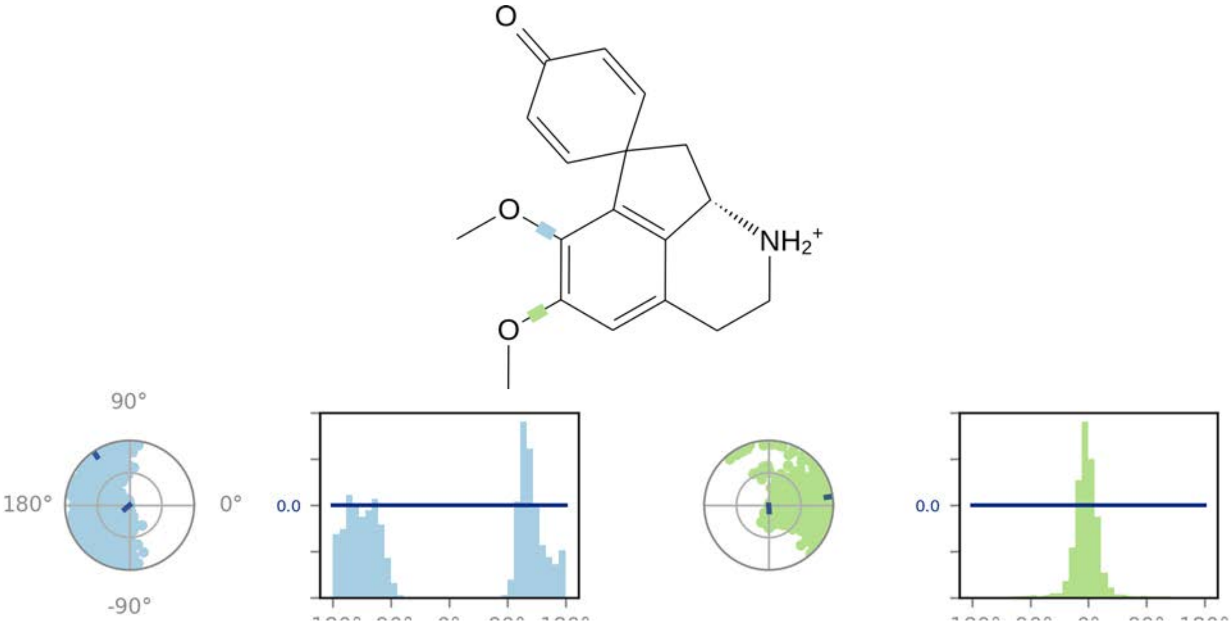

The ligand torsions plot summarizes the conformational evolution of every rotatable bond (RB) in the ligand throughout the simulation trajectory (0.00 through 10.00 nsec). The top panel shows the 2d schematic of a ligand with color-coded rotatable bonds. Each rotatable bond torsion is accompanied by a dial plot and bar plots of the same color.

Dial (or radial) plots describe the conformation of the torsion throughout the course of the simulation. The beginning of the simulation is in the center of the radial plot and the time evolution is plotted radially outwards.

The bar plots summarize the data on the dial plots, by showing the probability density of the torsion. If torsional potential information is available, the plot also shows the potential of the rotatable bond (by summing the potential of the related torsions). The values of the potential are on the left *Y*-axis of the chart, and are expressed in *kcal/mol*. Looking at the histogram and torsion potential relationships may give insights into the conformational strain the ligand undergoes to maintain a protein-bound conformation.

Ligand Properties

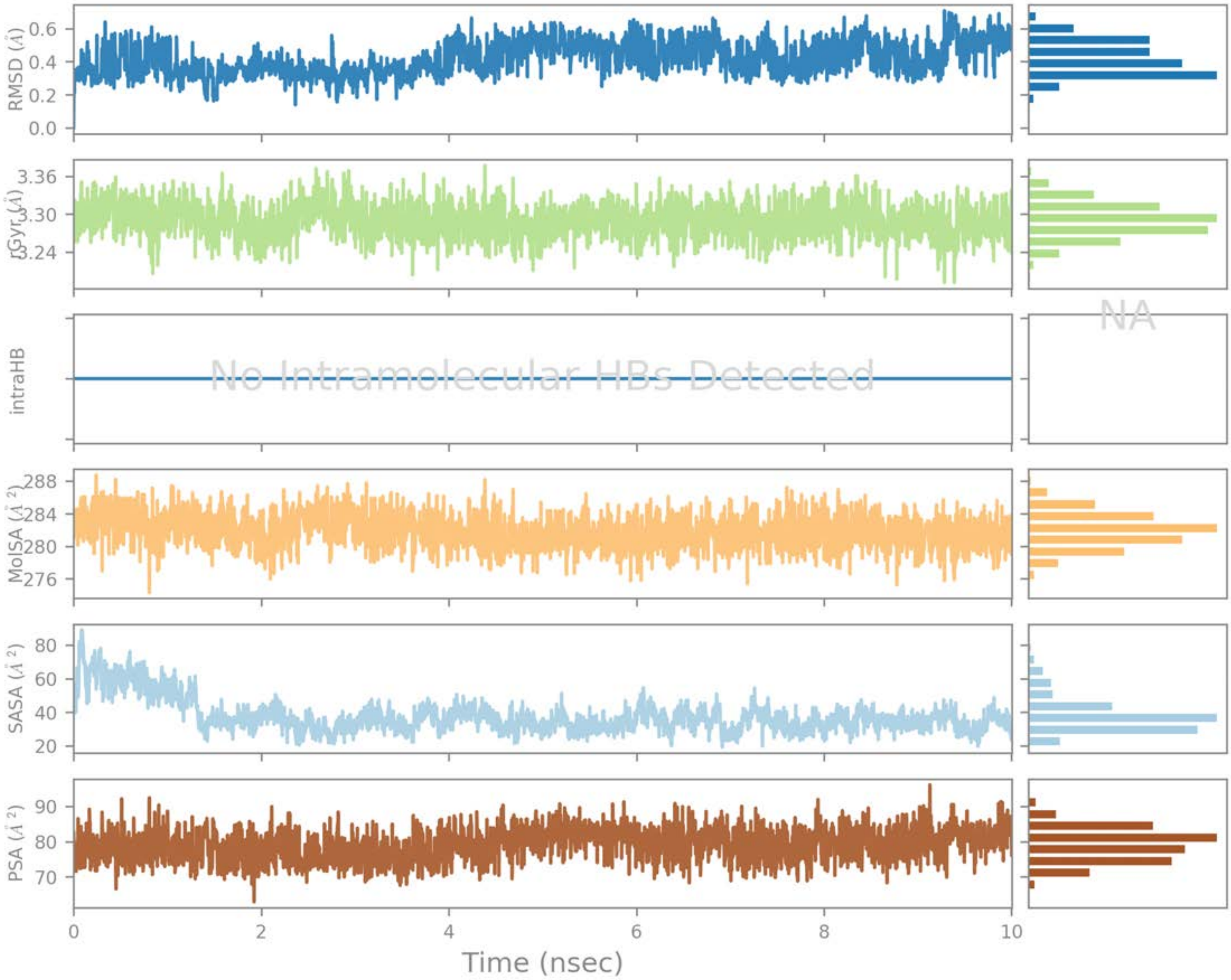

Ligand RMSD: Root mean square deviation of a ligand with respect to the reference conformation (typically the first frame is used as the reference and it is regarded as time *t*=0).

Radius of Gyration (rGyr): Measures the 'extendedness' of a ligand, and is equivalent to its principal moment of inertia.

Intramolecular Hydrogen Bonds (intraHB): Number of internal hydrogen bonds (HB) within a ligand molecule.

Molecular Surface Area (MolSA): Molecular surface calculation with 1.4 Å probe radius. This value is equivalent to a van der Waals surface area.

Solvent Accessible Surface Area (SASA): Surface area of a molecule accessible by a water molecule.

Polar Surface Area (PSA): Solvent accessible surface area in a molecule contributed only by oxygen and nitrogen atoms.

Report S4
MD Simulation Report on AChE - Palmatine Interactions

Simulation Details

Jobname: md_job_6H12_3-dock-2
Entry title: 3-dock-2

CPU #	Job Type	Ensemble	Temp. [K]	Sim. Time [ns]	# Atoms	# Waters	Charge
1	mdsim	NPT	300.0	10.005	62805	17950	0

Protein Information

Tot. Residues	Prot. Chain(s)	Res. in Chain(s)	# Atoms	# Heavy Atoms	Charge
562	'NoChainId'	ict_values([562]	8798	4471	-10

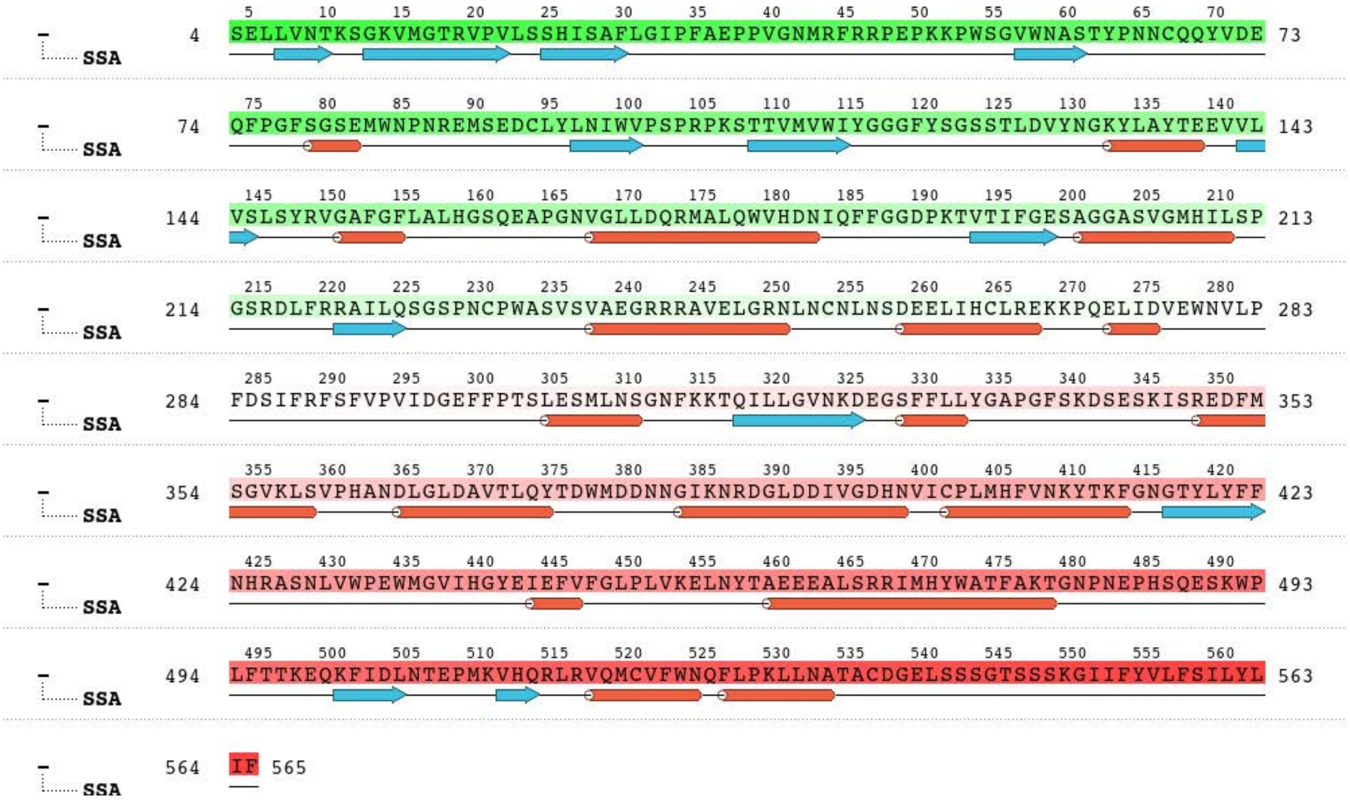

Ligand Information

SMILES	COc1c(OC)ccc(c12)cc3c4c(CC[n+]3c2)cc(OC)c(c4)OC
PDB Name	'UNK'
Num. of Atoms	48 (total) 26 (heavy)
Atomic Mass	352.414 au
Charge	+1
Mol. Formula	C21H22NO4
Num. of Fragments	1
Num. of Rot. Bonds	4

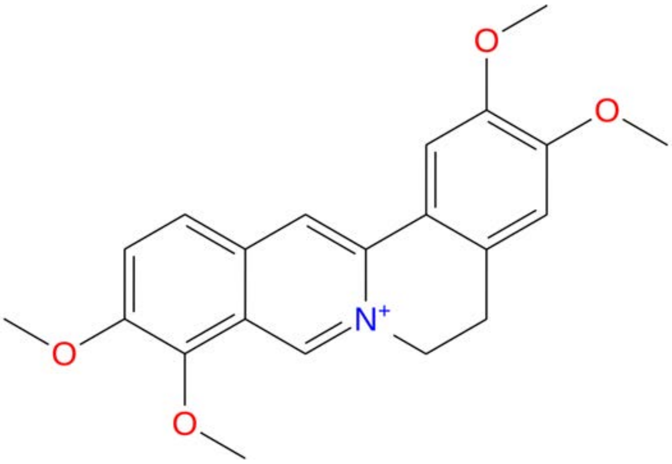

Counter Ion/Salt Information

Type	Num.	Concentration [mM]	Total Charge
Na	59	59.762	+59
Cl	50	50.646	-50

Protein-Ligand RMSD

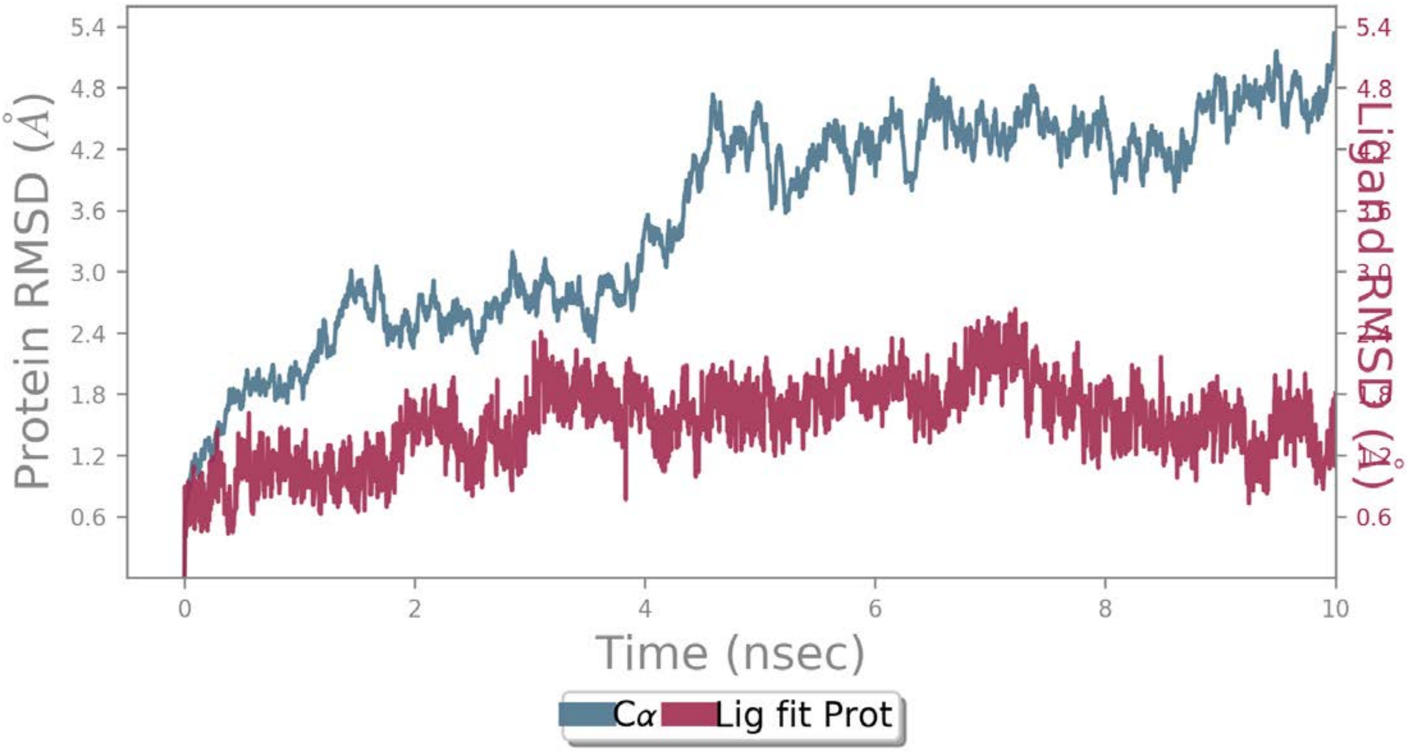

The Root Mean Square Deviation (RMSD) is used to measure the average change in displacement of a selection of atoms for a particular frame with respect to a reference frame. It is calculated for all frames in the trajectory. The RMSD for frame *x* is:

$$RMSD_x = \sqrt{\frac{1}{N}\sum_{i=1}^{N}(r'_i(t_x)) - r_i(t_{ref}))^2}$$

where *N* is the number of atoms in the atom selection; t_{ref} is the reference time, (typically the first frame is used as the reference and it is regarded as time *t*=0); and *r'* is the position of the selected atoms in frame *x* after superimposing on the reference frame, where frame *x* is recorded at time t_x. The procedure is repeated for every frame in the simulation trajectory.

Protein RMSD: The above plot shows the RMSD evolution of a protein (left *Y*-axis). All protein frames are first aligned on the reference frame backbone, and then the RMSD is calculated based on the atom selection. Monitoring the RMSD of the protein can give insights into its structural conformation throughout the simulation. RMSD analysis can indicate if the simulation has equilibrated — its fluctuations towards the end of the simulation are around some thermal average structure. Changes of the order of 1-3 Å are perfectly acceptable for small, globular proteins. Changes much larger than that, however, indicate that the protein is undergoing a large conformational change during the simulation. It is also important that your simulation converges — the RMSD values stabilize around a fixed value. If the RMSD of the protein is still increasing or decreasing on average at the end of the simulation, then your system has not equilibrated, and your simulation may not be long enough for rigorous analysis.

Ligand RMSD: Ligand RMSD (right *Y*-axis) indicates how stable the ligand is with respect to the protein and its binding pocket. In the above plot, 'Lig fit Prot' shows the RMSD of a ligand when the protein-ligand complex is first aligned on the protein backbone of the reference and then the RMSD of the ligand heavy atoms is measured. If the values observed are significantly larger than the RMSD of the protein, then it is likely that the ligand has diffused away from its initial binding site.

Protein RMSF

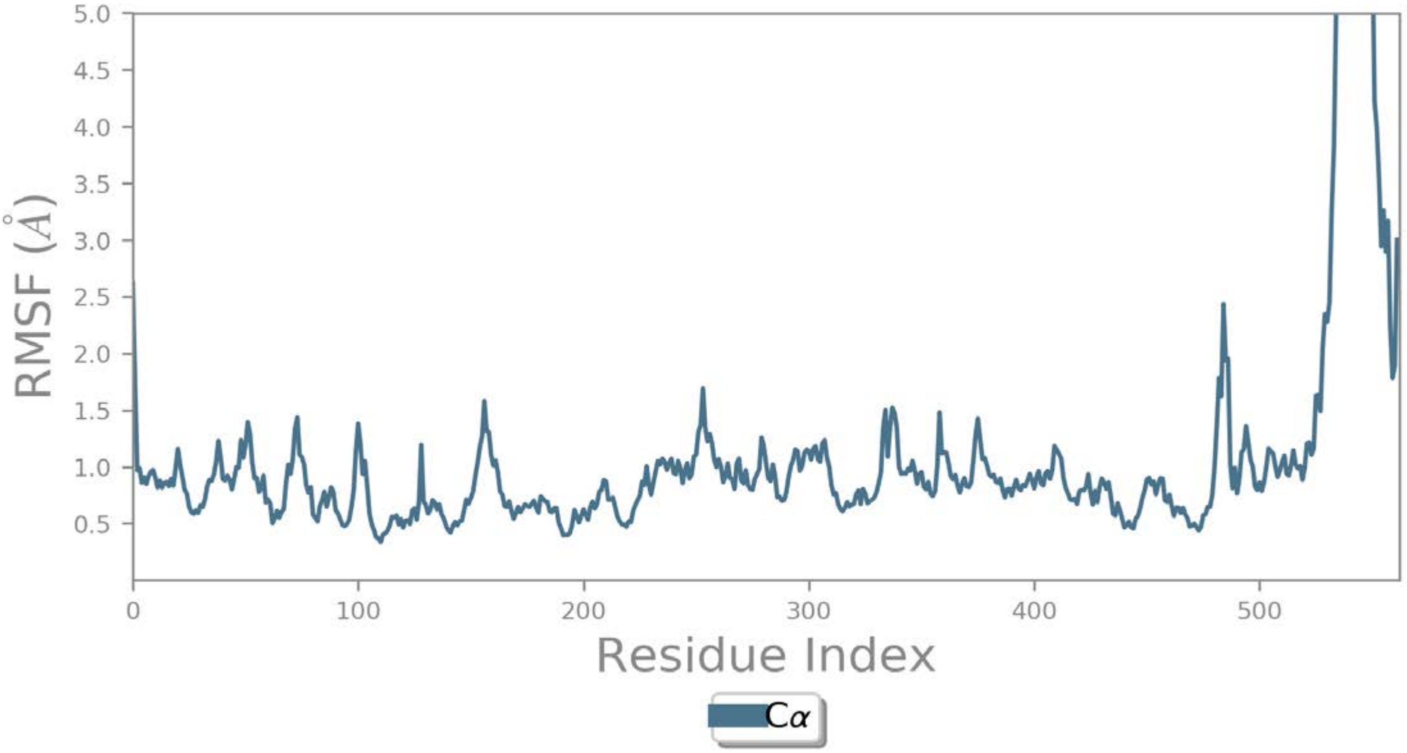

The Root Mean Square Fluctuation (RMSF) is useful for characterizing local changes along the protein chain. The RMSF for residue *i* is:

$$RMSF_i = \sqrt{\frac{1}{T}\sum_{t=1}^{T} < (r_i'(t)) - r_i(t_{ref}))^2 >}$$

where T is the trajectory time over which the RMSF is calculated, t_{ref} is the reference time, r_i is the position of residue *i*; *r'* is the position of atoms in residue *i* after superposition on the reference, and the angle brackets indicate that the average of the square distance is taken over the selection of atoms in the residue.

On this plot, peaks indicate areas of the protein that fluctuate the most during the simulation. Typically you will observe that the tails (*N*- and *C*-terminal) fluctuate more than any other part of the protein. Secondary structure elements like alpha helices and beta strands are usually more rigid than the unstructured part of the protein, and thus fluctuate less than the loop regions.

SCHRÖDINGER®

Protein Secondary Structure

% Helix	% Strand	% Total SSE
29.83	12.65	42.48

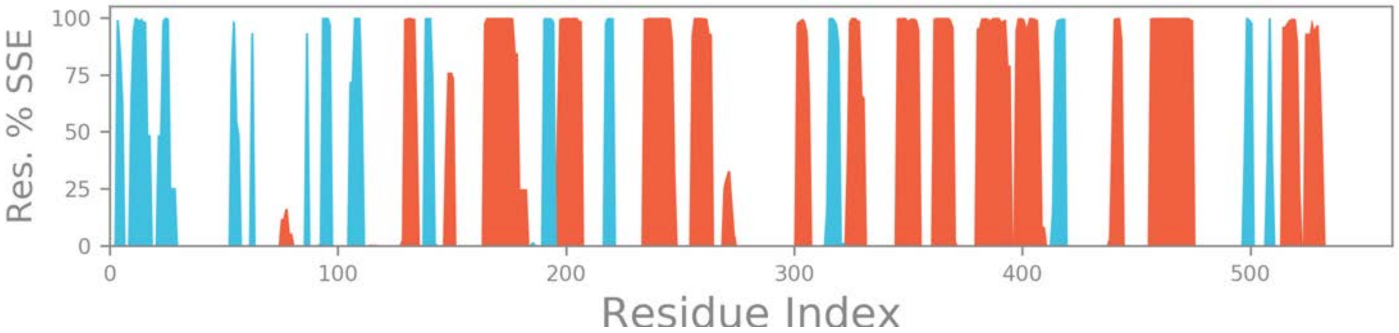

Protein secondary structure elements (SSE) like alpha-helices and beta-strands are monitored throughout the simulation. The plot above reports SSE distribution by residue index throughout the protein structure. The plot below summarizes the SSE composition for each trajectory frame over the course of the simulation, and the plot at the bottom monitors each residue and its SSE assignment over time.

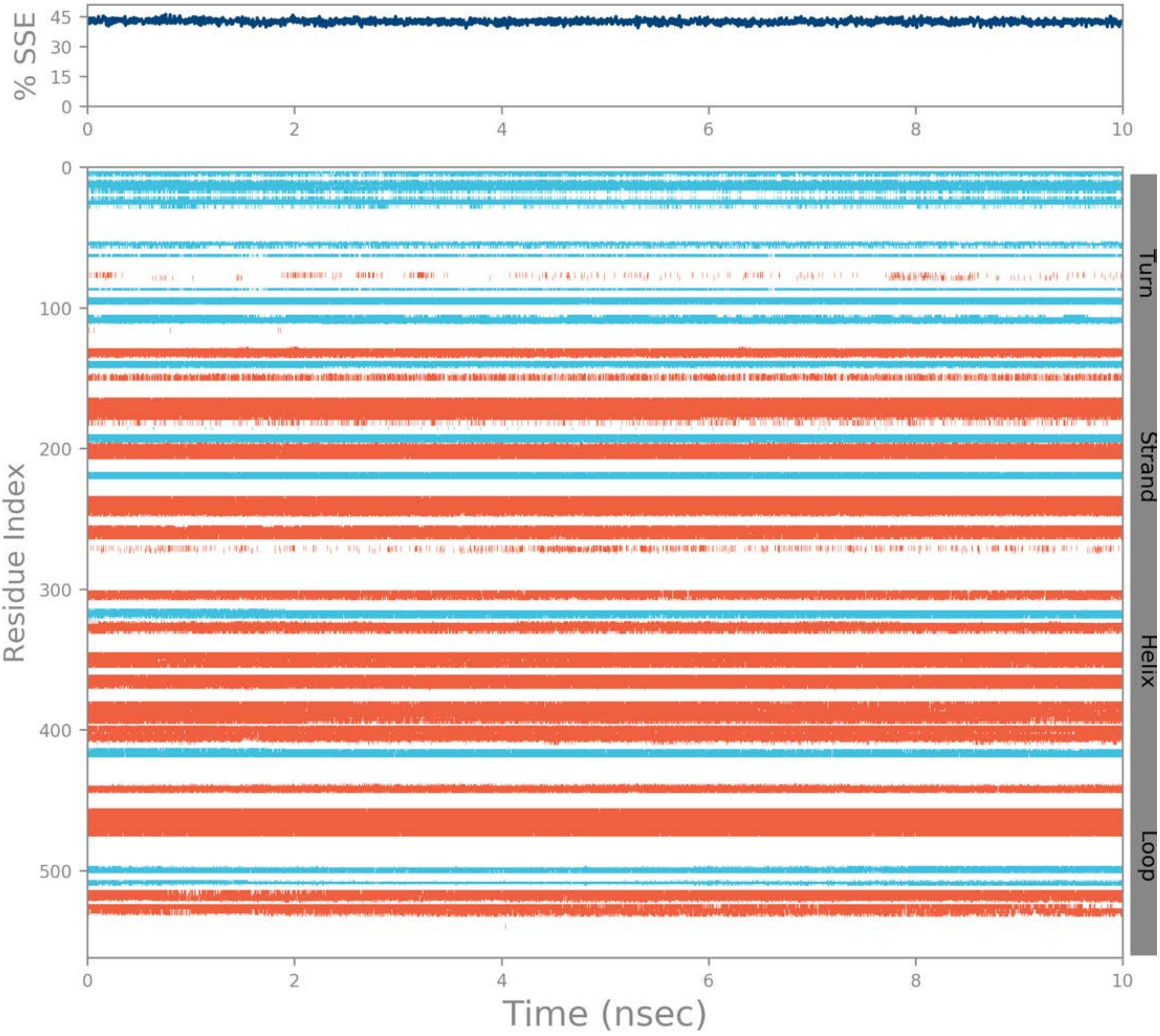

Palmatine Ligand RMSF

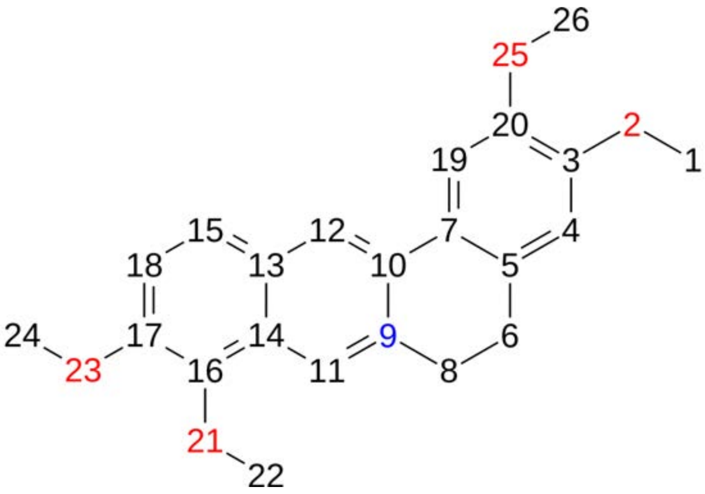

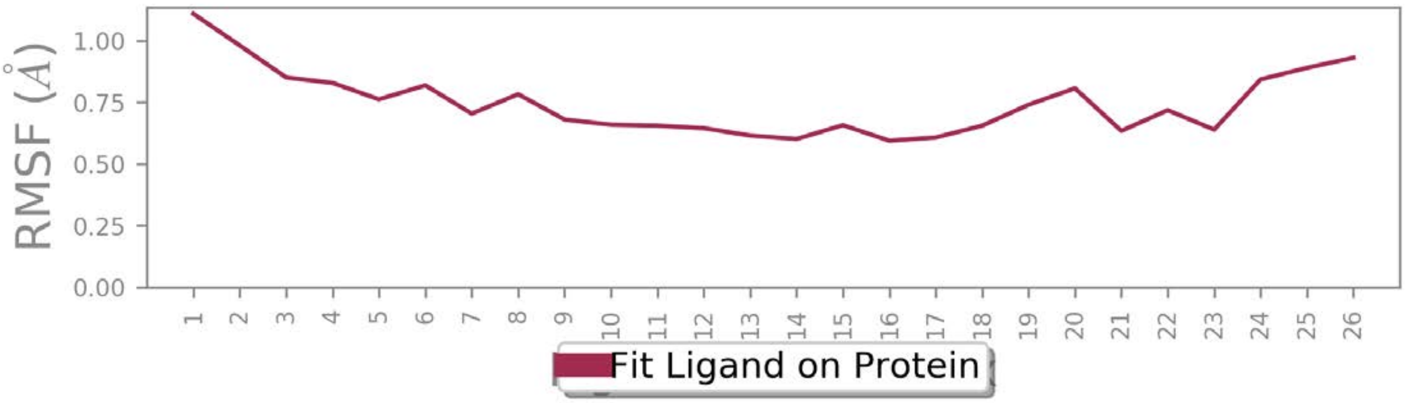

The Ligand Root Mean Square Fluctuation (L-RMSF) is useful for characterizing changes in the ligand atom positions. The RMSF for atom *i* is:

$$RMSF_i = \sqrt{\frac{1}{T}\sum_{t=1}^{T}(r'_i(t)) - r_i(t_{ref}))^2}$$

where *T* is the trajectory time over which the RMSF is calculated, t_{ref} is the reference time (usually for the first frame, and is regarded as the *zero* of time); *r* is the position of atom *i* in the reference at time t_{ref}, and *r'* is the position of atom *i* at time *t* after superposition on the reference frame.

Ligand RMSF shows the ligand's fluctuations broken down by atom, corresponding to the 2D structure in the top panel. The ligand RMSF may give you insights on how ligand fragments interact with the protein and their entropic role in the binding event. In the bottom panel, the 'Fit Ligand on Protein' line shows the ligand fluctuations, with respect to the protein. The protein-ligand complex is first aligned on the protein backbone and then the ligand RMSF is measured on the ligand heavy atoms.

Protein-Ligand Contacts

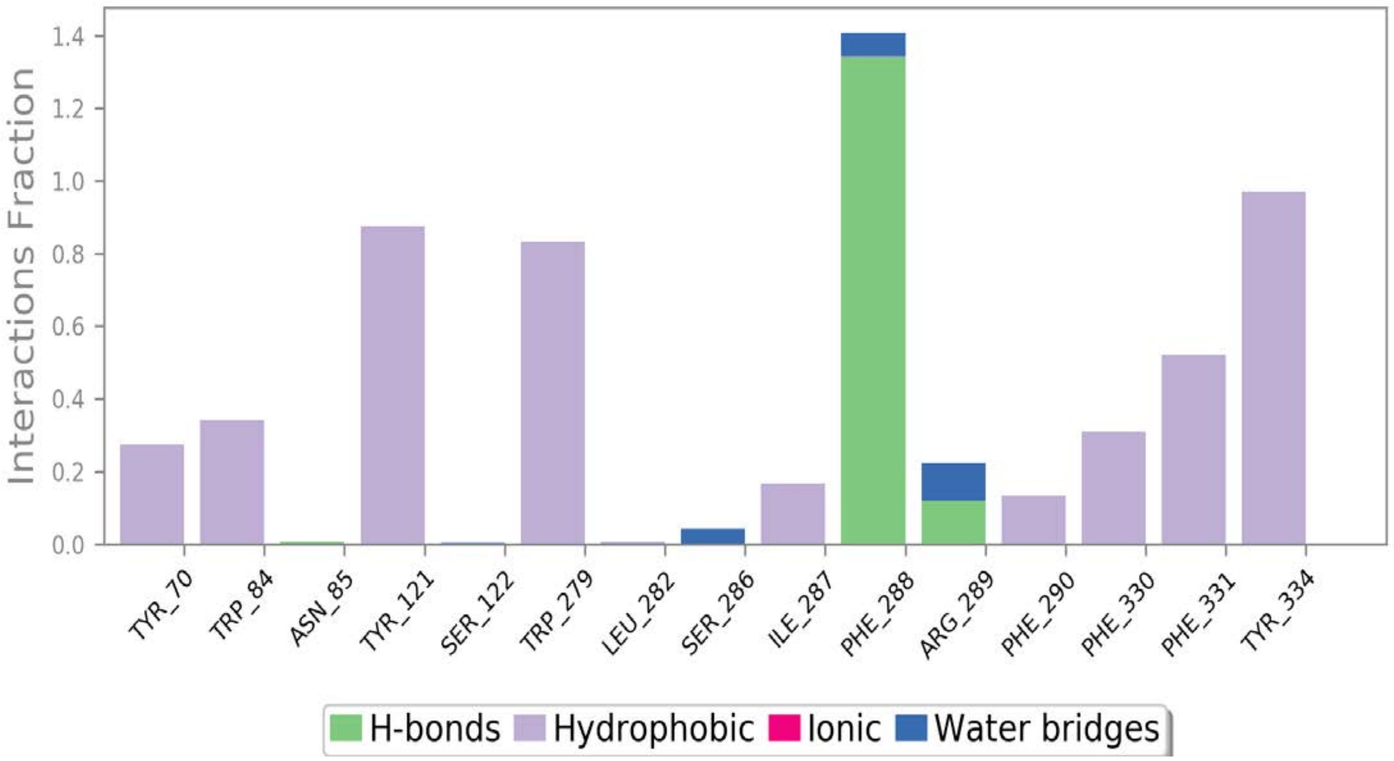

Protein interactions with the ligand can be monitored throughout the simulation. These interactions can be categorized by type and summarized, as shown in the plot above. Protein-ligand interactions (or 'contacts') are categorized into four types: Hydrogen Bonds, Hydrophobic, Ionic and Water Bridges. Each interaction type contains more specific subtypes, which can be explored through the 'Simulation Interactions Diagram' panel. The stacked bar charts are normalized over the course of the trajectory: for example, a value of 0.7 suggests that 70% of the simulation time the specific interaction is maintained. Values over 1.0 are possible as some protein residue may make multiple contacts of same subtype with the ligand.

Hydrogen Bonds: (H-bonds) play a significant role in ligand binding. Consideration of hydrogen-bonding properties in drug design is important because of their strong influence on drug specificity, metabolization and adsorption. Hydrogen bonds between a protein and a ligand can be further broken down into four subtypes: backbone acceptor; backbone donor; side-chain acceptor; side-chain donor.
The current geometric criteria for protein-ligand H-bond is: distance of 2.5 Å between the donor and acceptor atoms (D—H···A); a donor angle of ≥120° between the donor-hydrogen-acceptor atoms (D—H···A); and an acceptor angle of ≥90° between the hydrogen-acceptor-bonded_atom atoms (H···A—X).

Hydrophobic contacts: fall into three subtypes: π-Cation; π-π; and Other, non-specific interactions. Generally these type of interactions involve a hydrophobic amino acid and an aromatic or aliphatic group on the ligand, but we have extended this category to also include π-Cation interactions.
The current geometric criteria for hydrophobic interactions is as follows: π-Cation — Aromatic and charged groups within 4.5 Å; π-π — Two aromatic groups stacked face-to-face or face-to-edge; Other — A non-specific hydrophobic sidechain within 3.6 Å of a ligand's aromatic or aliphatic carbons.

Ionic interactions: or polar interactions, are between two oppositely charged atoms that are within 3.7 Å of each other and do not involve a hydrogen bond. We also monitor Protein-Metal-Ligand interactions, which are defined by a metal ion coordinated within 3.4 Å of protein's and ligand's heavy atoms (except carbon). All ionic interactions are broken down into two subtypes: those mediated by a protein backbone or side chains.

Water Bridges: are hydrogen-bonded protein-ligand interactions mediated by a water molecule. The hydrogen-bond geometry is slightly relaxed from the standard H-bond definition.
The current geometric criteria for a protein-water or water-ligand H-bond are: a distance of 2.8 Å between the donor and acceptor atoms (D—H···A); a donor angle of ≥110° between the donor-hydrogen-acceptor atoms (D—H···A); and an acceptor angle of ≥90° between the hydrogen-acceptor-bonded_atom atoms (H···A—X).

Protein-Ligand Contacts (cont.)

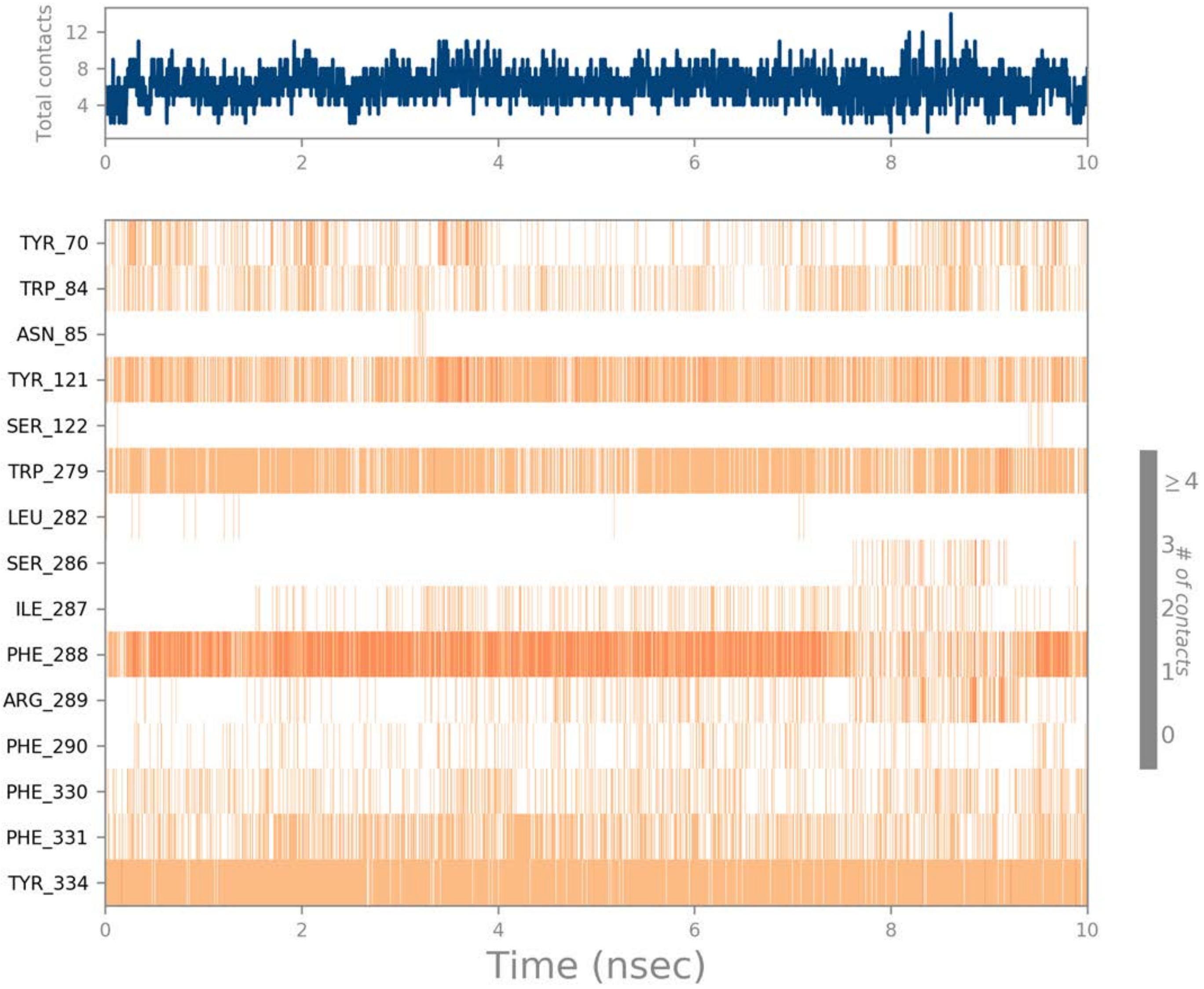

A timeline representation of the interactions and contacts (**H-bonds**, **Hydrophobic**, **Ionic**, **Water bridges**) summarized in the previous page. The top panel shows the total number of specific contacts the protein makes with the ligand over the course of the trajectory. The bottom panel shows which residues interact with the ligand in each trajectory frame. Some residues make more than one specific contact with the ligand, which is represented by a darker shade of orange, according to the scale to the right of the plot.

Ligand-Protein Contacts

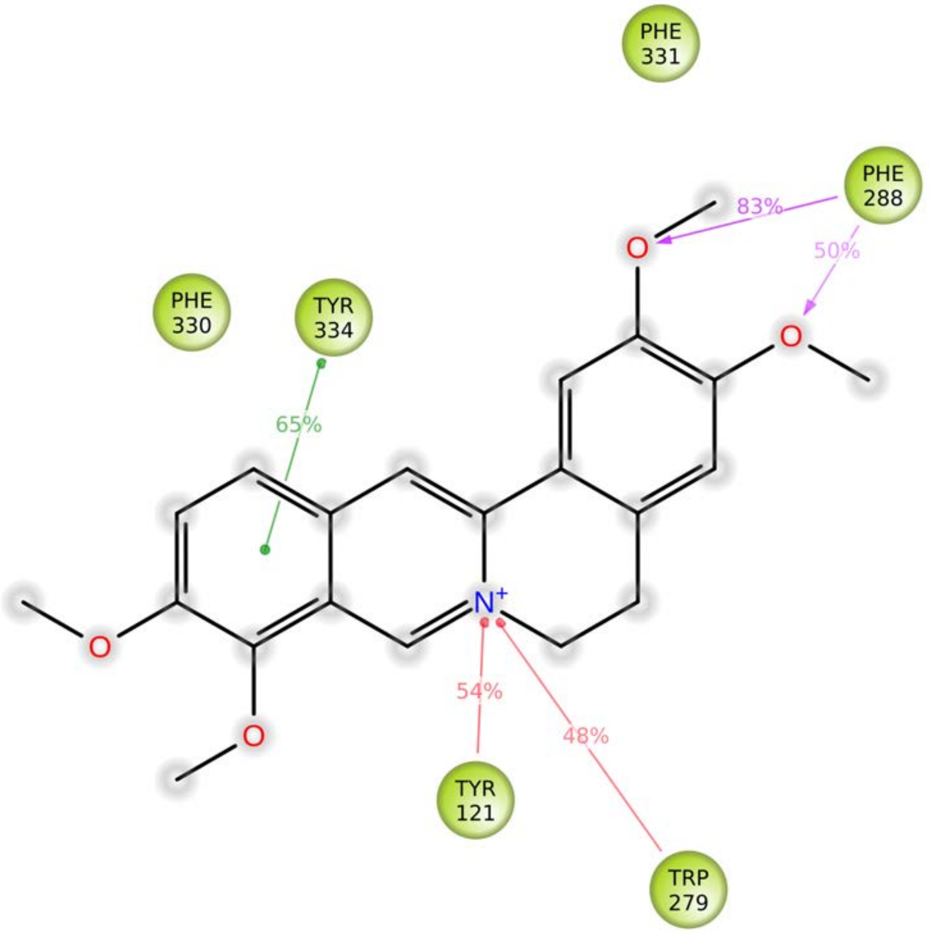

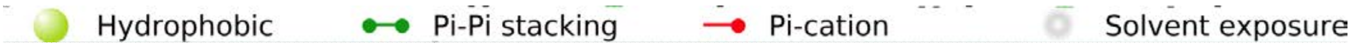

A schematic of detailed ligand atom interactions with the protein residues. Interactions that occur more than **30.0%** of the simulation time in the selected trajectory (0.00 through 10.00 nsec), are shown.
Note: it is possible to have interactions with >100% as some residues may have multiple interactions of a single type with the same ligand atom. For example, the ARG side chain has four H-bond donors that can all hydrogen-bond to a single H-bond acceptor.

Ligand Torsion Profile

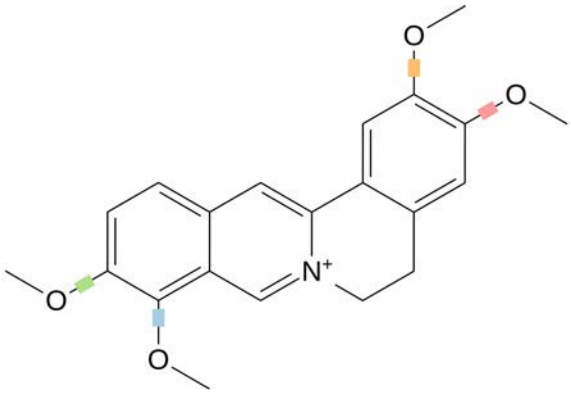

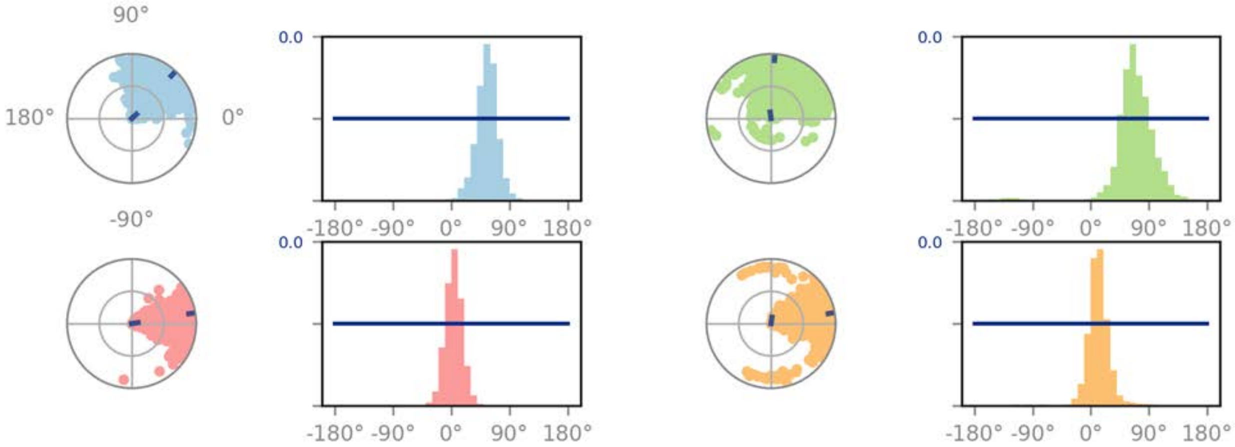

The ligand torsions plot summarizes the conformational evolution of every rotatable bond (RB) in the ligand throughout the simulation trajectory (0.00 through 10.00 nsec). The top panel shows the 2d schematic of a ligand with color-coded rotatable bonds. Each rotatable bond torsion is accompanied by a dial plot and bar plots of the same color.

Dial (or radial) plots describe the conformation of the torsion throughout the course of the simulation. The beginning of the simulation is in the center of the radial plot and the time evolution is plotted radially outwards.

The bar plots summarize the data on the dial plots, by showing the probability density of the torsion. If torsional potential information is available, the plot also shows the potential of the rotatable bond (by summing the potential of the related torsions). The values of the potential are on the left *Y*-axis of the chart, and are expressed in *kcal/mol*. Looking at the histogram and torsion potential relationships may give insights into the conformational strain the ligand undergoes to maintain a protein-bound conformation.

Ligand Properties

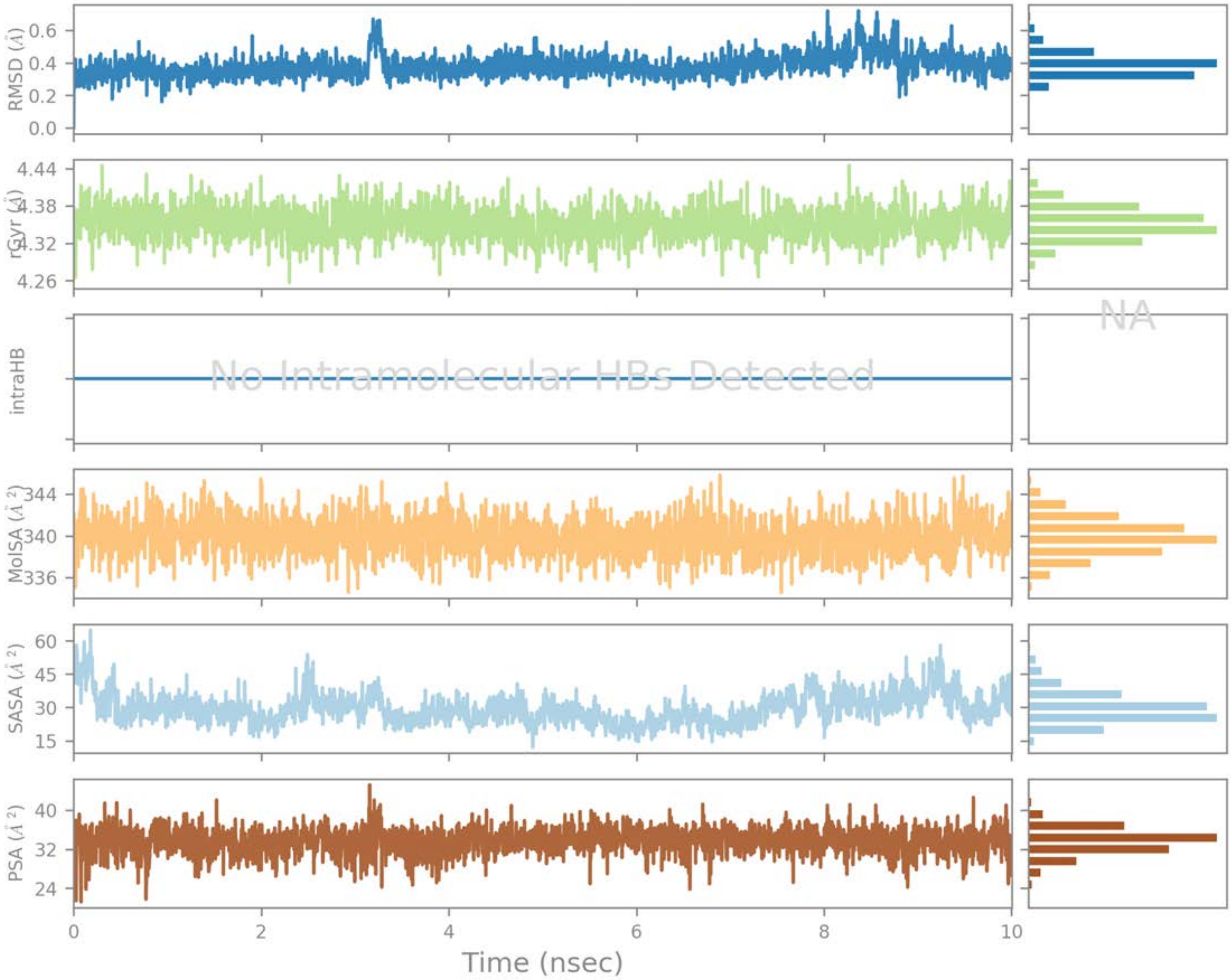

Ligand RMSD: Root mean square deviation of a ligand with respect to the reference conformation (typically the first frame is used as the reference and it is regarded as time *t*=0).

Radius of Gyration (rGyr): Measures the 'extendedness' of a ligand, and is equivalent to its principal moment of inertia.

Intramolecular Hydrogen Bonds (intraHB): Number of internal hydrogen bonds (HB) within a ligand molecule.

Molecular Surface Area (MolSA): Molecular surface calculation with 1.4 Å probe radius. This value is equivalent to a van der Waals surface area.

Solvent Accessible Surface Area (SASA): Surface area of a molecule accessible by a water molecule.

Polar Surface Area (PSA): Solvent accessible surface area in a molecule contributed only by oxygen and nitrogen atoms.

Report S5

MD Simulation Report on AChE - 5-*N*-Methylmaytenine Interactions

Simulation Details

Jobname: md_job_6H12_4-dock-1
Entry title: 4-dock-1

CPU #	Job Type	Ensemble	Temp. [K]	Sim. Time [ns]	# Atoms	# Waters	Charge
1	mdsim	NPT	300.0	10.005	62876	17968	0

Protein Information

Tot. Residues	Prot. Chain(s)	Res. in Chain(s)	# Atoms	# Heavy Atoms	Charge
562	'NoChainId'	ict_values([562]	8798	4471	-10

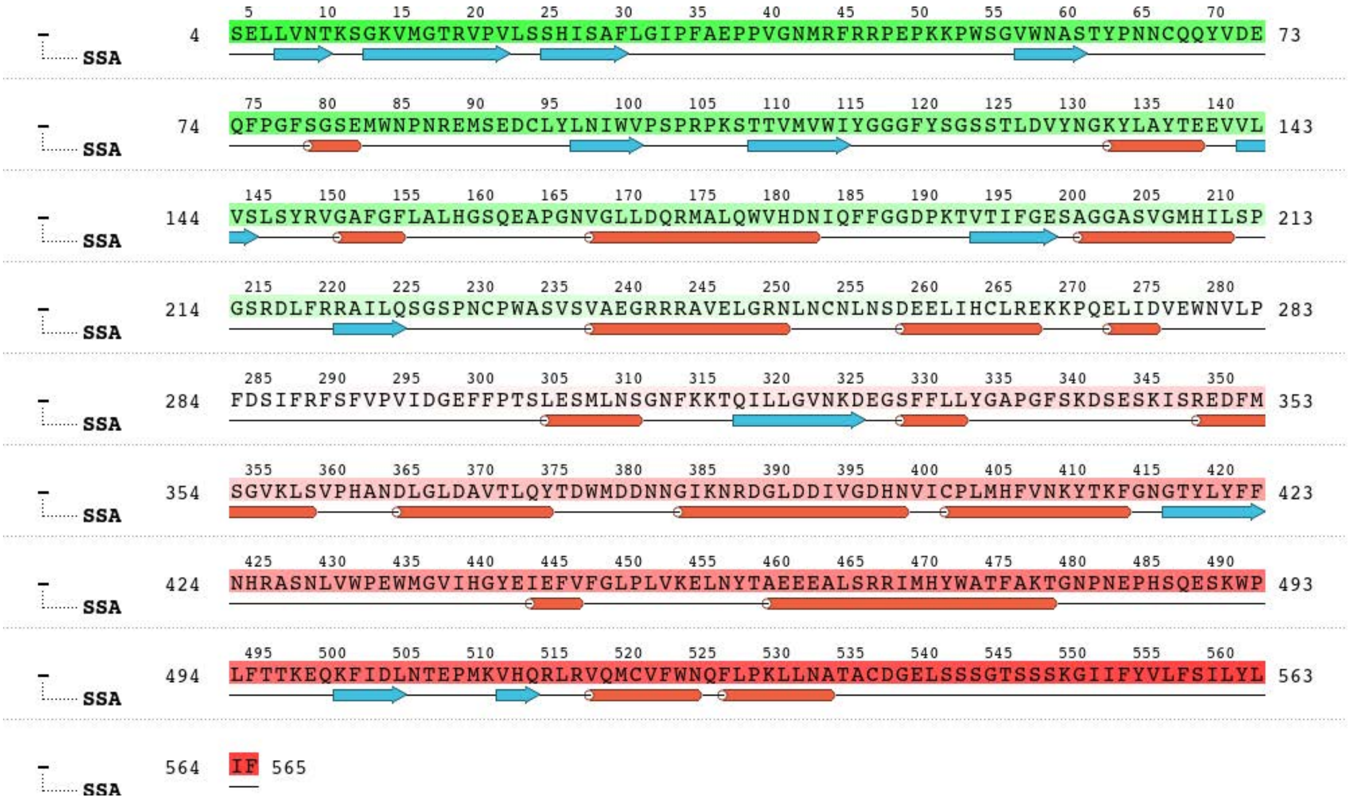

Ligand Information

SMILES	c1ccccc1\C=C\C(=O)NCCCC[N@H+](C)CCCNC(=O)/C=C/c2ccccc2
PDB Name	'UNK'
Num. of Atoms	65 (total) 31 (heavy)
Atomic Mass	420.580 au
Charge	+1
Mol. Formula	C26H34N3O2
Num. of Fragments	9
Num. of Rot. Bonds	15

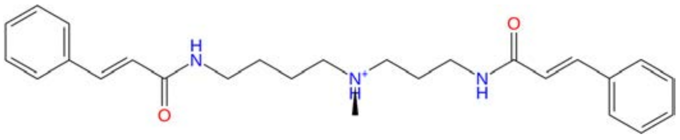

Counter Ion/Salt Information

Type	Num.	Concentration [mM]	Total Charge
Na	59	59.702	+59
Cl	50	50.595	-50

Protein-Ligand RMSD

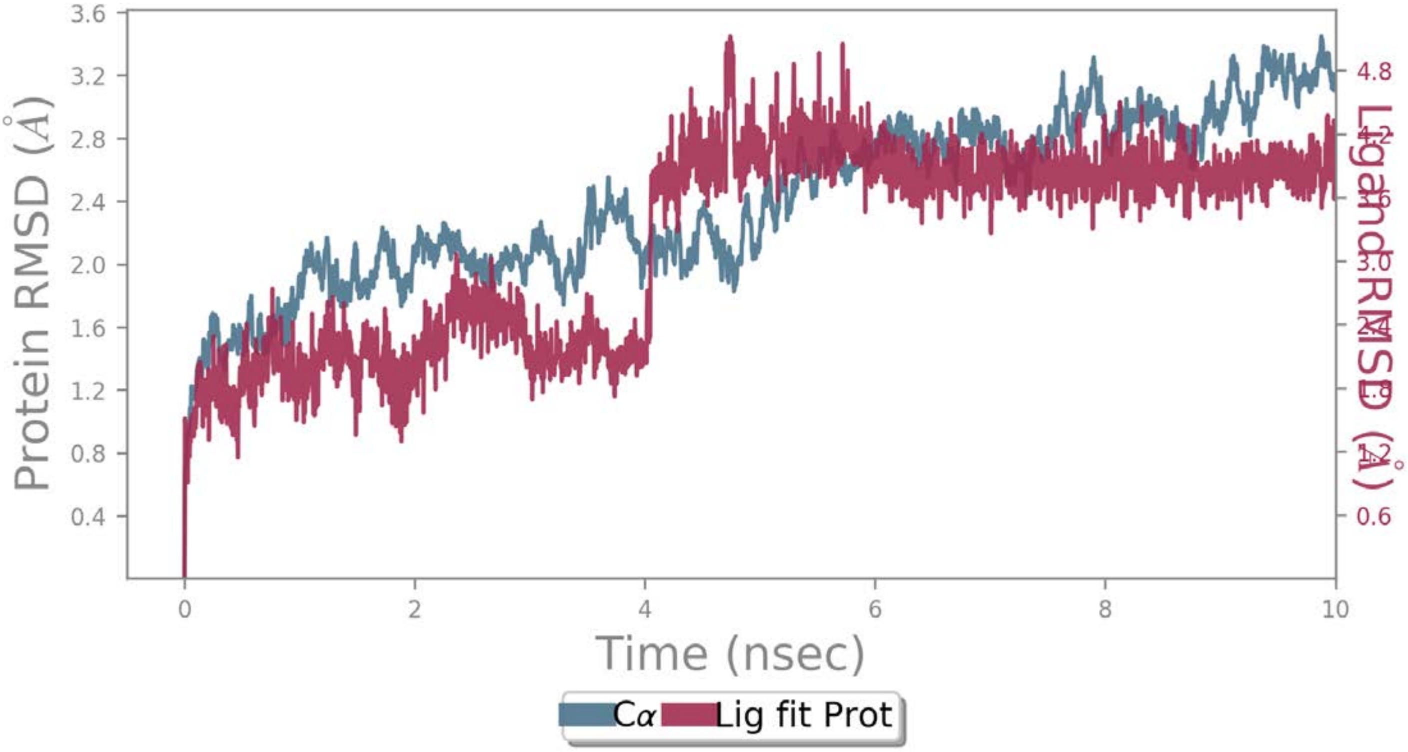

The Root Mean Square Deviation (RMSD) is used to measure the average change in displacement of a selection of atoms for a particular frame with respect to a reference frame. It is calculated for all frames in the trajectory. The RMSD for frame *x* is:

$$RMSD_x = \sqrt{\frac{1}{N}\sum_{i=1}^{N}(r'_i(t_x)) - r_i(t_{ref}))^2}$$

where *N* is the number of atoms in the atom selection; t_{ref} is the reference time, (typically the first frame is used as the reference and it is regarded as time *t*=0); and *r'* is the position of the selected atoms in frame *x* after superimposing on the reference frame, where frame *x* is recorded at time t_x. The procedure is repeated for every frame in the simulation trajectory.

Protein RMSD: The above plot shows the RMSD evolution of a protein (left *Y*-axis). All protein frames are first aligned on the reference frame backbone, and then the RMSD is calculated based on the atom selection. Monitoring the RMSD of the protein can give insights into its structural conformation throughout the simulation. RMSD analysis can indicate if the simulation has equilibrated — its fluctuations towards the end of the simulation are around some thermal average structure. Changes of the order of 1-3 Å are perfectly acceptable for small, globular proteins. Changes much larger than that, however, indicate that the protein is undergoing a large conformational change during the simulation. It is also important that your simulation converges — the RMSD values stabilize around a fixed value. If the RMSD of the protein is still increasing or decreasing on average at the end of the simulation, then your system has not equilibrated, and your simulation may not be long enough for rigorous analysis.

Ligand RMSD: Ligand RMSD (right *Y*-axis) indicates how stable the ligand is with respect to the protein and its binding pocket. In the above plot, 'Lig fit Prot' shows the RMSD of a ligand when the protein-ligand complex is first aligned on the protein backbone of the reference and then the RMSD of the ligand heavy atoms is measured. If the values observed are significantly larger than the RMSD of the protein, then it is likely that the ligand has diffused away from its initial binding site.

Protein RMSF

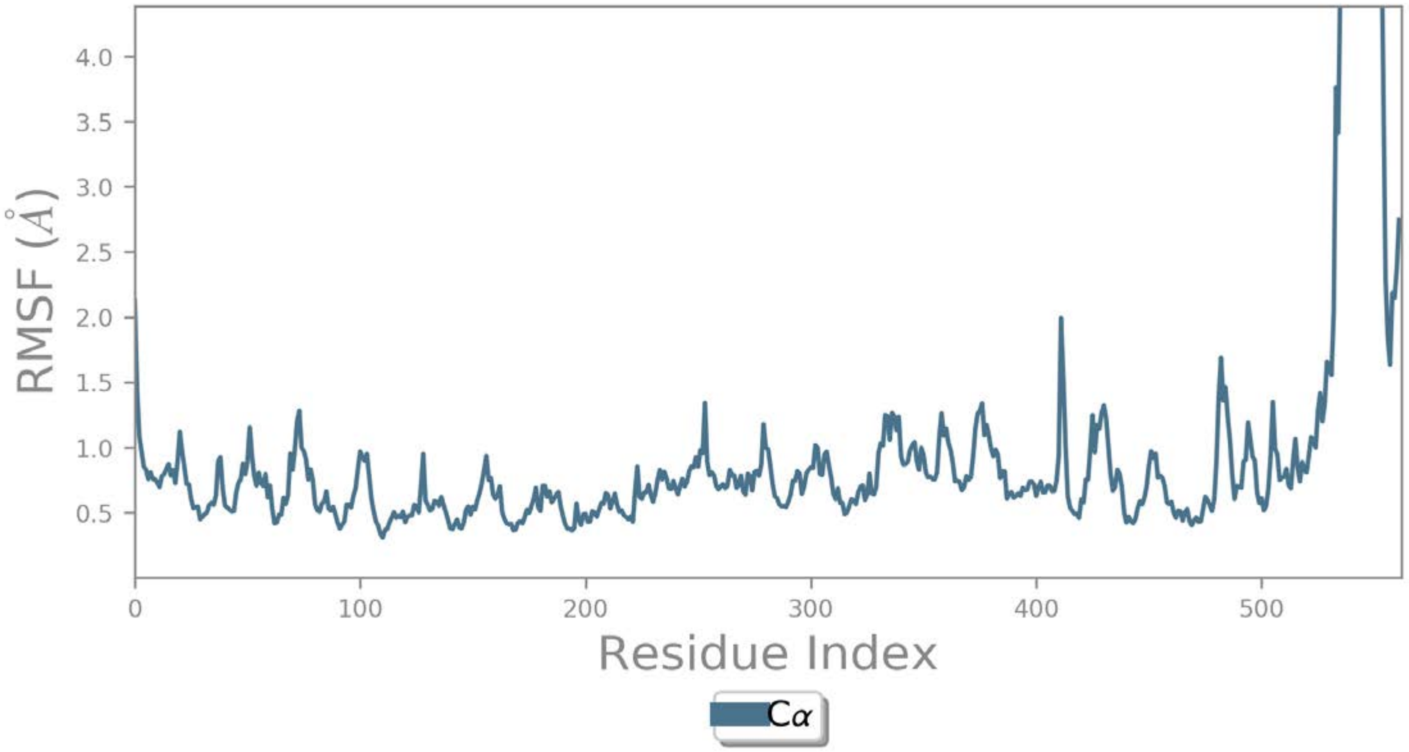

The Root Mean Square Fluctuation (RMSF) is useful for characterizing local changes along the protein chain. The RMSF for residue *i* is:

$$RMSF_i = \sqrt{\frac{1}{T}\sum_{t=1}^{T} < (r_i'(t)) - r_i(t_{ref}))^2 >}$$

where T is the trajectory time over which the RMSF is calculated, t_{ref} is the reference time, r_i is the position of residue i; r' is the position of atoms in residue i after superposition on the reference, and the angle brackets indicate that the average of the square distance is taken over the selection of atoms in the residue.

On this plot, peaks indicate areas of the protein that fluctuate the most during the simulation. Typically you will observe that the tails (*N*- and *C*-terminal) fluctuate more than any other part of the protein. Secondary structure elements like alpha helices and beta strands are usually more rigid than the unstructured part of the protein, and thus fluctuate less than the loop regions.

Protein Secondary Structure

% Helix	% Strand	% Total SSE
29.66	12.92	42.58

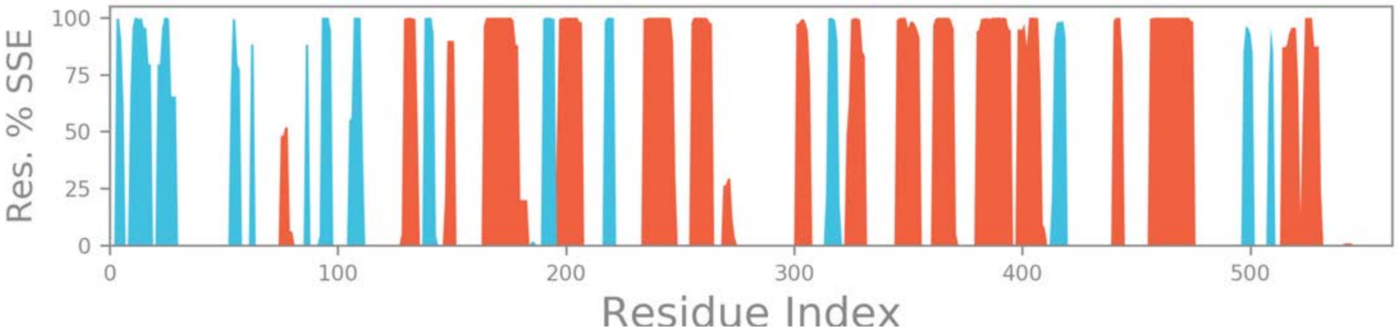

Protein secondary structure elements (SSE) like alpha-helices and beta-strands are monitored throughout the simulation. The plot above reports SSE distribution by residue index throughout the protein structure. The plot below summarizes the SSE composition for each trajectory frame over the course of the simulation, and the plot at the bottom monitors each residue and its SSE assignment over time.

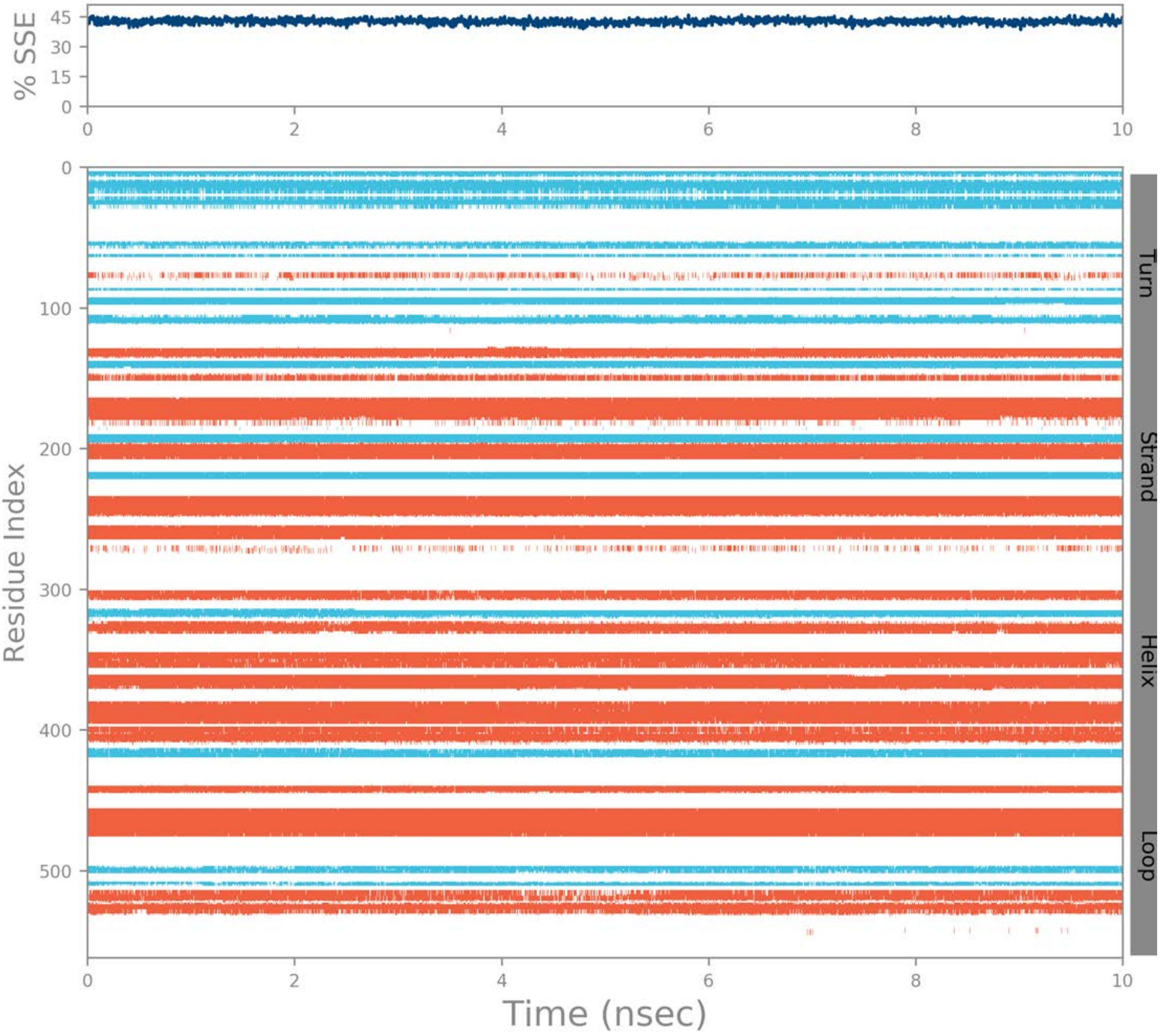

5-*N*-Methylmaitenine Ligand RMSF

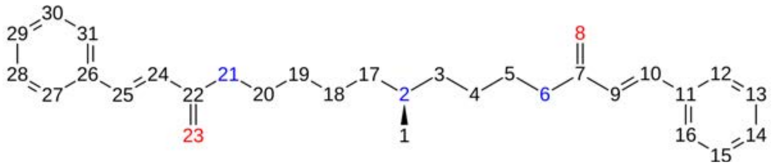

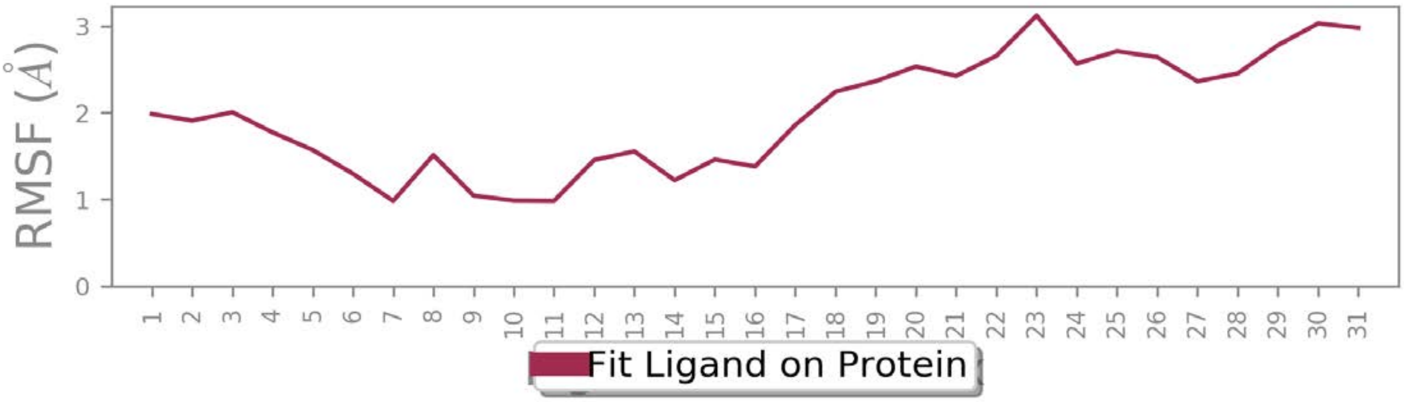

The Ligand Root Mean Square Fluctuation (L-RMSF) is useful for characterizing changes in the ligand atom positions. The RMSF for atom *i* is:

$$RMSF_i = \sqrt{\frac{1}{T}\sum_{t=1}^{T}(r'_i(t)) - r_i(t_{ref}))^2}$$

where T is the trajectory time over which the RMSF is calculated, t_{ref} is the reference time (usually for the first frame, and is regarded as the *zero* of time); r is the position of atom i in the reference at time t_{ref}, and r' is the position of atom i at time t after superposition on the reference frame.

Ligand RMSF shows the ligand's fluctuations broken down by atom, corresponding to the 2D structure in the top panel. The ligand RMSF may give you insights on how ligand fragments interact with the protein and their entropic role in the binding event. In the bottom panel, the 'Fit Ligand on Protein' line shows the ligand fluctuations, with respect to the protein. The protein-ligand complex is first aligned on the protein backbone and then the ligand RMSF is measured on the ligand heavy atoms.

Protein-Ligand Contacts

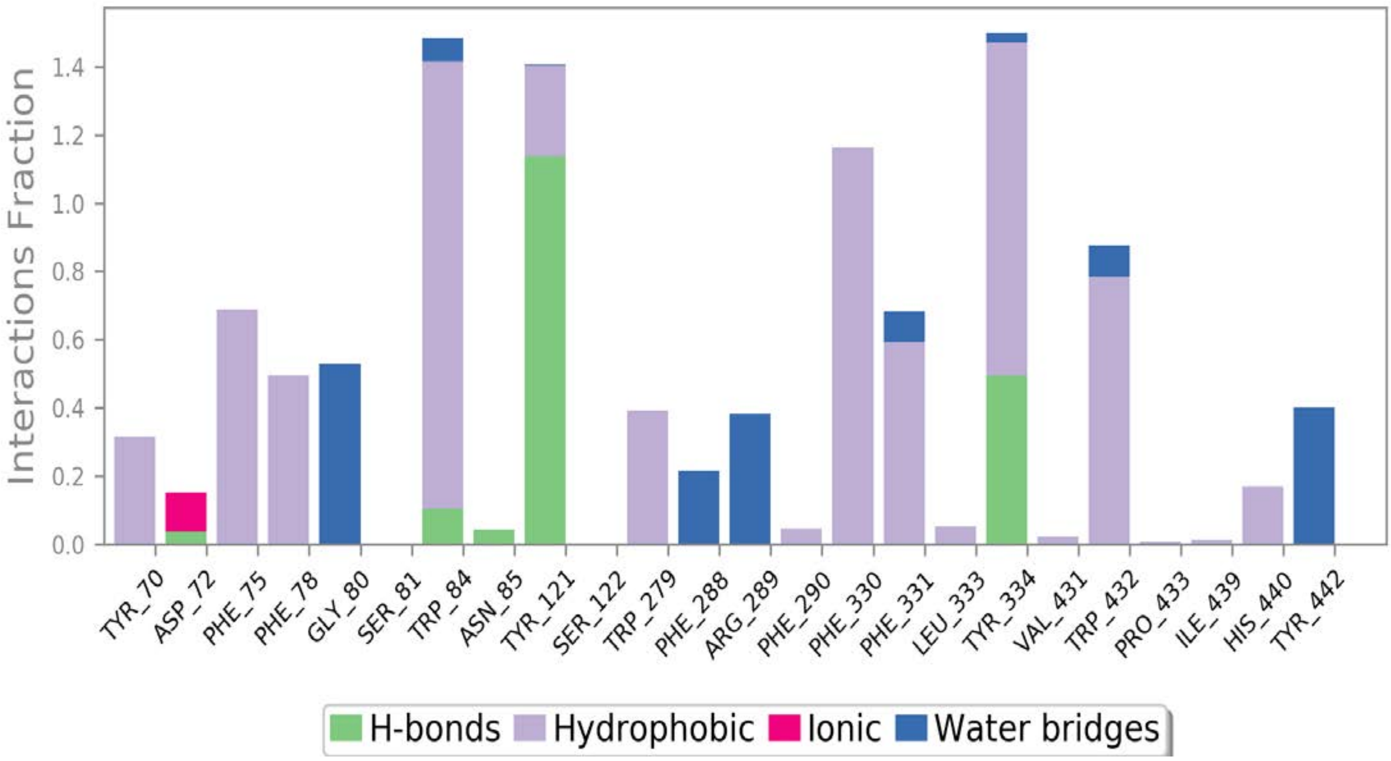

Protein interactions with the ligand can be monitored throughout the simulation. These interactions can be categorized by type and summarized, as shown in the plot above. Protein-ligand interactions (or 'contacts') are categorized into four types: Hydrogen Bonds, Hydrophobic, Ionic and Water Bridges. Each interaction type contains more specific subtypes, which can be explored through the 'Simulation Interactions Diagram' panel. The stacked bar charts are normalized over the course of the trajectory: for example, a value of 0.7 suggests that 70% of the simulation time the specific interaction is maintained. Values over 1.0 are possible as some protein residue may make multiple contacts of same subtype with the ligand.

Hydrogen Bonds: (H-bonds) play a significant role in ligand binding. Consideration of hydrogen-bonding properties in drug design is important because of their strong influence on drug specificity, metabolization and adsorption. Hydrogen bonds between a protein and a ligand can be further broken down into four subtypes: backbone acceptor; backbone donor; side-chain acceptor; side-chain donor.
The current geometric criteria for protein-ligand H-bond is: distance of 2.5 Å between the donor and acceptor atoms (D—H···A); a donor angle of ≥120° between the donor-hydrogen-acceptor atoms (D—H···A); and an acceptor angle of ≥90° between the hydrogen-acceptor-bonded_atom atoms (H···A—X).

Hydrophobic contacts: fall into three subtypes: π-Cation; π-π; and Other, non-specific interactions. Generally these type of interactions involve a hydrophobic amino acid and an aromatic or aliphatic group on the ligand, but we have extended this category to also include π-Cation interactions.
The current geometric criteria for hydrophobic interactions is as follows: π-Cation — Aromatic and charged groups within 4.5 Å; π-π — Two aromatic groups stacked face-to-face or face-to-edge; Other — A non-specific hydrophobic sidechain within 3.6 Å of a ligand's aromatic or aliphatic carbons.

Ionic interactions: or polar interactions, are between two oppositely charged atoms that are within 3.7 Å of each other and do not involve a hydrogen bond. We also monitor Protein-Metal-Ligand interactions, which are defined by a metal ion coordinated within 3.4 Å of protein's and ligand's heavy atoms (except carbon). All ionic interactions are broken down into two subtypes: those mediated by a protein backbone or side chains.

Water Bridges: are hydrogen-bonded protein-ligand interactions mediated by a water molecule. The hydrogen-bond geometry is slightly relaxed from the standard H-bond definition.
The current geometric criteria for a protein-water or water-ligand H-bond are: a distance of 2.8 Å between the donor and acceptor atoms (D—H···A); a donor angle of ≥110° between the donor-hydrogen-acceptor atoms (D—H···A); and an acceptor angle of ≥90° between the hydrogen-acceptor-bonded_atom atoms (H···A—X).

Protein-Ligand Contacts (cont.)

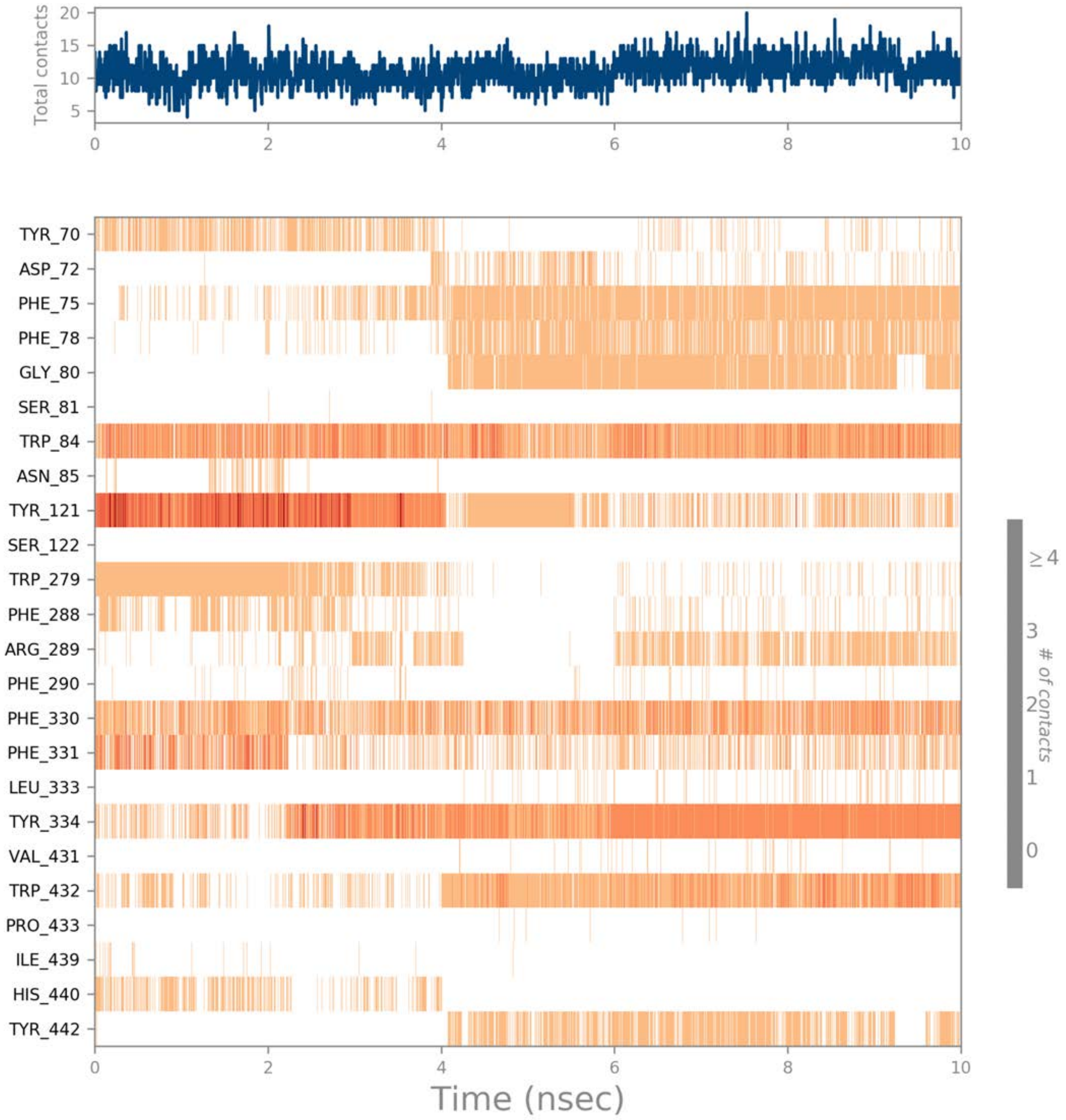

A timeline representation of the interactions and contacts (**H-bonds**, **Hydrophobic**, **Ionic**, **Water bridges**) summarized in the previous page. The top panel shows the total number of specific contacts the protein makes with the ligand over the course of the trajectory. The bottom panel shows which residues interact with the ligand in each trajectory frame. Some residues make more than one specific contact with the ligand, which is represented by a darker shade of orange, according to the scale to the right of the plot.

Ligand-Protein Contacts

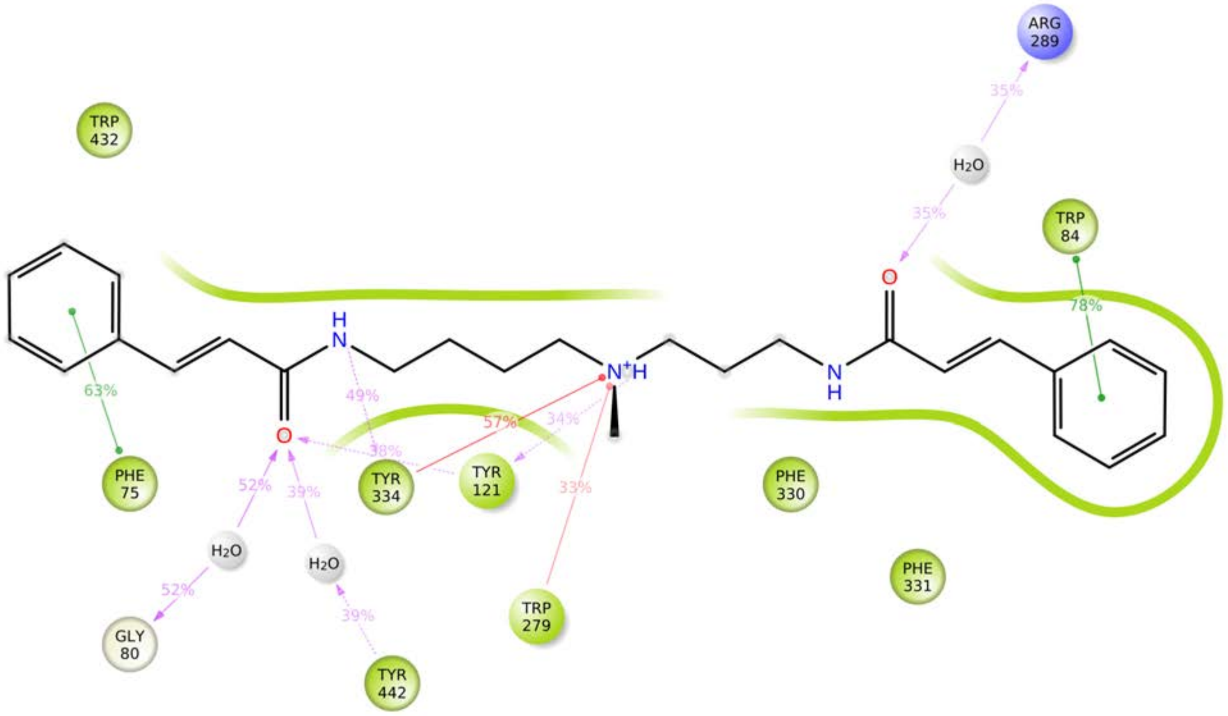

Charged (positive) | Hydrophobic | Pi-Pi stacking | Solvent exposure
Glycine | Water | Pi-cation

A schematic of detailed ligand atom interactions with the protein residues. Interactions that occur more than **30.0%** of the simulation time in the selected trajectory (0.00 through 10.00 nsec), are shown.
Note: it is possible to have interactions with >100% as some residues may have multiple interactions of a single type with the same ligand atom. For example, the ARG side chain has four H-bond donors that can all hydrogen-bond to a single H-bond acceptor.

Ligand Torsion Profile

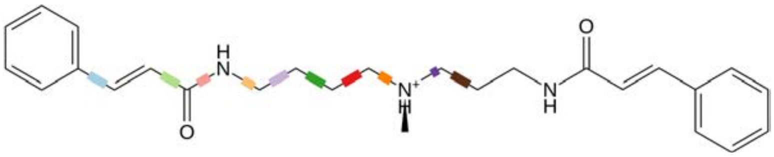

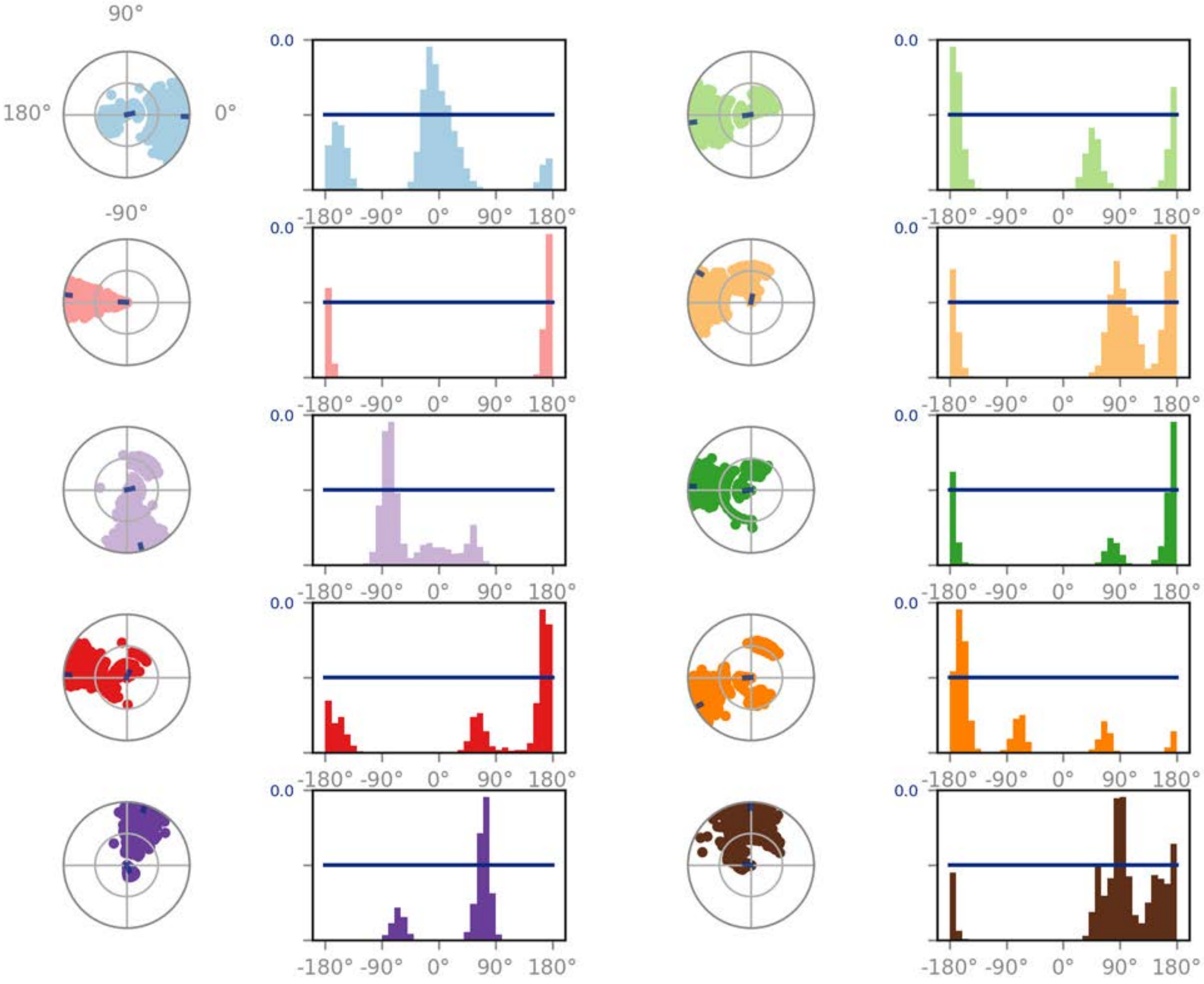

Ligand Torsion Profile (cont.)

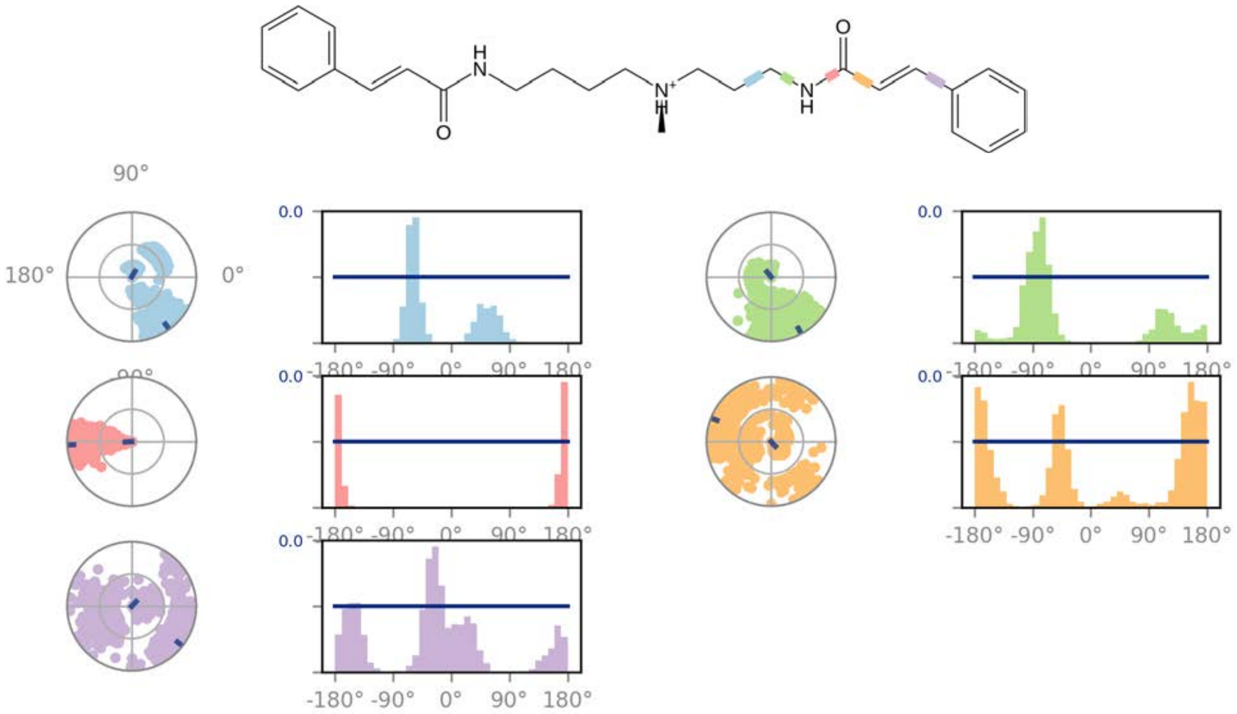

The ligand torsions plot summarizes the conformational evolution of every rotatable bond (RB) in the ligand throughout the simulation trajectory (0.00 through 10.00 nsec). The top panel shows the 2d schematic of a ligand with color-coded rotatable bonds. Each rotatable bond torsion is accompanied by a dial plot and bar plots of the same color.

Dial (or radial) plots describe the conformation of the torsion throughout the course of the simulation. The beginning of the simulation is in the center of the radial plot and the time evolution is plotted radially outwards.

The bar plots summarize the data on the dial plots, by showing the probability density of the torsion. If torsional potential information is available, the plot also shows the potential of the rotatable bond (by summing the potential of the related torsions). The values of the potential are on the left *Y*-axis of the chart, and are expressed in *kcal/mol*. Looking at the histogram and torsion potential relationships may give insights into the conformational strain the ligand undergoes to maintain a protein-bound conformation.

Ligand Properties

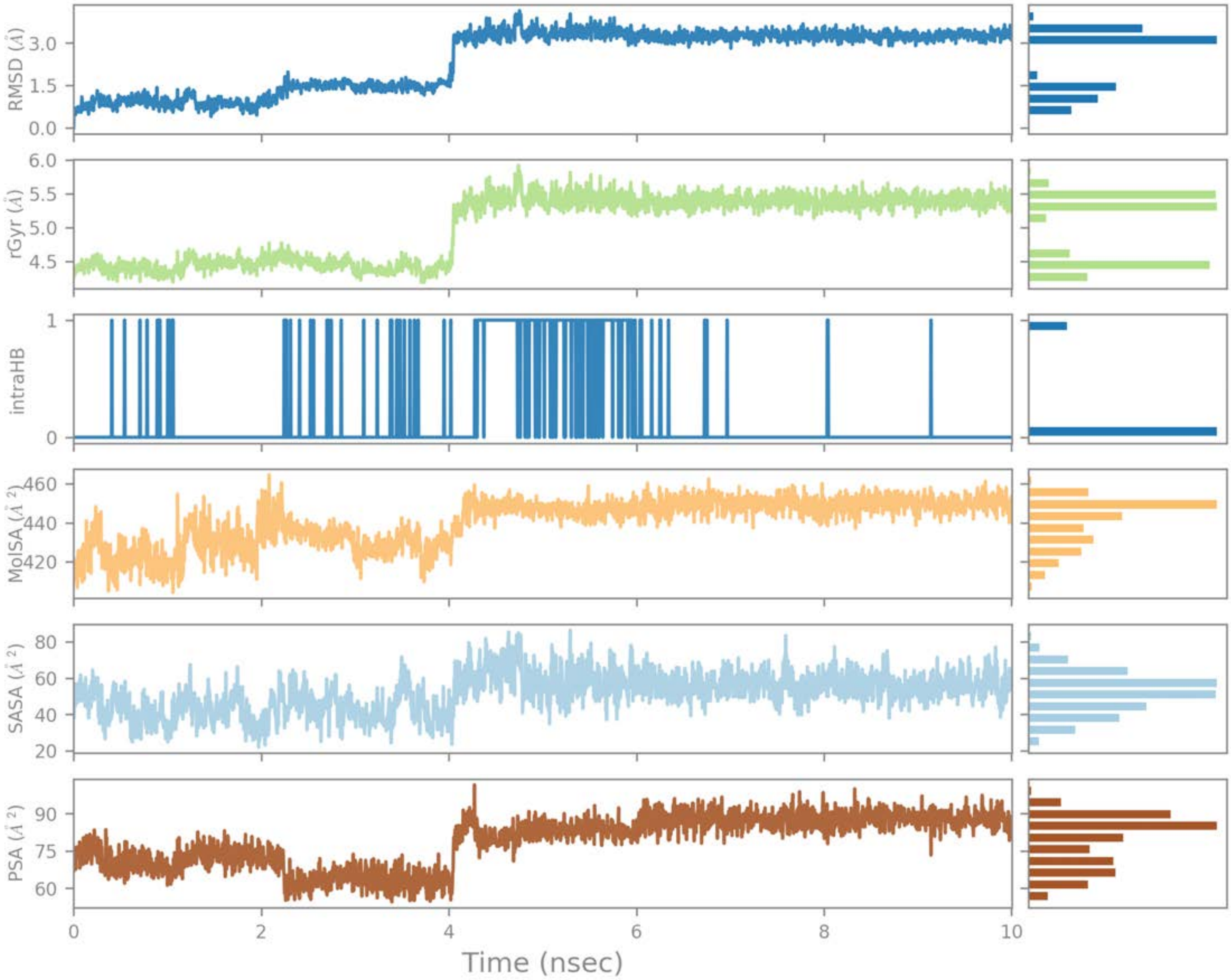

Ligand RMSD: Root mean square deviation of a ligand with respect to the reference conformation (typically the first frame is used as the reference and it is regarded as time *t*=0).

Radius of Gyration (rGyr): Measures the 'extendedness' of a ligand, and is equivalent to its principal moment of inertia.

Intramolecular Hydrogen Bonds (intraHB): Number of internal hydrogen bonds (HB) within a ligand molecule.

Molecular Surface Area (MolSA): Molecular surface calculation with 1.4 Å probe radius. This value is equivalent to a van der Waals surface area.

Solvent Accessible Surface Area (SASA): Surface area of a molecule accessible by a water molecule.

Polar Surface Area (PSA): Solvent accessible surface area in a molecule contributed only by oxygen and nitrogen atoms.

Report S6

MD Simulation Report on AChE - *N-trans*-Feruloyltyramine Interactions

Simulation Details

Jobname: md_job_6H12_5-vina_1
Entry title: 5-dock-1-rec

CPU #	Job Type	Ensemble	Temp. [K]	Sim. Time [ns]	# Atoms	# Waters	Charge
1	mdsim	NPT	300.0	10.005	62851	17967	0

Protein Information

Tot. Residues	Prot. Chain(s)	Res. in Chain(s)	# Atoms	# Heavy Atoms	Charge
562	'NoChainId'	ict_values([562]	8798	4471	-10

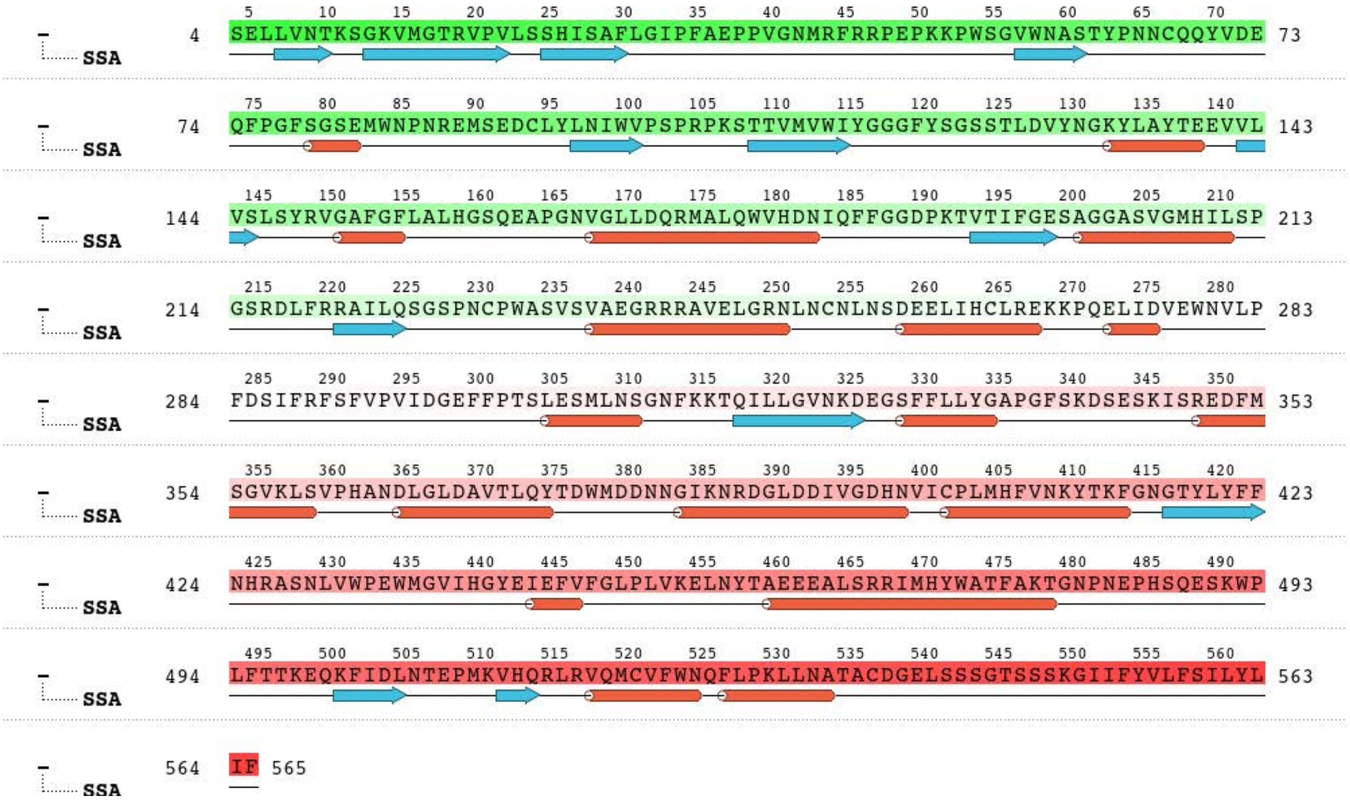

Ligand Information

SMILES	COc(cc1)c(O)cc1\C=C\C(=O)NCCc2ccc(O)cc2
PDB Name	'UNK'
Num. of Atoms	42 (total) 23 (heavy)
Atomic Mass	313.356 au
Charge	0
Mol. Formula	C18H19NO4
Num. of Fragments	4
Num. of Rot. Bonds	9

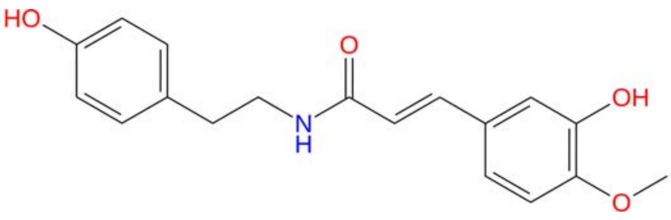

Counter Ion/Salt Information

Type	Num.	Concentration [mM]	Total Charge
Na	60	60.717	+60
Cl	50	50.598	-50

Protein-Ligand RMSD

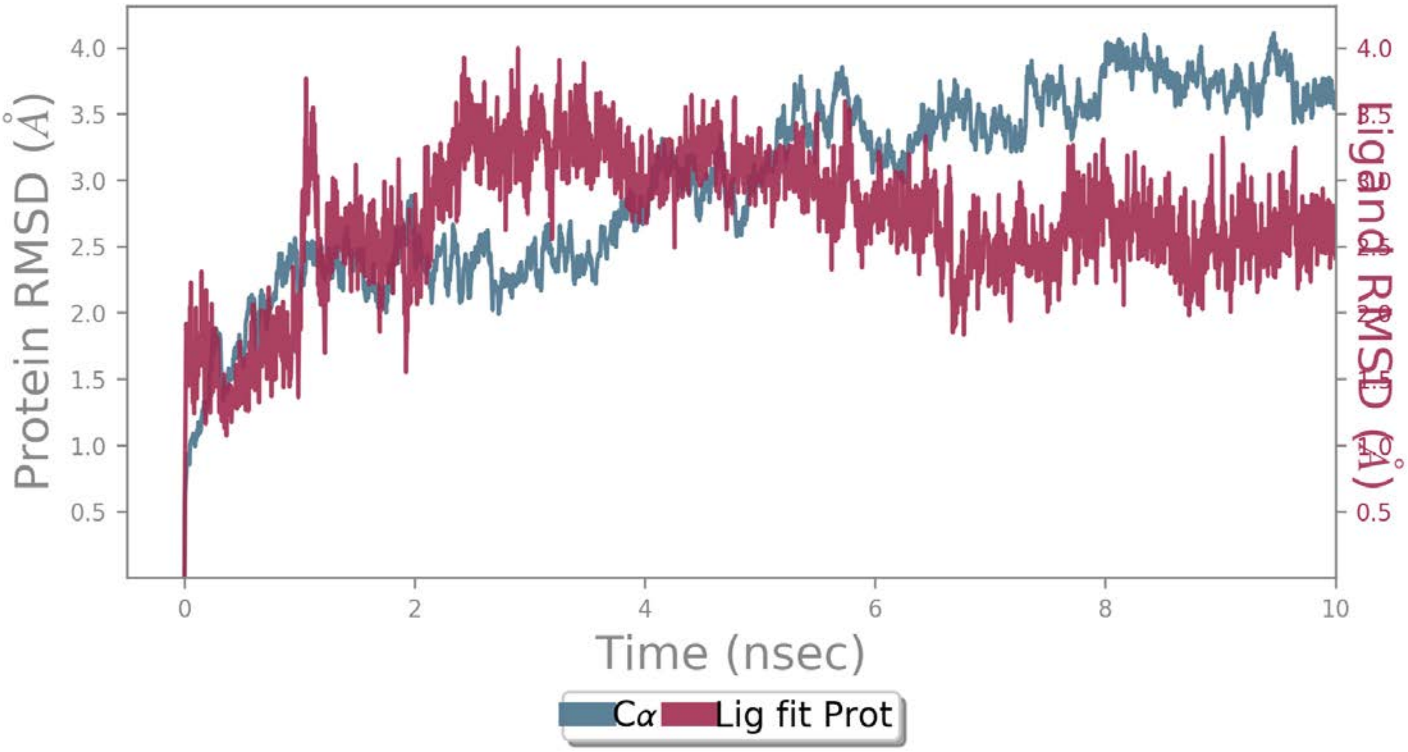

The Root Mean Square Deviation (RMSD) is used to measure the average change in displacement of a selection of atoms for a particular frame with respect to a reference frame. It is calculated for all frames in the trajectory. The RMSD for frame *x* is:

$$RMSD_x = \sqrt{\frac{1}{N}\sum_{i=1}^{N}(r'_i(t_x)) - r_i(t_{ref}))^2}$$

where *N* is the number of atoms in the atom selection; t_{ref} is the reference time, (typically the first frame is used as the reference and it is regarded as time *t*=0); and *r'* is the position of the selected atoms in frame *x* after superimposing on the reference frame, where frame *x* is recorded at time t_x. The procedure is repeated for every frame in the simulation trajectory.

Protein RMSD: The above plot shows the RMSD evolution of a protein (left *Y*-axis). All protein frames are first aligned on the reference frame backbone, and then the RMSD is calculated based on the atom selection. Monitoring the RMSD of the protein can give insights into its structural conformation throughout the simulation. RMSD analysis can indicate if the simulation has equilibrated — its fluctuations towards the end of the simulation are around some thermal average structure. Changes of the order of 1-3 Å are perfectly acceptable for small, globular proteins. Changes much larger than that, however, indicate that the protein is undergoing a large conformational change during the simulation. It is also important that your simulation converges — the RMSD values stabilize around a fixed value. If the RMSD of the protein is still increasing or decreasing on average at the end of the simulation, then your system has not equilibrated, and your simulation may not be long enough for rigorous analysis.

Ligand RMSD: Ligand RMSD (right *Y*-axis) indicates how stable the ligand is with respect to the protein and its binding pocket. In the above plot, 'Lig fit Prot' shows the RMSD of a ligand when the protein-ligand complex is first aligned on the protein backbone of the reference and then the RMSD of the ligand heavy atoms is measured. If the values observed are significantly larger than the RMSD of the protein, then it is likely that the ligand has diffused away from its initial binding site.

Protein RMSF

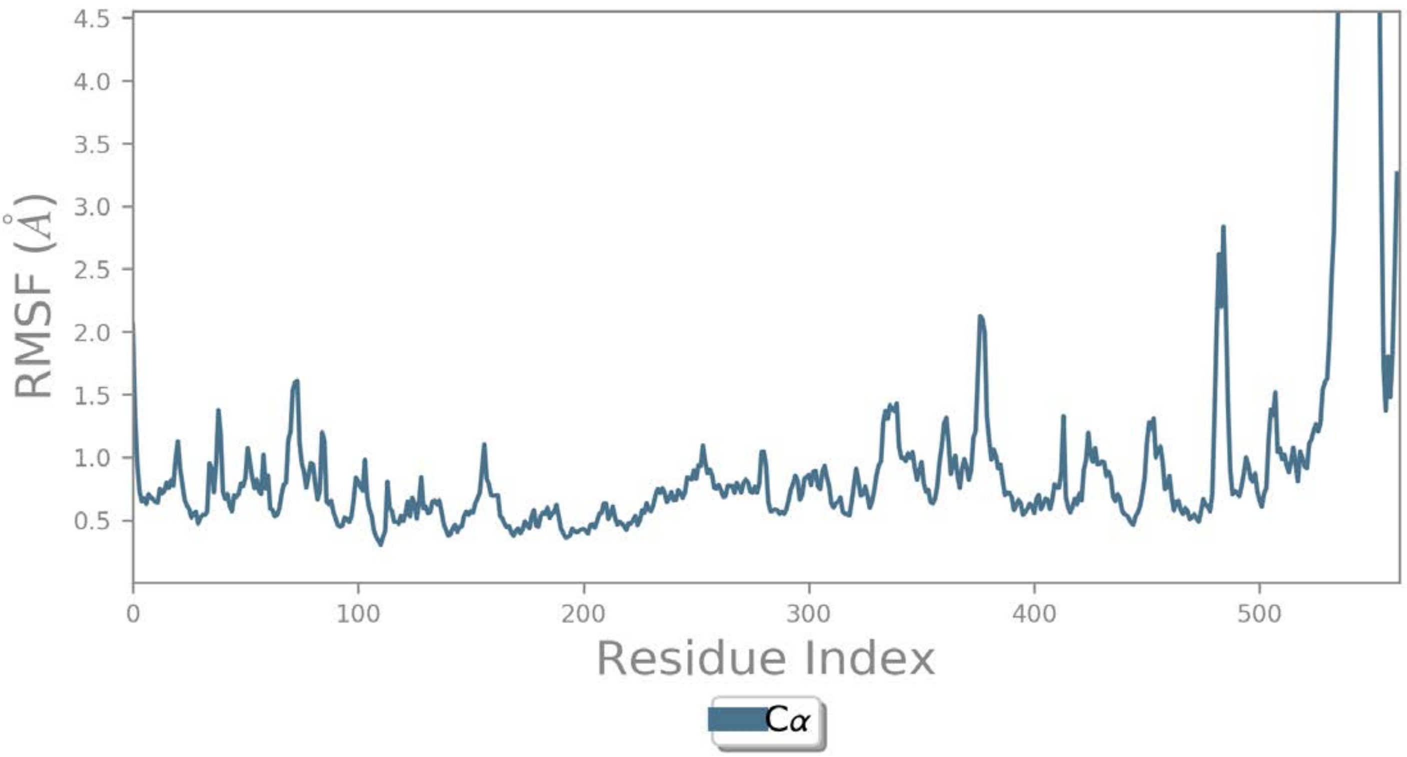

The Root Mean Square Fluctuation (RMSF) is useful for characterizing local changes along the protein chain. The RMSF for residue *i* is:

$$RMSF_i = \sqrt{\frac{1}{T}\sum_{t=1}^{T} < (r'_i(t)) - r_i(t_{ref}))^2 >}$$

where T is the trajectory time over which the RMSF is calculated, t_{ref} is the reference time, r_i is the position of residue *i*; *r'* is the position of atoms in residue *i* after superposition on the reference, and the angle brackets indicate that the average of the square distance is taken over the selection of atoms in the residue.

On this plot, peaks indicate areas of the protein that fluctuate the most during the simulation. Typically you will observe that the tails (*N*- and *C*-terminal) fluctuate more than any other part of the protein. Secondary structure elements like alpha helices and beta strands are usually more rigid than the unstructured part of the protein, and thus fluctuate less than the loop regions.

Protein Secondary Structure

% Helix	% Strand	% Total SSE
28.93	13.26	42.19

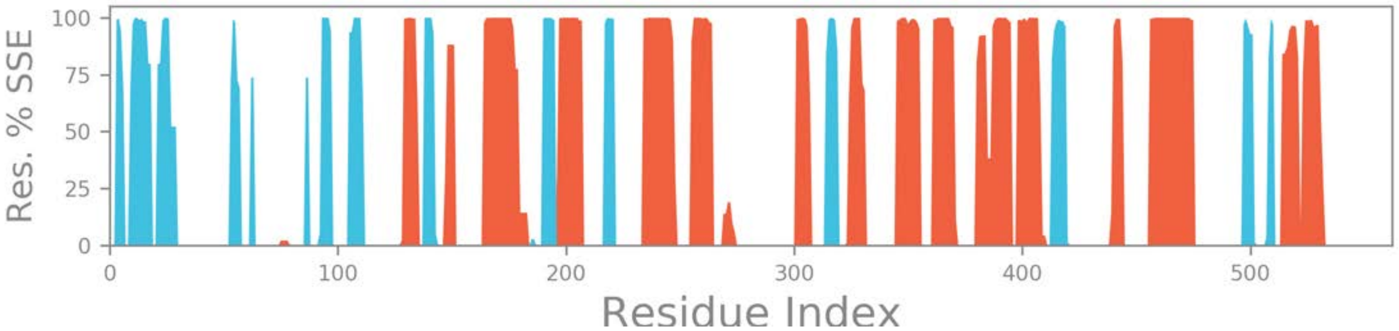

Protein secondary structure elements (SSE) like alpha-helices and beta-strands are monitored throughout the simulation. The plot above reports SSE distribution by residue index throughout the protein structure. The plot below summarizes the SSE composition for each trajectory frame over the course of the simulation, and the plot at the bottom monitors each residue and its SSE assignment over time.

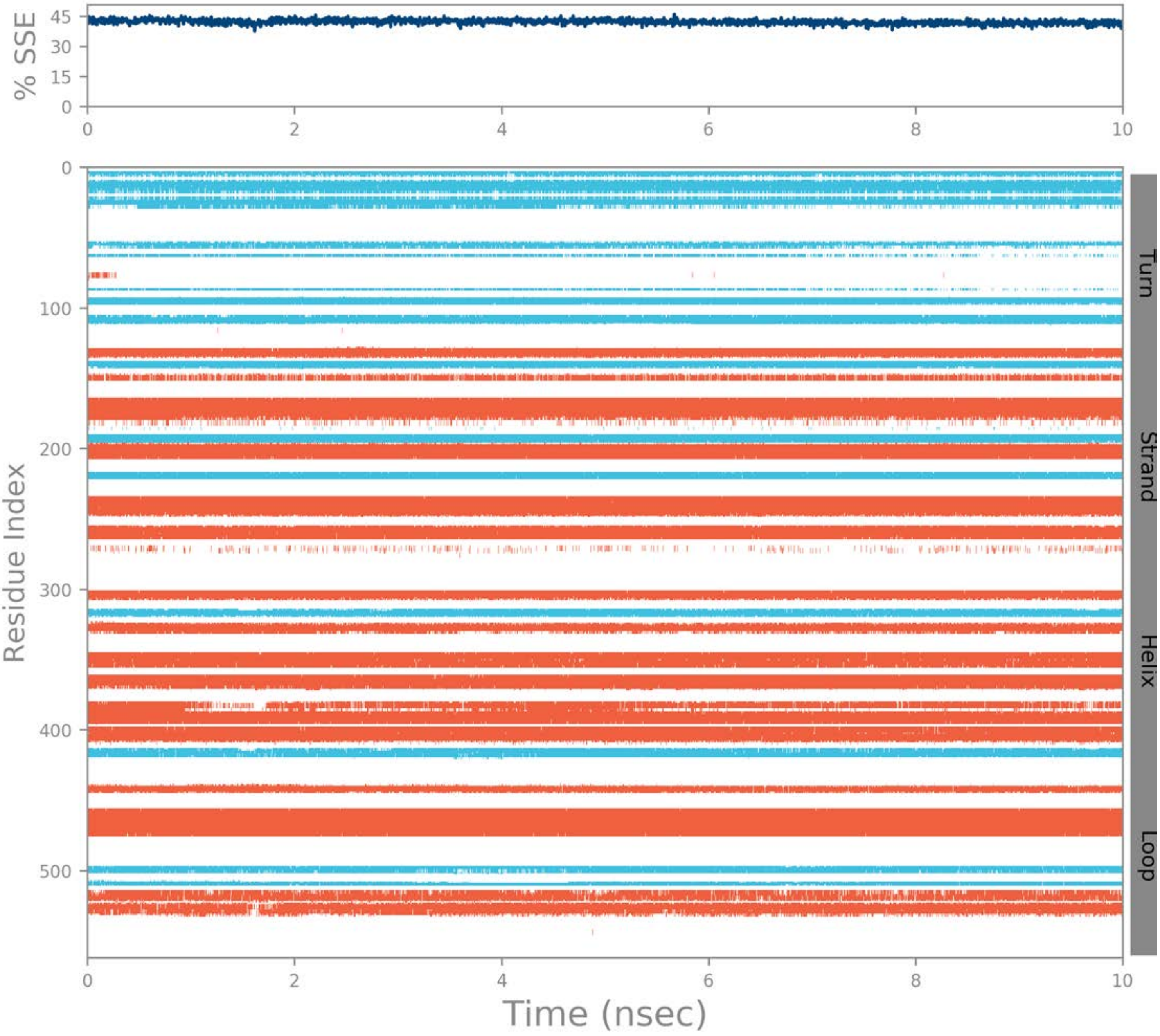

RMSF of *N-trans*-Feruloyltyramine Ligand

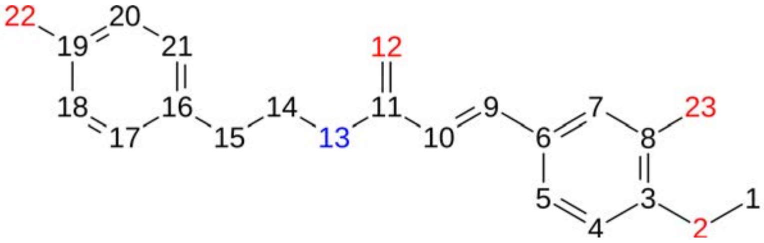

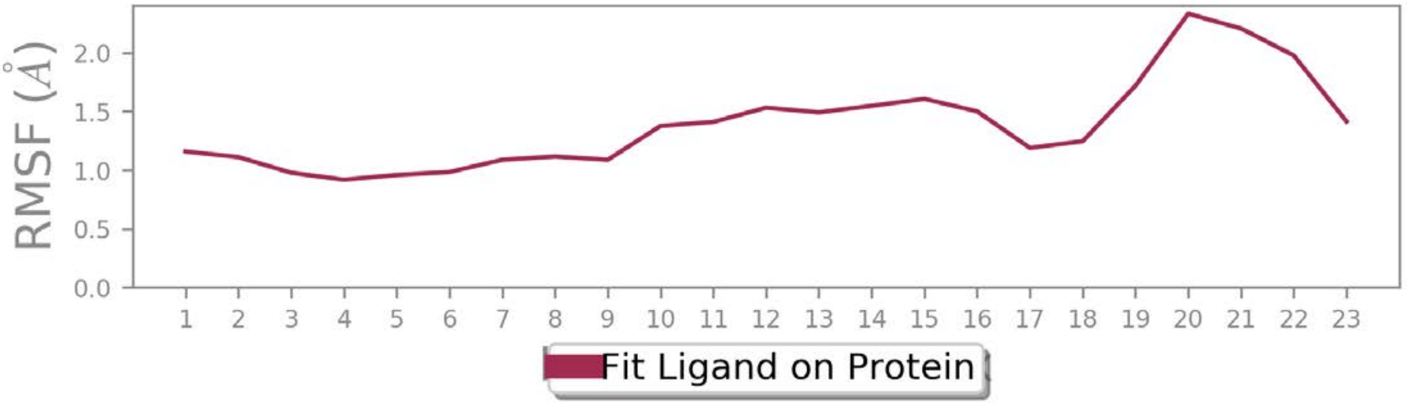

The Ligand Root Mean Square Fluctuation (L-RMSF) is useful for characterizing changes in the ligand atom positions. The RMSF for atom *i* is:

$$RMSF_i = \sqrt{\frac{1}{T}\sum_{t=1}^{T}(r_i'(t)) - r_i(t_{ref}))^2}$$

where *T* is the trajectory time over which the RMSF is calculated, t_{ref} is the reference time (usually for the first frame, and is regarded as the *zero* of time); *r* is the position of atom *i* in the reference at time t_{ref}, and *r'* is the position of atom *i* at time *t* after superposition on the reference frame.

Ligand RMSF shows the ligand's fluctuations broken down by atom, corresponding to the 2D structure in the top panel. The ligand RMSF may give you insights on how ligand fragments interact with the protein and their entropic role in the binding event. In the bottom panel, the 'Fit Ligand on Protein' line shows the ligand fluctuations, with respect to the protein. The protein-ligand complex is first aligned on the protein backbone and then the ligand RMSF is measured on the ligand heavy atoms.

Protein-Ligand Contacts

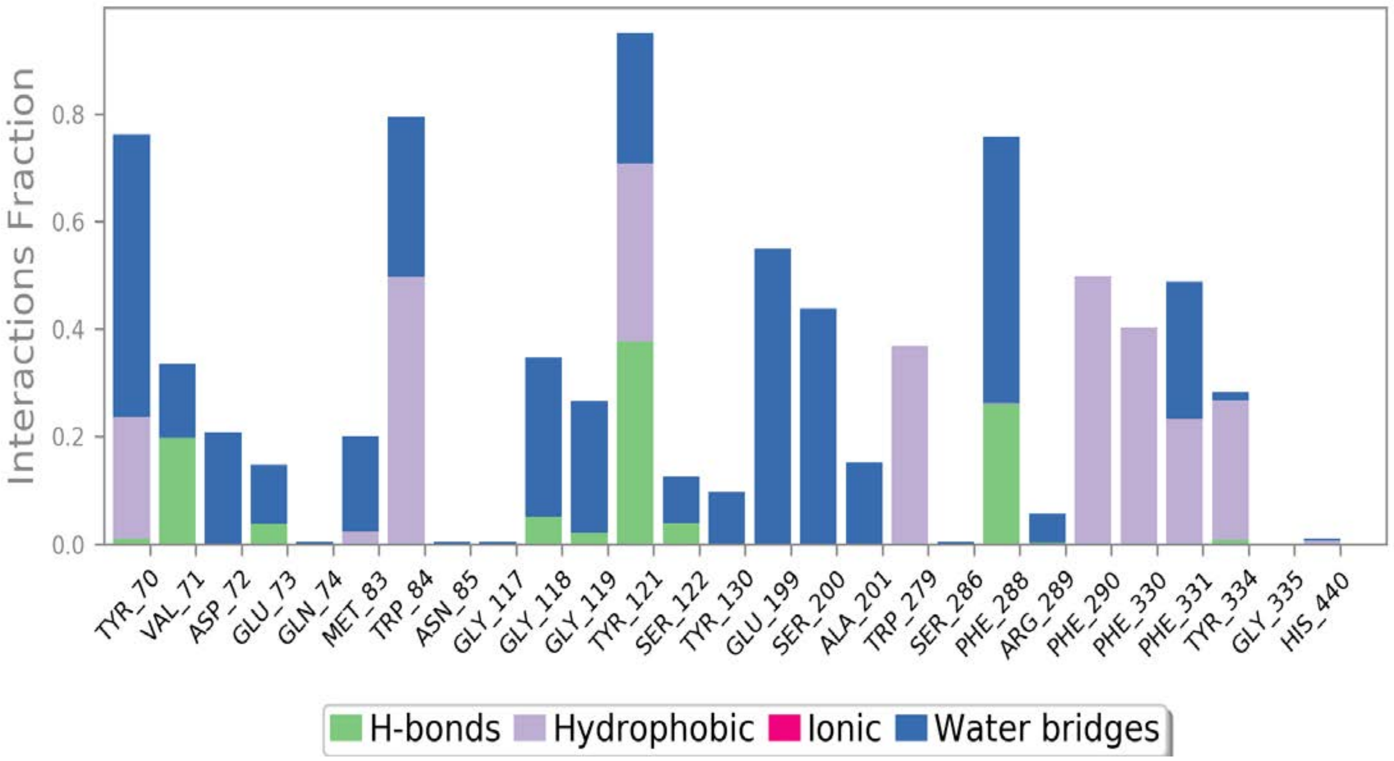

Protein interactions with the ligand can be monitored throughout the simulation. These interactions can be categorized by type and summarized, as shown in the plot above. Protein-ligand interactions (or 'contacts') are categorized into four types: Hydrogen Bonds, Hydrophobic, Ionic and Water Bridges. Each interaction type contains more specific subtypes, which can be explored through the 'Simulation Interactions Diagram' panel. The stacked bar charts are normalized over the course of the trajectory: for example, a value of 0.7 suggests that 70% of the simulation time the specific interaction is maintained. Values over 1.0 are possible as some protein residue may make multiple contacts of same subtype with the ligand.

Hydrogen Bonds: (H-bonds) play a significant role in ligand binding. Consideration of hydrogen-bonding properties in drug design is important because of their strong influence on drug specificity, metabolization and adsorption. Hydrogen bonds between a protein and a ligand can be further broken down into four subtypes: backbone acceptor; backbone donor; side-chain acceptor; side-chain donor.
The current geometric criteria for protein-ligand H-bond is: distance of 2.5 Å between the donor and acceptor atoms (D—H···A); a donor angle of ≥120° between the donor-hydrogen-acceptor atoms (D—H···A); and an acceptor angle of ≥90° between the hydrogen-acceptor-bonded_atom atoms (H···A—X).

Hydrophobic contacts: fall into three subtypes: π-Cation; π-π; and Other, non-specific interactions. Generally these type of interactions involve a hydrophobic amino acid and an aromatic or aliphatic group on the ligand, but we have extended this category to also include π-Cation interactions.
The current geometric criteria for hydrophobic interactions is as follows: π-Cation — Aromatic and charged groups within 4.5 Å; π-π — Two aromatic groups stacked face-to-face or face-to-edge; Other — A non-specific hydrophobic sidechain within 3.6 Å of a ligand's aromatic or aliphatic carbons.

Ionic interactions: or polar interactions, are between two oppositely charged atoms that are within 3.7 Å of each other and do not involve a hydrogen bond. We also monitor Protein-Metal-Ligand interactions, which are defined by a metal ion coordinated within 3.4 Å of protein's and ligand's heavy atoms (except carbon). All ionic interactions are broken down into two subtypes: those mediated by a protein backbone or side chains.

Water Bridges: are hydrogen-bonded protein-ligand interactions mediated by a water molecule. The hydrogen-bond geometry is slightly relaxed from the standard H-bond definition.
The current geometric criteria for a protein-water or water-ligand H-bond are: a distance of 2.8 Å between the donor and acceptor atoms (D—H···A); a donor angle of ≥110° between the donor-hydrogen-acceptor atoms (D—H···A); and an acceptor angle of ≥90° between the hydrogen-acceptor-bonded_atom atoms (H···A—X).

Protein-Ligand Contacts (cont.)

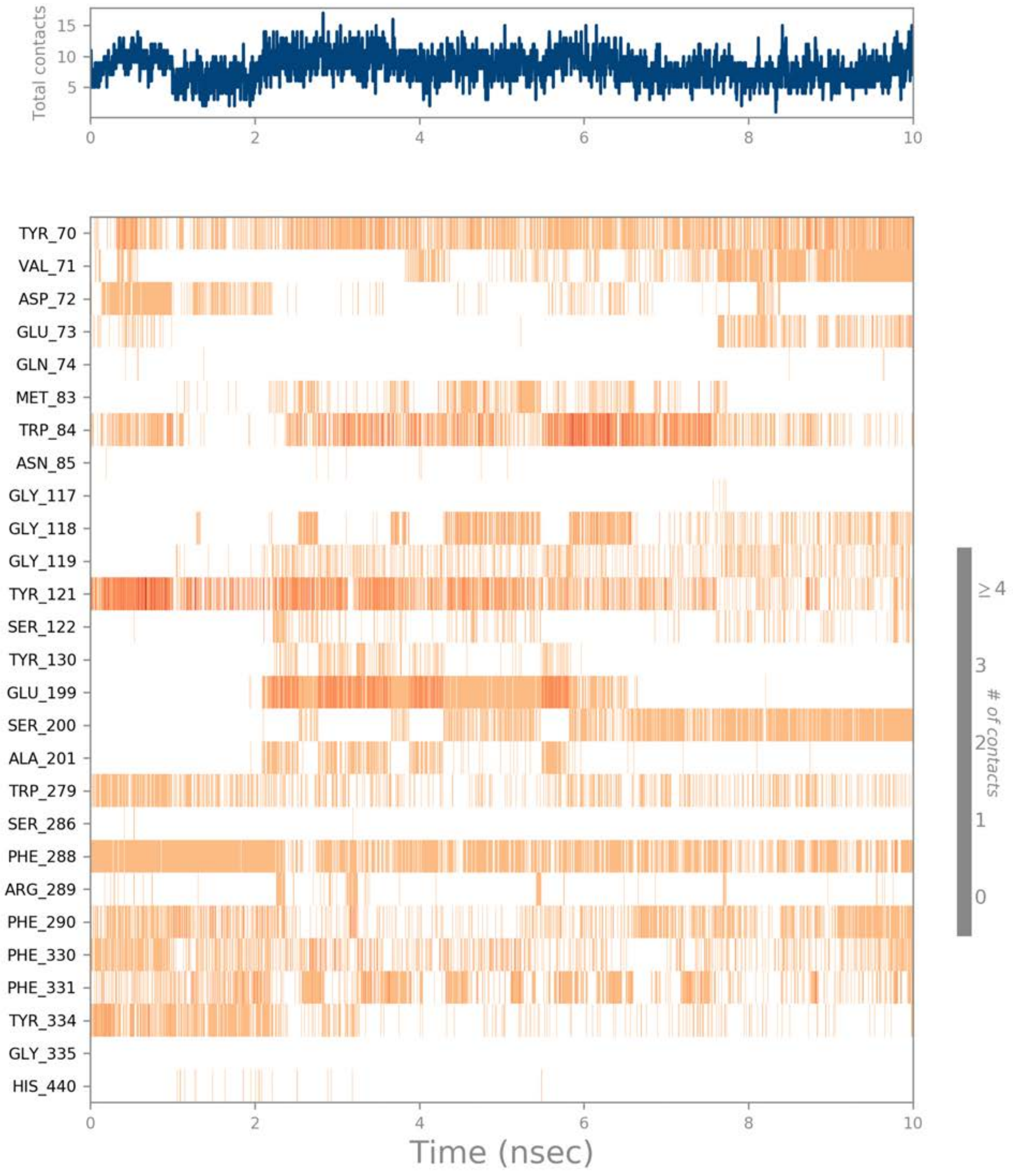

A timeline representation of the interactions and contacts (**H-bonds**, **Hydrophobic**, **Ionic**, **Water bridges**) summarized in the previous page. The top panel shows the total number of specific contacts the protein makes with the ligand over the course of the trajectory. The bottom panel shows which residues interact with the ligand in each trajectory frame. Some residues make more than one specific contact with the ligand, which is represented by a darker shade of orange, according to the scale to the right of the plot.

Ligand-Protein Contacts

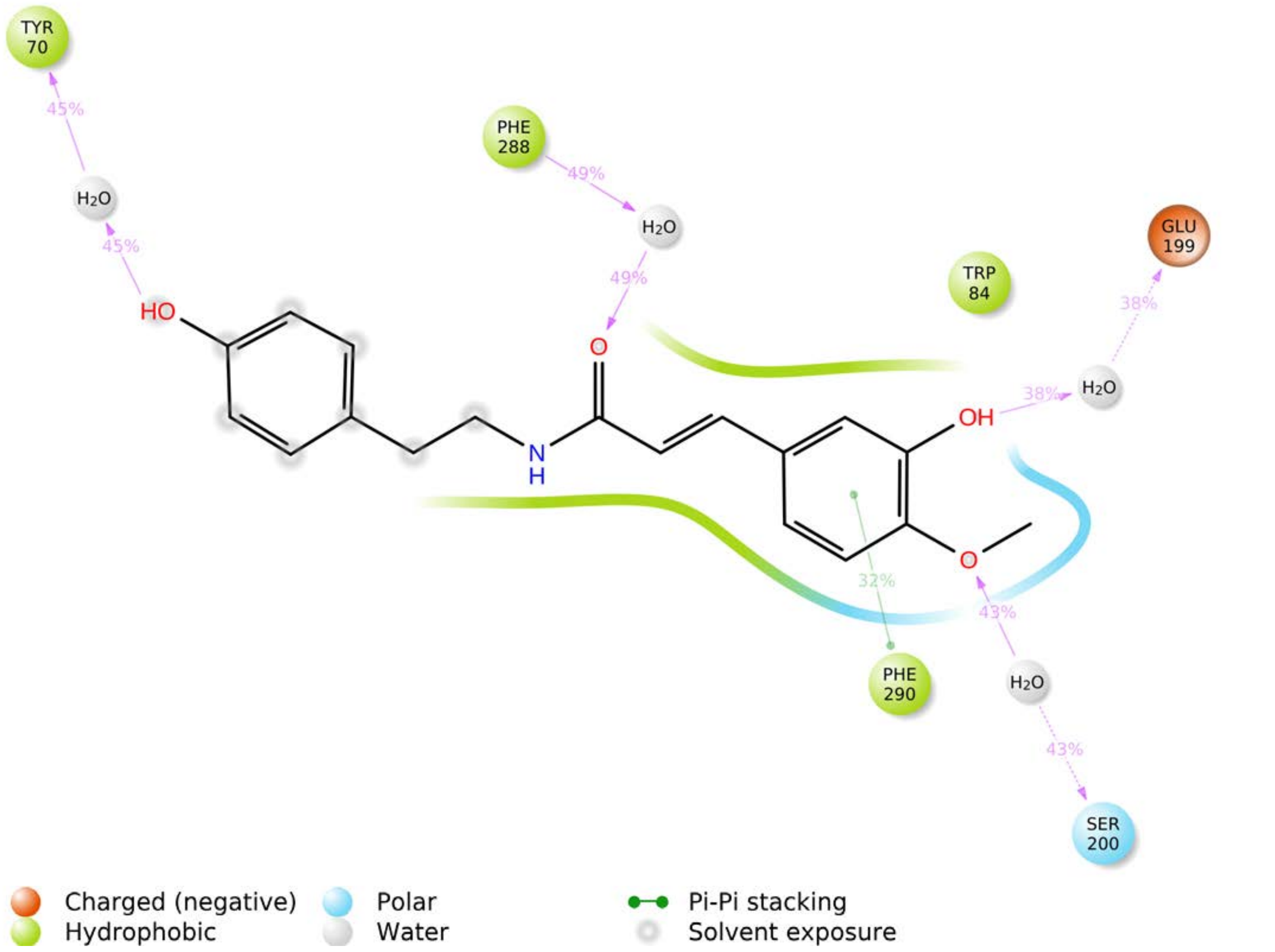

A schematic of detailed ligand atom interactions with the protein residues. Interactions that occur more than **30.0%** of the simulation time in the selected trajectory (0.00 through 10.00 nsec), are shown.
Note: it is possible to have interactions with >100% as some residues may have multiple interactions of a single type with the same ligand atom. For example, the ARG side chain has four H-bond donors that can all hydrogen-bond to a single H-bond acceptor.

Ligand Torsion Profile

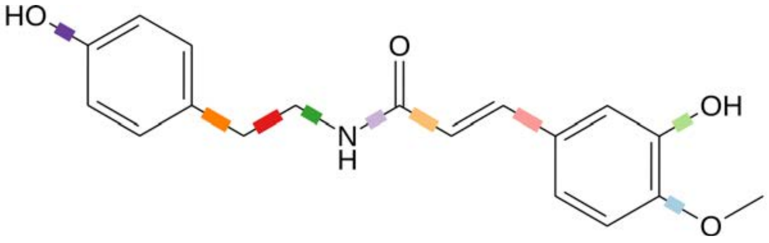

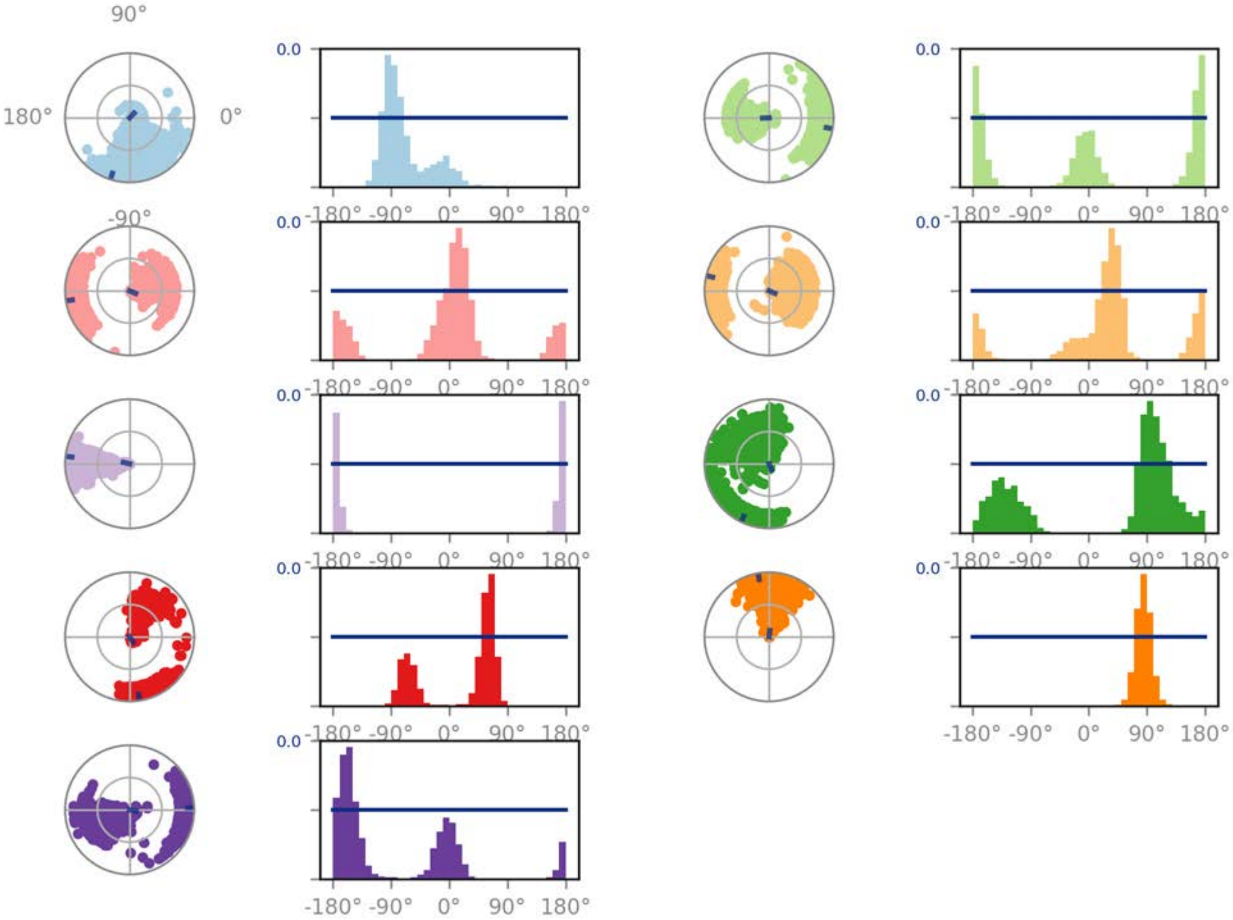

The ligand torsions plot summarizes the conformational evolution of every rotatable bond (RB) in the ligand throughout the simulation trajectory (0.00 through 10.00 nsec). The top panel shows the 2d schematic of a ligand with color-coded rotatable bonds. Each rotatable bond torsion is accompanied by a dial plot and bar plots of the same color.

Dial (or radial) plots describe the conformation of the torsion throughout the course of the simulation. The beginning of the simulation is in the center of the radial plot and the time evolution is plotted radially outwards.

The bar plots summarize the data on the dial plots, by showing the probability density of the torsion. If torsional potential information is available, the plot also shows the potential of the rotatable bond (by summing the potential of the related torsions). The values of the potential are on the left *Y*-axis of the chart, and are expressed in *kcal/mol*. Looking at the histogram and torsion potential relationships may give insights into the conformational strain the ligand undergoes to maintain a protein-bound conformation.

Ligand Properties

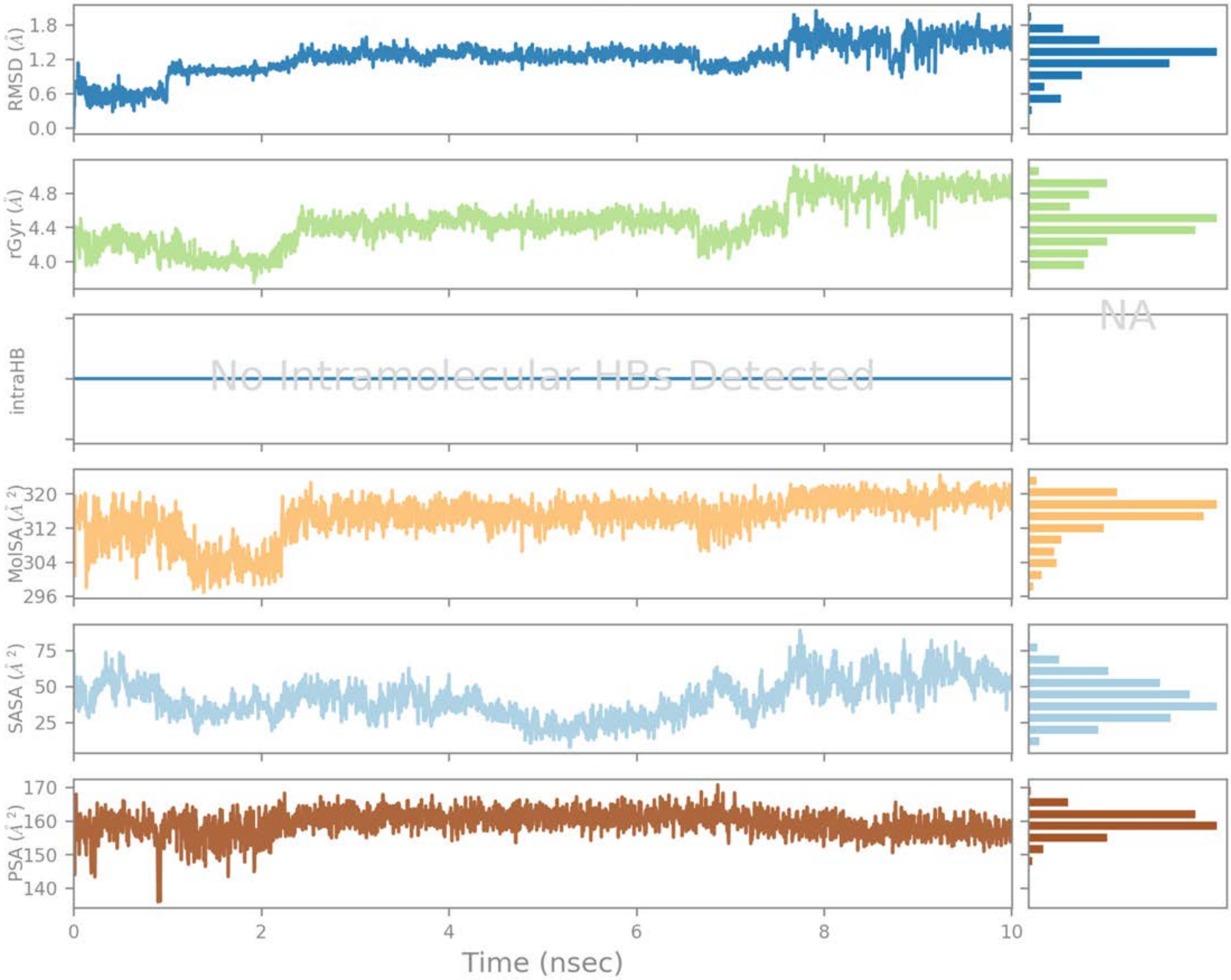

Ligand RMSD: Root mean square deviation of a ligand with respect to the reference conformation (typically the first frame is used as the reference and it is regarded as time *t*=0).

Radius of Gyration (rGyr): Measures the 'extendedness' of a ligand, and is equivalent to its principal moment of inertia.

Intramolecular Hydrogen Bonds (intraHB): Number of internal hydrogen bonds (HB) within a ligand molecule.

Molecular Surface Area (MolSA): Molecular surface calculation with 1.4 Å probe radius. This value is equivalent to a van der Waals surface area.

Solvent Accessible Surface Area (SASA): Surface area of a molecule accessible by a water molecule.

Polar Surface Area (PSA): Solvent accessible surface area in a molecule contributed only by oxygen and nitrogen atoms.

SCHRÖDINGER®

Report S7
MD Simulation Report on BChE - Neostigmine Interactions

Simulation Details

Jobname: md_BChE_NEO_XXX
Entry title: 6EP4 - preprocessed NEO_from_MD - preprocessed NEO_from_LigPrep - preprocessed

CPU #	Job Type	Ensemble	Temp. [K]	Sim. Time [ns]	# Atoms	# Waters	Charge
1	mdsim	NPT	300.0	10.006	48097	13242	0

Protein Information

Tot. Residues	Prot. Chain(s)	Res. in Chain(s)	# Atoms	# Heavy Atoms	Charge
523	'A'	ict_values([523]	8258	4171	+4

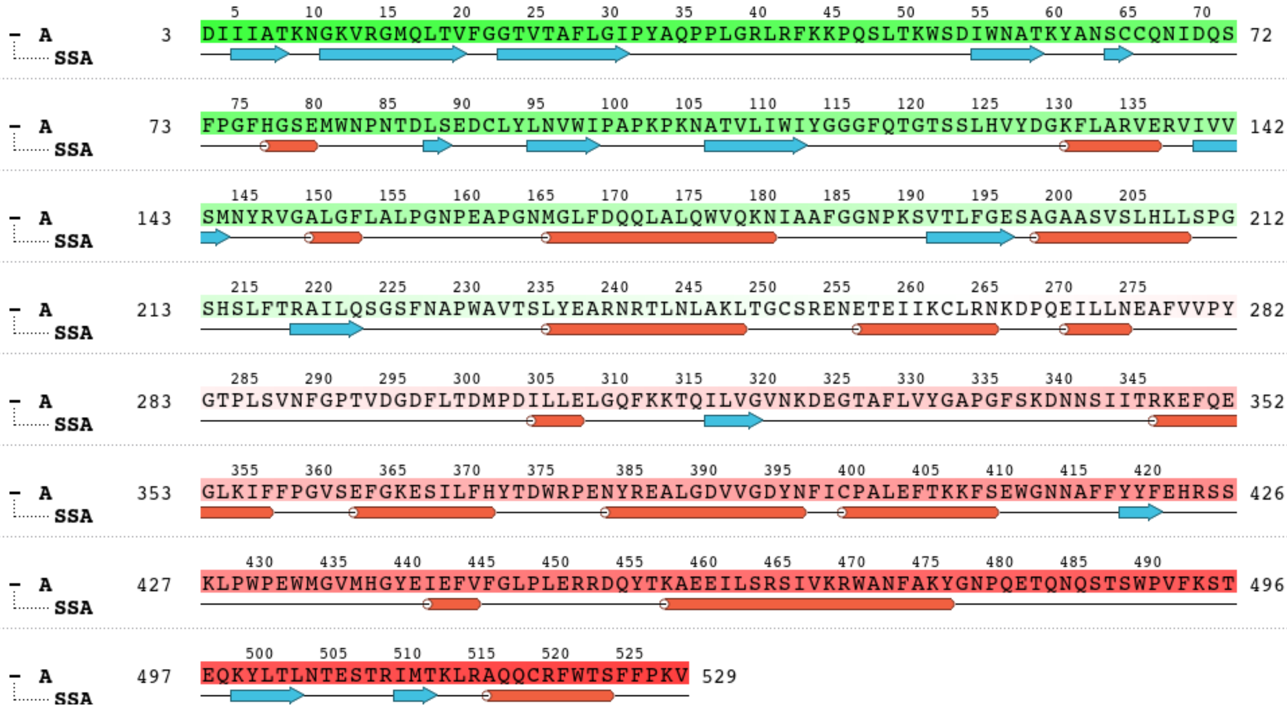

Ligand Information

SMILES	CN(C)C(=O)Oc(n1)cccc1[N+](C)(C)C
PDB Name	'UNK'
Num. of Atoms	34 (total) 16 (heavy)
Atomic Mass	224.285 au
Charge	+1
Mol. Formula	C11H18N3O2
Num. of Fragments	5
Num. of Rot. Bonds	4

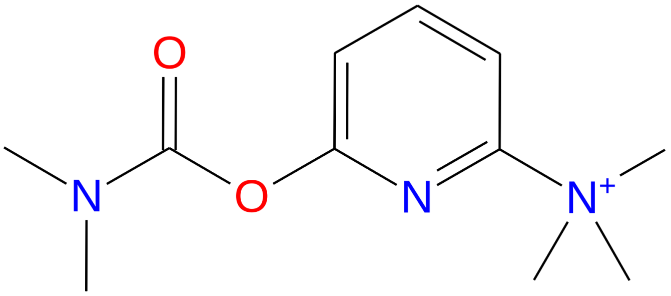

Counter Ion/Salt Information

Type	Num.	Concentration [mM]	Total Charge
Cl	42	57.668	-42
Na	37	50.803	+37

Protein-Ligand RMSD

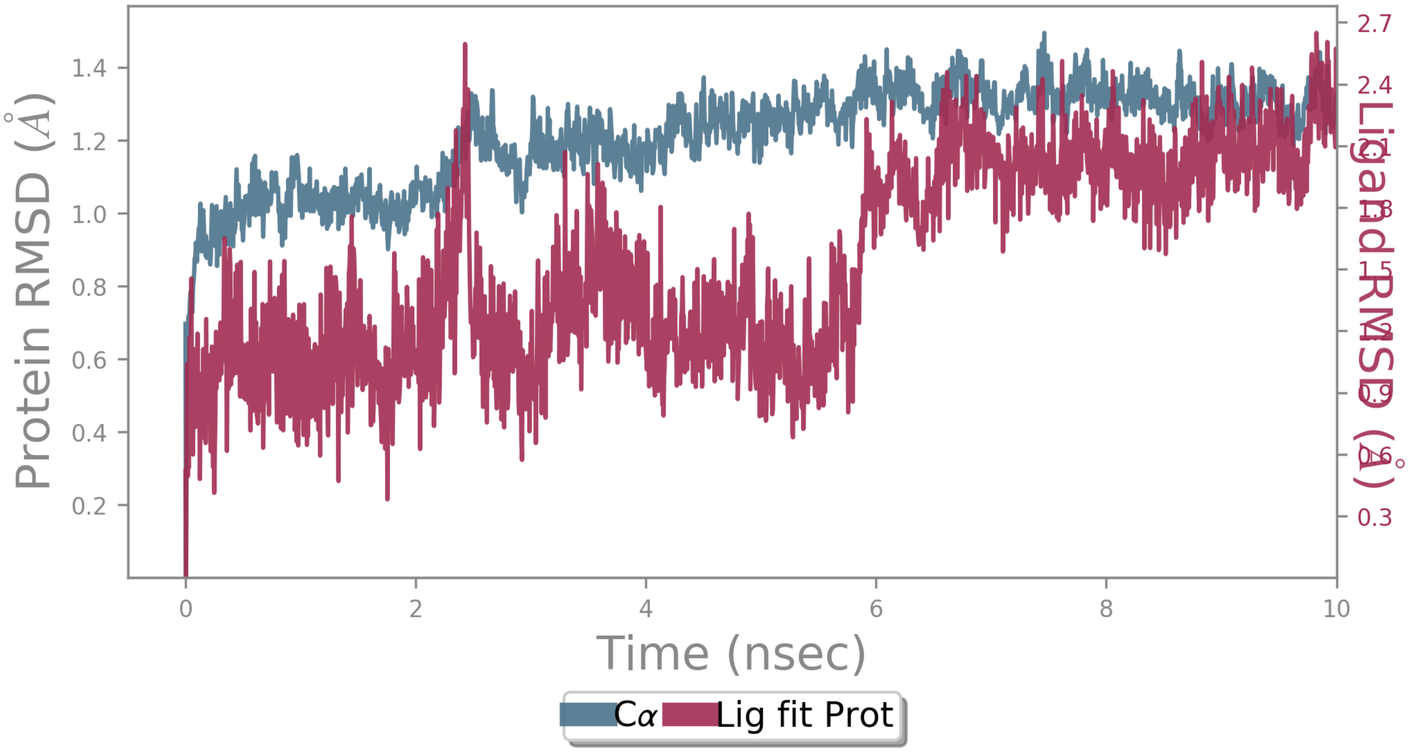

The Root Mean Square Deviation (RMSD) is used to measure the average change in displacement of a selection of atoms for a particular frame with respect to a reference frame. It is calculated for all frames in the trajectory. The RMSD for frame *x* is:

$$RMSD_x = \sqrt{\frac{1}{N}\sum_{i=1}^{N}(r'_i(t_x)) - r_i(t_{ref}))^2}$$

where *N* is the number of atoms in the atom selection; t_{ref} is the reference time, (typically the first frame is used as the reference and it is regarded as time *t*=0); and *r'* is the position of the selected atoms in frame *x* after superimposing on the reference frame, where frame *x* is recorded at time t_x. The procedure is repeated for every frame in the simulation trajectory.

Protein RMSD: The above plot shows the RMSD evolution of a protein (left *Y*-axis). All protein frames are first aligned on the reference frame backbone, and then the RMSD is calculated based on the atom selection. Monitoring the RMSD of the protein can give insights into its structural conformation throughout the simulation. RMSD analysis can indicate if the simulation has equilibrated — its fluctuations towards the end of the simulation are around some thermal average structure. Changes of the order of 1-3 Å are perfectly acceptable for small, globular proteins. Changes much larger than that, however, indicate that the protein is undergoing a large conformational change during the simulation. It is also important that your simulation converges — the RMSD values stabilize around a fixed value. If the RMSD of the protein is still increasing or decreasing on average at the end of the simulation, then your system has not equilibrated, and your simulation may not be long enough for rigorous analysis.

Ligand RMSD: Ligand RMSD (right *Y*-axis) indicates how stable the ligand is with respect to the protein and its binding pocket. In the above plot, 'Lig fit Prot' shows the RMSD of a ligand when the protein-ligand complex is first aligned on the protein backbone of the reference and then the RMSD of the ligand heavy atoms is measured. If the values observed are significantly larger than the RMSD of the protein, then it is likely that the ligand has diffused away from its initial binding site.

Protein RMSF

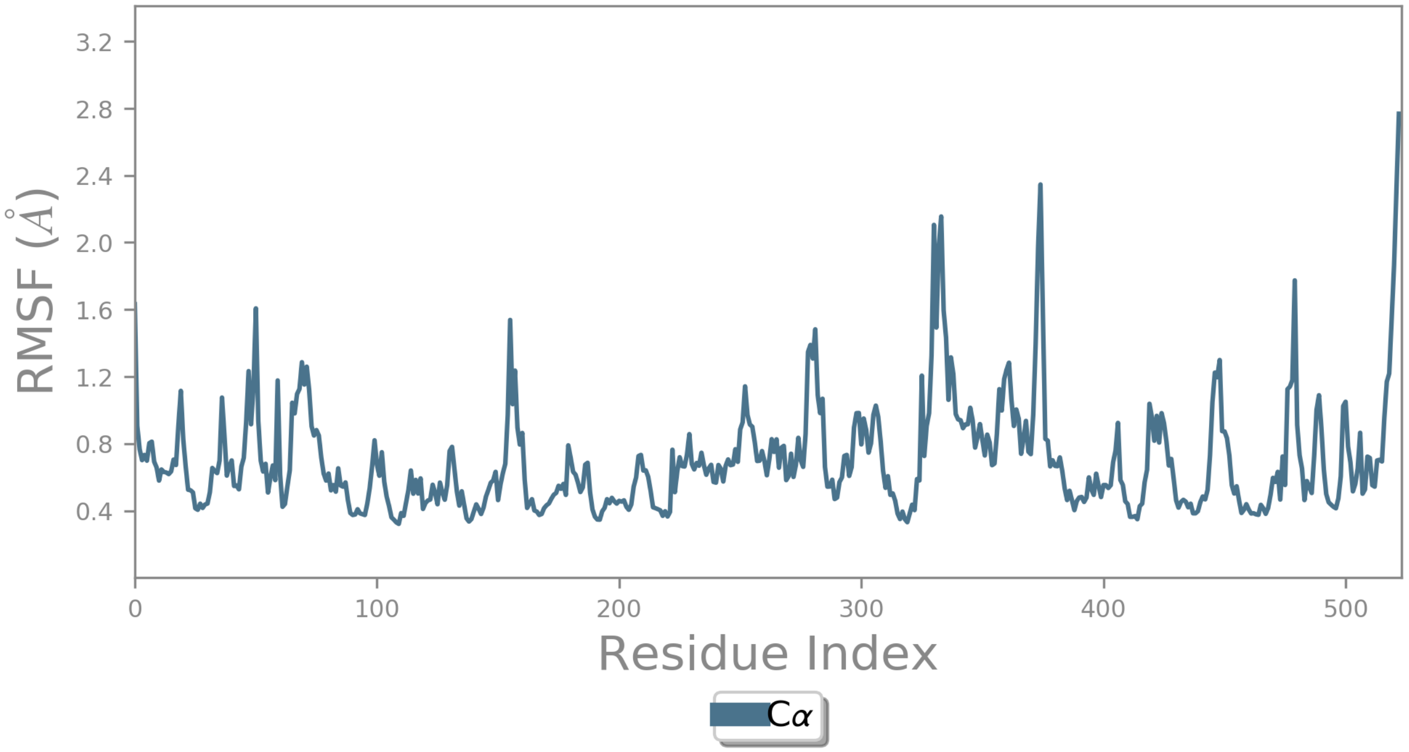

The Root Mean Square Fluctuation (RMSF) is useful for characterizing local changes along the protein chain. The RMSF for residue *i* is:

$$RMSF_i = \sqrt{\frac{1}{T}\sum_{t=1}^{T} < (r_i'(t)) - r_i(t_{ref}))^2 >}$$

where T is the trajectory time over which the RMSF is calculated, t_{ref} is the reference time, r_i is the position of residue *i*; *r'* is the position of atoms in residue *i* after superposition on the reference, and the angle brackets indicate that the average of the square distance is taken over the selection of atoms in the residue.

On this plot, peaks indicate areas of the protein that fluctuate the most during the simulation. Typically you will observe that the tails (*N*- and *C*-terminal) fluctuate more than any other part of the protein. Secondary structure elements like alpha helices and beta strands are usually more rigid than the unstructured part of the protein, and thus fluctuate less than the loop regions.

Protein Secondary Structure

% Helix	% Strand	% Total SSE
29.32	12.71	42.04

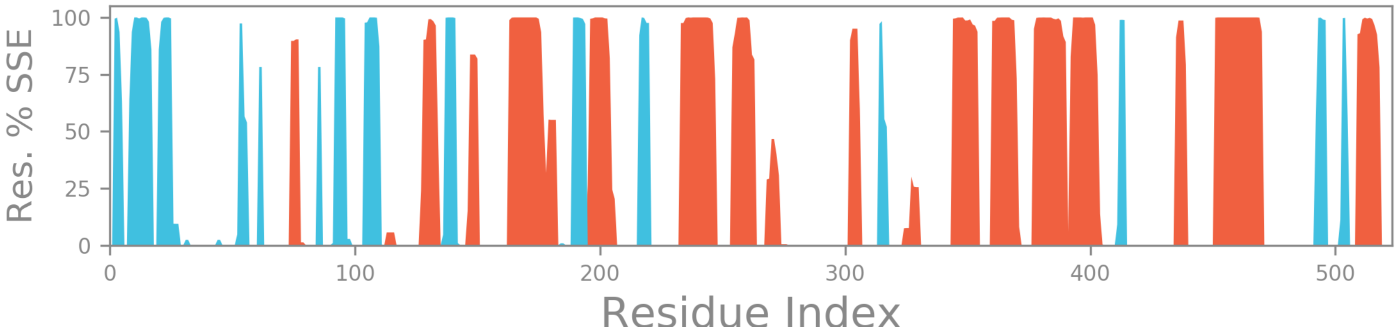

Protein secondary structure elements (SSE) like alpha-helices and beta-strands are monitored throughout the simulation. The plot above reports SSE distribution by residue index throughout the protein structure. The plot below summarizes the SSE composition for each trajectory frame over the course of the simulation, and the plot at the bottom monitors each residue and its SSE assignment over time.

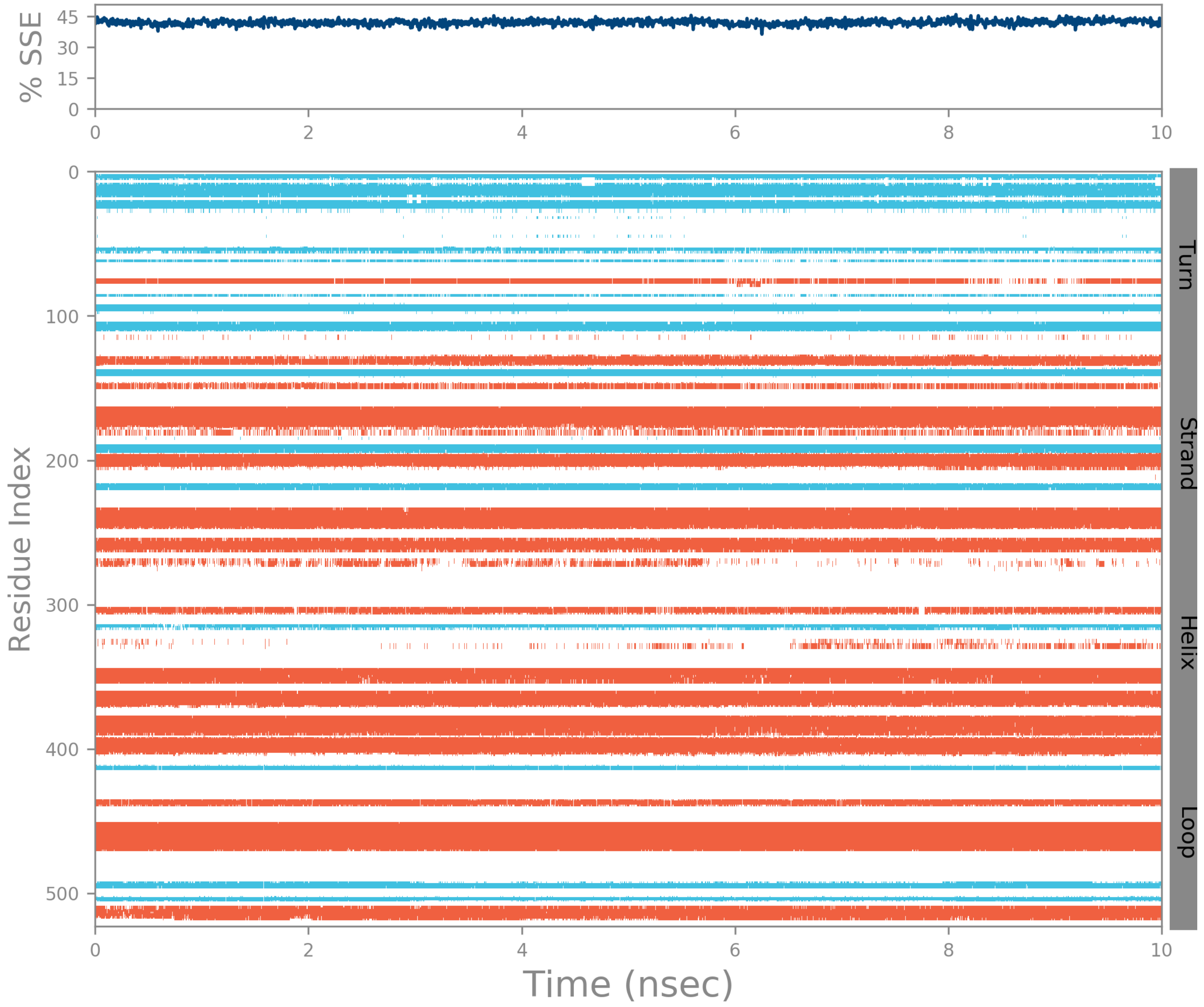

Neostigmine Ligand RMSF

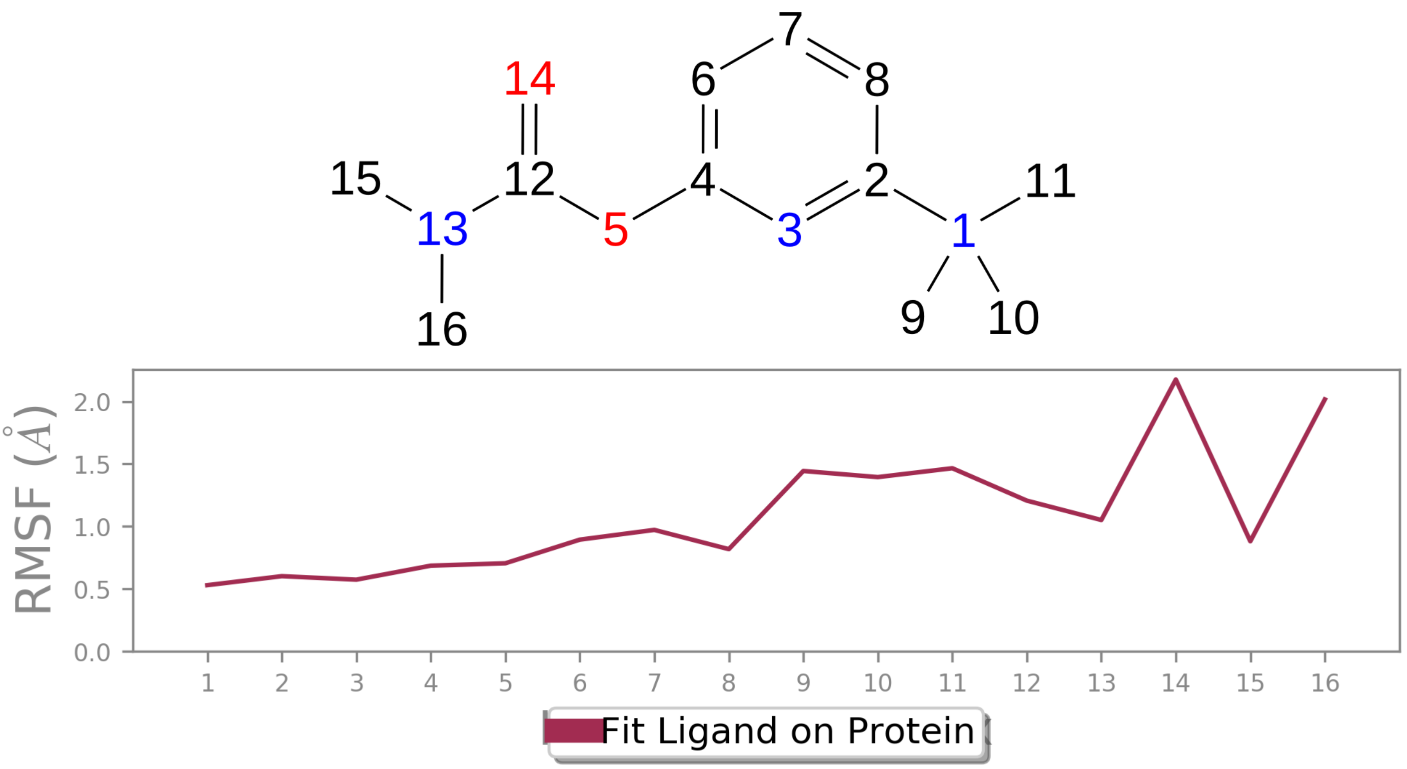

The Ligand Root Mean Square Fluctuation (L-RMSF) is useful for characterizing changes in the ligand atom positions. The RMSF for atom *i* is:

$$RMSF_i = \sqrt{\frac{1}{T}\sum_{t=1}^{T}(r_i'(t)) - r_i(t_{ref}))^2}$$

where T is the trajectory time over which the RMSF is calculated, t_{ref} is the reference time (usually for the first frame, and is regarded as the *zero* of time); r is the position of atom i in the reference at time t_{ref}, and r' is the position of atom i at time t after superposition on the reference frame.

Ligand RMSF shows the ligand's fluctuations broken down by atom, corresponding to the 2D structure in the top panel. The ligand RMSF may give you insights on how ligand fragments interact with the protein and their entropic role in the binding event. In the bottom panel, the 'Fit Ligand on Protein' line shows the ligand fluctuations, with respect to the protein. The protein-ligand complex is first aligned on the protein backbone and then the ligand RMSF is measured on the ligand heavy atoms.

Protein-Ligand Contacts

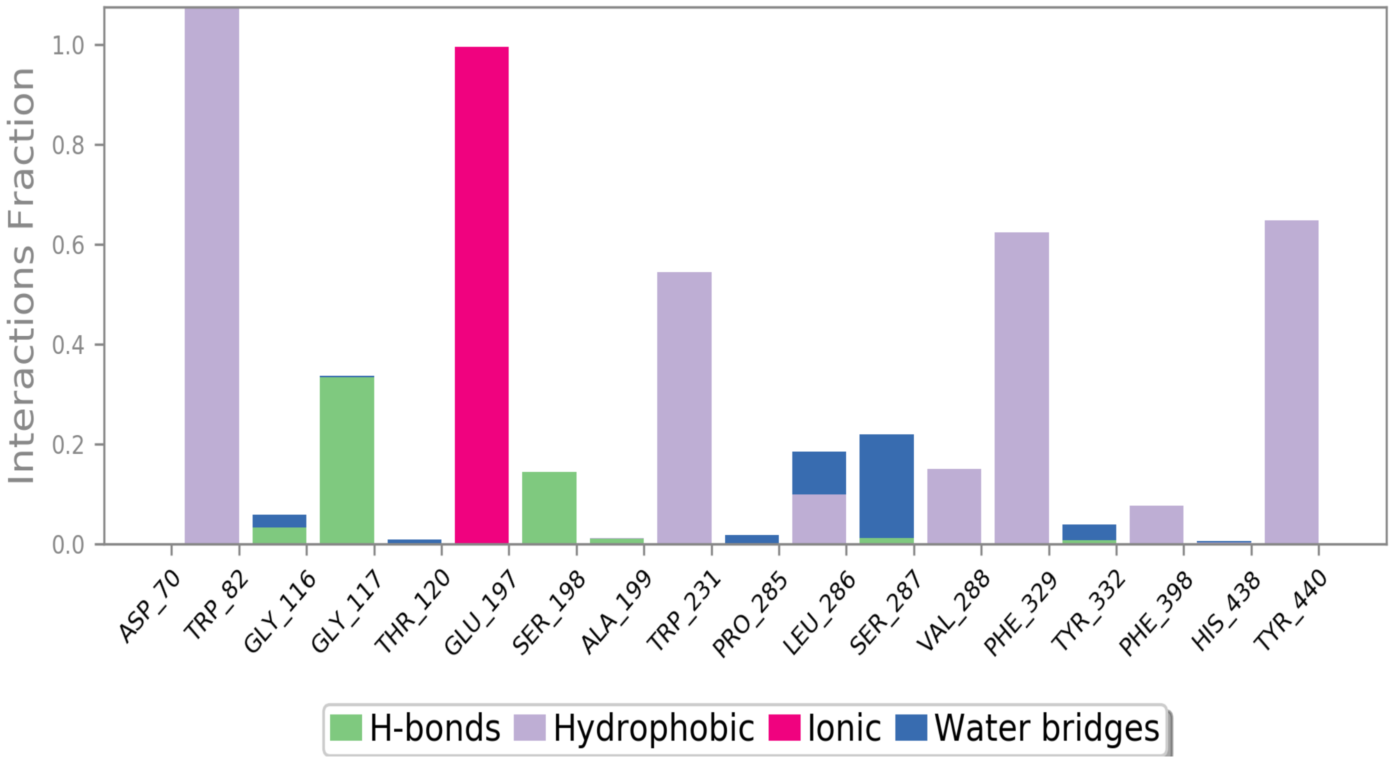

Protein interactions with the ligand can be monitored throughout the simulation. These interactions can be categorized by type and summarized, as shown in the plot above. Protein-ligand interactions (or 'contacts') are categorized into four types: Hydrogen Bonds, Hydrophobic, Ionic and Water Bridges. Each interaction type contains more specific subtypes, which can be explored through the 'Simulation Interactions Diagram' panel. The stacked bar charts are normalized over the course of the trajectory: for example, a value of 0.7 suggests that 70% of the simulation time the specific interaction is maintained. Values over 1.0 are possible as some protein residue may make multiple contacts of same subtype with the ligand.

Hydrogen Bonds: (H-bonds) play a significant role in ligand binding. Consideration of hydrogen-bonding properties in drug design is important because of their strong influence on drug specificity, metabolization and adsorption. Hydrogen bonds between a protein and a ligand can be further broken down into four subtypes: backbone acceptor; backbone donor; side-chain acceptor; side-chain donor.
The current geometric criteria for protein-ligand H-bond is: distance of 2.5 Å between the donor and acceptor atoms (D—H···A); a donor angle of ≥120° between the donor-hydrogen-acceptor atoms (D—H···A); and an acceptor angle of ≥90° between the hydrogen-acceptor-bonded_atom atoms (H···A—X).

Hydrophobic contacts: fall into three subtypes: π-Cation; π-π; and Other, non-specific interactions. Generally these type of interactions involve a hydrophobic amino acid and an aromatic or aliphatic group on the ligand, but we have extended this category to also include π-Cation interactions.
The current geometric criteria for hydrophobic interactions is as follows: π-Cation — Aromatic and charged groups within 4.5 Å; π-π — Two aromatic groups stacked face-to-face or face-to-edge; Other — A non-specific hydrophobic sidechain within 3.6 Å of a ligand's aromatic or aliphatic carbons.

Ionic interactions: or polar interactions, are between two oppositely charged atoms that are within 3.7 Å of each other and do not involve a hydrogen bond. We also monitor Protein-Metal-Ligand interactions, which are defined by a metal ion coordinated within 3.4 Å of protein's and ligand's heavy atoms (except carbon). All ionic interactions are broken down into two subtypes: those mediated by a protein backbone or side chains.

Water Bridges: are hydrogen-bonded protein-ligand interactions mediated by a water molecule. The hydrogen-bond geometry is slightly relaxed from the standard H-bond definition.
The current geometric criteria for a protein-water or water-ligand H-bond are: a distance of 2.8 Å between the donor and acceptor atoms (D—H···A); a donor angle of ≥110° between the donor-hydrogen-acceptor atoms (D—H···A); and an acceptor angle of ≥90° between the hydrogen-acceptor-bonded_atom atoms (H···A—X).

Protein-Ligand Contacts (cont.)

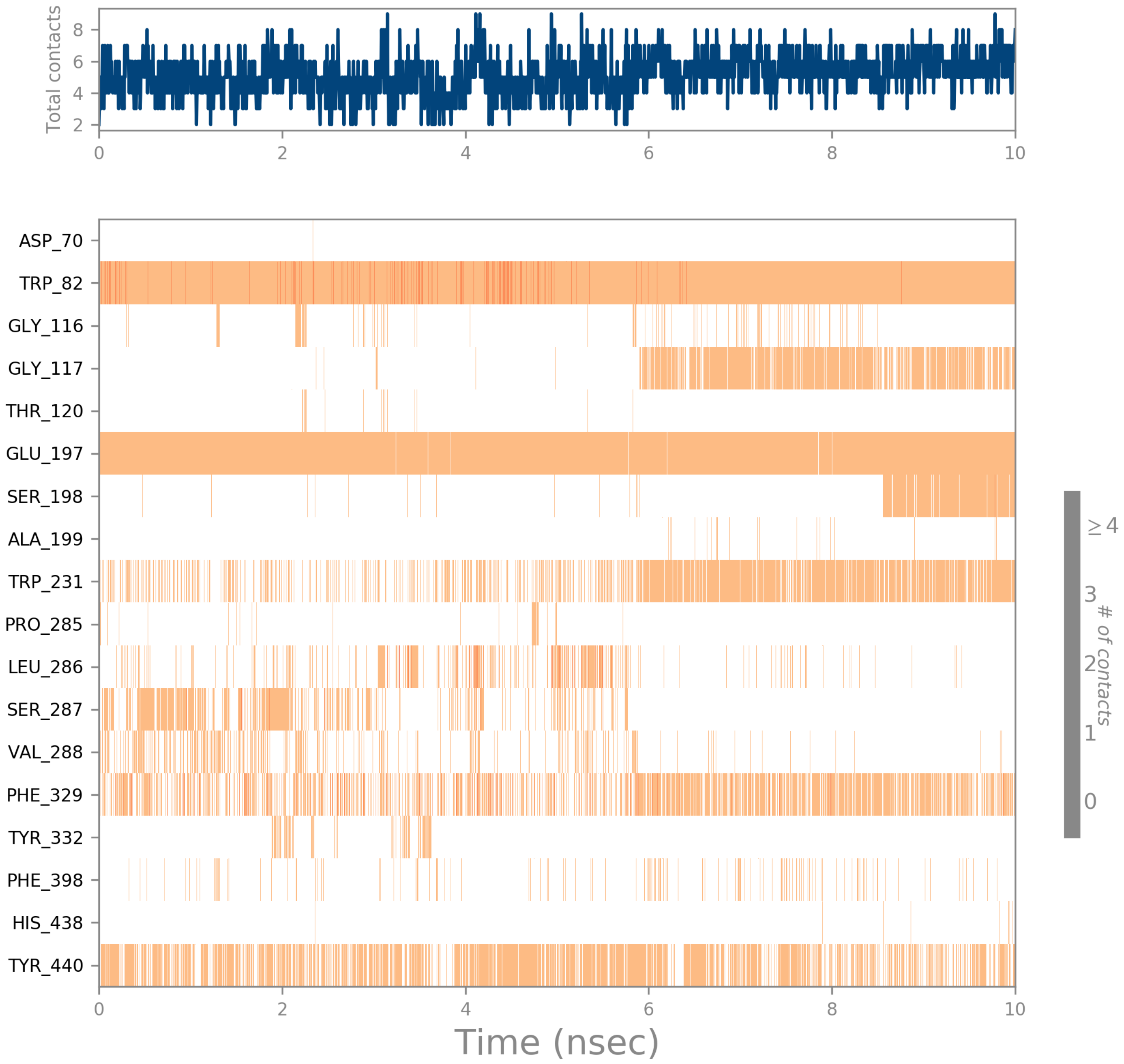

A timeline representation of the interactions and contacts (**H-bonds**, **Hydrophobic**, **Ionic**, **Water bridges**) summarized in the previous page. The top panel shows the total number of specific contacts the protein makes with the ligand over the course of the trajectory. The bottom panel shows which residues interact with the ligand in each trajectory frame. Some residues make more than one specific contact with the ligand, which is represented by a darker shade of orange, according to the scale to the right of the plot.

Ligand-Protein Contacts

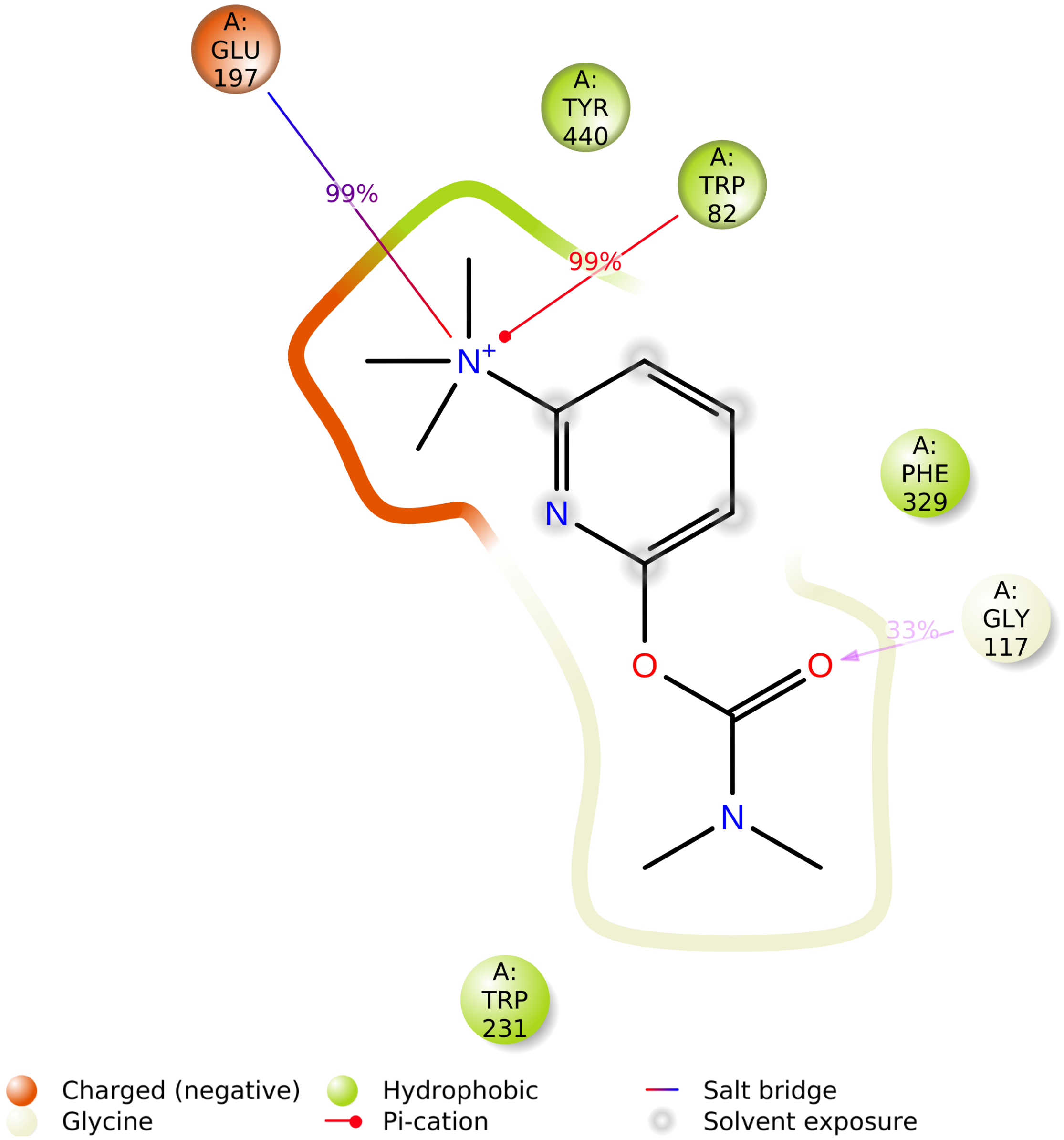

A schematic of detailed ligand atom interactions with the protein residues. Interactions that occur more than **30.0%** of the simulation time in the selected trajectory (0.00 through 10.00 nsec), are shown.
Note: it is possible to have interactions with >100% as some residues may have multiple interactions of a single type with the same ligand atom. For example, the ARG side chain has four H-bond donors that can all hydrogen-bond to a single H-bond acceptor.

Ligand Torsion Profile

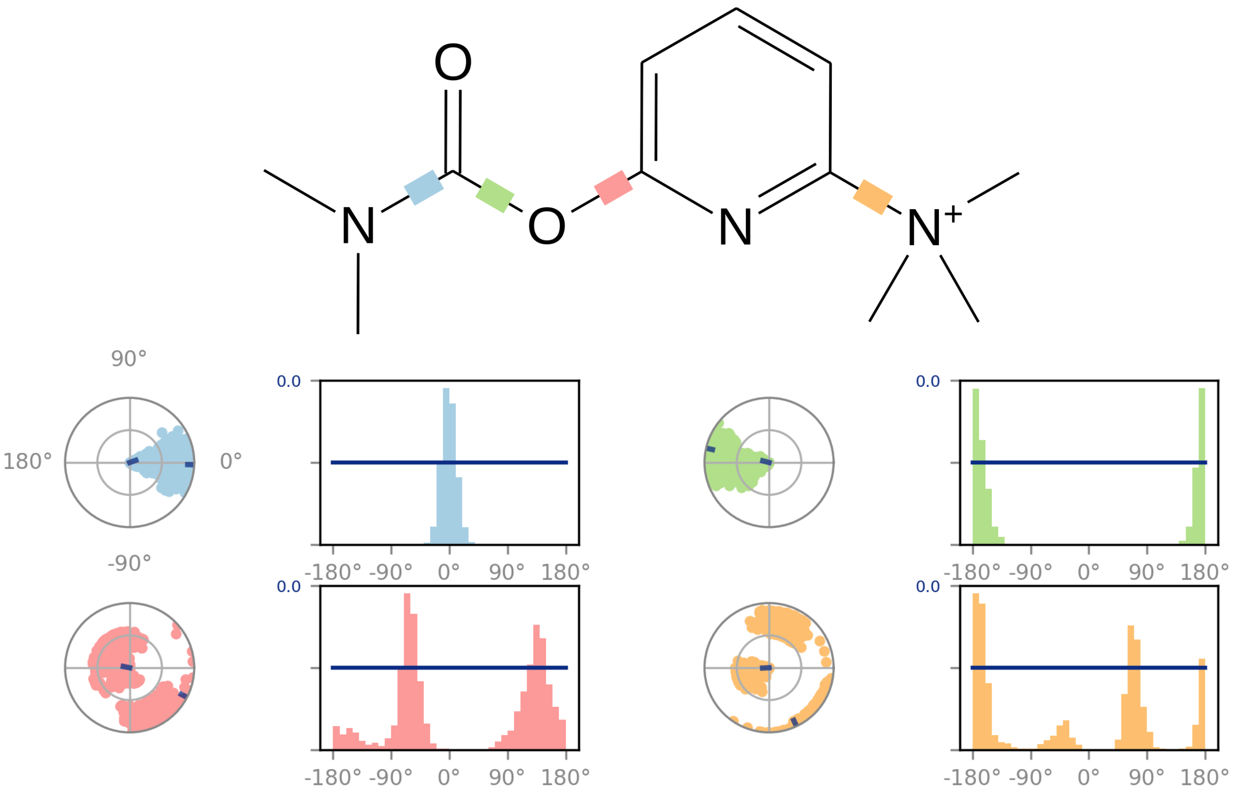

The ligand torsions plot summarizes the conformational evolution of every rotatable bond (RB) in the ligand throughout the simulation trajectory (0.00 through 10.00 nsec). The top panel shows the 2d schematic of a ligand with color-coded rotatable bonds. Each rotatable bond torsion is accompanied by a dial plot and bar plots of the same color.

Dial (or radial) plots describe the conformation of the torsion throughout the course of the simulation. The beginning of the simulation is in the center of the radial plot and the time evolution is plotted radially outwards.

The bar plots summarize the data on the dial plots, by showing the probability density of the torsion. If torsional potential information is available, the plot also shows the potential of the rotatable bond (by summing the potential of the related torsions). The values of the potential are on the left *Y*-axis of the chart, and are expressed in *kcal/mol*. Looking at the histogram and torsion potential relationships may give insights into the conformational strain the ligand undergoes to maintain a protein-bound conformation.

Ligand Properties

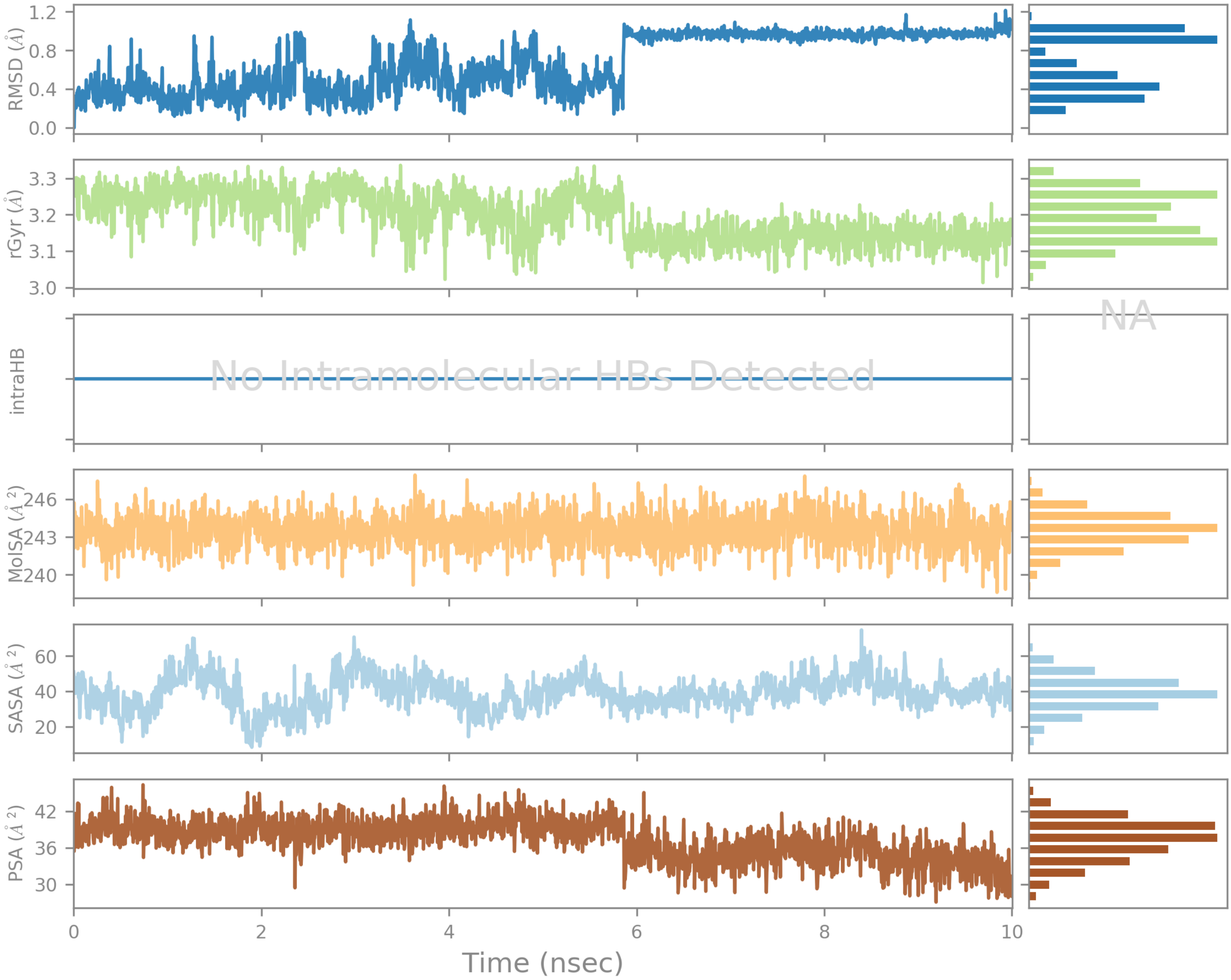

Ligand RMSD: Root mean square deviation of a ligand with respect to the reference conformation (typically the first frame is used as the reference and it is regarded as time *t*=0).

Radius of Gyration (rGyr): Measures the 'extendedness' of a ligand, and is equivalent to its principal moment of inertia.

Intramolecular Hydrogen Bonds (intraHB): Number of internal hydrogen bonds (HB) within a ligand molecule.

Molecular Surface Area (MolSA): Molecular surface calculation with 1.4 Å probe radius. This value is equivalent to a van der Waals surface area.

Solvent Accessible Surface Area (SASA): Surface area of a molecule accessible by a water molecule.

Polar Surface Area (PSA): Solvent accessible surface area in a molecule contributed only by oxygen and nitrogen atoms.

Report S8
MD Simulation Report on BChE - Lindoldhamine Isomer Interactions

Simulation Details

Jobname: md_job_6EP4_1_dock-1
Entry title: 6EP4_1_dock_1

CPU #	Job Type	Ensemble	Temp. [K]	Sim. Time [ns]	# Atoms	# Waters	Charge
1	mdsim	NPT	300.0	10.005	50973	14166	0

Protein Information

Tot. Residues	Prot. Chain(s)	Res. in Chain(s)	# Atoms	# Heavy Atoms	Charge
527	'NoChainId'	ict_values([527]	8313	4203	+2

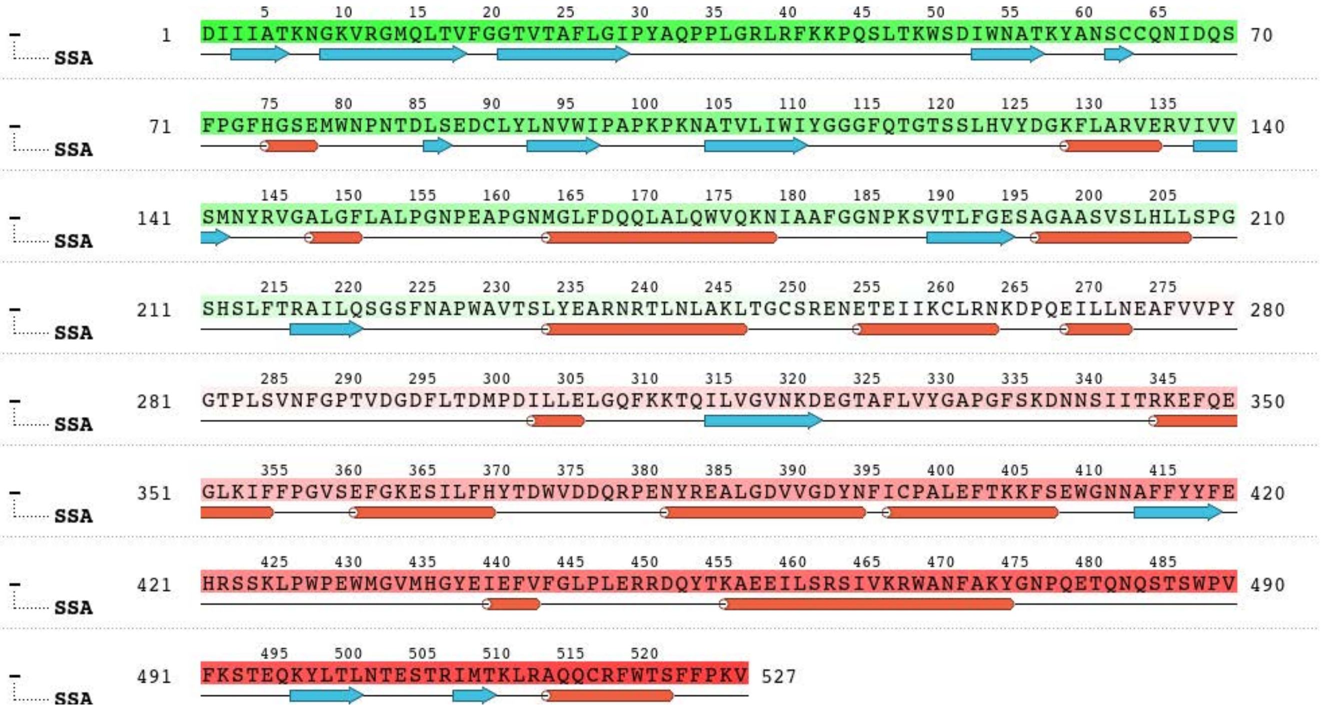

Ligand Information

SMILES	COc(c(c1)O)cc(c12)CC[NH2+][C@H]2Cc3cc(c(O)cc3)Oc(c4)c(OC)cc(c45)CC[NH2+][C@H]5Cc6ccc(O)cc6	
PDB Name	'UNK'	
Num. of Atoms	80 (total) 42 (heavy)	
Atomic Mass	570.692 au	
Charge	+2	
Mol. Formula	C34H38N2O6	
Num. of Fragments	3	
Num. of Rot. Bonds	11	

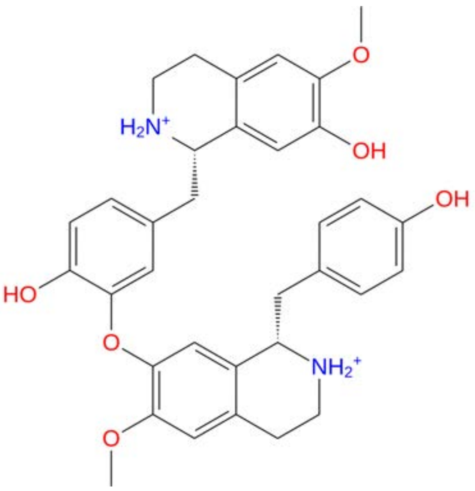

Counter Ion/Salt Information

Type	Num.	Concentration [mM]	Total Charge
Cl	43	55.190	-43
Na	39	50.056	+39

Protein-Ligand RMSD

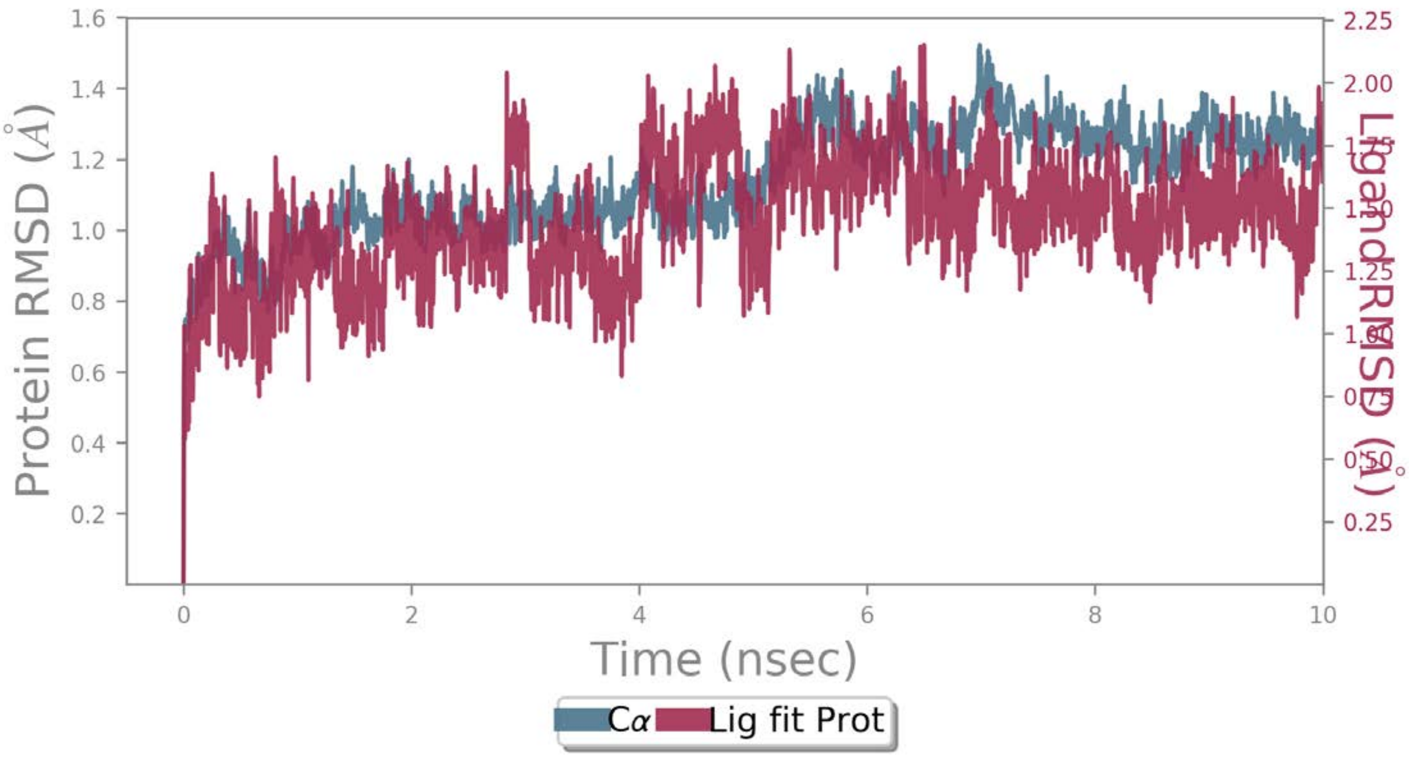

The Root Mean Square Deviation (RMSD) is used to measure the average change in displacement of a selection of atoms for a particular frame with respect to a reference frame. It is calculated for all frames in the trajectory. The RMSD for frame *x* is:

$$RMSD_x = \sqrt{\frac{1}{N}\sum_{i=1}^{N}(r_i'(t_x)) - r_i(t_{ref}))^2}$$

where *N* is the number of atoms in the atom selection; t_{ref} is the reference time, (typically the first frame is used as the reference and it is regarded as time *t*=0); and *r'* is the position of the selected atoms in frame *x* after superimposing on the reference frame, where frame *x* is recorded at time t_x. The procedure is repeated for every frame in the simulation trajectory.

Protein RMSD: The above plot shows the RMSD evolution of a protein (left *Y*-axis). All protein frames are first aligned on the reference frame backbone, and then the RMSD is calculated based on the atom selection. Monitoring the RMSD of the protein can give insights into its structural conformation throughout the simulation. RMSD analysis can indicate if the simulation has equilibrated — its fluctuations towards the end of the simulation are around some thermal average structure. Changes of the order of 1-3 Å are perfectly acceptable for small, globular proteins. Changes much larger than that, however, indicate that the protein is undergoing a large conformational change during the simulation. It is also important that your simulation converges — the RMSD values stabilize around a fixed value. If the RMSD of the protein is still increasing or decreasing on average at the end of the simulation, then your system has not equilibrated, and your simulation may not be long enough for rigorous analysis.

Ligand RMSD: Ligand RMSD (right *Y*-axis) indicates how stable the ligand is with respect to the protein and its binding pocket. In the above plot, 'Lig fit Prot' shows the RMSD of a ligand when the protein-ligand complex is first aligned on the protein backbone of the reference and then the RMSD of the ligand heavy atoms is measured. If the values observed are significantly larger than the RMSD of the protein, then it is likely that the ligand has diffused away from its initial binding site.

Protein RMSF

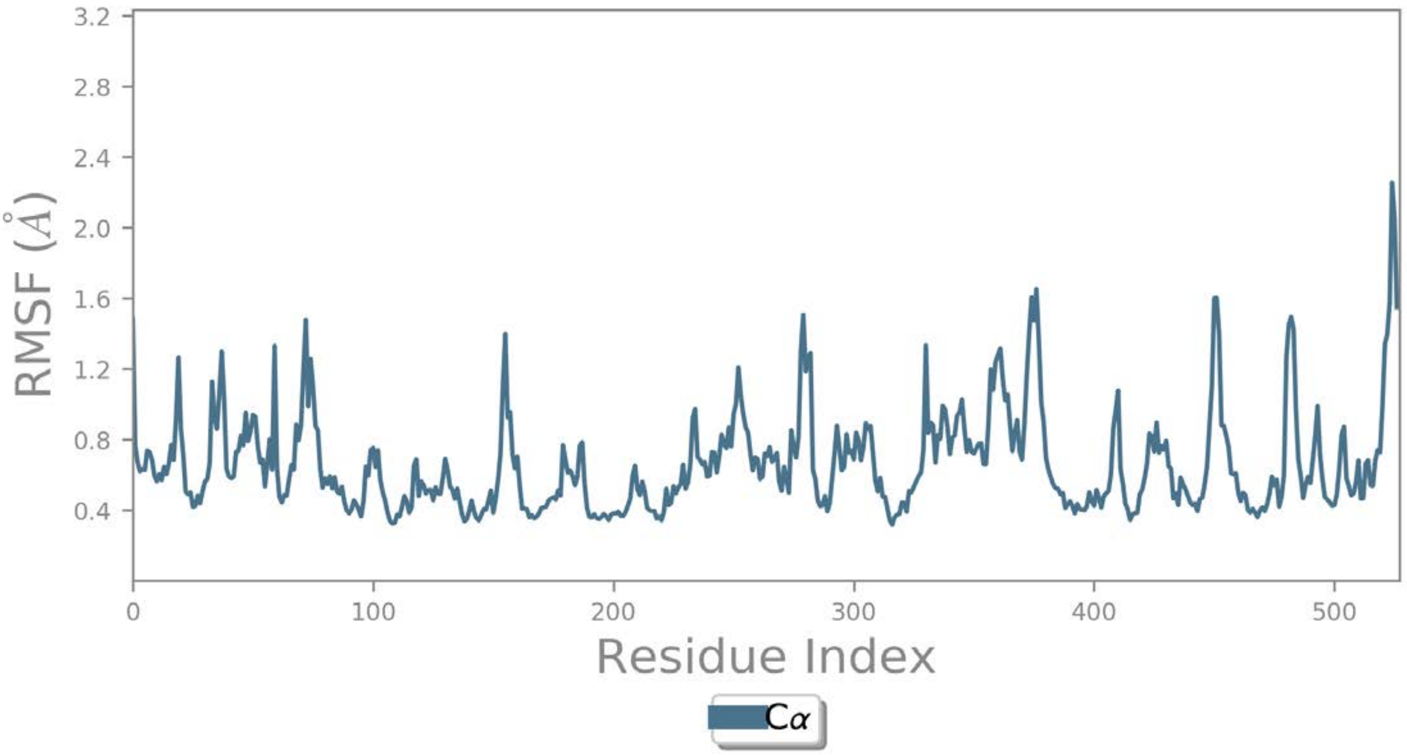

The Root Mean Square Fluctuation (RMSF) is useful for characterizing local changes along the protein chain. The RMSF for residue *i* is:

$$RMSF_i = \sqrt{\frac{1}{T}\sum_{t=1}^{T} < (r'_i(t)) - r_i(t_{ref}))^2 >}$$

where T is the trajectory time over which the RMSF is calculated, t_{ref} is the reference time, r_i is the position of residue i; r' is the position of atoms in residue i after superposition on the reference, and the angle brackets indicate that the average of the square distance is taken over the selection of atoms in the residue.

On this plot, peaks indicate areas of the protein that fluctuate the most during the simulation. Typically you will observe that the tails (*N*- and *C*-terminal) fluctuate more than any other part of the protein. Secondary structure elements like alpha helices and beta strands are usually more rigid than the unstructured part of the protein, and thus fluctuate less than the loop regions.

Protein Secondary Structure

% Helix	% Strand	% Total SSE
28.64	13.79	42.43

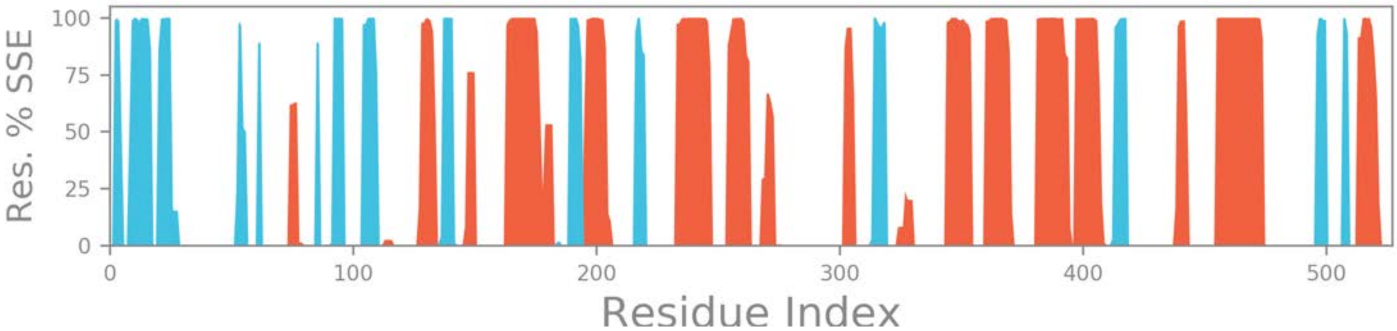

Protein secondary structure elements (SSE) like alpha-helices and beta-strands are monitored throughout the simulation. The plot above reports SSE distribution by residue index throughout the protein structure. The plot below summarizes the SSE composition for each trajectory frame over the course of the simulation, and the plot at the bottom monitors each residue and its SSE assignment over time.

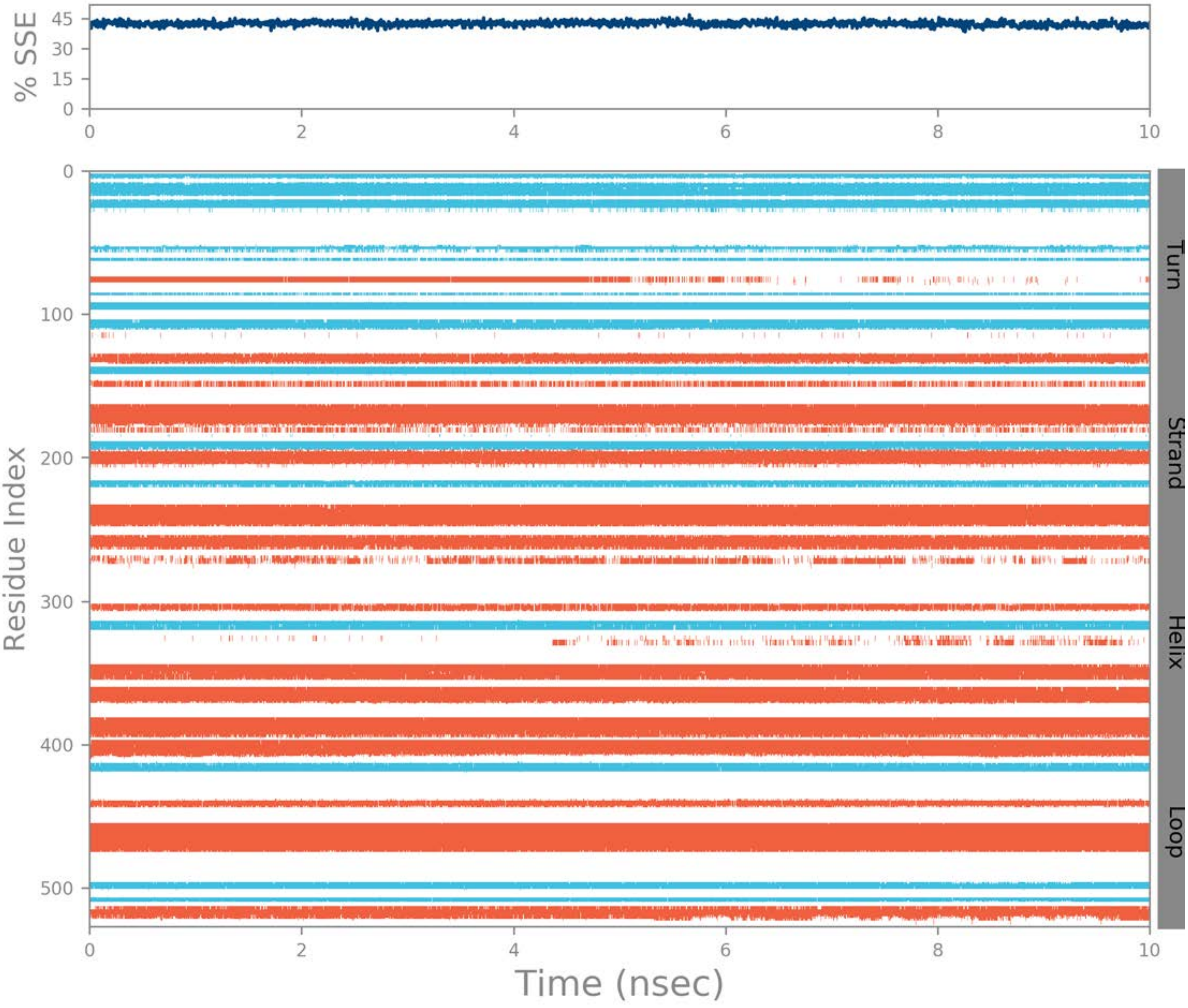

RMSF of Lindholdamine Isomer Ligand

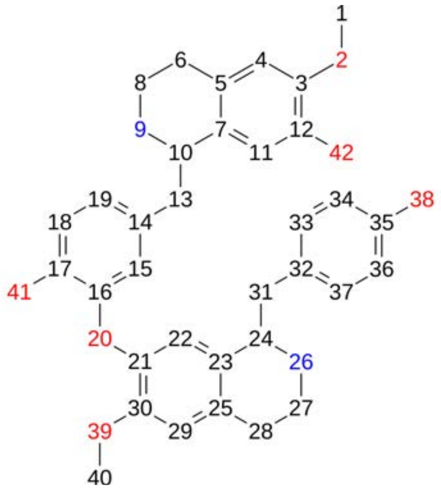

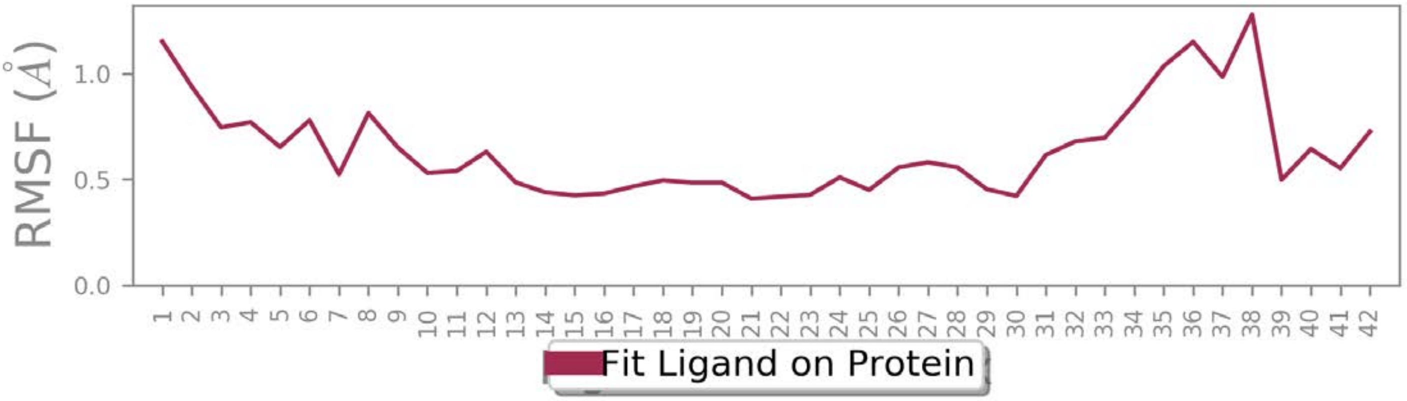

The Ligand Root Mean Square Fluctuation (L-RMSF) is useful for characterizing changes in the ligand atom positions. The RMSF for atom *i* is:

$$RMSF_i = \sqrt{\frac{1}{T}\sum_{t=1}^{T}(r_i'(t)) - r_i(t_{ref}))^2}$$

where *T* is the trajectory time over which the RMSF is calculated, t_{ref} is the reference time (usually for the first frame, and is regarded as the *zero* of time); *r* is the position of atom *i* in the reference at time t_{ref}, and *r'* is the position of atom *i* at time *t* after superposition on the reference frame.

Ligand RMSF shows the ligand's fluctuations broken down by atom, corresponding to the 2D structure in the top panel. The ligand RMSF may give you insights on how ligand fragments interact with the protein and their entropic role in the binding event. In the bottom panel, the 'Fit Ligand on Protein' line shows the ligand fluctuations, with respect to the protein. The protein-ligand complex is first aligned on the protein backbone and then the ligand RMSF is measured on the ligand heavy atoms.

Protein-Ligand Contacts

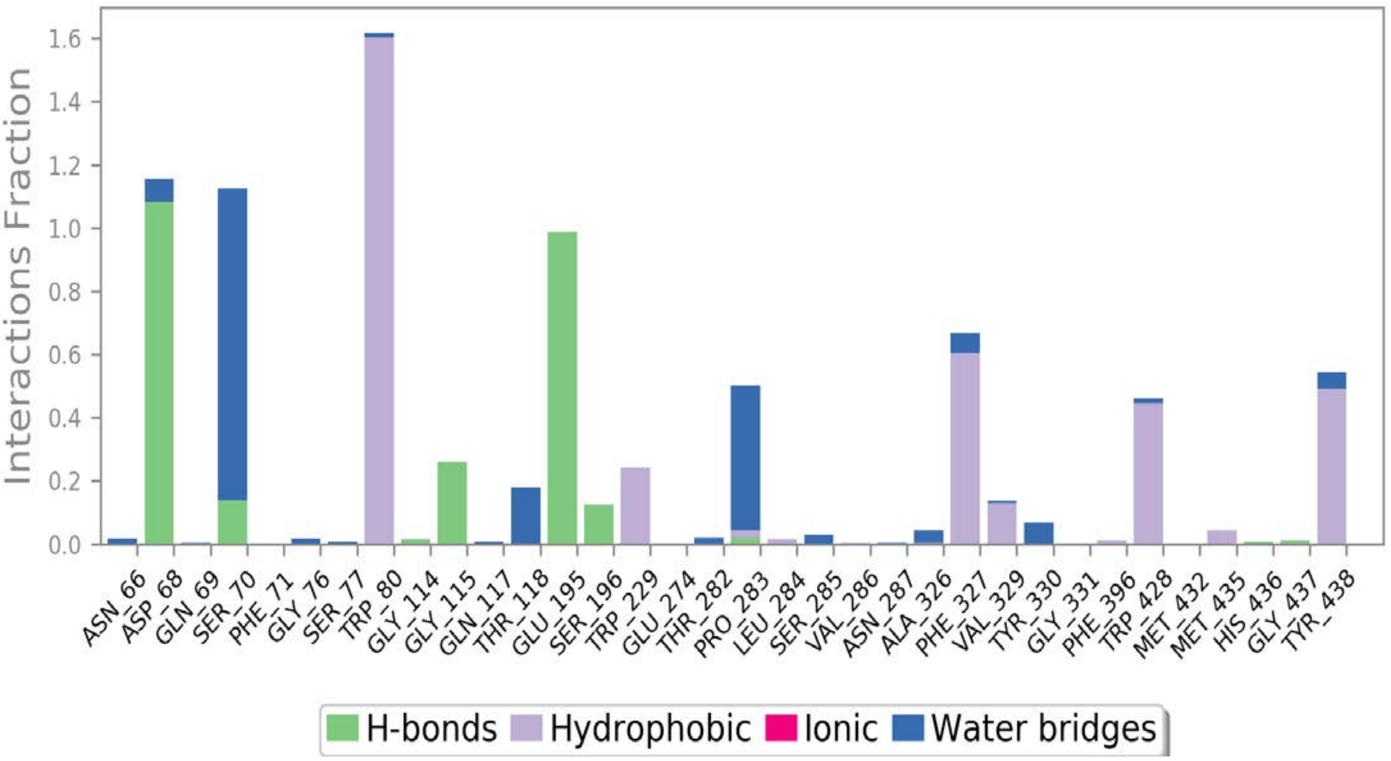

Protein interactions with the ligand can be monitored throughout the simulation. These interactions can be categorized by type and summarized, as shown in the plot above. Protein-ligand interactions (or 'contacts') are categorized into four types: Hydrogen Bonds, Hydrophobic, Ionic and Water Bridges. Each interaction type contains more specific subtypes, which can be explored through the 'Simulation Interactions Diagram' panel. The stacked bar charts are normalized over the course of the trajectory: for example, a value of 0.7 suggests that 70% of the simulation time the specific interaction is maintained. Values over 1.0 are possible as some protein residue may make multiple contacts of same subtype with the ligand.

Hydrogen Bonds: (H-bonds) play a significant role in ligand binding. Consideration of hydrogen-bonding properties in drug design is important because of their strong influence on drug specificity, metabolization and adsorption. Hydrogen bonds between a protein and a ligand can be further broken down into four subtypes: backbone acceptor; backbone donor; side-chain acceptor; side-chain donor.
The current geometric criteria for protein-ligand H-bond is: distance of 2.5 Å between the donor and acceptor atoms (D—H···A); a donor angle of ≥120° between the donor-hydrogen-acceptor atoms (D—H···A); and an acceptor angle of ≥90° between the hydrogen-acceptor-bonded_atom atoms (H···A—X).

Hydrophobic contacts: fall into three subtypes: π-Cation; π-π; and Other, non-specific interactions. Generally these type of interactions involve a hydrophobic amino acid and an aromatic or aliphatic group on the ligand, but we have extended this category to also include π-Cation interactions.
The current geometric criteria for hydrophobic interactions is as follows: π-Cation — Aromatic and charged groups within 4.5 Å; π-π — Two aromatic groups stacked face-to-face or face-to-edge; Other — A non-specific hydrophobic sidechain within 3.6 Å of a ligand's aromatic or aliphatic carbons.

Ionic interactions: or polar interactions, are between two oppositely charged atoms that are within 3.7 Å of each other and do not involve a hydrogen bond. We also monitor Protein-Metal-Ligand interactions, which are defined by a metal ion coordinated within 3.4 Å of protein's and ligand's heavy atoms (except carbon). All ionic interactions are broken down into two subtypes: those mediated by a protein backbone or side chains.

Water Bridges: are hydrogen-bonded protein-ligand interactions mediated by a water molecule. The hydrogen-bond geometry is slightly relaxed from the standard H-bond definition.
The current geometric criteria for a protein-water or water-ligand H-bond are: a distance of 2.8 Å between the donor and acceptor atoms (D—H···A); a donor angle of ≥110° between the donor-hydrogen-acceptor atoms (D—H···A); and an acceptor angle of ≥90° between the hydrogen-acceptor-bonded_atom atoms (H···A—X).

Protein-Ligand Contacts (cont.)

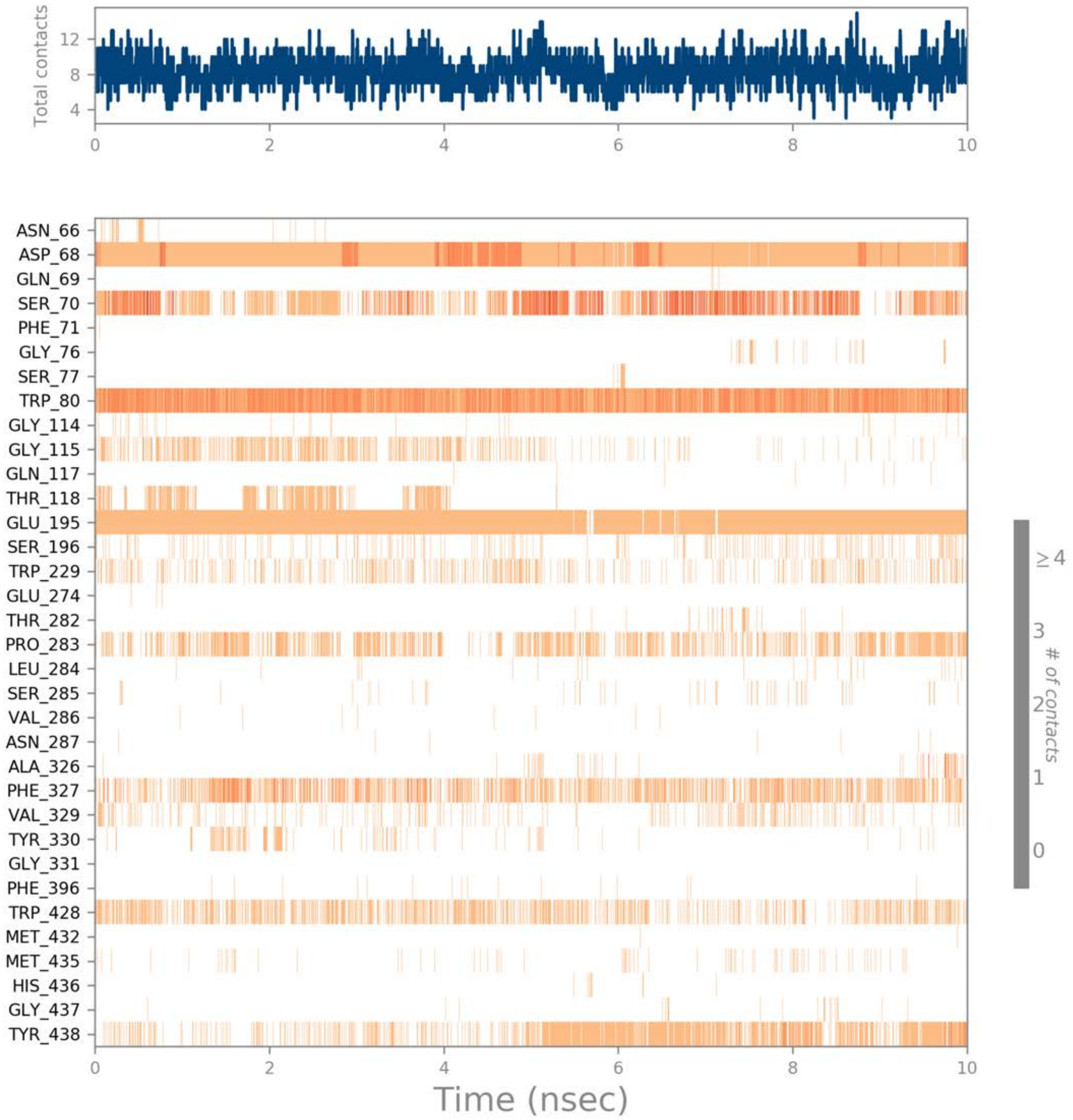

A timeline representation of the interactions and contacts (**H-bonds**, **Hydrophobic**, **Ionic**, **Water bridges**) summarized in the previous page. The top panel shows the total number of specific contacts the protein makes with the ligand over the course of the trajectory. The bottom panel shows which residues interact with the ligand in each trajectory frame. Some residues make more than one specific contact with the ligand, which is represented by a darker shade of orange, according to the scale to the right of the plot.

Ligand-Protein Contacts

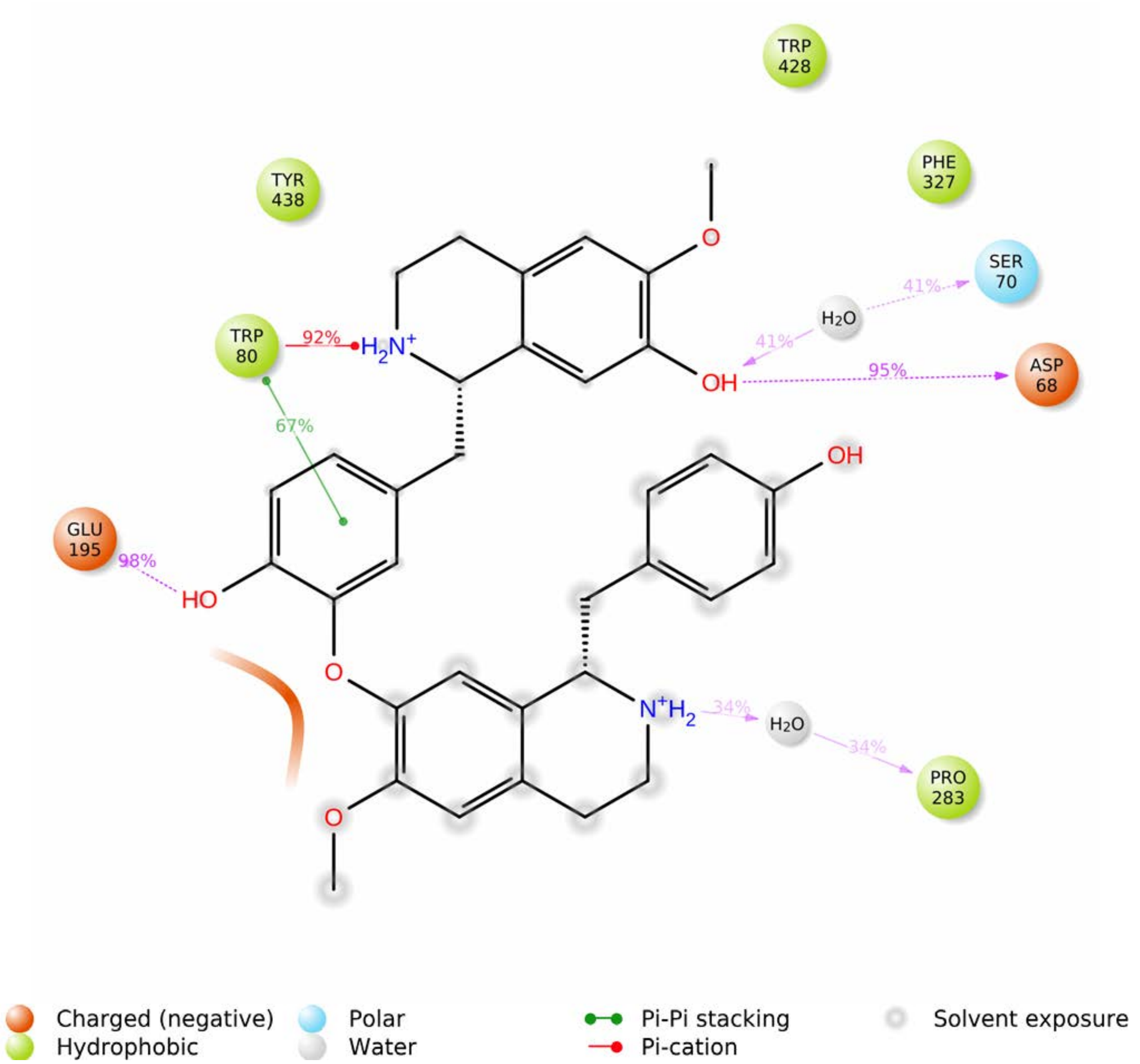

A schematic of detailed ligand atom interactions with the protein residues. Interactions that occur more than **30.0%** of the simulation time in the selected trajectory (0.00 through 10.00 nsec), are shown.
Note: it is possible to have interactions with >100% as some residues may have multiple interactions of a single type with the same ligand atom. For example, the ARG side chain has four H-bond donors that can all hydrogen-bond to a single H-bond acceptor.

Ligand Torsion Profile

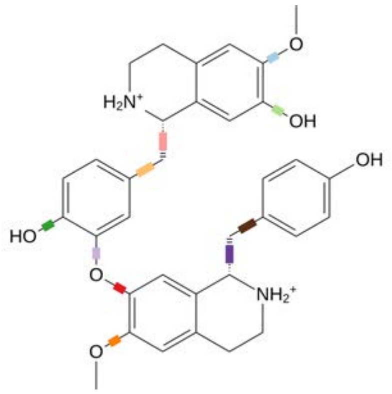

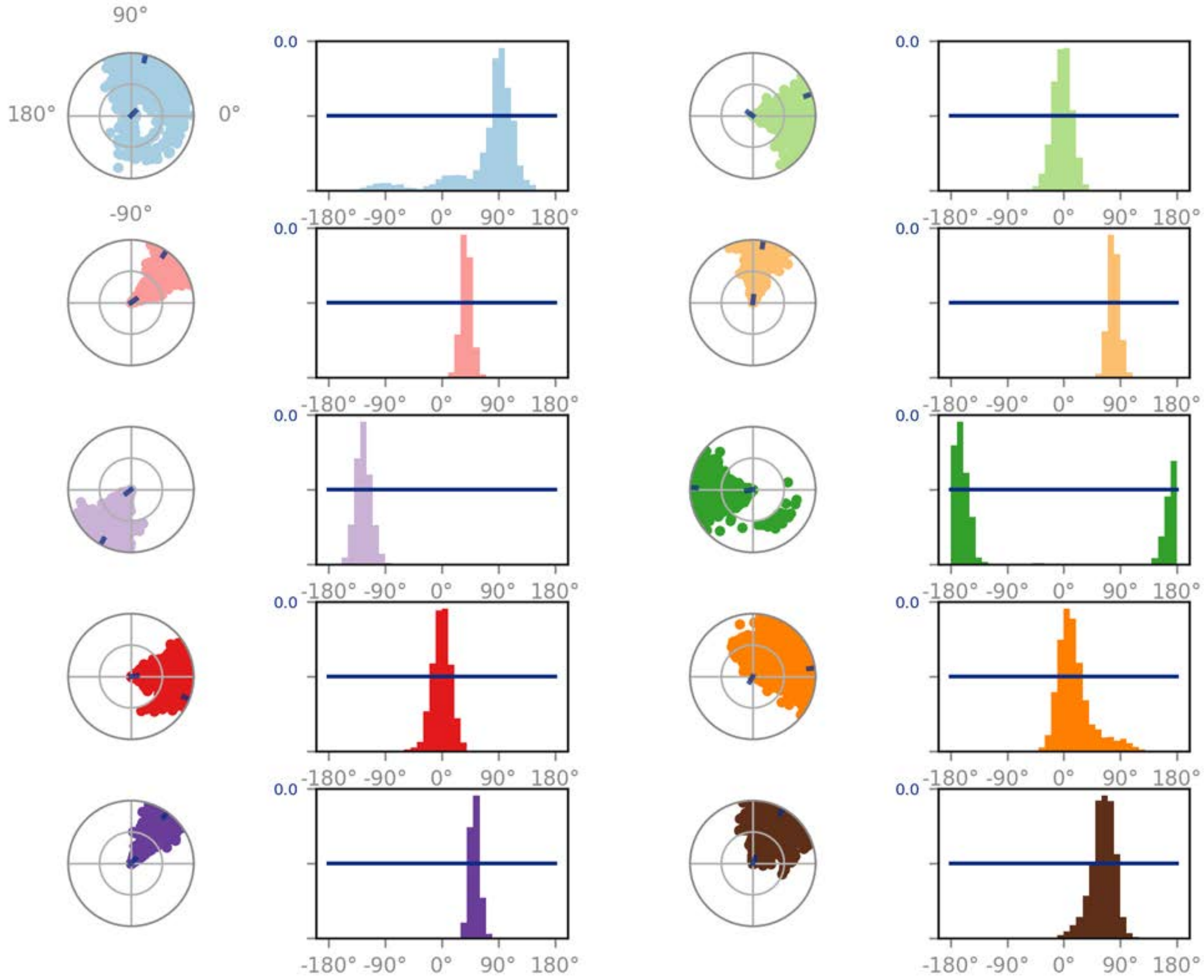

Ligand Torsion Profile (cont.)

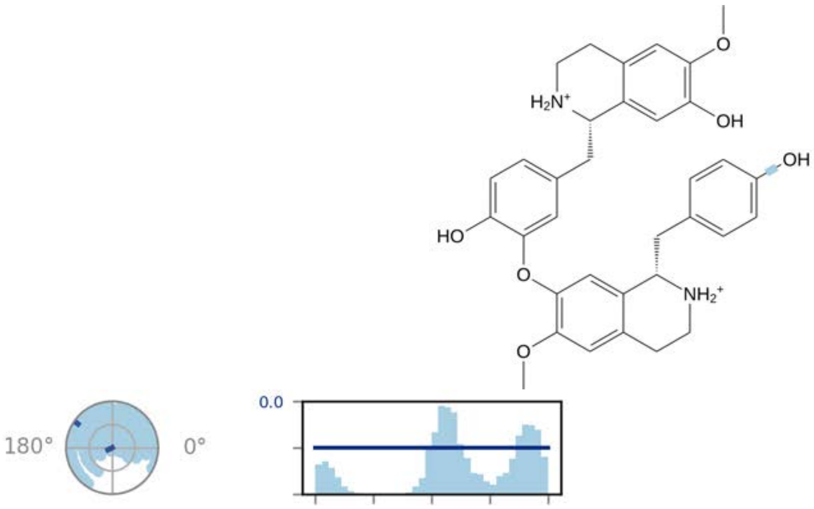

The ligand torsions plot summarizes the conformational evolution of every rotatable bond (RB) in the ligand throughout the simulation trajectory (0.00 through 10.00 nsec). The top panel shows the 2d schematic of a ligand with color-coded rotatable bonds. Each rotatable bond torsion is accompanied by a dial plot and bar plots of the same color.

Dial (or radial) plots describe the conformation of the torsion throughout the course of the simulation. The beginning of the simulation is in the center of the radial plot and the time evolution is plotted radially outwards.

The bar plots summarize the data on the dial plots, by showing the probability density of the torsion. If torsional potential information is available, the plot also shows the potential of the rotatable bond (by summing the potential of the related torsions). The values of the potential are on the left *Y*-axis of the chart, and are expressed in *kcal/mol*. Looking at the histogram and torsion potential relationships may give insights into the conformational strain the ligand undergoes to maintain a protein-bound conformation.

Ligand Properties

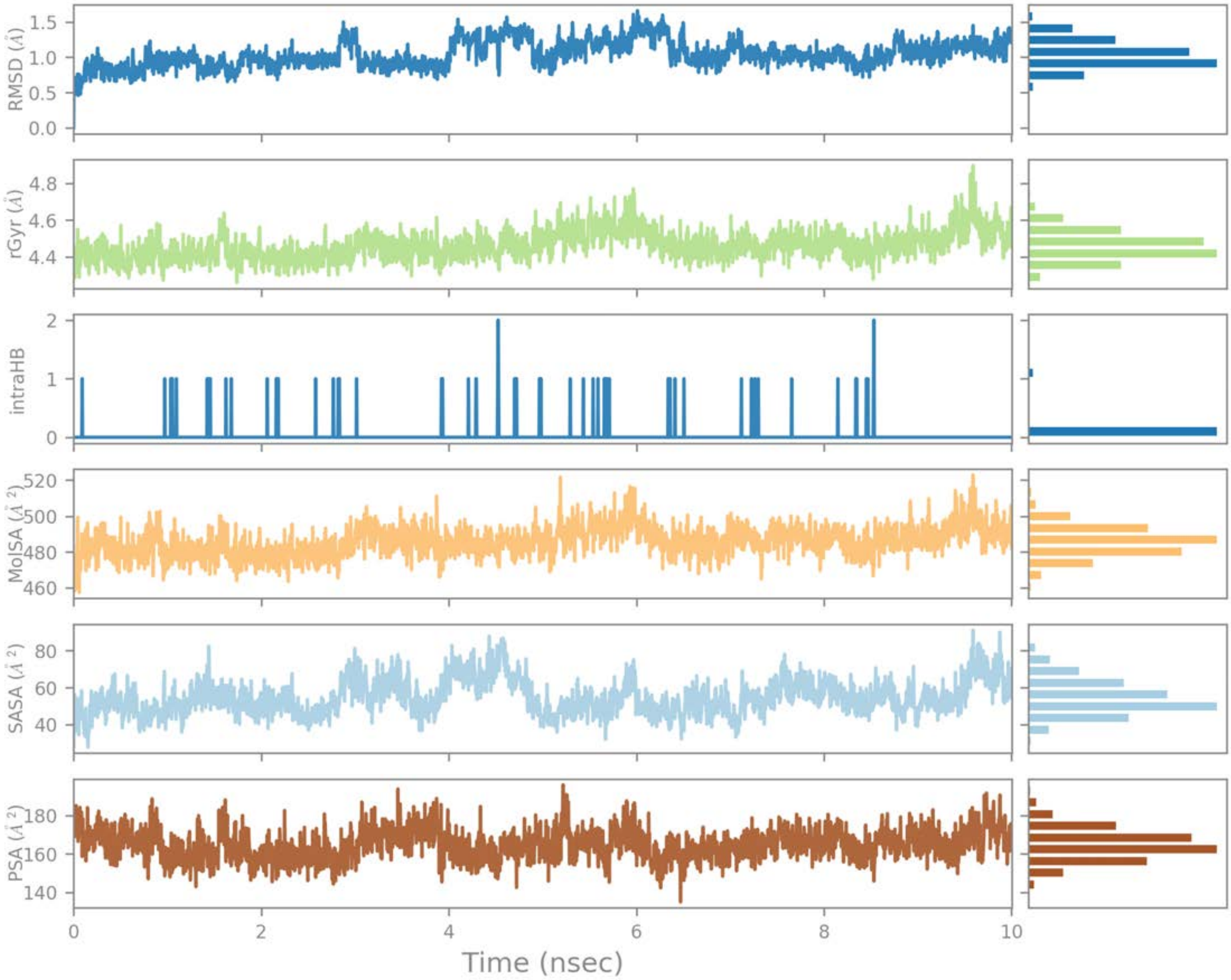

Ligand RMSD: Root mean square deviation of a ligand with respect to the reference conformation (typically the first frame is used as the reference and it is regarded as time *t*=0).

Radius of Gyration (rGyr): Measures the 'extendedness' of a ligand, and is equivalent to its principal moment of inertia.

Intramolecular Hydrogen Bonds (intraHB): Number of internal hydrogen bonds (HB) within a ligand molecule.

Molecular Surface Area (MolSA): Molecular surface calculation with 1.4 Å probe radius. This value is equivalent to a van der Waals surface area.

Solvent Accessible Surface Area (SASA): Surface area of a molecule accessible by a water molecule.

Polar Surface Area (PSA): Solvent accessible surface area in a molecule contributed only by oxygen and nitrogen atoms.

Report S9
MD Simulation Report on BChE - Stepharine Interactions

Simulation Details

Jobname: md_job_6EP4_2_dock-1
Entry title: 6EP4_2_dock_1

CPU #	Job Type	Ensemble	Temp. [K]	Sim. Time [ns]	# Atoms	# Waters	Charge
1	mdsim	NPT	300.0	10.005	50997	14187	0

Protein Information

Tot. Residues	Prot. Chain(s)	Res. in Chain(s)	# Atoms	# Heavy Atoms	Charge
527	'NoChainId'	ict_values([527]	8313	4203	+2

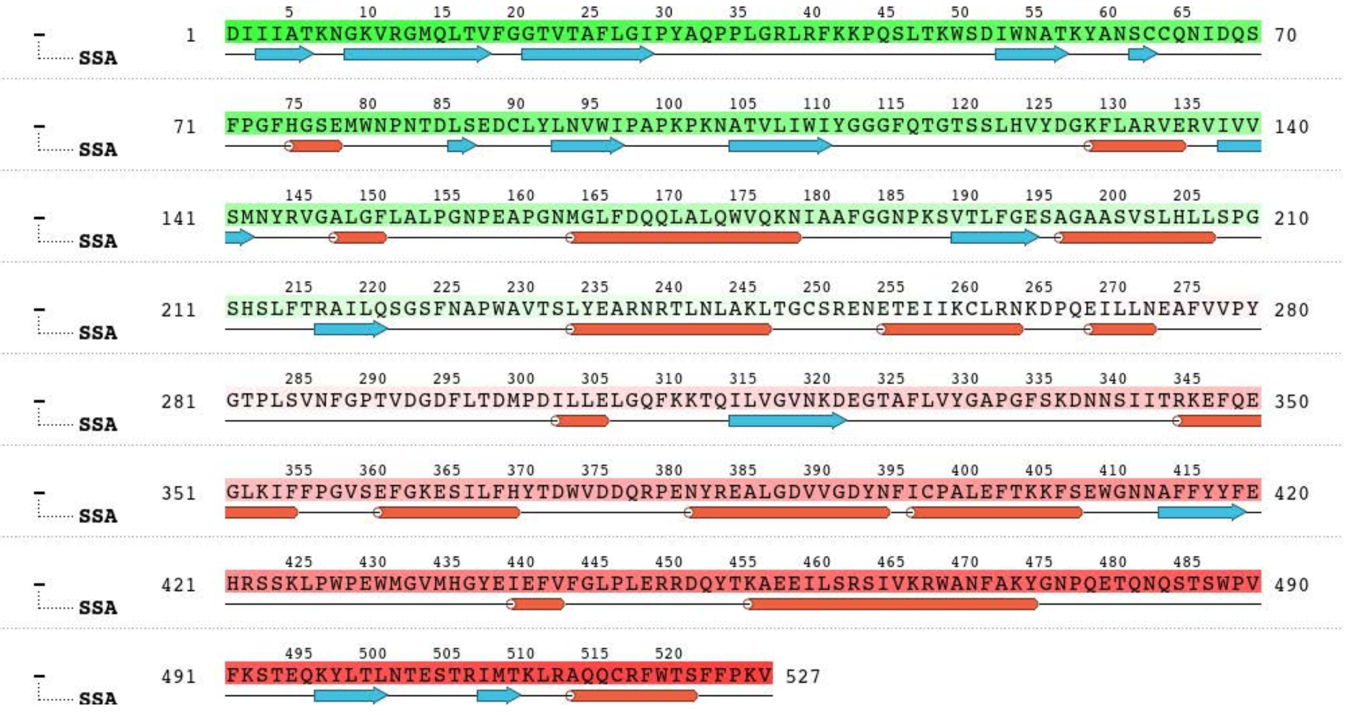

Ligand Information

SMILES	COc1c(OC)cc(CC[NH2+][C@H]2C3)c2c1C34C=CC(=O)C=C4
PDB Name	'UNK'
Num. of Atoms	42 (total) 22 (heavy)
Atomic Mass	298.365 au
Charge	+1
Mol. Formula	C18H20NO3
Num. of Fragments	1
Num. of Rot. Bonds	2

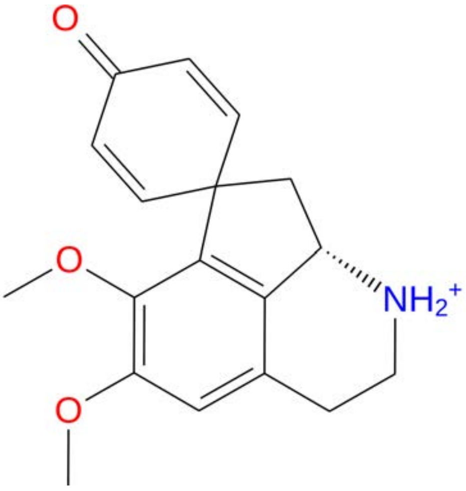

Counter Ion/Salt Information

Type	Num.	Concentration [mM]	Total Charge

Cl	42	53.826	-42
Na	39	49.982	+39

Protein-Ligand RMSD

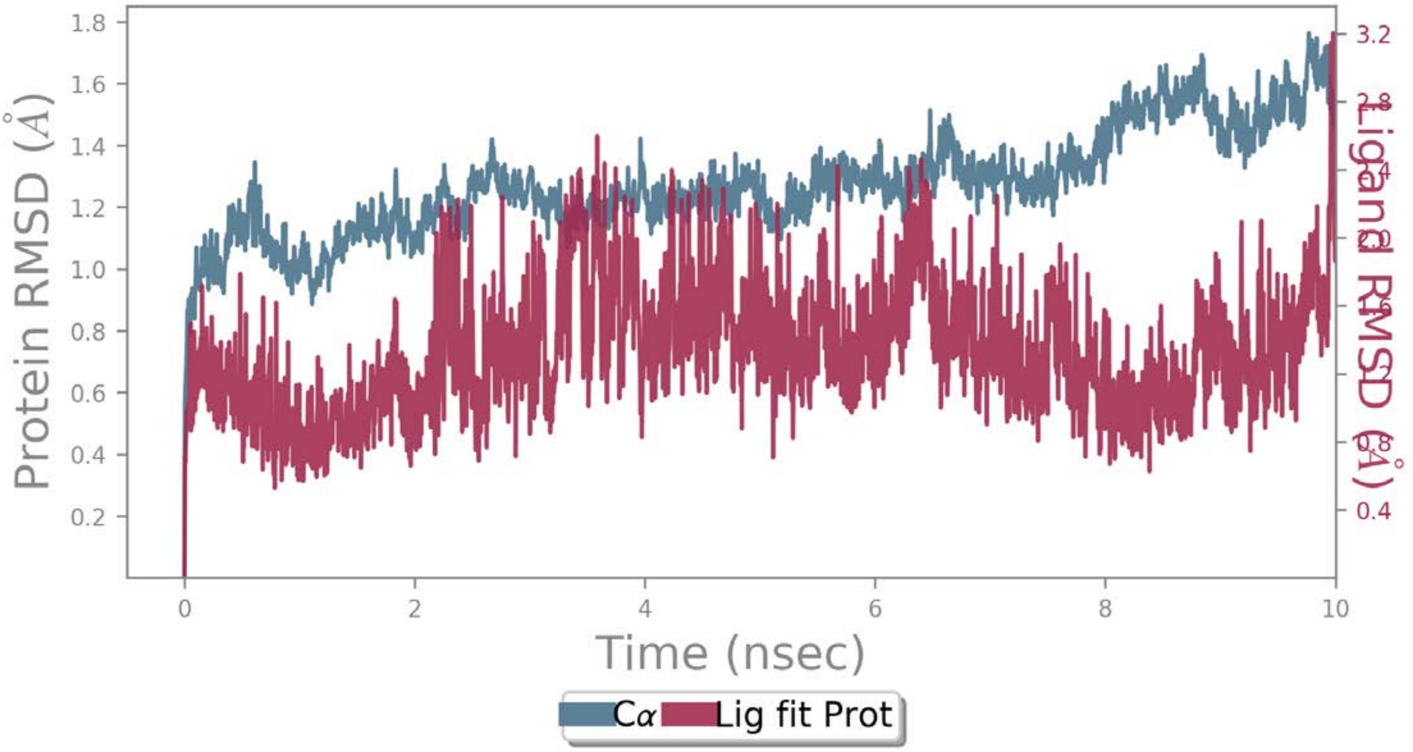

The Root Mean Square Deviation (RMSD) is used to measure the average change in displacement of a selection of atoms for a particular frame with respect to a reference frame. It is calculated for all frames in the trajectory. The RMSD for frame *x* is:

$$RMSD_x = \sqrt{\frac{1}{N}\sum_{i=1}^{N}(r'_i(t_x)) - r_i(t_{ref}))^2}$$

where *N* is the number of atoms in the atom selection; t_{ref} is the reference time, (typically the first frame is used as the reference and it is regarded as time *t*=0); and *r'* is the position of the selected atoms in frame *x* after superimposing on the reference frame, where frame *x* is recorded at time t_x. The procedure is repeated for every frame in the simulation trajectory.

Protein RMSD: The above plot shows the RMSD evolution of a protein (left *Y*-axis). All protein frames are first aligned on the reference frame backbone, and then the RMSD is calculated based on the atom selection. Monitoring the RMSD of the protein can give insights into its structural conformation throughout the simulation. RMSD analysis can indicate if the simulation has equilibrated — its fluctuations towards the end of the simulation are around some thermal average structure. Changes of the order of 1-3 Å are perfectly acceptable for small, globular proteins. Changes much larger than that, however, indicate that the protein is undergoing a large conformational change during the simulation. It is also important that your simulation converges — the RMSD values stabilize around a fixed value. If the RMSD of the protein is still increasing or decreasing on average at the end of the simulation, then your system has not equilibrated, and your simulation may not be long enough for rigorous analysis.

Ligand RMSD: Ligand RMSD (right *Y*-axis) indicates how stable the ligand is with respect to the protein and its binding pocket. In the above plot, 'Lig fit Prot' shows the RMSD of a ligand when the protein-ligand complex is first aligned on the protein backbone of the reference and then the RMSD of the ligand heavy atoms is measured. If the values observed are significantly larger than the RMSD of the protein, then it is likely that the ligand has diffused away from its initial binding site.

Protein RMSF

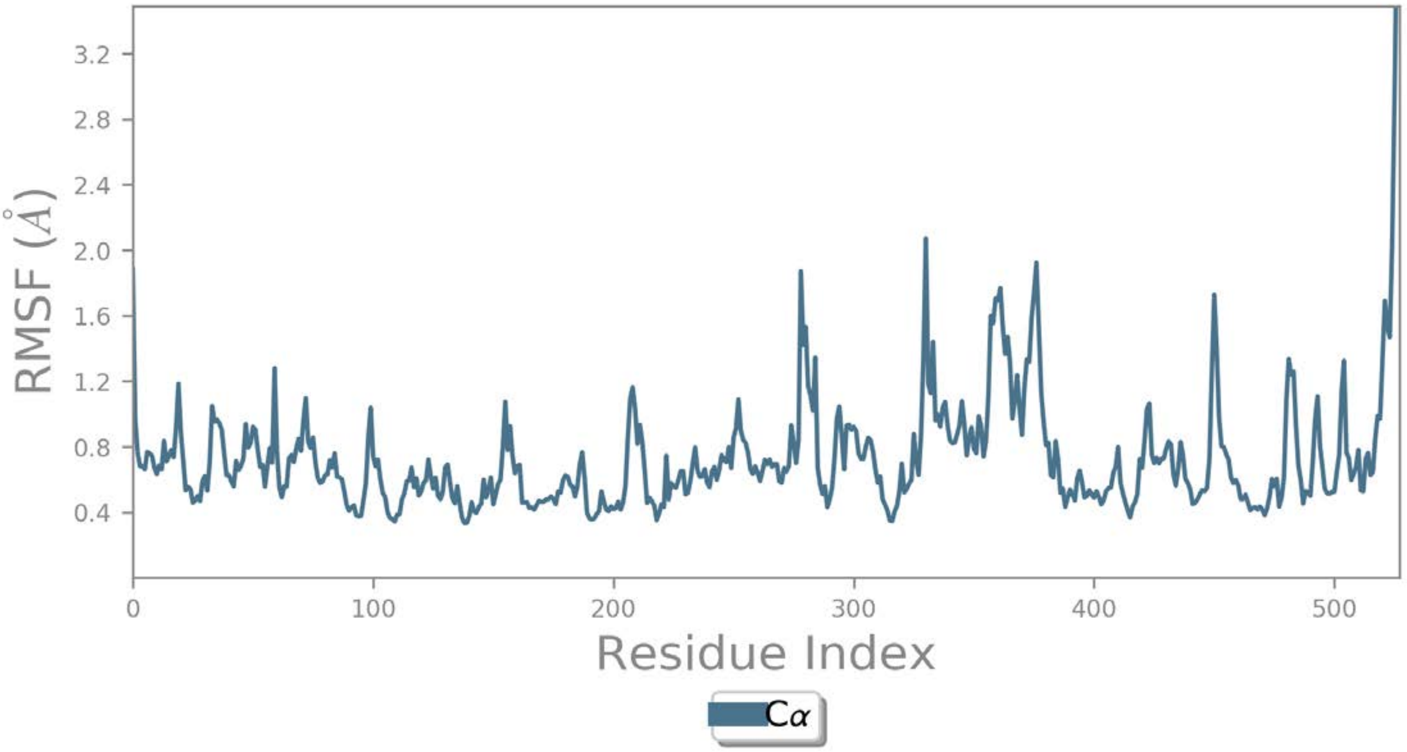

The Root Mean Square Fluctuation (RMSF) is useful for characterizing local changes along the protein chain. The RMSF for residue *i* is:

$$RMSF_i = \sqrt{\frac{1}{T}\sum_{t=1}^{T} < (r'_i(t)) - r_i(t_{ref}))^2 >}$$

where *T* is the trajectory time over which the RMSF is calculated, t_{ref} is the reference time, r_i is the position of residue *i*; *r'* is the position of atoms in residue *i* after superposition on the reference, and the angle brackets indicate that the average of the square distance is taken over the selection of atoms in the residue.

On this plot, peaks indicate areas of the protein that fluctuate the most during the simulation. Typically you will observe that the tails (*N*- and *C*-terminal) fluctuate more than any other part of the protein. Secondary structure elements like alpha helices and beta strands are usually more rigid than the unstructured part of the protein, and thus fluctuate less than the loop regions.

Protein Secondary Structure

% Helix	% Strand	% Total SSE
28.54	13.60	42.13

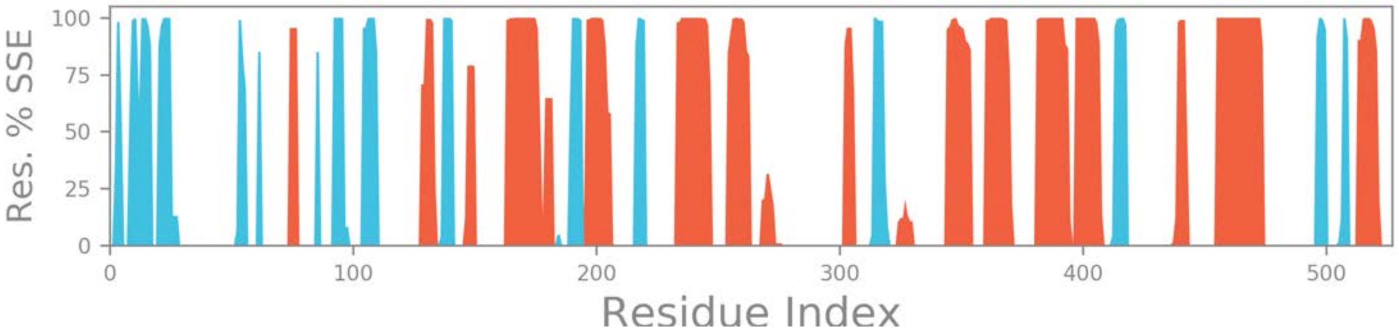

Protein secondary structure elements (SSE) like alpha-helices and beta-strands are monitored throughout the simulation. The plot above reports SSE distribution by residue index throughout the protein structure. The plot below summarizes the SSE composition for each trajectory frame over the course of the simulation, and the plot at the bottom monitors each residue and its SSE assignment over time.

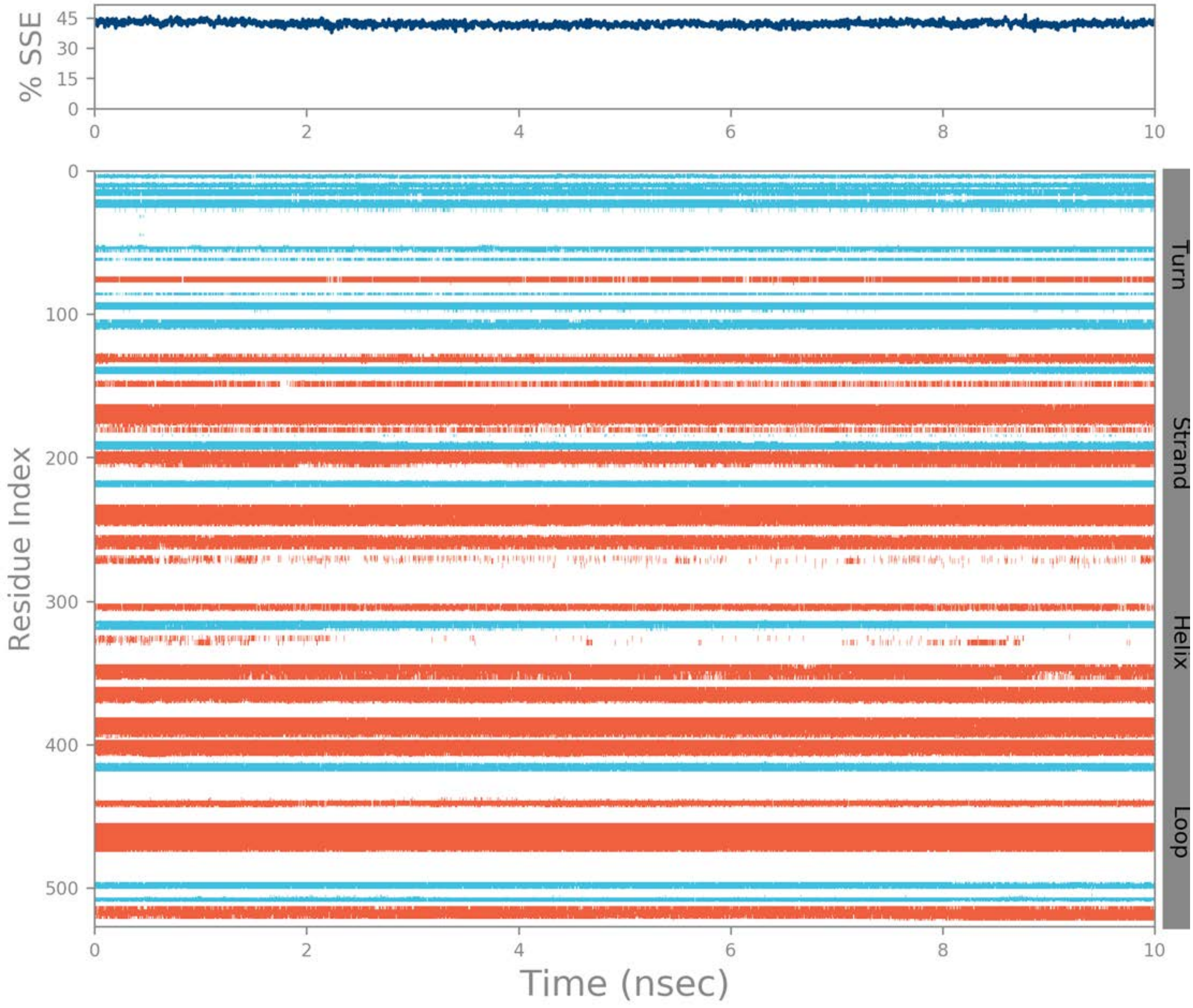

Stepharine Ligand RMSF

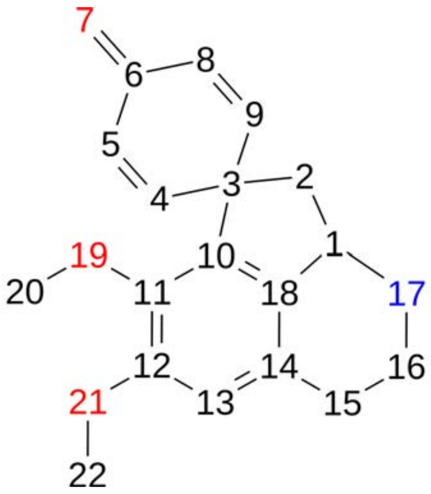

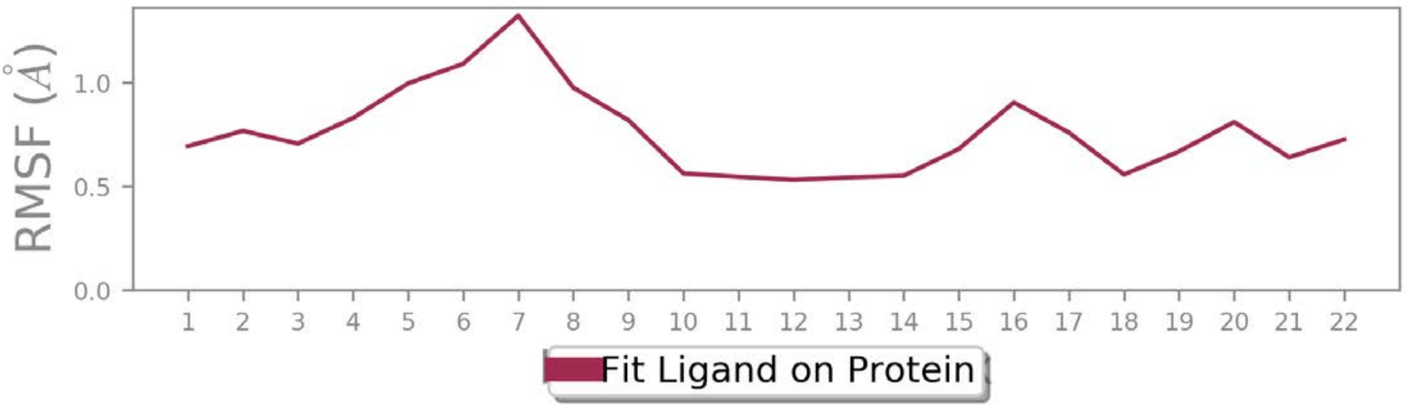

The Ligand Root Mean Square Fluctuation (L-RMSF) is useful for characterizing changes in the ligand atom positions. The RMSF for atom *i* is:

$$RMSF_i = \sqrt{\frac{1}{T}\sum_{t=1}^{T}(r_i'(t)) - r_i(t_{ref}))^2}$$

where T is the trajectory time over which the RMSF is calculated, t_{ref} is the reference time (usually for the first frame, and is regarded as the *zero* of time); r is the position of atom i in the reference at time t_{ref}, and r' is the position of atom i at time t after superposition on the reference frame.

Ligand RMSF shows the ligand's fluctuations broken down by atom, corresponding to the 2D structure in the top panel. The ligand RMSF may give you insights on how ligand fragments interact with the protein and their entropic role in the binding event. In the bottom panel, the 'Fit Ligand on Protein' line shows the ligand fluctuations, with respect to the protein. The protein-ligand complex is first aligned on the protein backbone and then the ligand RMSF is measured on the ligand heavy atoms.

Protein-Ligand Contacts

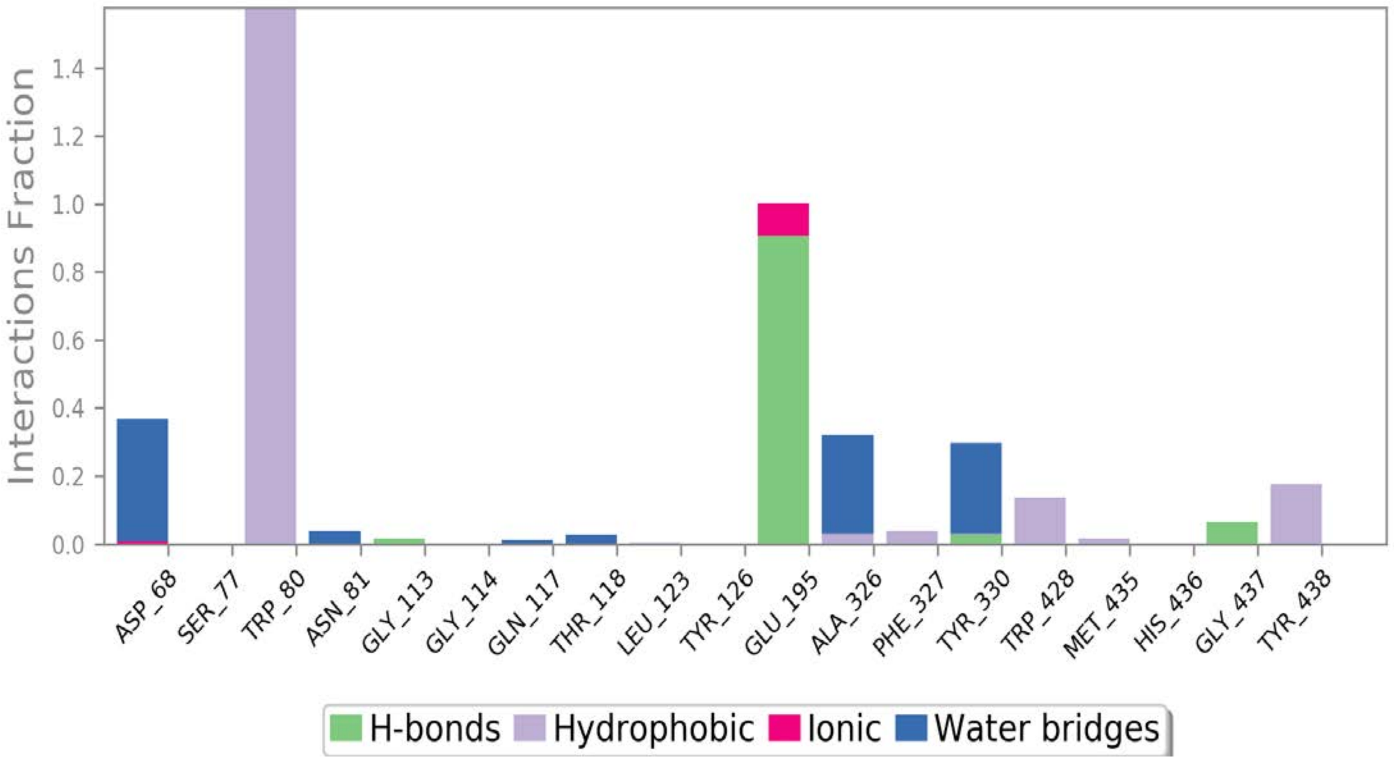

Protein interactions with the ligand can be monitored throughout the simulation. These interactions can be categorized by type and summarized, as shown in the plot above. Protein-ligand interactions (or 'contacts') are categorized into four types: Hydrogen Bonds, Hydrophobic, Ionic and Water Bridges. Each interaction type contains more specific subtypes, which can be explored through the 'Simulation Interactions Diagram' panel. The stacked bar charts are normalized over the course of the trajectory: for example, a value of 0.7 suggests that 70% of the simulation time the specific interaction is maintained. Values over 1.0 are possible as some protein residue may make multiple contacts of same subtype with the ligand.

Hydrogen Bonds: (H-bonds) play a significant role in ligand binding. Consideration of hydrogen-bonding properties in drug design is important because of their strong influence on drug specificity, metabolization and adsorption. Hydrogen bonds between a protein and a ligand can be further broken down into four subtypes: backbone acceptor; backbone donor; side-chain acceptor; side-chain donor.
The current geometric criteria for protein-ligand H-bond is: distance of 2.5 Å between the donor and acceptor atoms (D—H···A); a donor angle of ≥120° between the donor-hydrogen-acceptor atoms (D—H···A); and an acceptor angle of ≥90° between the hydrogen-acceptor-bonded_atom atoms (H···A—X).

Hydrophobic contacts: fall into three subtypes: π-Cation; π-π; and Other, non-specific interactions. Generally these type of interactions involve a hydrophobic amino acid and an aromatic or aliphatic group on the ligand, but we have extended this category to also include π-Cation interactions.
The current geometric criteria for hydrophobic interactions is as follows: π-Cation — Aromatic and charged groups within 4.5 Å; π-π — Two aromatic groups stacked face-to-face or face-to-edge; Other — A non-specific hydrophobic sidechain within 3.6 Å of a ligand's aromatic or aliphatic carbons.

Ionic interactions: or polar interactions, are between two oppositely charged atoms that are within 3.7 Å of each other and do not involve a hydrogen bond. We also monitor Protein-Metal-Ligand interactions, which are defined by a metal ion coordinated within 3.4 Å of protein's and ligand's heavy atoms (except carbon). All ionic interactions are broken down into two subtypes: those mediated by a protein backbone or side chains.

Water Bridges: are hydrogen-bonded protein-ligand interactions mediated by a water molecule. The hydrogen-bond geometry is slightly relaxed from the standard H-bond definition.
The current geometric criteria for a protein-water or water-ligand H-bond are: a distance of 2.8 Å between the donor and acceptor atoms (D—H···A); a donor angle of ≥110° between the donor-hydrogen-acceptor atoms (D—H···A); and an acceptor angle of ≥90° between the hydrogen-acceptor-bonded_atom atoms (H···A—X).

Protein-Ligand Contacts (cont.)

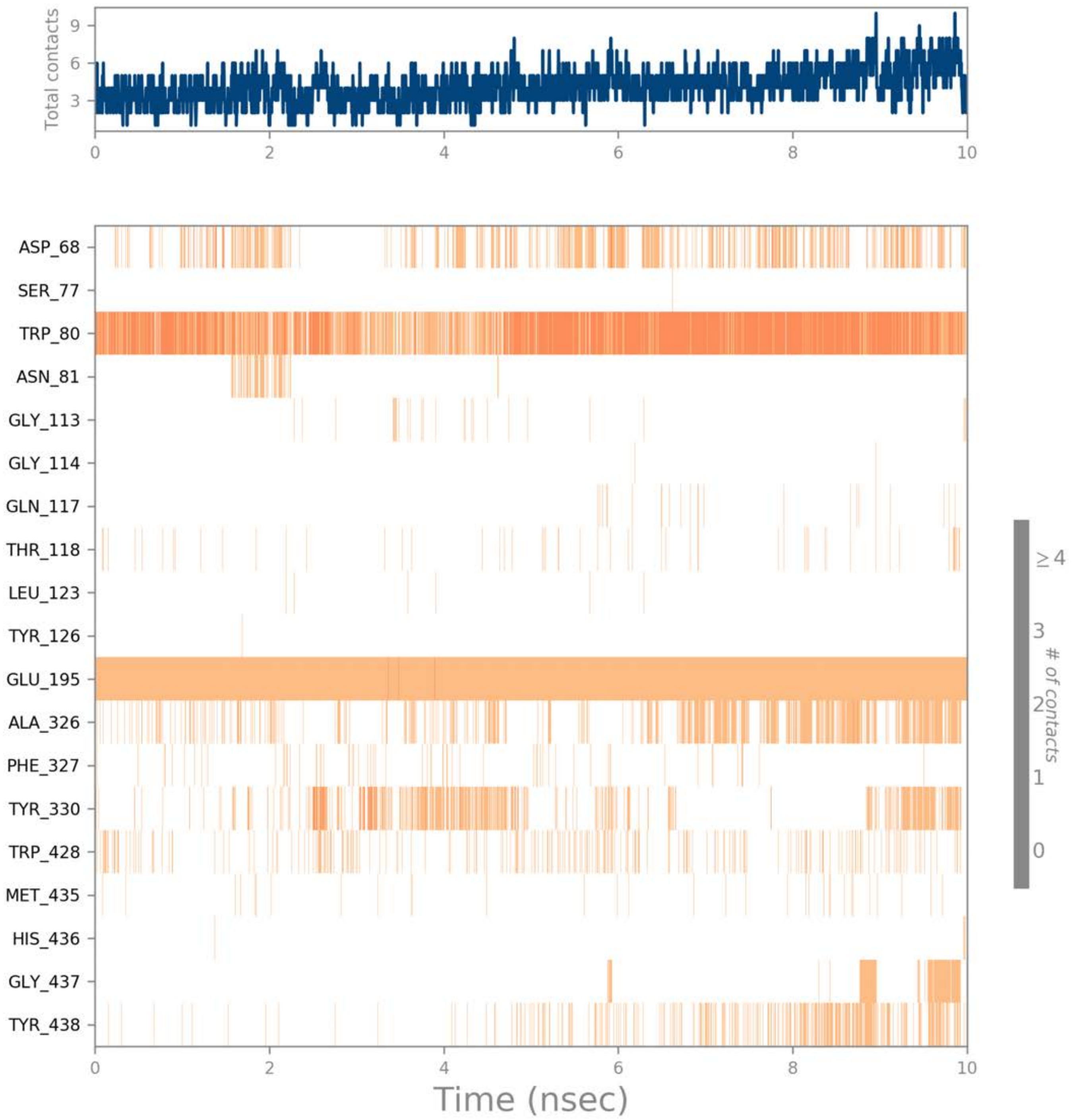

A timeline representation of the interactions and contacts (**H-bonds**, **Hydrophobic**, **Ionic**, **Water bridges**) summarized in the previous page. The top panel shows the total number of specific contacts the protein makes with the ligand over the course of the trajectory. The bottom panel shows which residues interact with the ligand in each trajectory frame. Some residues make more than one specific contact with the ligand, which is represented by a darker shade of orange, according to the scale to the right of the plot.

Ligand-Protein Contacts

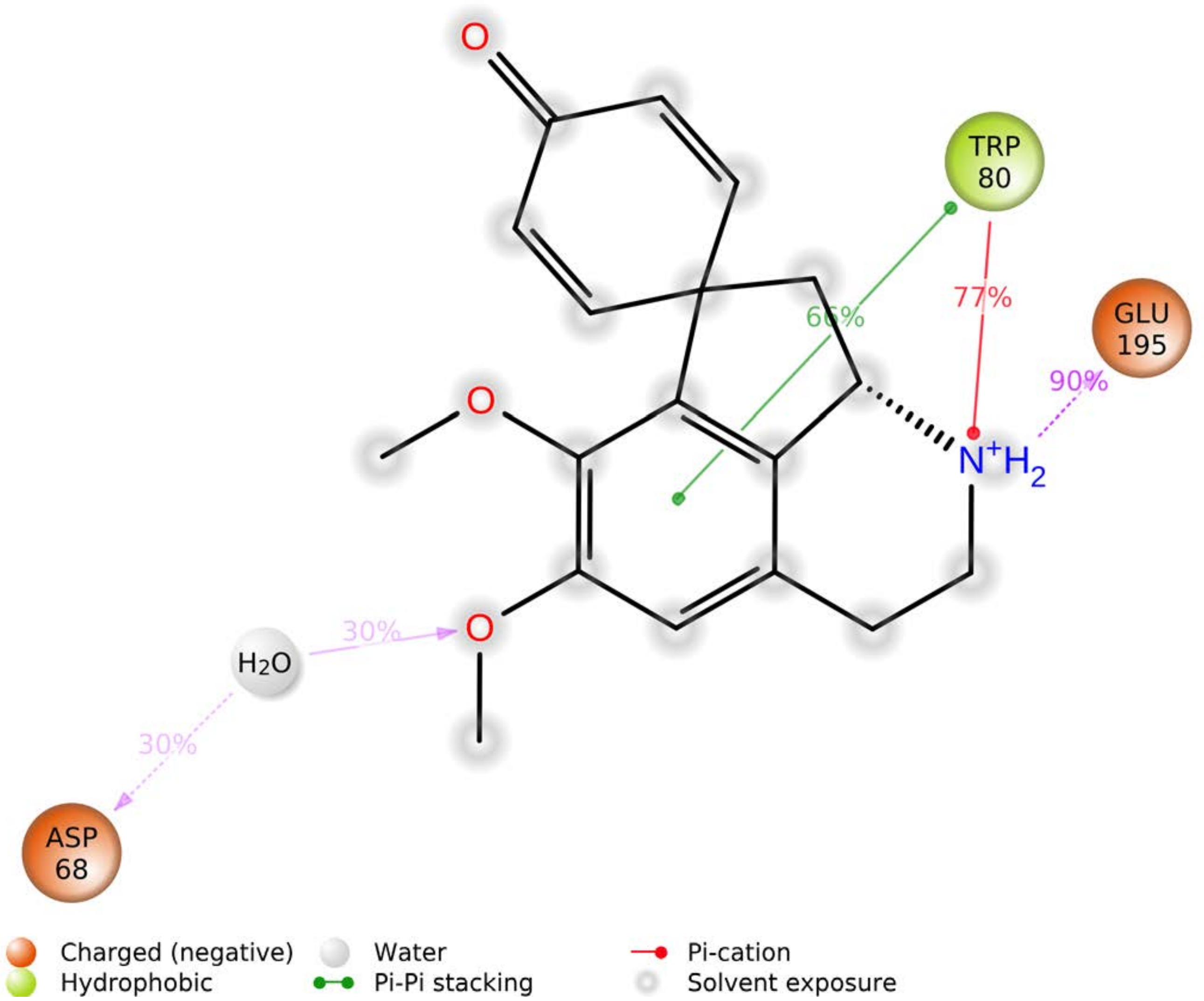

A schematic of detailed ligand atom interactions with the protein residues. Interactions that occur more than **30.0%** of the simulation time in the selected trajectory (0.00 through 10.00 nsec), are shown.
Note: it is possible to have interactions with >100% as some residues may have multiple interactions of a single type with the same ligand atom. For example, the ARG side chain has four H-bond donors that can all hydrogen-bond to a single H-bond acceptor.

Ligand Torsion Profile

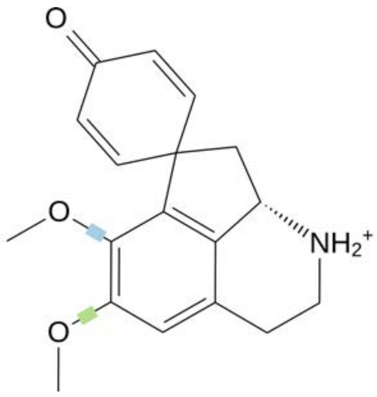

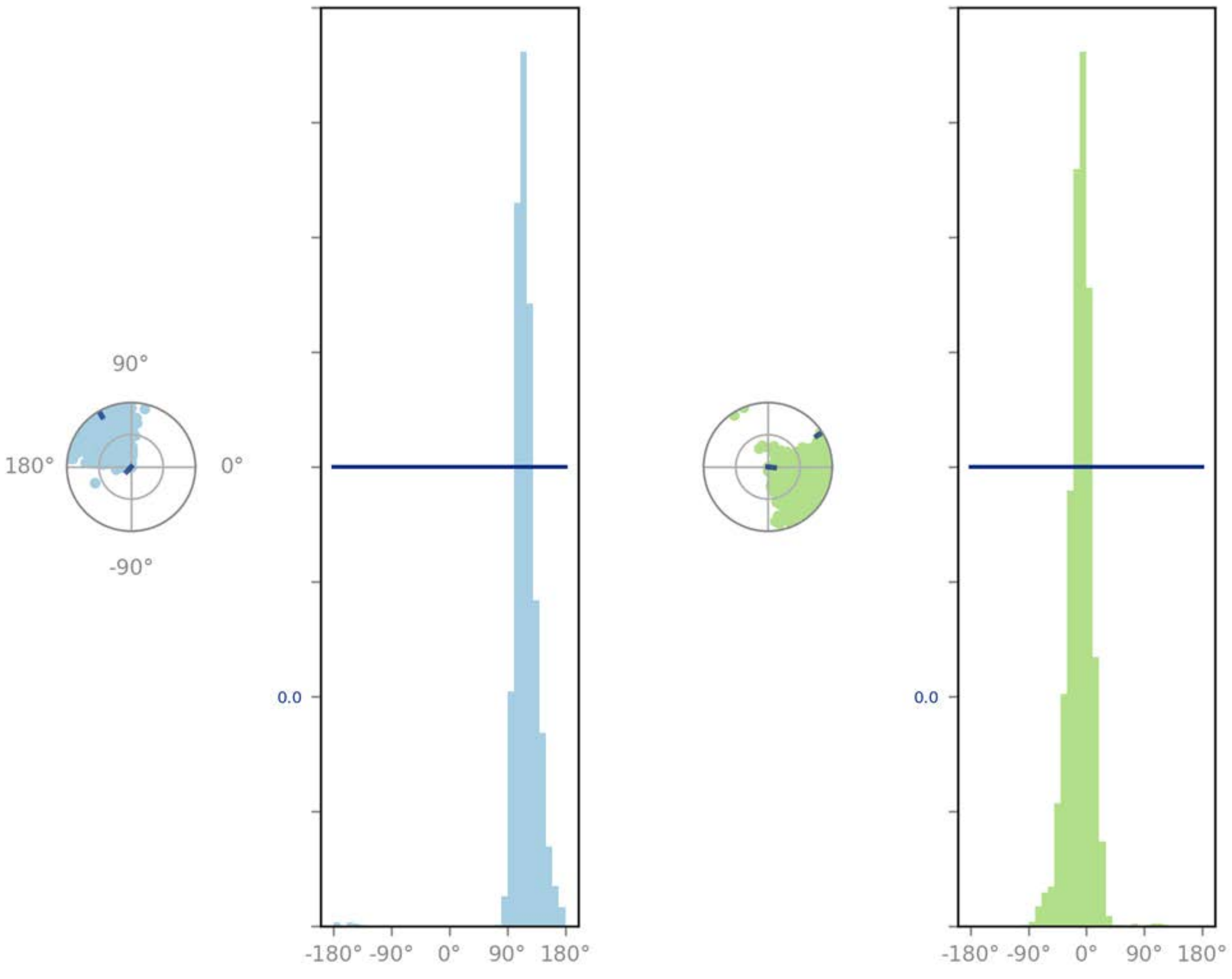

The ligand torsions plot summarizes the conformational evolution of every rotatable bond (RB) in the ligand throughout the simulation trajectory (0.00 through 10.00 nsec). The top panel shows the 2d schematic of a ligand with color-coded rotatable bonds. Each rotatable bond torsion is accompanied by a dial plot and bar plots of the same color.

Dial (or radial) plots describe the conformation of the torsion throughout the course of the simulation. The beginning of the simulation is in the center of the radial plot and the time evolution is plotted radially outwards.

The bar plots summarize the data on the dial plots, by showing the probability density of the torsion. If torsional potential information is available, the plot also shows the potential of the rotatable bond (by summing the potential of the related torsions). The values of the potential are on the left *Y*-axis of the chart, and are expressed in *kcal/mol*. Looking at the histogram and torsion potential relationships may give insights into the

conformational strain the ligand undergoes to maintain a protein-bound conformation.

Ligand Properties

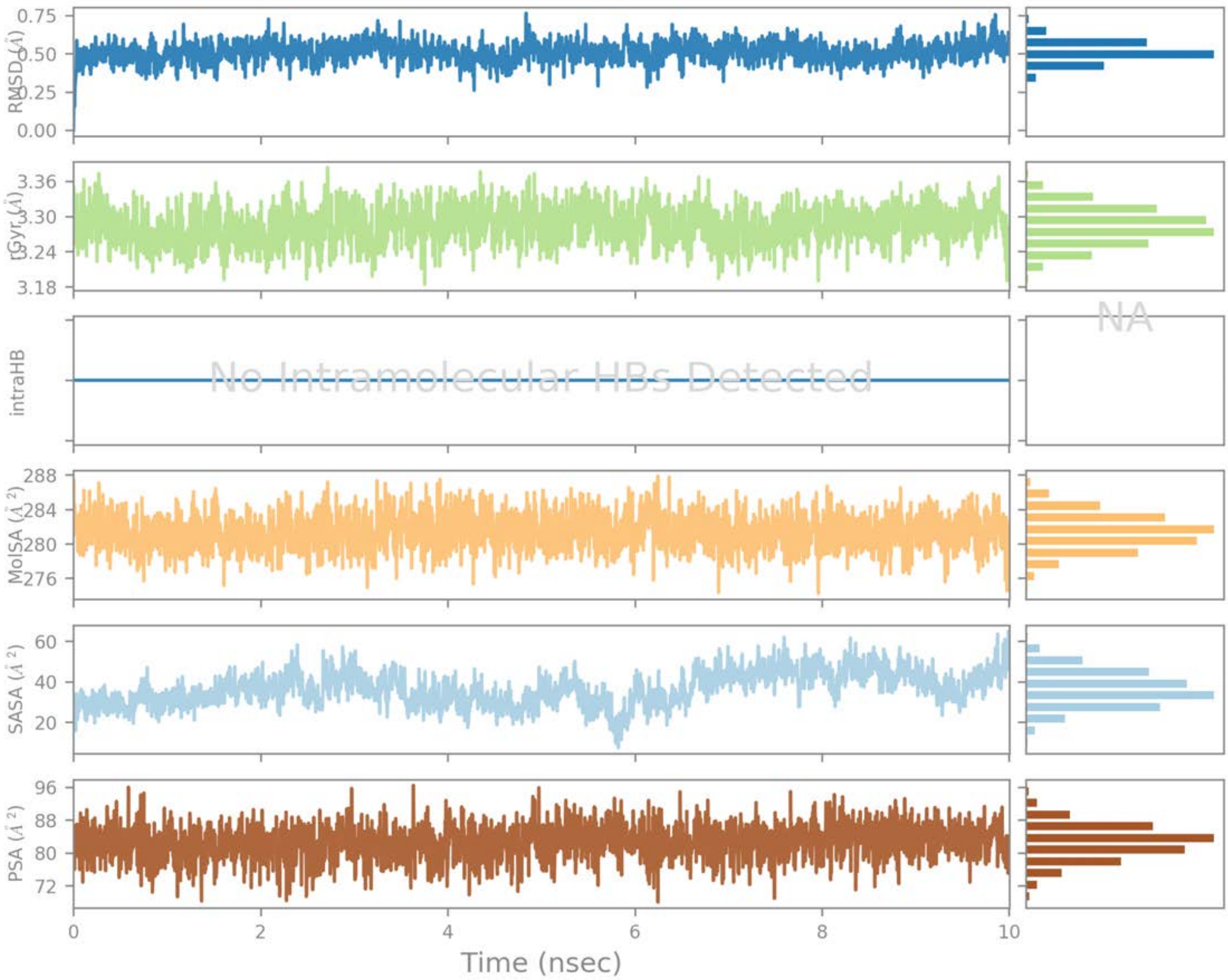

Ligand RMSD: Root mean square deviation of a ligand with respect to the reference conformation (typically the first frame is used as the reference and it is regarded as time *t*=0).

Radius of Gyration (rGyr): Measures the 'extendedness' of a ligand, and is equivalent to its principal moment of inertia.

Intramolecular Hydrogen Bonds (intraHB): Number of internal hydrogen bonds (HB) within a ligand molecule.

Molecular Surface Area (MolSA): Molecular surface calculation with 1.4 Å probe radius. This value is equivalent to a van der Waals surface area.

Solvent Accessible Surface Area (SASA): Surface area of a molecule accessible by a water molecule.

Polar Surface Area (PSA): Solvent accessible surface area in a molecule contributed only by oxygen and nitrogen atoms.

Report S10
MD Simulation Report on BChE - Palmatine Interactions

Simulation Details

Jobname: md_job_6EP4_3-dock-2
Entry title: 6EP4_3-dock-2

CPU #	Job Type	Ensemble	Temp. [K]	Sim. Time [ns]	# Atoms	# Waters	Charge
1	mdsim	NPT	300.0	10.005	51012	14190	0

Protein Information

Tot. Residues	Prot. Chain(s)	Res. in Chain(s)	# Atoms	# Heavy Atoms	Charge
527	'NoChainId'	ict_values([527]	8313	4203	+2

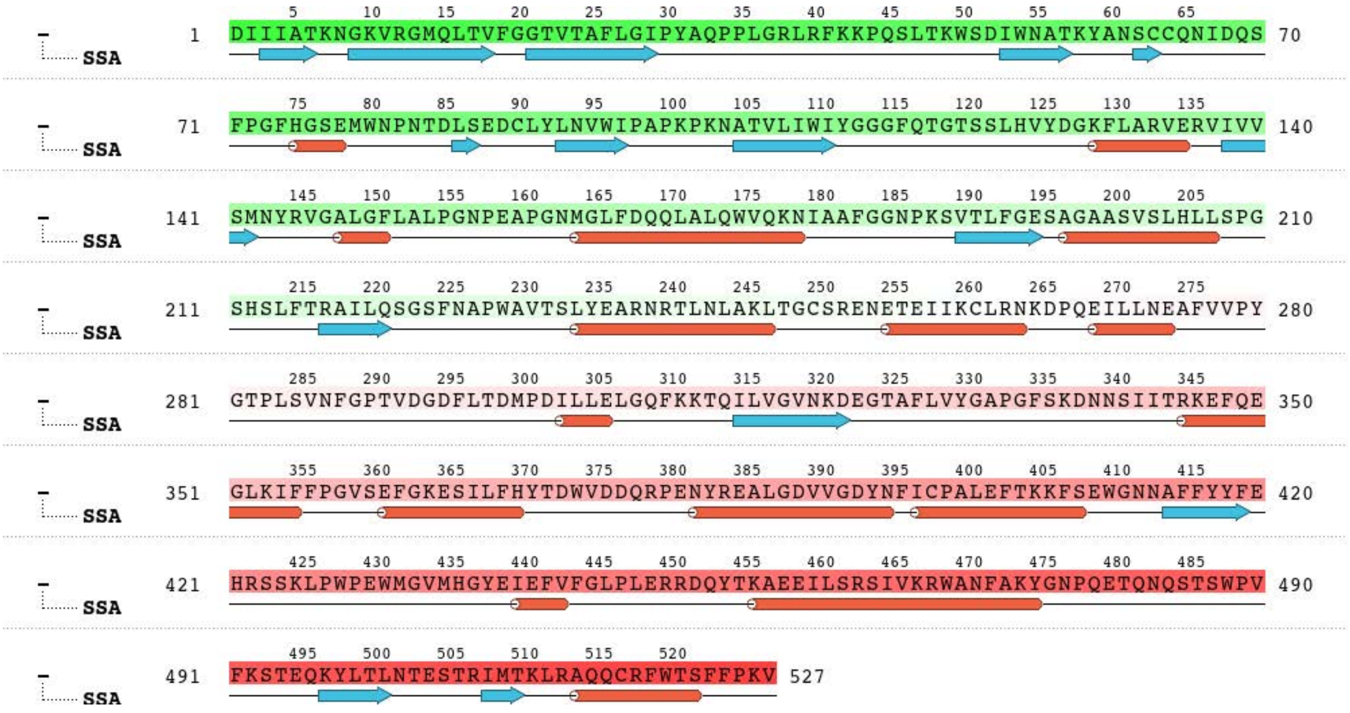

Ligand Information

SMILES	COc1c(OC)ccc(c12)cc3c4c(CC[n+]3c2)cc(OC)c(c4)OC
PDB Name	'UNK'
Num. of Atoms	48 (total) 26 (heavy)
Atomic Mass	352.414 au
Charge	+1
Mol. Formula	C21H22NO4
Num. of Fragments	1
Num. of Rot. Bonds	4

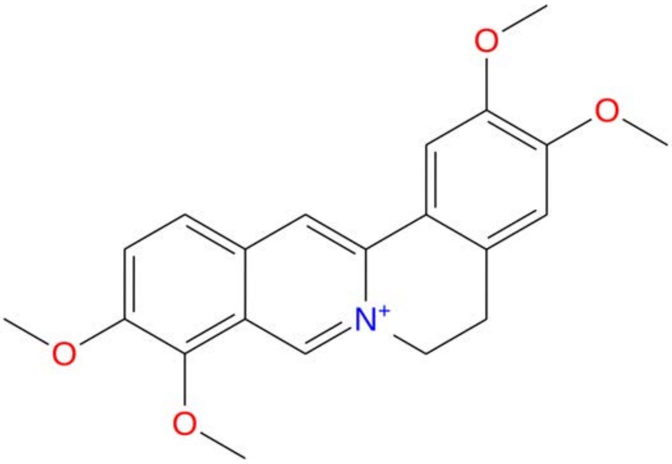

Counter Ion/Salt Information

Type	Num.	Concentration [mM]	Total Charge
Cl	42	53.815	-42

Na	39	49.971	+39

Protein-Ligand RMSD

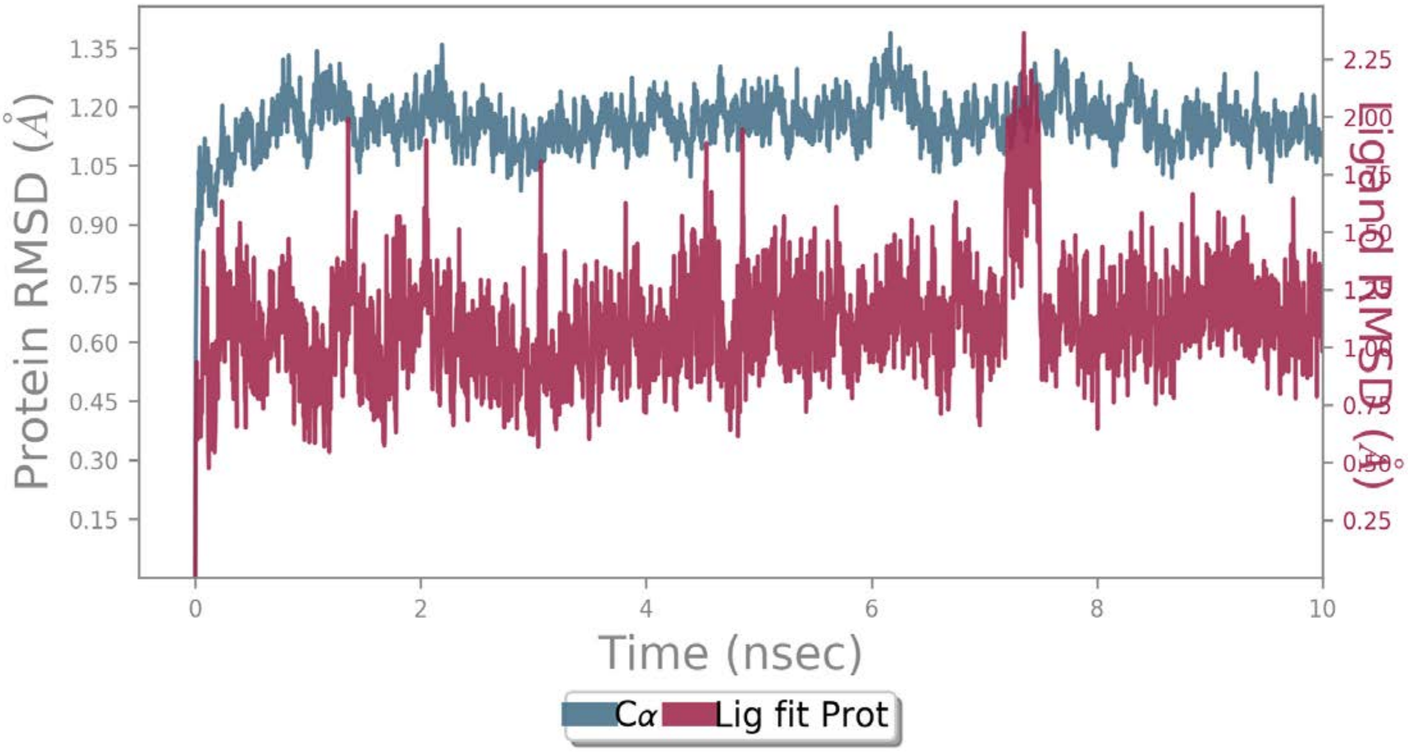

The Root Mean Square Deviation (RMSD) is used to measure the average change in displacement of a selection of atoms for a particular frame with respect to a reference frame. It is calculated for all frames in the trajectory. The RMSD for frame *x* is:

$$RMSD_x = \sqrt{\frac{1}{N}\sum_{i=1}^{N}(r'_i(t_x)) - r_i(t_{ref}))^2}$$

where *N* is the number of atoms in the atom selection; t_{ref} is the reference time, (typically the first frame is used as the reference and it is regarded as time *t*=0); and *r'* is the position of the selected atoms in frame *x* after superimposing on the reference frame, where frame *x* is recorded at time t_x. The procedure is repeated for every frame in the simulation trajectory.

Protein RMSD: The above plot shows the RMSD evolution of a protein (left *Y*-axis). All protein frames are first aligned on the reference frame backbone, and then the RMSD is calculated based on the atom selection. Monitoring the RMSD of the protein can give insights into its structural conformation throughout the simulation. RMSD analysis can indicate if the simulation has equilibrated — its fluctuations towards the end of the simulation are around some thermal average structure. Changes of the order of 1-3 Å are perfectly acceptable for small, globular proteins. Changes much larger than that, however, indicate that the protein is undergoing a large conformational change during the simulation. It is also important that your simulation converges — the RMSD values stabilize around a fixed value. If the RMSD of the protein is still increasing or decreasing on average at the end of the simulation, then your system has not equilibrated, and your simulation may not be long enough for rigorous analysis.

Ligand RMSD: Ligand RMSD (right *Y*-axis) indicates how stable the ligand is with respect to the protein and its binding pocket. In the above plot, 'Lig fit Prot' shows the RMSD of a ligand when the protein-ligand complex is first aligned on the protein backbone of the reference and then the RMSD of the ligand heavy atoms is measured. If the values observed are significantly larger than the RMSD of the protein, then it is likely that the ligand has diffused away from its initial binding site.

Protein RMSF

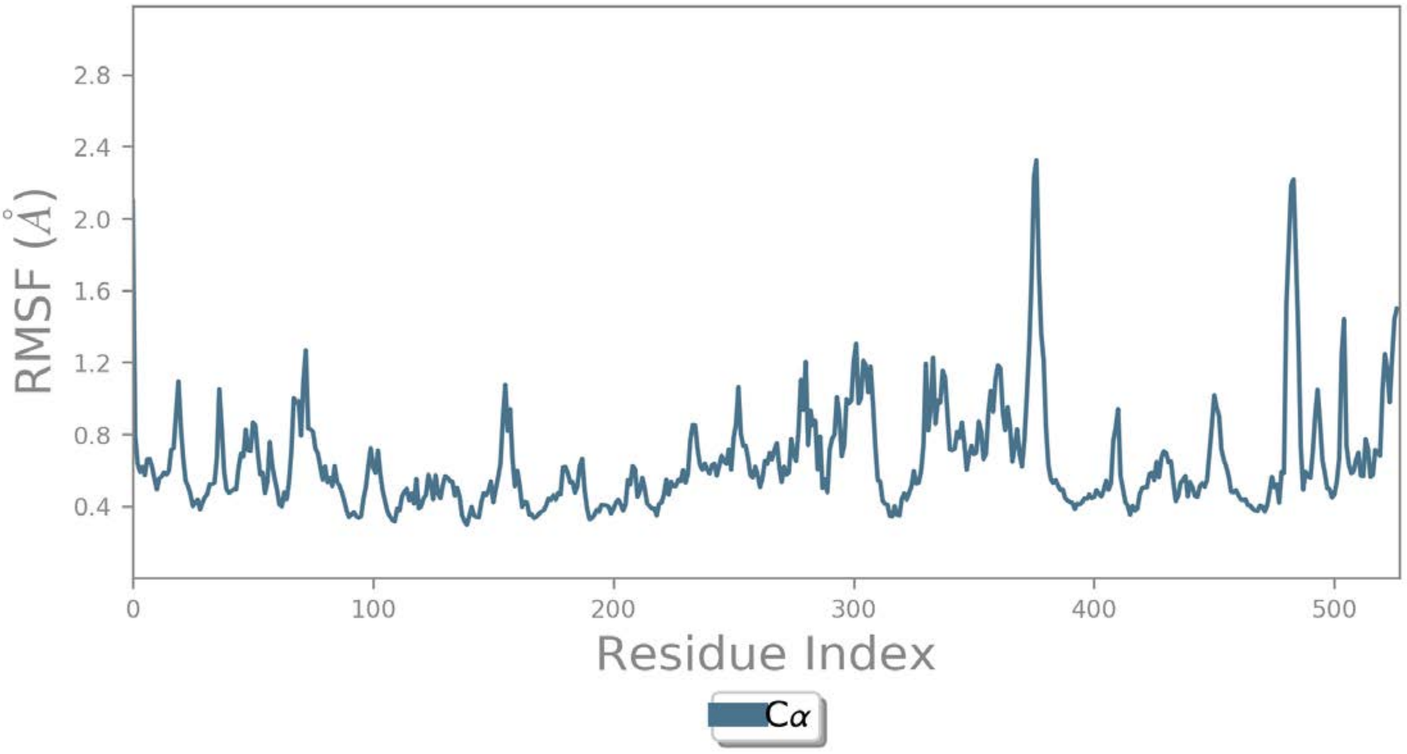

The Root Mean Square Fluctuation (RMSF) is useful for characterizing local changes along the protein chain. The RMSF for residue *i* is:

$$RMSF_i = \sqrt{\frac{1}{T}\sum_{t=1}^{T} < (r'_i(t)) - r_i(t_{ref}))^2 >}$$

where T is the trajectory time over which the RMSF is calculated, t_{ref} is the reference time, r_i is the position of residue *i*; *r'* is the position of atoms in residue *i* after superposition on the reference, and the angle brackets indicate that the average of the square distance is taken over the selection of atoms in the residue.

On this plot, peaks indicate areas of the protein that fluctuate the most during the simulation. Typically you will observe that the tails (*N*- and *C*-terminal) fluctuate more than any other part of the protein. Secondary structure elements like alpha helices and beta strands are usually more rigid than the unstructured part of the protein, and thus fluctuate less than the loop regions.

Protein Secondary Structure

% Helix	% Strand	% Total SSE
28.19	13.62	41.82

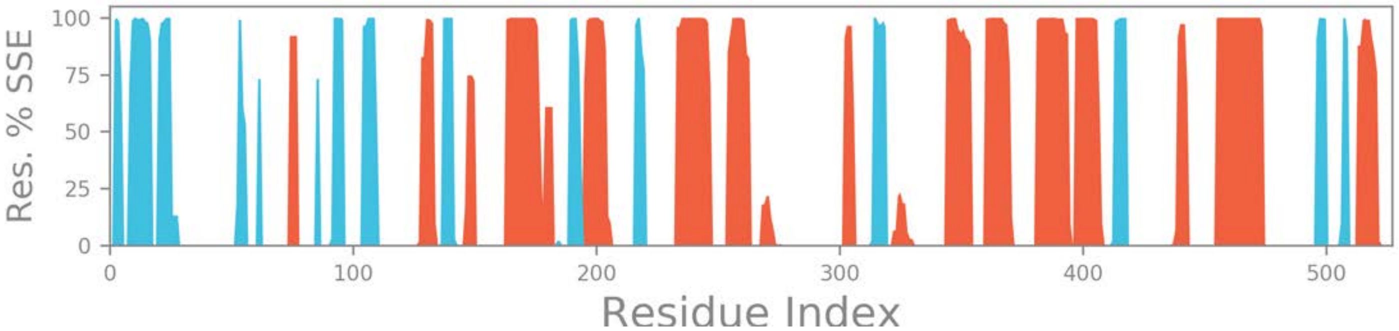

Protein secondary structure elements (SSE) like alpha-helices and beta-strands are monitored throughout the simulation. The plot above reports SSE distribution by residue index throughout the protein structure. The plot below summarizes the SSE composition for each trajectory frame over the course of the simulation, and the plot at the bottom monitors each residue and its SSE assignment over time.

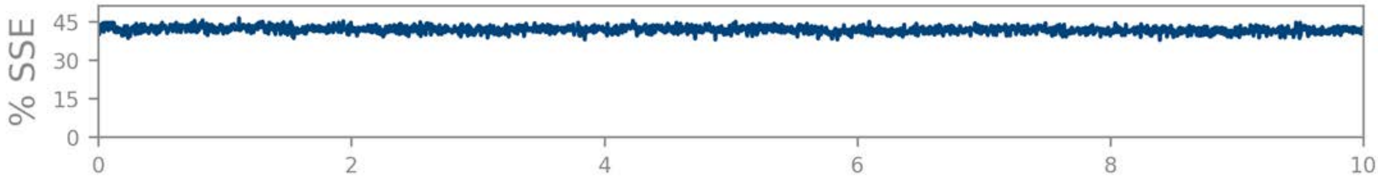

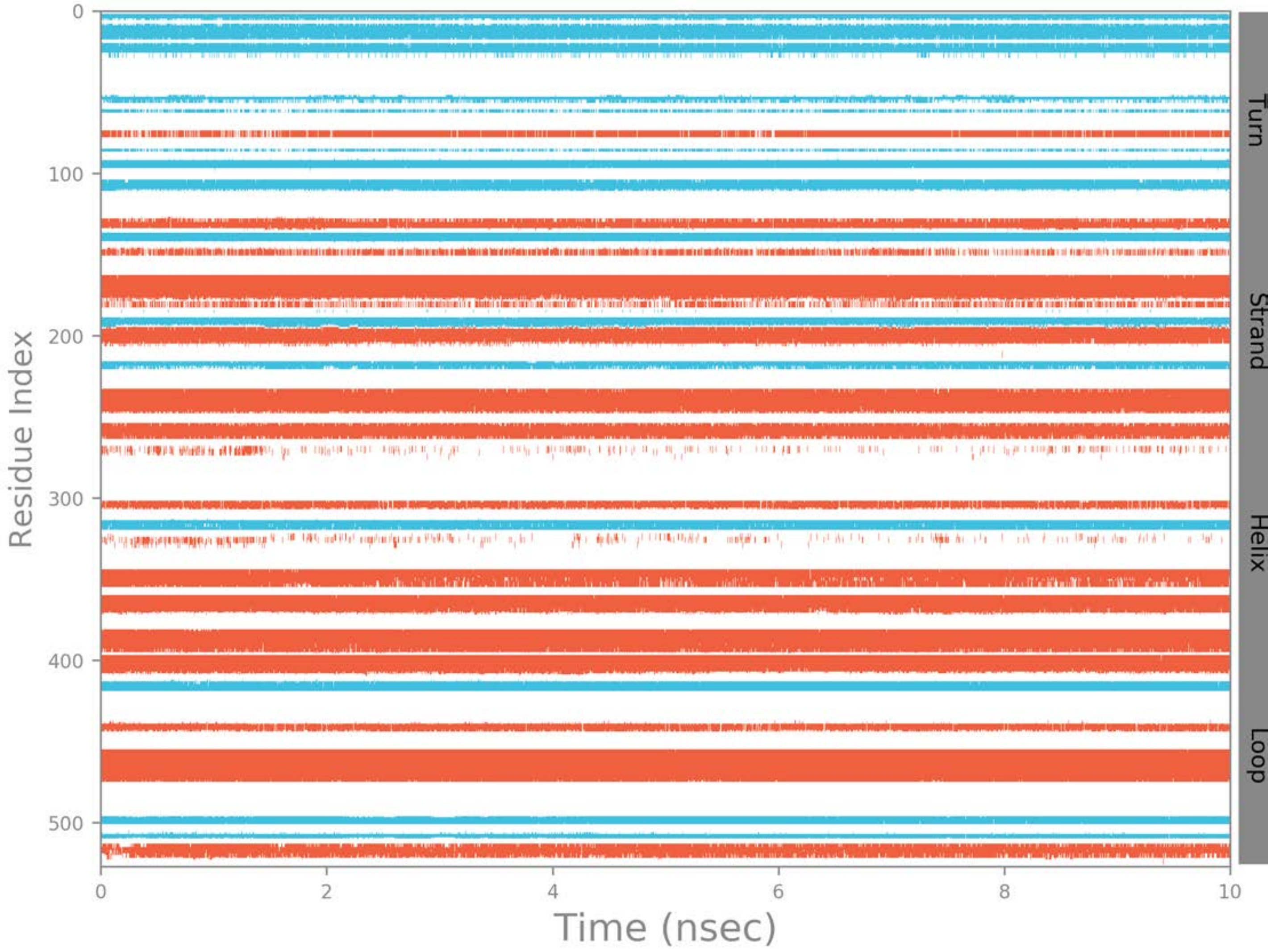

Palmatine Ligand RMSF

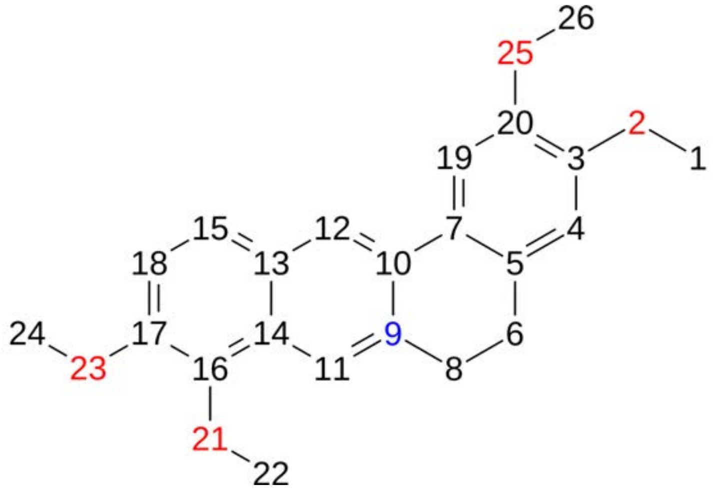

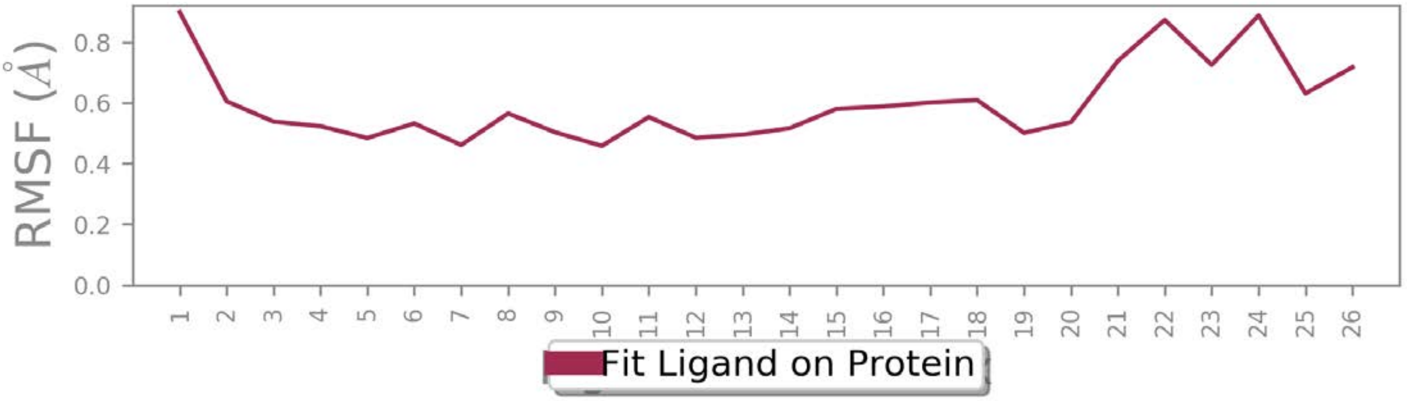

The Ligand Root Mean Square Fluctuation (L-RMSF) is useful for characterizing changes in the ligand atom positions. The RMSF for atom *i* is:

$$RMSF_i = \sqrt{\frac{1}{T}\sum_{t=1}^{T}(r_i'(t)) - r_i(t_{ref}))^2}$$

where T is the trajectory time over which the RMSF is calculated, t_{ref} is the reference time (usually for the first frame, and is regarded as the *zero* of time); r is the position of atom i in the reference at time t_{ref}, and r' is the position of atom i at time t after superposition on the reference frame.

Ligand RMSF shows the ligand's fluctuations broken down by atom, corresponding to the 2D structure in the top panel. The ligand RMSF may give you insights on how ligand fragments interact with the protein and their entropic role in the binding event. In the bottom panel, the 'Fit Ligand on Protein' line shows the ligand fluctuations, with respect to the protein. The protein-ligand complex is first aligned on the protein backbone and then the ligand RMSF is measured on the ligand heavy atoms.

Protein-Ligand Contacts

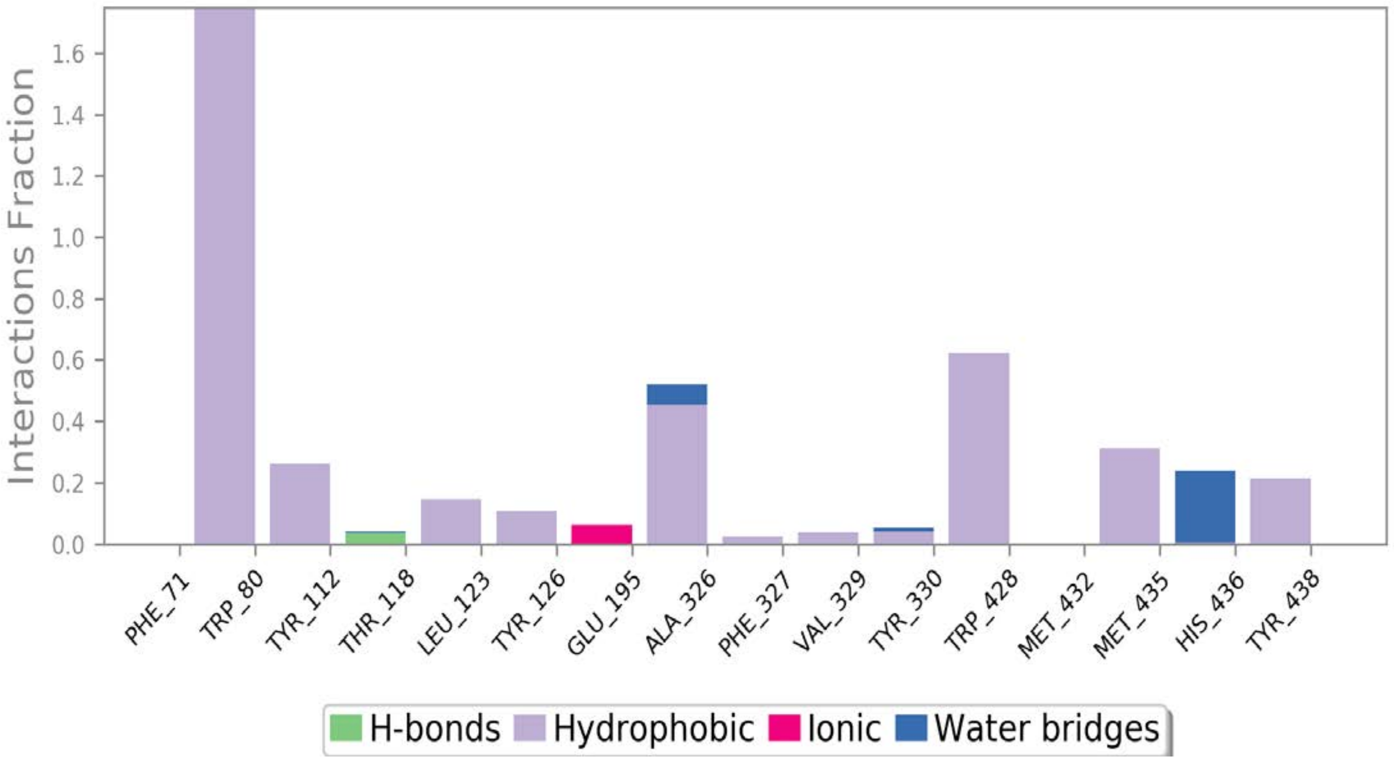

Protein interactions with the ligand can be monitored throughout the simulation. These interactions can be categorized by type and summarized, as shown in the plot above. Protein-ligand interactions (or 'contacts') are categorized into four types: Hydrogen Bonds, Hydrophobic, Ionic and Water Bridges. Each interaction type contains more specific subtypes, which can be explored through the 'Simulation Interactions Diagram' panel. The stacked bar charts are normalized over the course of the trajectory: for example, a value of 0.7 suggests that 70% of the simulation time the specific interaction is maintained. Values over 1.0 are possible as some protein residue may make multiple contacts of same subtype with the ligand.

Hydrogen Bonds: (H-bonds) play a significant role in ligand binding. Consideration of hydrogen-bonding properties in drug design is important because of their strong influence on drug specificity, metabolization and adsorption. Hydrogen bonds between a protein and a ligand can be further broken down into four subtypes: backbone acceptor; backbone donor; side-chain acceptor; side-chain donor.
The current geometric criteria for protein-ligand H-bond is: distance of 2.5 Å between the donor and acceptor atoms (D—H···A); a donor angle of ≥120° between the donor-hydrogen-acceptor atoms (D—H···A); and an acceptor angle of ≥90° between the hydrogen-acceptor-bonded_atom atoms (H···A—X).

Hydrophobic contacts: fall into three subtypes: π-Cation; π-π; and Other, non-specific interactions. Generally these type of interactions involve a hydrophobic amino acid and an aromatic or aliphatic group on the ligand, but we have extended this category to also include π-Cation interactions.
The current geometric criteria for hydrophobic interactions is as follows: π-Cation — Aromatic and charged groups within 4.5 Å; π-π — Two aromatic groups stacked face-to-face or face-to-edge; Other — A non-specific hydrophobic sidechain within 3.6 Å of a ligand's aromatic or aliphatic carbons.

Ionic interactions: or polar interactions, are between two oppositely charged atoms that are within 3.7 Å of each other and do not involve a hydrogen bond. We also monitor Protein-Metal-Ligand interactions, which are defined by a metal ion coordinated within 3.4 Å of protein's and ligand's heavy atoms (except carbon). All ionic interactions are broken down into two subtypes: those mediated by a protein backbone or side chains.

Water Bridges: are hydrogen-bonded protein-ligand interactions mediated by a water molecule. The hydrogen-bond geometry is slightly relaxed from the standard H-bond definition.
The current geometric criteria for a protein-water or water-ligand H-bond are: a distance of 2.8 Å between the donor and acceptor atoms (D—H···A); a donor angle of ≥110° between the donor-hydrogen-acceptor atoms (D—H···A); and an acceptor angle of ≥90° between the hydrogen-acceptor-bonded_atom atoms (H···A—X).

Protein-Ligand Contacts (cont.)

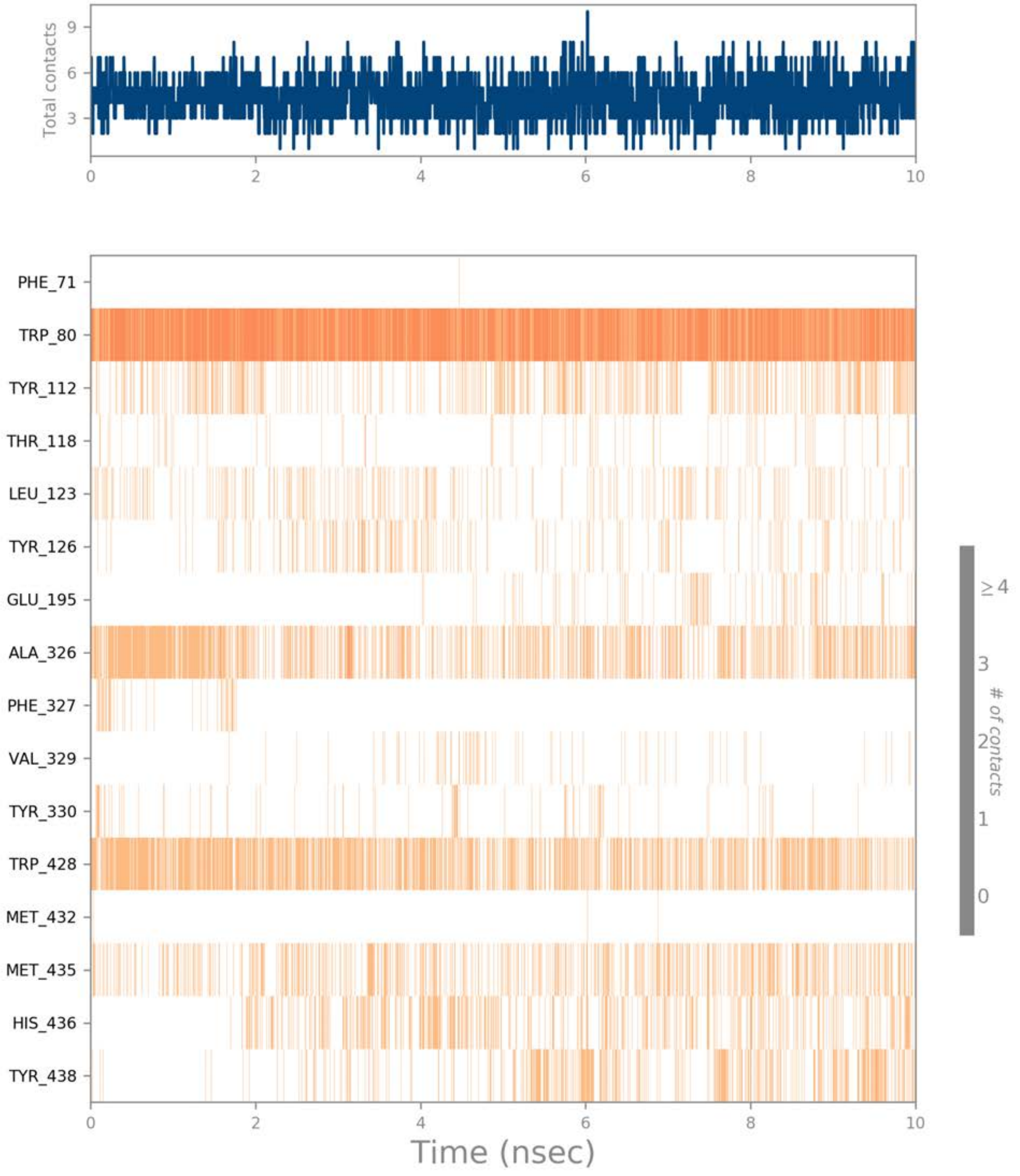

A timeline representation of the interactions and contacts (**H-bonds**, **Hydrophobic**, **Ionic**, **Water bridges**) summarized in the previous page. The top panel shows the total number of specific contacts the protein makes with the ligand over the course of the trajectory. The bottom panel shows which residues interact with the ligand in each trajectory frame. Some residues make more than one specific contact with the ligand, which is represented by a darker shade of orange, according to the scale to the right of the plot.

Ligand-Protein Contacts

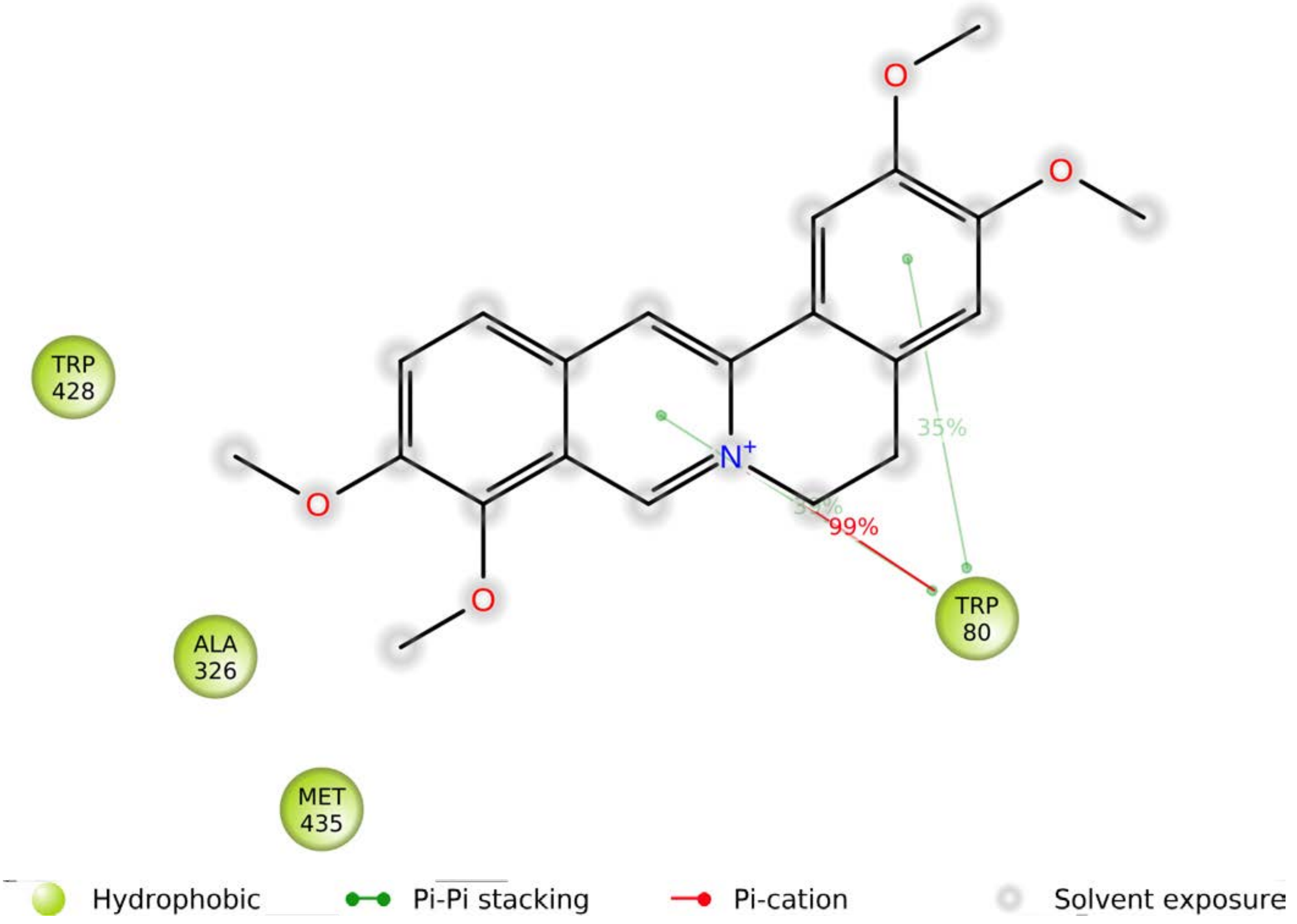

A schematic of detailed ligand atom interactions with the protein residues. Interactions that occur more than **30.0%** of the simulation time in the selected trajectory (0.00 through 10.00 nsec), are shown.
Note: it is possible to have interactions with >100% as some residues may have multiple interactions of a single type with the same ligand atom. For example, the ARG side chain has four H-bond donors that can all hydrogen-bond to a single H-bond acceptor.

Ligand Torsion Profile

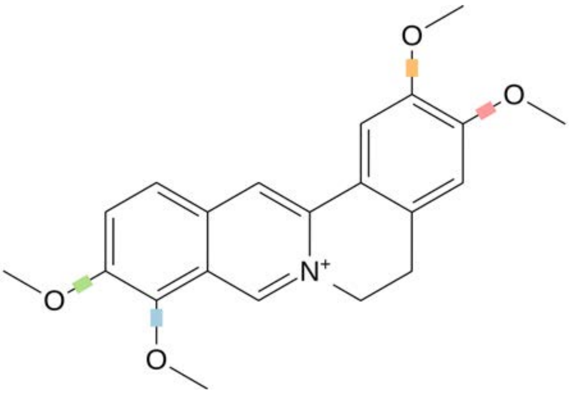

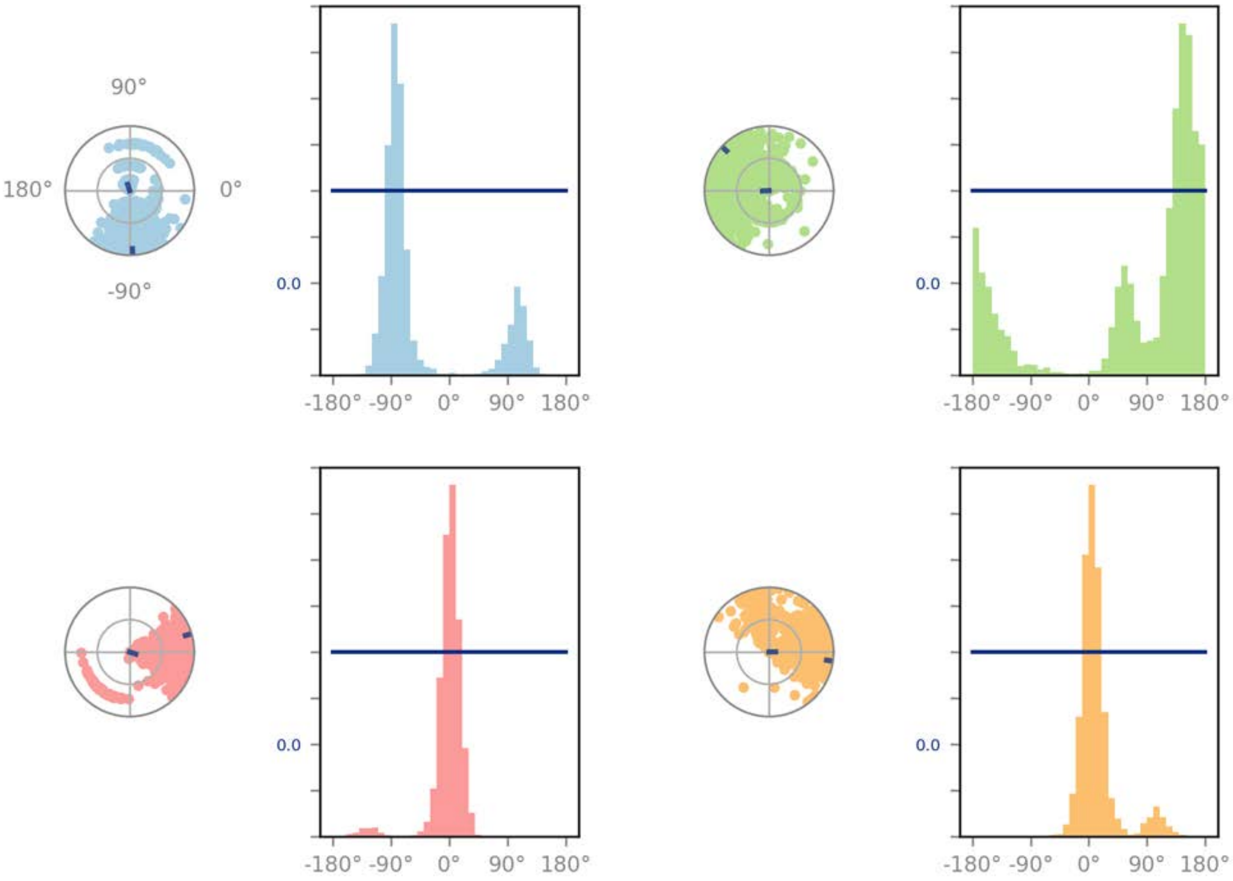

The ligand torsions plot summarizes the conformational evolution of every rotatable bond (RB) in the ligand throughout the simulation trajectory (0.00 through 10.00 nsec). The top panel shows the 2d schematic of a ligand with color-coded rotatable bonds. Each rotatable bond torsion is accompanied by a dial plot and bar plots of the same color.

Dial (or radial) plots describe the conformation of the torsion throughout the course of the simulation. The beginning of the simulation is in the center of the radial plot and the time evolution is plotted radially outwards.

The bar plots summarize the data on the dial plots, by showing the probability density of the torsion. If torsional potential information is available, the plot also shows the potential of the rotatable bond (by summing the potential of the related torsions). The values of the potential are on the left *Y*-axis of the chart, and are expressed in *kcal/mol*. Looking at the histogram and torsion potential relationships may give insights into the conformational strain the ligand undergoes to maintain a protein-bound conformation.

Ligand Properties

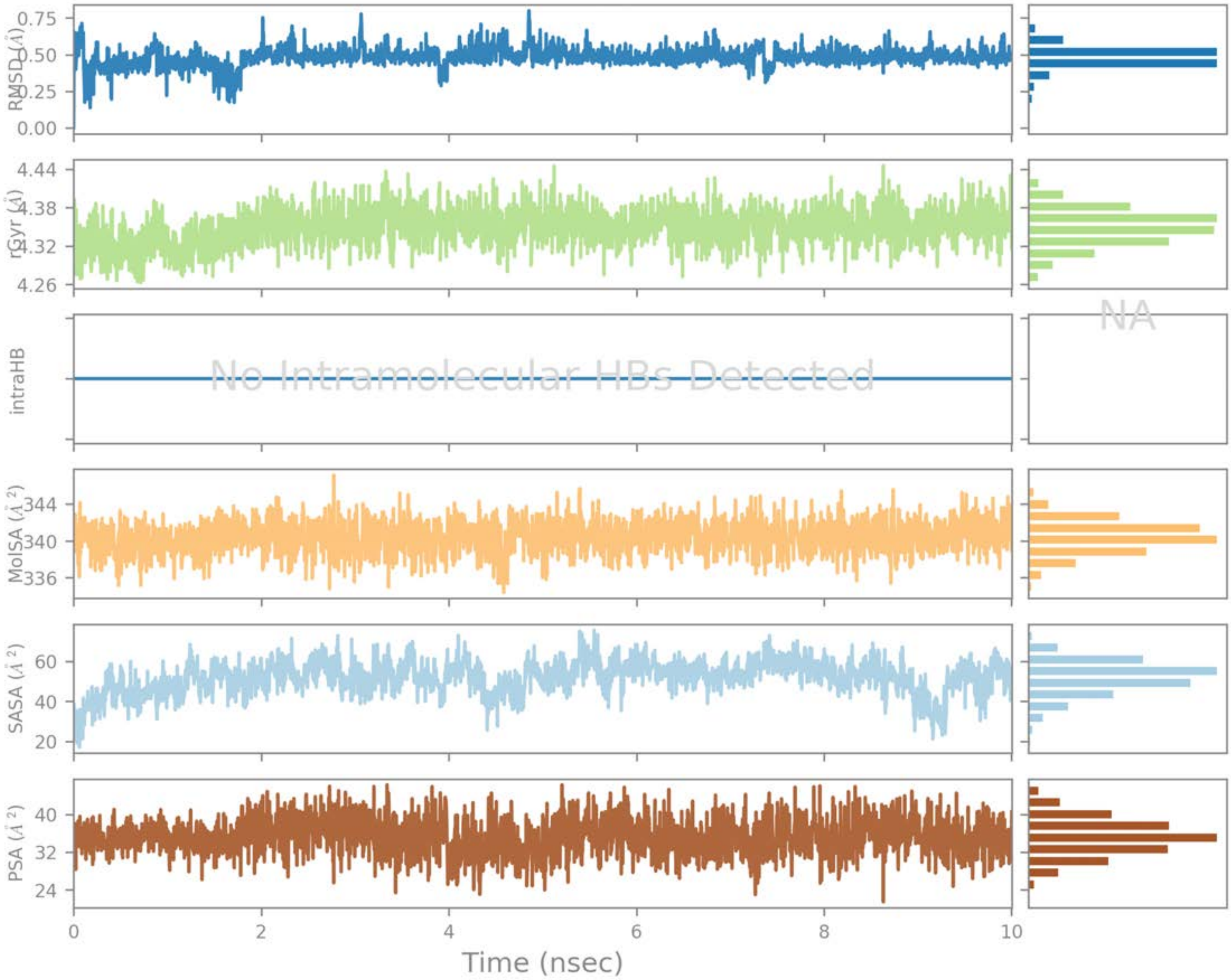

Ligand RMSD: Root mean square deviation of a ligand with respect to the reference conformation (typically the first frame is used as the reference and it is regarded as time *t*=0).

Radius of Gyration (rGyr): Measures the 'extendedness' of a ligand, and is equivalent to its principal moment of inertia.

Intramolecular Hydrogen Bonds (intraHB): Number of internal hydrogen bonds (HB) within a ligand molecule.

Molecular Surface Area (MolSA): Molecular surface calculation with 1.4 Å probe radius. This value is equivalent to a van der Waals surface area.

Solvent Accessible Surface Area (SASA): Surface area of a molecule accessible by a water molecule.

Polar Surface Area (PSA): Solvent accessible surface area in a molecule contributed only by oxygen and nitrogen atoms.

Report S11

MD Simulation Report on BChE - 5-*N*-Methylmaytenine Interactions

Simulation Details

Jobname: md_job_6EP4_4-dock-1
Entry title: 6EP4_4-dock-1

CPU #	Job Type	Ensemble	Temp. [K]	Sim. Time [ns]	# Atoms	# Waters	Charge
1	mdsim	NPT	300.0	10.005	51011	14184	0

Protein Information

Tot. Residues	Prot. Chain(s)	Res. in Chain(s)	# Atoms	# Heavy Atoms	Charge
527	'NoChainId'	ict_values([527]	8313	4203	+2

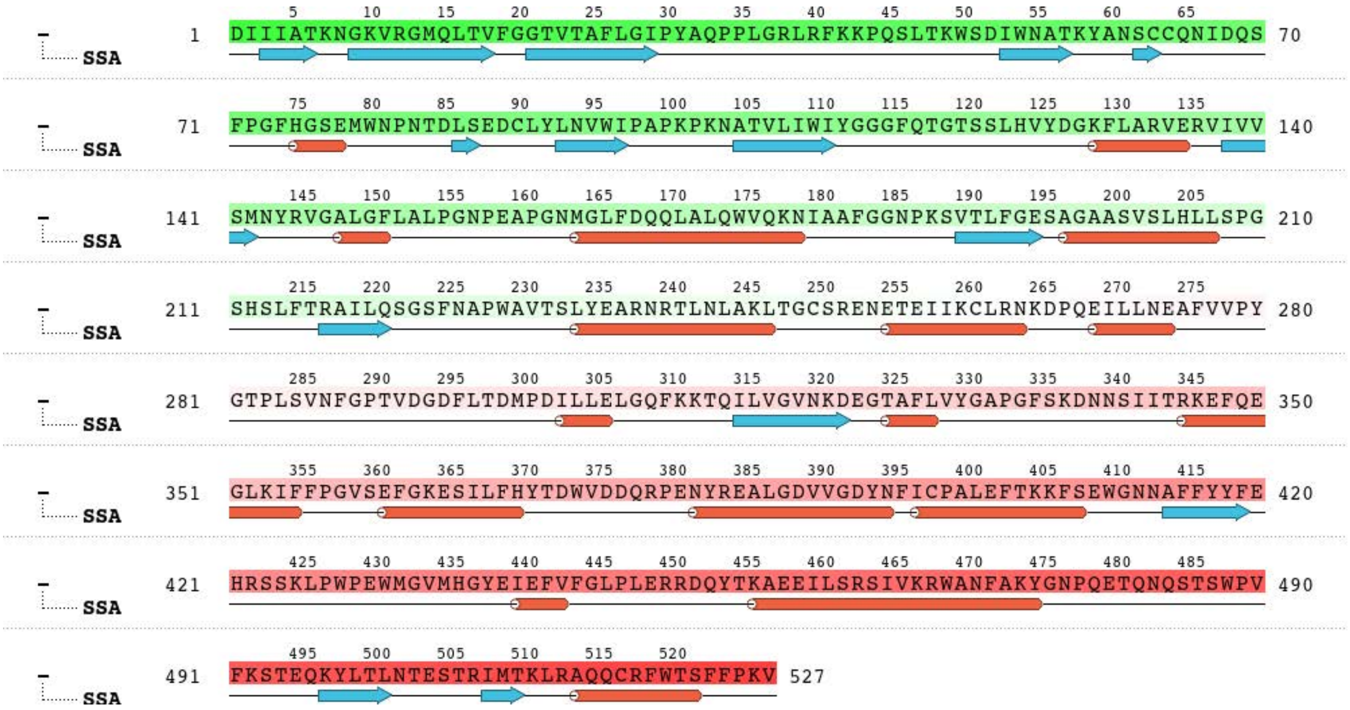

Ligand Information

SMILES	c1ccccc1\C=C\C(=O)NCCCC[N@H+](C)CCCNC(=O)/C=C/c2ccccc2
PDB Name	'UNK'
Num. of Atoms	65 (total) 31 (heavy)
Atomic Mass	420.580 au
Charge	+1
Mol. Formula	C26H34N3O2
Num. of Fragments	9
Num. of Rot. Bonds	15

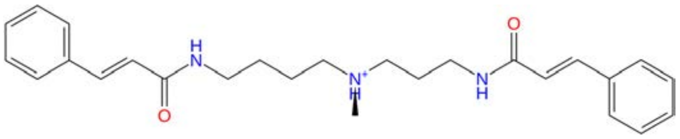

Counter Ion/Salt Information

Type	Num.	Concentration [mM]	Total Charge
Cl	42	53.838	-42
Na	39	49.992	+39

Protein-Ligand RMSD

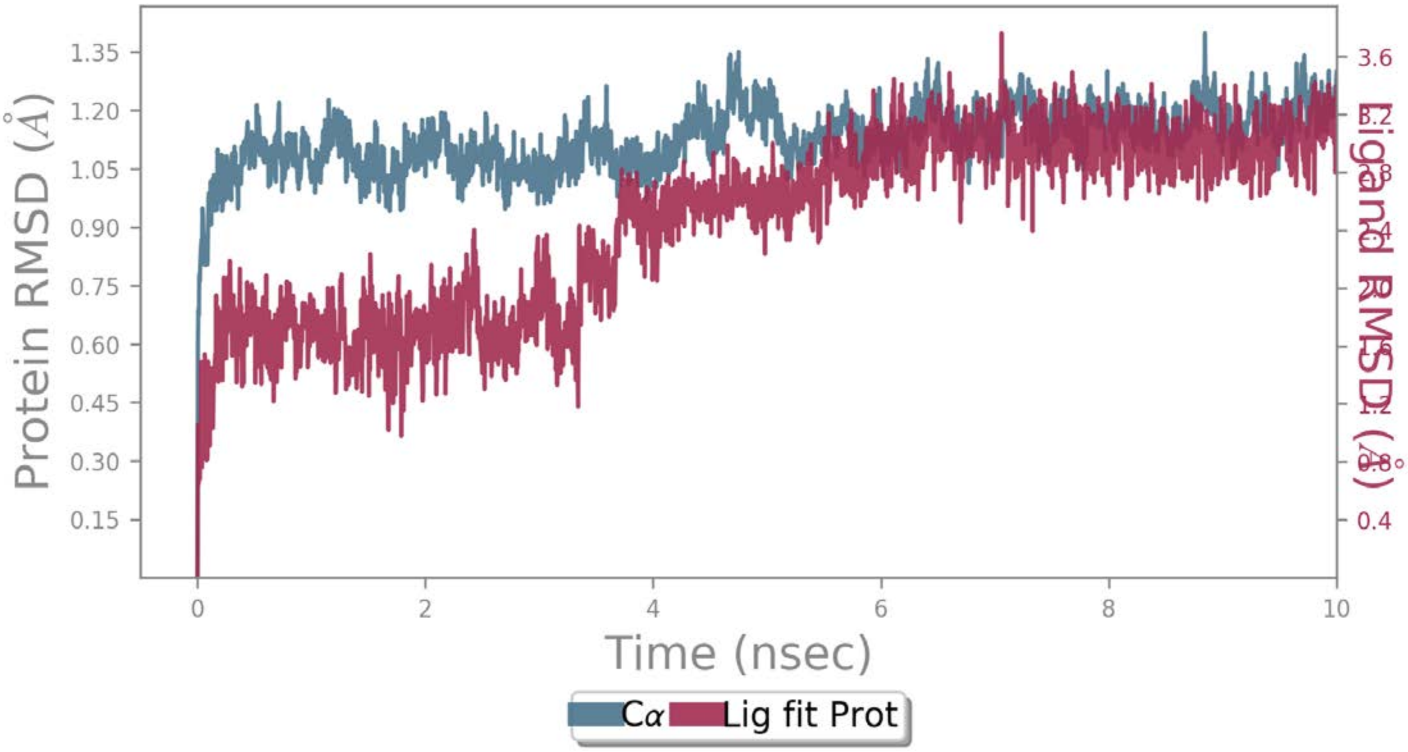

The Root Mean Square Deviation (RMSD) is used to measure the average change in displacement of a selection of atoms for a particular frame with respect to a reference frame. It is calculated for all frames in the trajectory. The RMSD for frame *x* is:

$$RMSD_x = \sqrt{\frac{1}{N}\sum_{i=1}^{N}(r'_i(t_x)) - r_i(t_{ref}))^2}$$

where *N* is the number of atoms in the atom selection; t_{ref} is the reference time, (typically the first frame is used as the reference and it is regarded as time *t*=0); and *r'* is the position of the selected atoms in frame *x* after superimposing on the reference frame, where frame *x* is recorded at time t_x. The procedure is repeated for every frame in the simulation trajectory.

Protein RMSD: The above plot shows the RMSD evolution of a protein (left *Y*-axis). All protein frames are first aligned on the reference frame backbone, and then the RMSD is calculated based on the atom selection. Monitoring the RMSD of the protein can give insights into its structural conformation throughout the simulation. RMSD analysis can indicate if the simulation has equilibrated — its fluctuations towards the end of the simulation are around some thermal average structure. Changes of the order of 1-3 Å are perfectly acceptable for small, globular proteins. Changes much larger than that, however, indicate that the protein is undergoing a large conformational change during the simulation. It is also important that your simulation converges — the RMSD values stabilize around a fixed value. If the RMSD of the protein is still increasing or decreasing on average at the end of the simulation, then your system has not equilibrated, and your simulation may not be long enough for rigorous analysis.

Ligand RMSD: Ligand RMSD (right *Y*-axis) indicates how stable the ligand is with respect to the protein and its binding pocket. In the above plot, 'Lig fit Prot' shows the RMSD of a ligand when the protein-ligand complex is first aligned on the protein backbone of the reference and then the RMSD of the ligand heavy atoms is measured. If the values observed are significantly larger than the RMSD of the protein, then it is likely that the ligand has diffused away from its initial binding site.

Protein RMSF

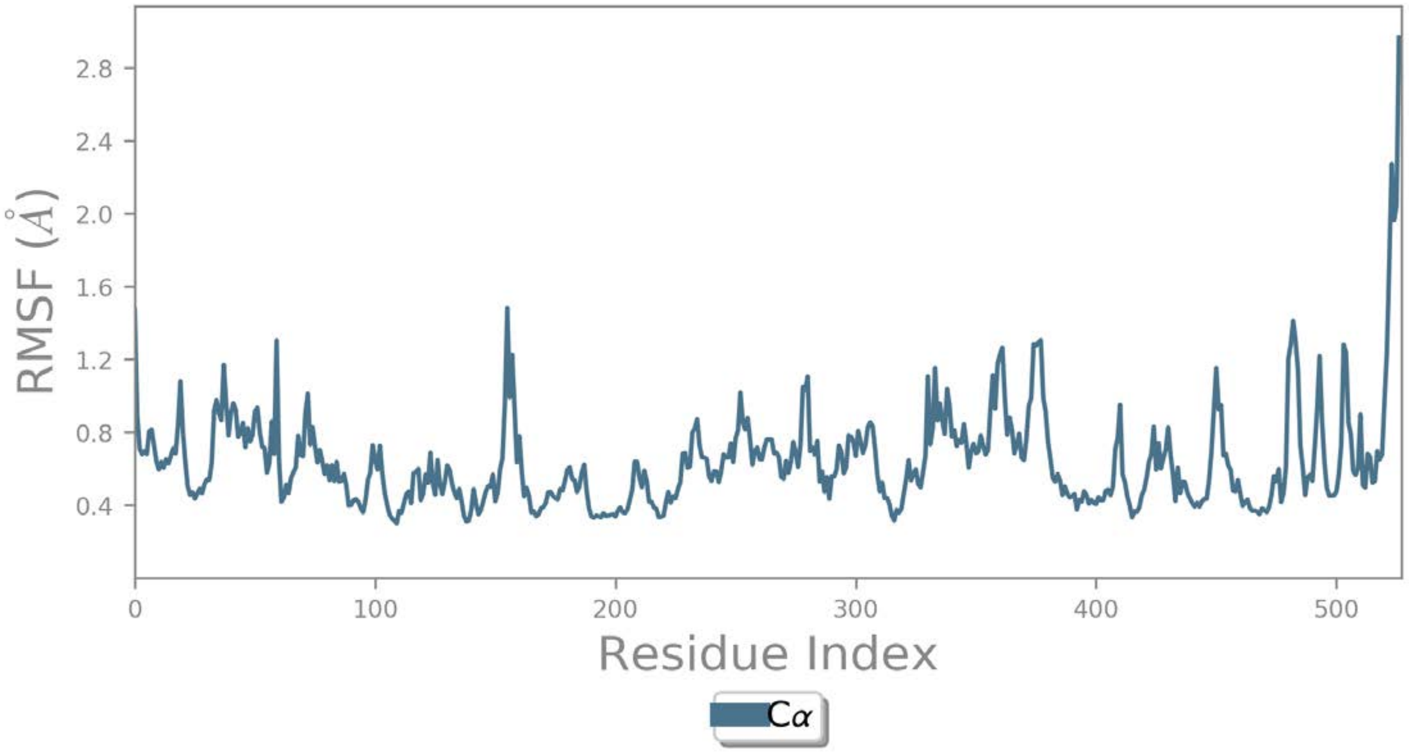

The Root Mean Square Fluctuation (RMSF) is useful for characterizing local changes along the protein chain. The RMSF for residue *i* is:

$$RMSF_i = \sqrt{\frac{1}{T}\sum_{t=1}^{T} < (r'_i(t)) - r_i(t_{ref}))^2 >}$$

where T is the trajectory time over which the RMSF is calculated, t_{ref} is the reference time, r_i is the position of residue *i*; *r'* is the position of atoms in residue *i* after superposition on the reference, and the angle brackets indicate that the average of the square distance is taken over the selection of atoms in the residue.

On this plot, peaks indicate areas of the protein that fluctuate the most during the simulation. Typically you will observe that the tails (*N*- and *C*-terminal) fluctuate more than any other part of the protein. Secondary structure elements like alpha helices and beta strands are usually more rigid than the unstructured part of the protein, and thus fluctuate less than the loop regions.

Protein Secondary Structure

% Helix	% Strand	% Total SSE
28.38	13.66	42.03

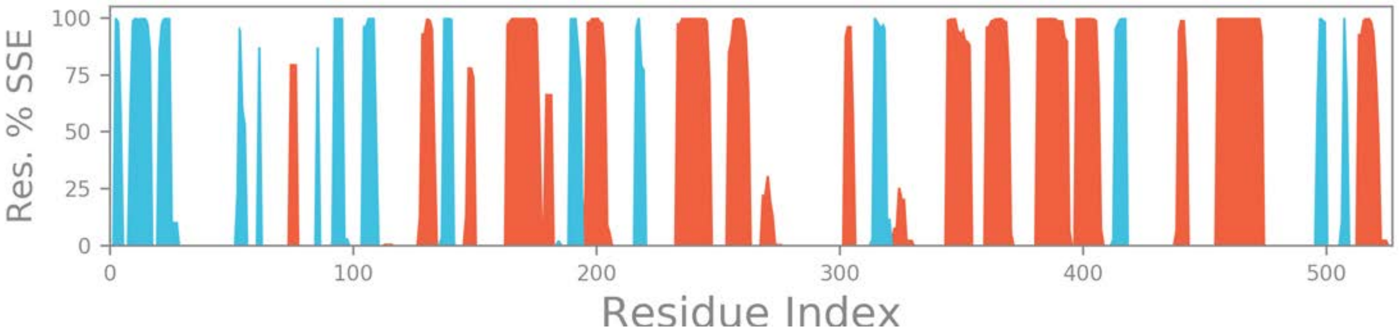

Protein secondary structure elements (SSE) like alpha-helices and beta-strands are monitored throughout the simulation. The plot above reports SSE distribution by residue index throughout the protein structure. The plot below summarizes the SSE composition for each trajectory frame over the course of the simulation, and the plot at the bottom monitors each residue and its SSE assignment over time.

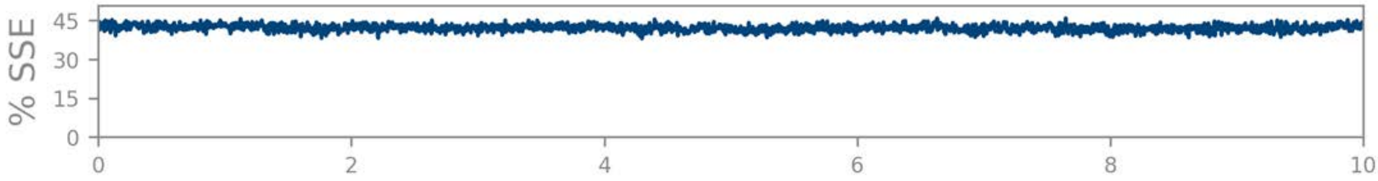

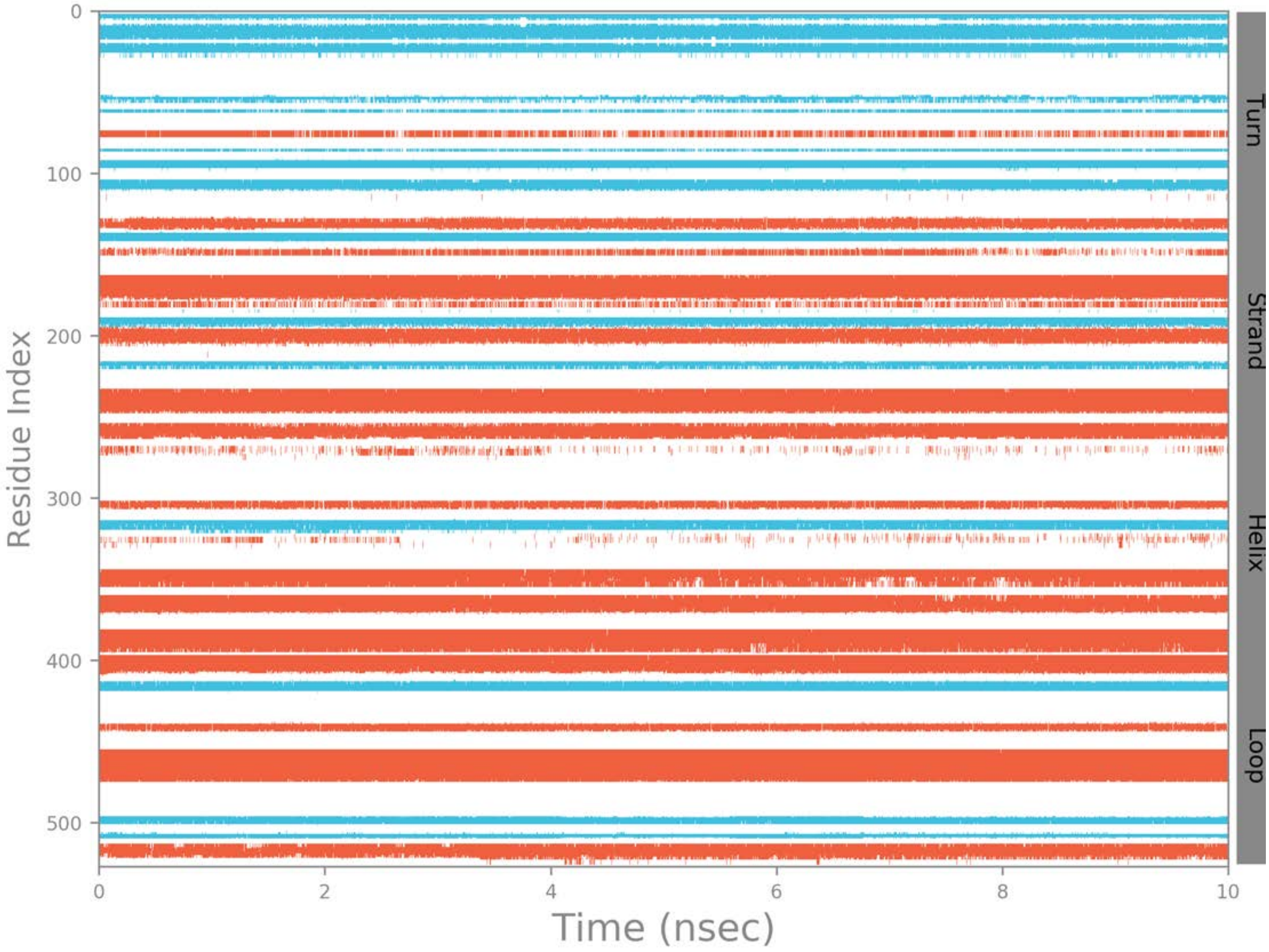

RMSF of 5-*N*-Methylmaitenine Ligand

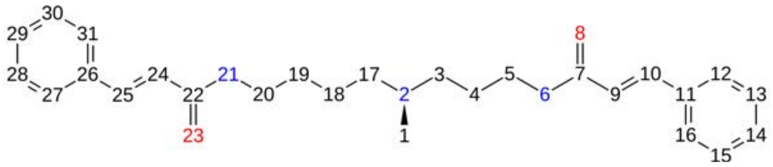

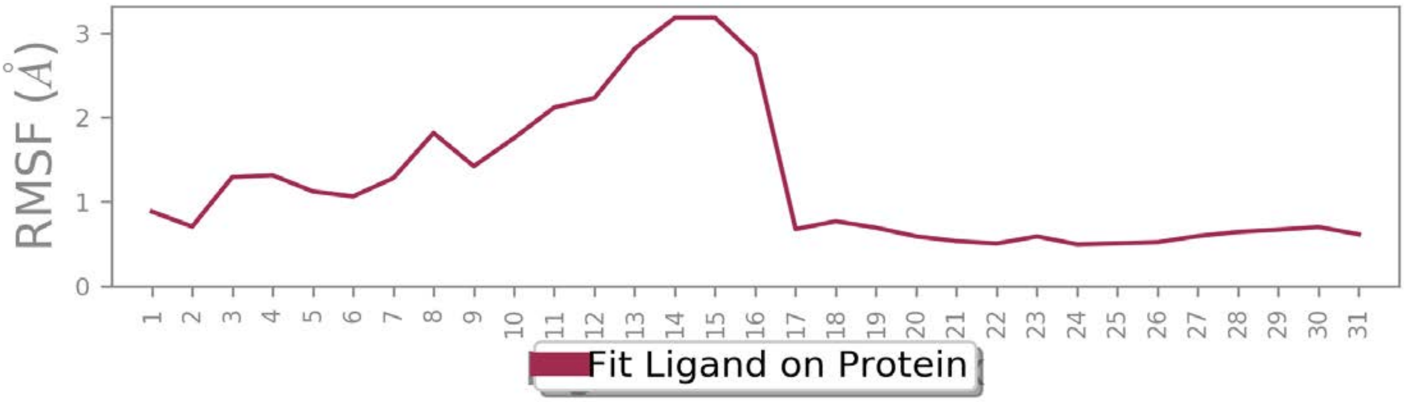

The Ligand Root Mean Square Fluctuation (L-RMSF) is useful for characterizing changes in the ligand atom positions. The RMSF for atom *i* is:

$$RMSF_i = \sqrt{\frac{1}{T}\sum_{t=1}^{T}(r_i'(t)) - r_i(t_{ref}))^2}$$

where T is the trajectory time over which the RMSF is calculated, t_{ref} is the reference time (usually for the first frame, and is regarded as the *zero* of time); r is the position of atom i in the reference at time t_{ref}, and r' is the position of atom i at time t after superposition on the reference frame.

Ligand RMSF shows the ligand's fluctuations broken down by atom, corresponding to the 2D structure in the top panel. The ligand RMSF may give you insights on how ligand fragments interact with the protein and their entropic role in the binding event. In the bottom panel, the 'Fit Ligand on Protein' line shows the ligand fluctuations, with respect to the protein. The protein-ligand complex is first aligned on the protein backbone and then the ligand RMSF is measured on the ligand heavy atoms.

Protein-Ligand Contacts

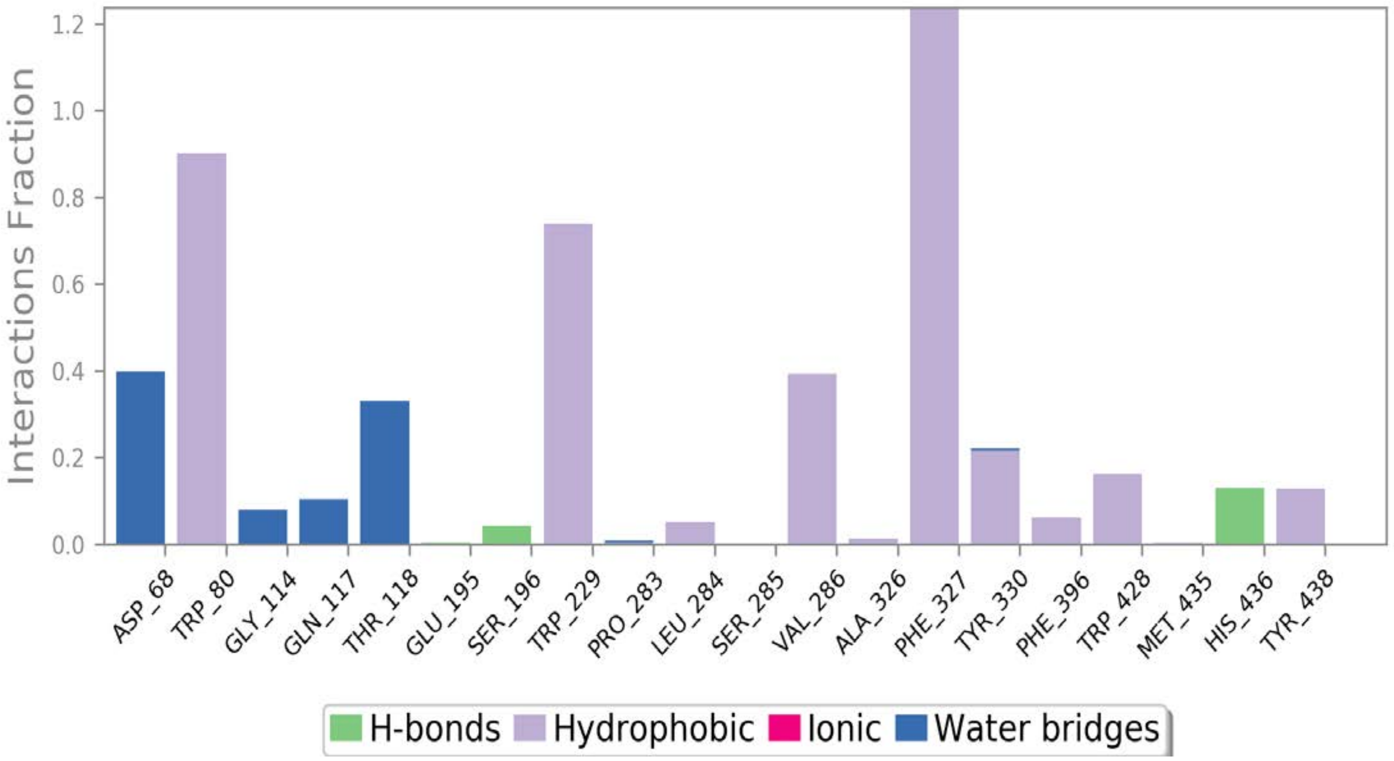

Protein interactions with the ligand can be monitored throughout the simulation. These interactions can be categorized by type and summarized, as shown in the plot above. Protein-ligand interactions (or 'contacts') are categorized into four types: Hydrogen Bonds, Hydrophobic, Ionic and Water Bridges. Each interaction type contains more specific subtypes, which can be explored through the 'Simulation Interactions Diagram' panel. The stacked bar charts are normalized over the course of the trajectory: for example, a value of 0.7 suggests that 70% of the simulation time the specific interaction is maintained. Values over 1.0 are possible as some protein residue may make multiple contacts of same subtype with the ligand.

Hydrogen Bonds: (H-bonds) play a significant role in ligand binding. Consideration of hydrogen-bonding properties in drug design is important because of their strong influence on drug specificity, metabolization and adsorption. Hydrogen bonds between a protein and a ligand can be further broken down into four subtypes: backbone acceptor; backbone donor; side-chain acceptor; side-chain donor.
The current geometric criteria for protein-ligand H-bond is: distance of 2.5 Å between the donor and acceptor atoms (D—H···A); a donor angle of ≥120° between the donor-hydrogen-acceptor atoms (D—H···A); and an acceptor angle of ≥90° between the hydrogen-acceptor-bonded_atom atoms (H···A—X).

Hydrophobic contacts: fall into three subtypes: π-Cation; π-π; and Other, non-specific interactions. Generally these type of interactions involve a hydrophobic amino acid and an aromatic or aliphatic group on the ligand, but we have extended this category to also include π-Cation interactions.
The current geometric criteria for hydrophobic interactions is as follows: π-Cation — Aromatic and charged groups within 4.5 Å; π-π — Two aromatic groups stacked face-to-face or face-to-edge; Other — A non-specific hydrophobic sidechain within 3.6 Å of a ligand's aromatic or aliphatic carbons.

Ionic interactions: or polar interactions, are between two oppositely charged atoms that are within 3.7 Å of each other and do not involve a hydrogen bond. We also monitor Protein-Metal-Ligand interactions, which are defined by a metal ion coordinated within 3.4 Å of protein's and ligand's heavy atoms (except carbon). All ionic interactions are broken down into two subtypes: those mediated by a protein backbone or side chains.

Water Bridges: are hydrogen-bonded protein-ligand interactions mediated by a water molecule. The hydrogen-bond geometry is slightly relaxed from the standard H-bond definition.
The current geometric criteria for a protein-water or water-ligand H-bond are: a distance of 2.8 Å between the donor and acceptor atoms (D—H···A); a donor angle of ≥110° between the donor-hydrogen-acceptor atoms (D—H···A); and an acceptor angle of ≥90° between the hydrogen-acceptor-bonded_atom atoms (H···A—X).

Protein-Ligand Contacts (cont.)

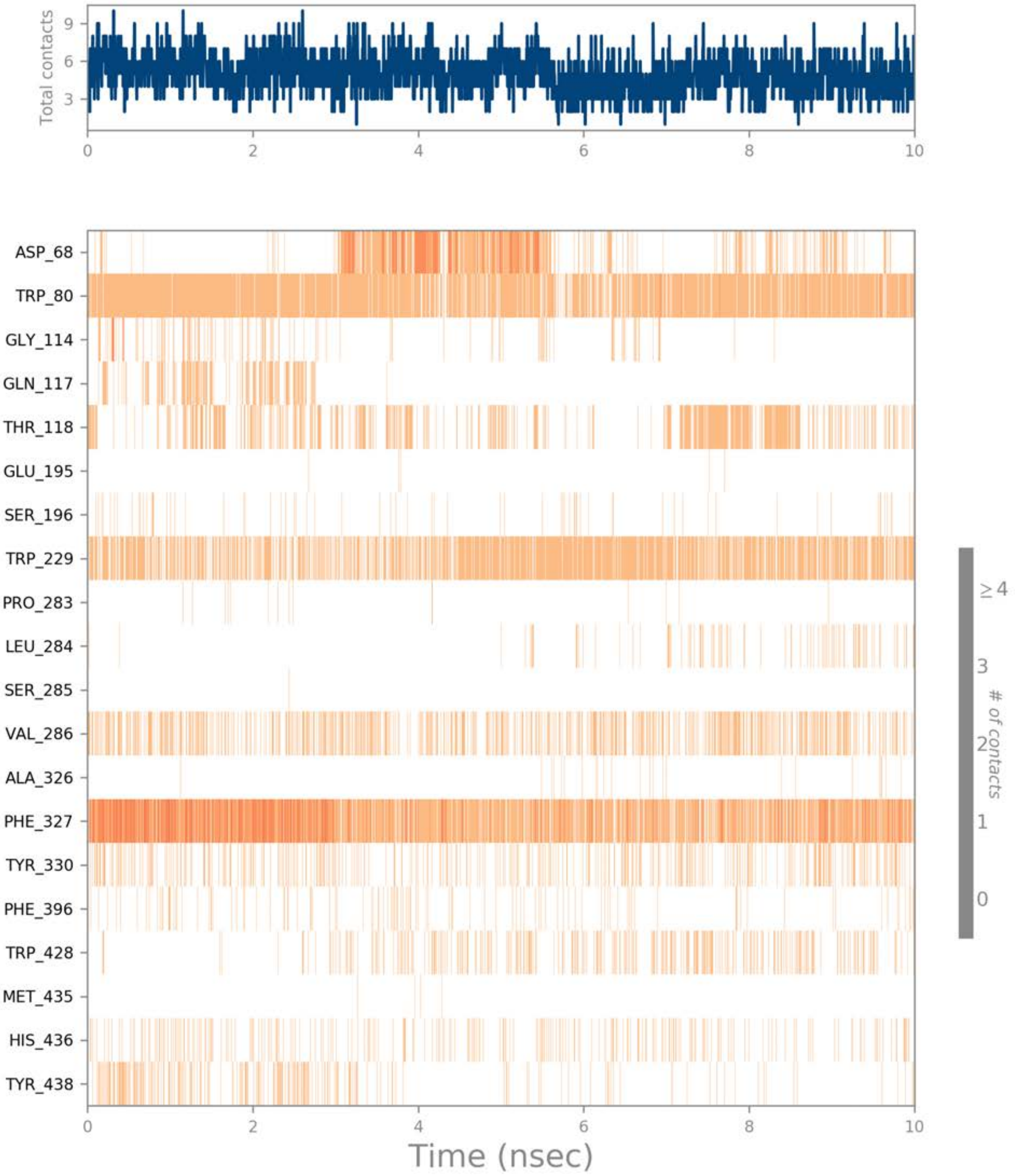

A timeline representation of the interactions and contacts (**H-bonds**, **Hydrophobic**, **Ionic**, **Water bridges**) summarized in the previous page. The top panel shows the total number of specific contacts the protein makes with the ligand over the course of the trajectory. The bottom panel shows which residues interact with the ligand in each trajectory frame. Some residues make more than one specific contact with the ligand, which is represented by a darker shade of orange, according to the scale to the right of the plot.

Ligand-Protein Contacts

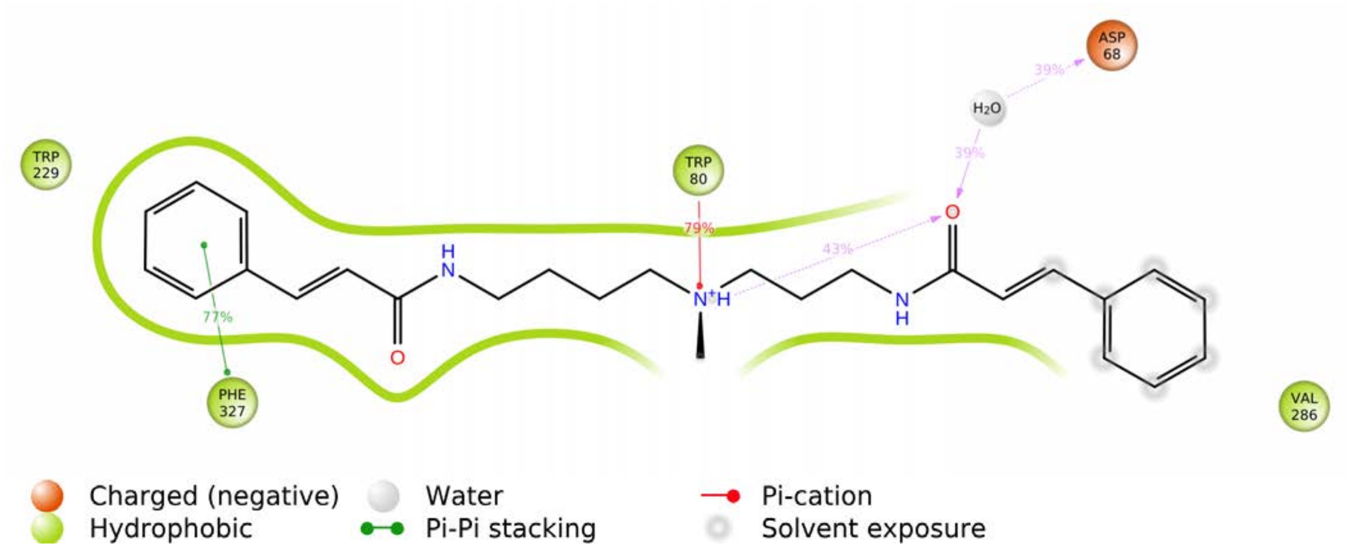

A schematic of detailed ligand atom interactions with the protein residues. Interactions that occur more than **30.0%** of the simulation time in the selected trajectory (0.00 through 10.00 nsec), are shown.
Note: it is possible to have interactions with >100% as some residues may have multiple interactions of a single type with the same ligand atom. For example, the ARG side chain has four H-bond donors that can all hydrogen-bond to a single H-bond acceptor.

Ligand Torsion Profile

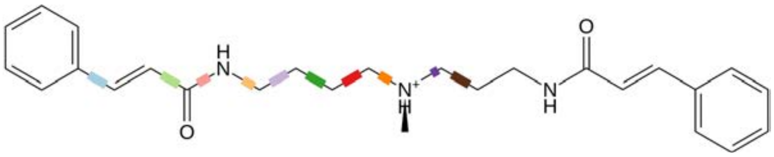

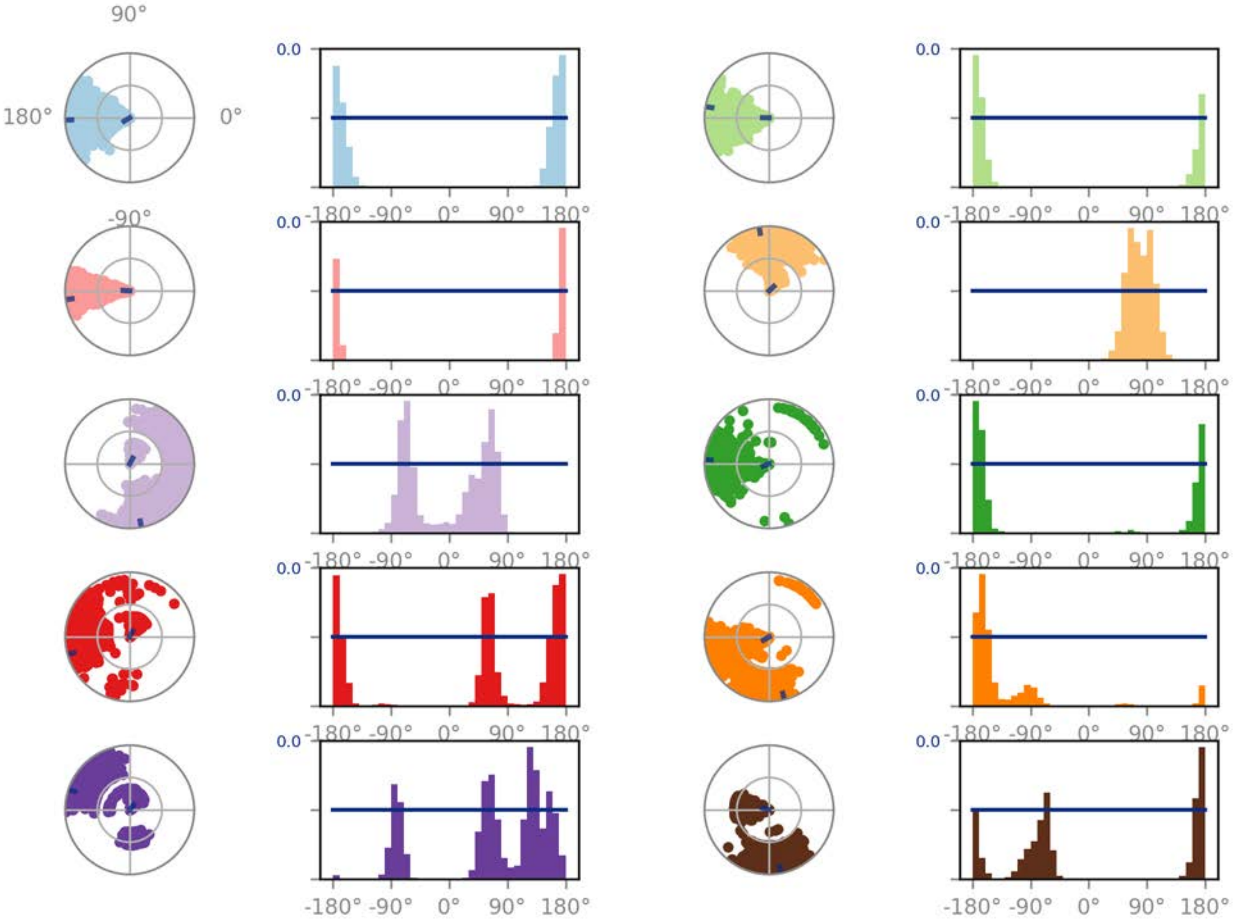

Ligand Torsion Profile (cont.)

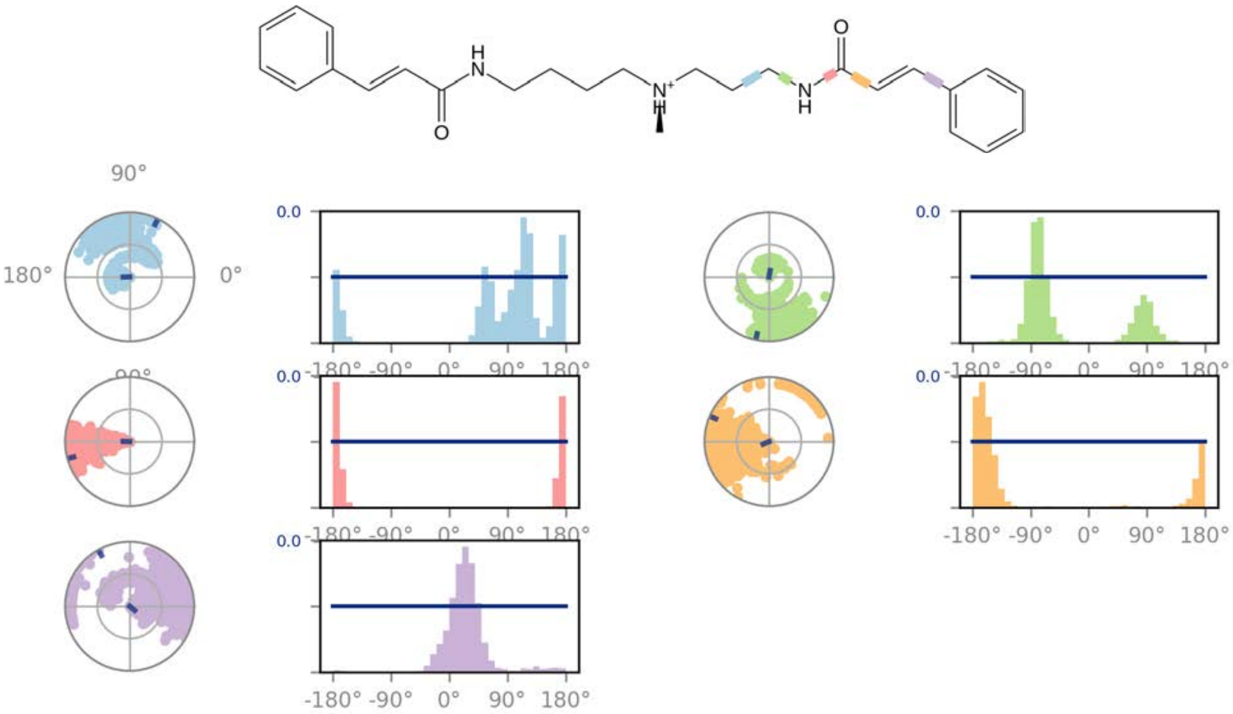

The ligand torsions plot summarizes the conformational evolution of every rotatable bond (RB) in the ligand throughout the simulation trajectory (0.00 through 10.00 nsec). The top panel shows the 2d schematic of a ligand with color-coded rotatable bonds. Each rotatable bond torsion is accompanied by a dial plot and bar plots of the same color.

Dial (or radial) plots describe the conformation of the torsion throughout the course of the simulation. The beginning of the simulation is in the center of the radial plot and the time evolution is plotted radially outwards.

The bar plots summarize the data on the dial plots, by showing the probability density of the torsion. If torsional potential information is available, the plot also shows the potential of the rotatable bond (by summing the potential of the related torsions). The values of the potential are on the left *Y*-axis of the chart, and are expressed in *kcal/mol*. Looking at the histogram and torsion potential relationships may give insights into the conformational strain the ligand undergoes to maintain a protein-bound conformation.

Ligand Properties

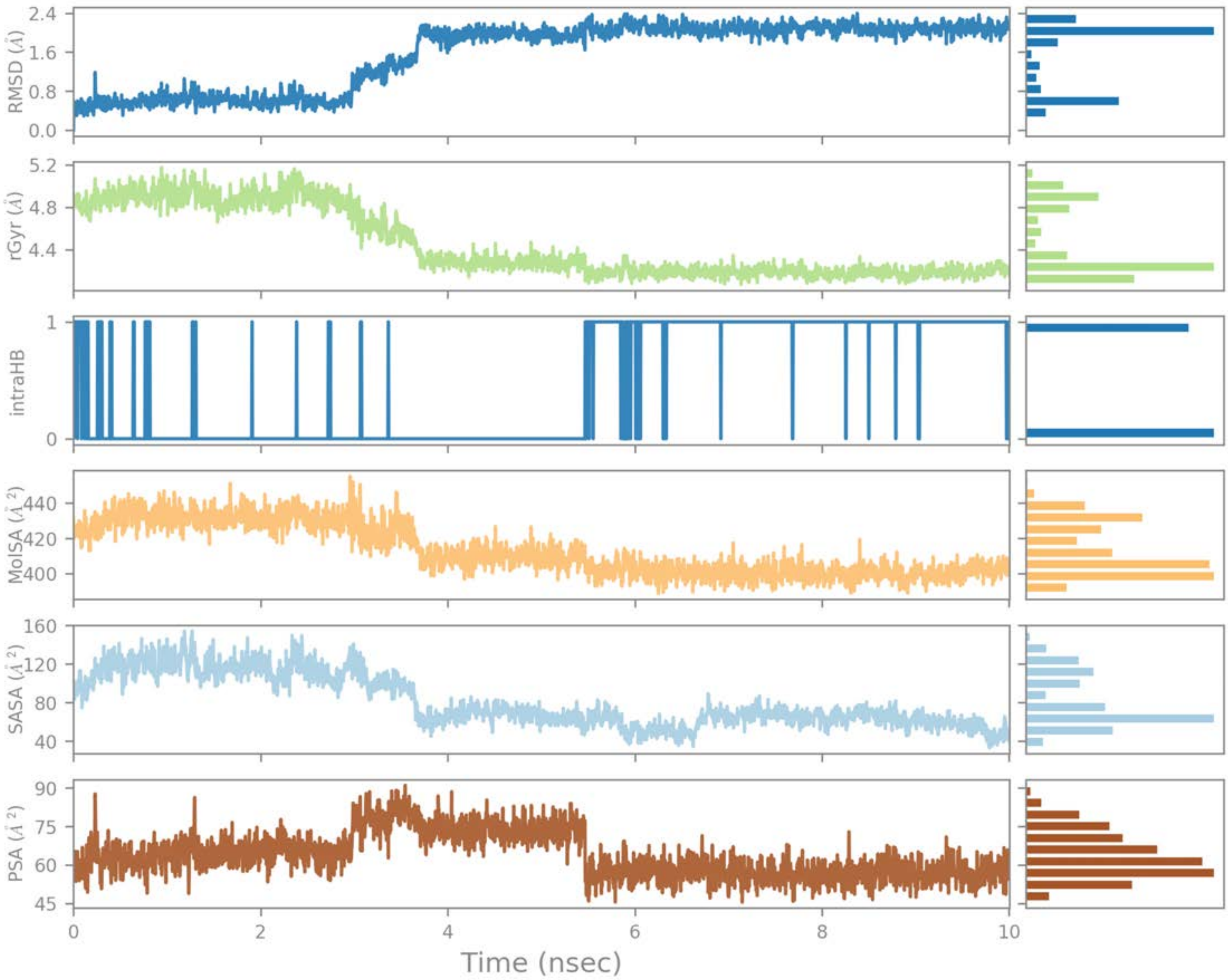

Ligand RMSD: Root mean square deviation of a ligand with respect to the reference conformation (typically the first frame is used as the reference and it is regarded as time *t*=0).

Radius of Gyration (rGyr): Measures the 'extendedness' of a ligand, and is equivalent to its principal moment of inertia.

Intramolecular Hydrogen Bonds (intraHB): Number of internal hydrogen bonds (HB) within a ligand molecule.

Molecular Surface Area (MolSA): Molecular surface calculation with 1.4 Å probe radius. This value is equivalent to a van der Waals surface area.

Solvent Accessible Surface Area (SASA): Surface area of a molecule accessible by a water molecule.

Polar Surface Area (PSA): Solvent accessible surface area in a molecule contributed only by oxygen and nitrogen atoms.

Report S12

MD Simulation Report on BChE - *N-trans*-Feruloyltyramine Interactions

Simulation Details

Jobname: md_job_6EP4_5-dock-1
Entry title: 6EP4_5-dock-1

CPU #	Job Type	Ensemble	Temp. [K]	Sim. Time [ns]	# Atoms	# Waters	Charge
1	mdsim	NPT	300.0	10.005	51014	14193	0

Protein Information

Tot. Residues	Prot. Chain(s)	Res. in Chain(s)	# Atoms	# Heavy Atoms	Charge
527	'NoChainId'	ict_values([527]	8313	4203	+2

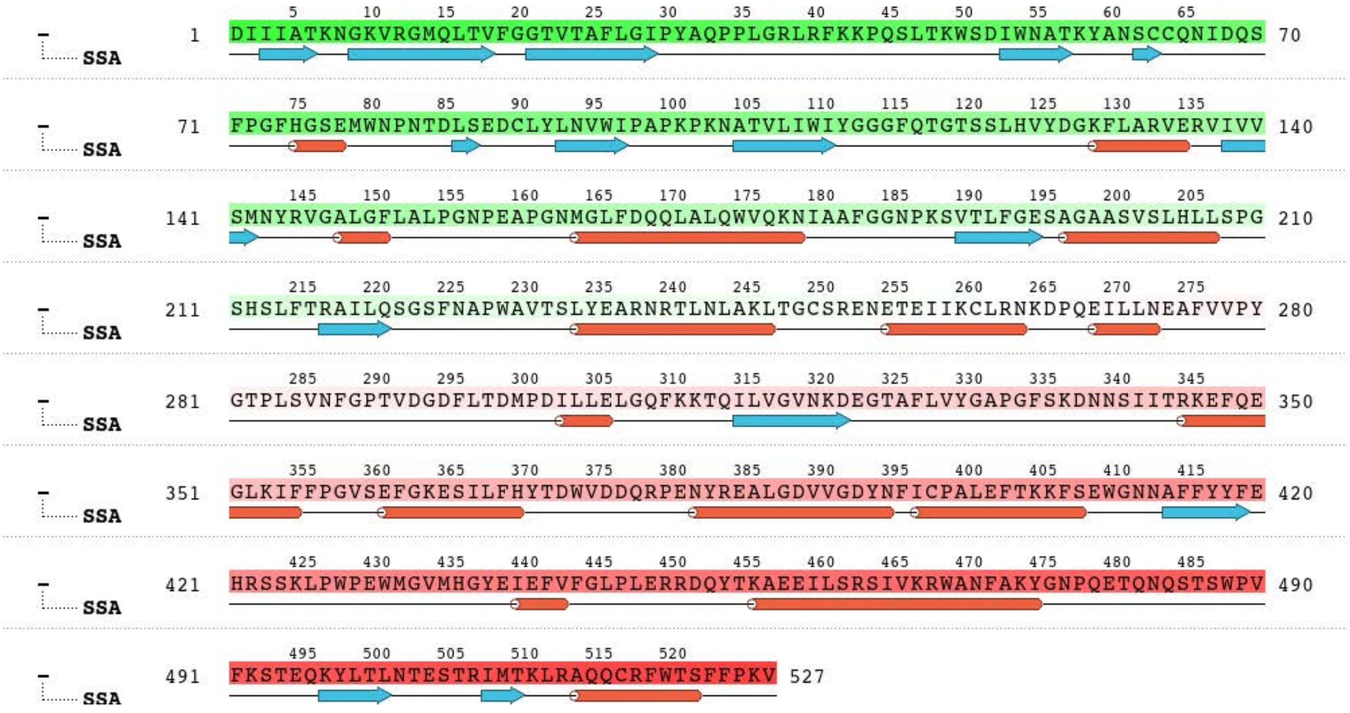

Ligand Information

SMILES	COc(cc1)c(O)cc1\C=C\C(=O)NCCc2ccc(O)cc2
PDB Name	'UNK'
Num. of Atoms	42 (total) 23 (heavy)
Atomic Mass	313.356 au
Charge	0
Mol. Formula	C18H19NO4
Num. of Fragments	4
Num. of Rot. Bonds	9

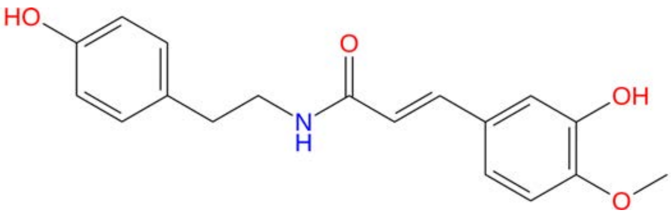

Counter Ion/Salt Information

Type	Num.	Concentration [mM]	Total Charge
Cl	41	52.523	-41
Na	39	49.961	+39

Protein-Ligand RMSD

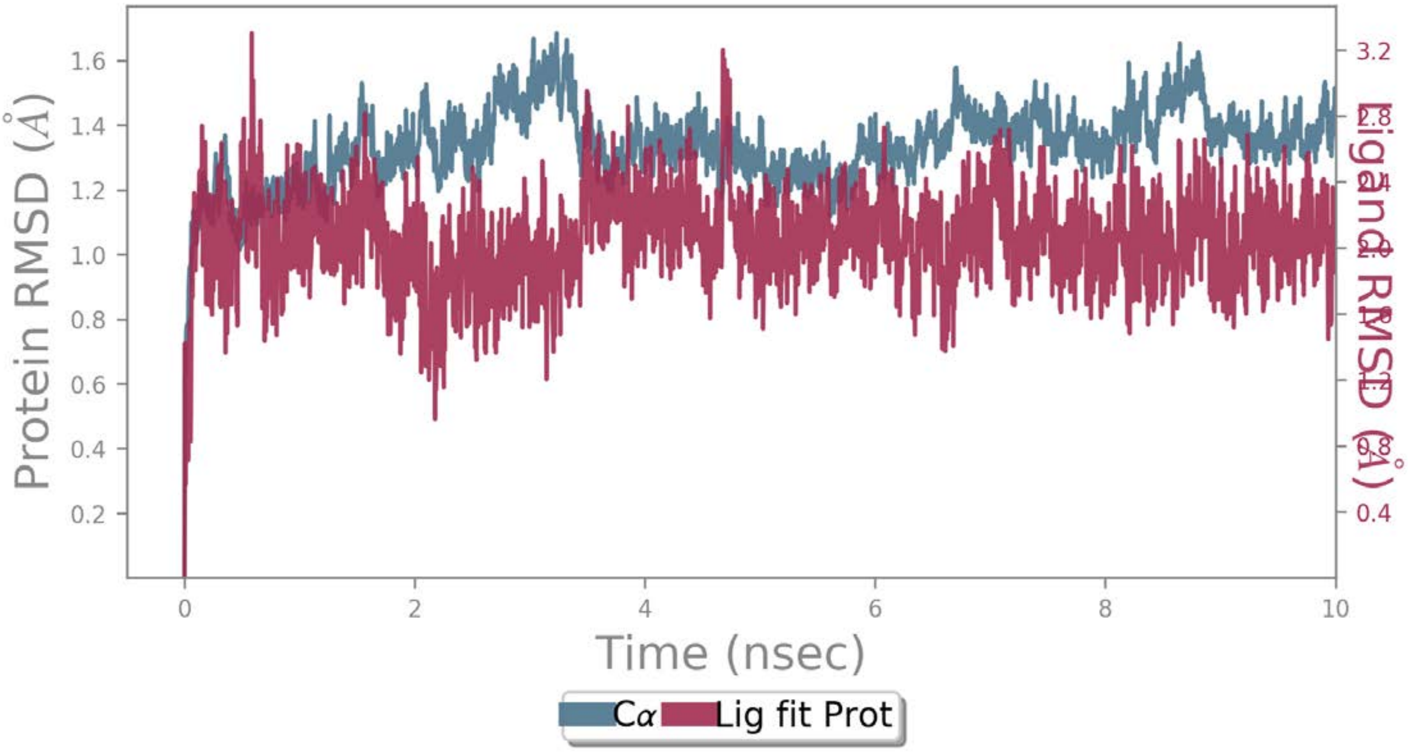

The Root Mean Square Deviation (RMSD) is used to measure the average change in displacement of a selection of atoms for a particular frame with respect to a reference frame. It is calculated for all frames in the trajectory. The RMSD for frame *x* is:

$$RMSD_x = \sqrt{\frac{1}{N}\sum_{i=1}^{N}(r'_i(t_x)) - r_i(t_{ref}))^2}$$

where *N* is the number of atoms in the atom selection; t_{ref} is the reference time, (typically the first frame is used as the reference and it is regarded as time *t*=0); and *r'* is the position of the selected atoms in frame *x* after superimposing on the reference frame, where frame *x* is recorded at time t_x. The procedure is repeated for every frame in the simulation trajectory.

Protein RMSD: The above plot shows the RMSD evolution of a protein (left *Y*-axis). All protein frames are first aligned on the reference frame backbone, and then the RMSD is calculated based on the atom selection. Monitoring the RMSD of the protein can give insights into its structural conformation throughout the simulation. RMSD analysis can indicate if the simulation has equilibrated — its fluctuations towards the end of the simulation are around some thermal average structure. Changes of the order of 1-3 Å are perfectly acceptable for small, globular proteins. Changes much larger than that, however, indicate that the protein is undergoing a large conformational change during the simulation. It is also important that your simulation converges — the RMSD values stabilize around a fixed value. If the RMSD of the protein is still increasing or decreasing on average at the end of the simulation, then your system has not equilibrated, and your simulation may not be long enough for rigorous analysis.

Ligand RMSD: Ligand RMSD (right *Y*-axis) indicates how stable the ligand is with respect to the protein and its binding pocket. In the above plot, 'Lig fit Prot' shows the RMSD of a ligand when the protein-ligand complex is first aligned on the protein backbone of the reference and then the RMSD of the ligand heavy atoms is measured. If the values observed are significantly larger than the RMSD of the protein, then it is likely that the ligand has diffused away from its initial binding site.

Protein RMSF

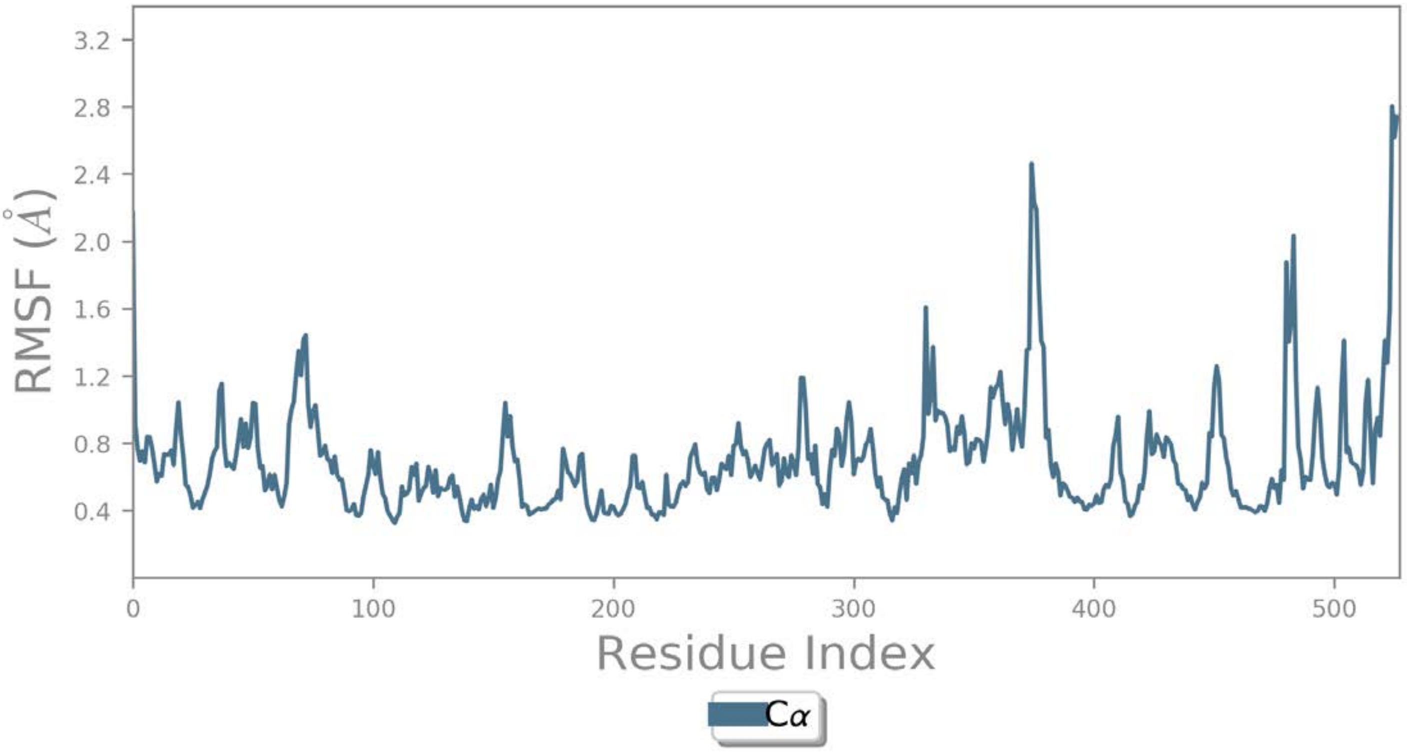

The Root Mean Square Fluctuation (RMSF) is useful for characterizing local changes along the protein chain. The RMSF for residue *i* is:

$$RMSF_i = \sqrt{\frac{1}{T}\sum_{t=1}^{T} < (r'_i(t)) - r_i(t_{ref}))^2 >}$$

where T is the trajectory time over which the RMSF is calculated, t_{ref} is the reference time, r_i is the position of residue *i*; *r'* is the position of atoms in residue *i* after superposition on the reference, and the angle brackets indicate that the average of the square distance is taken over the selection of atoms in the residue.

On this plot, peaks indicate areas of the protein that fluctuate the most during the simulation. Typically you will observe that the tails (*N*- and *C*-terminal) fluctuate more than any other part of the protein. Secondary structure elements like alpha helices and beta strands are usually more rigid than the unstructured part of the protein, and thus fluctuate less than the loop regions.

Protein Secondary Structure

% Helix	% Strand	% Total SSE
27.86	13.74	41.61

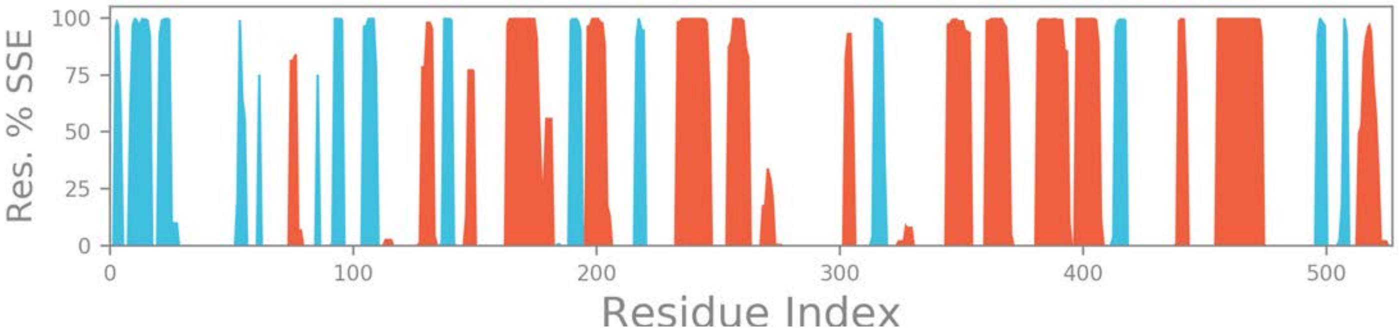

Protein secondary structure elements (SSE) like alpha-helices and beta-strands are monitored throughout the simulation. The plot above reports SSE distribution by residue index throughout the protein structure. The plot below summarizes the SSE composition for each trajectory frame over the course of the simulation, and the plot at the bottom monitors each residue and its SSE assignment over time.

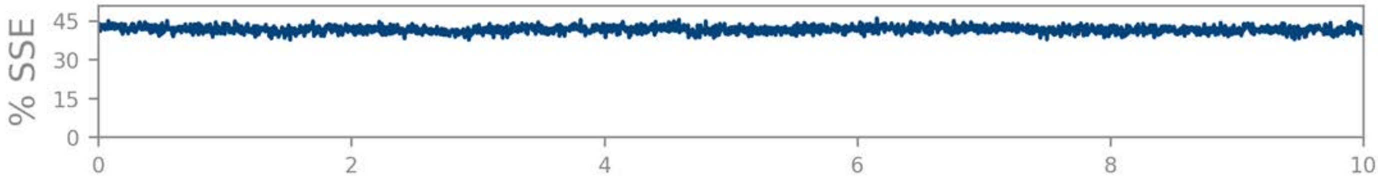

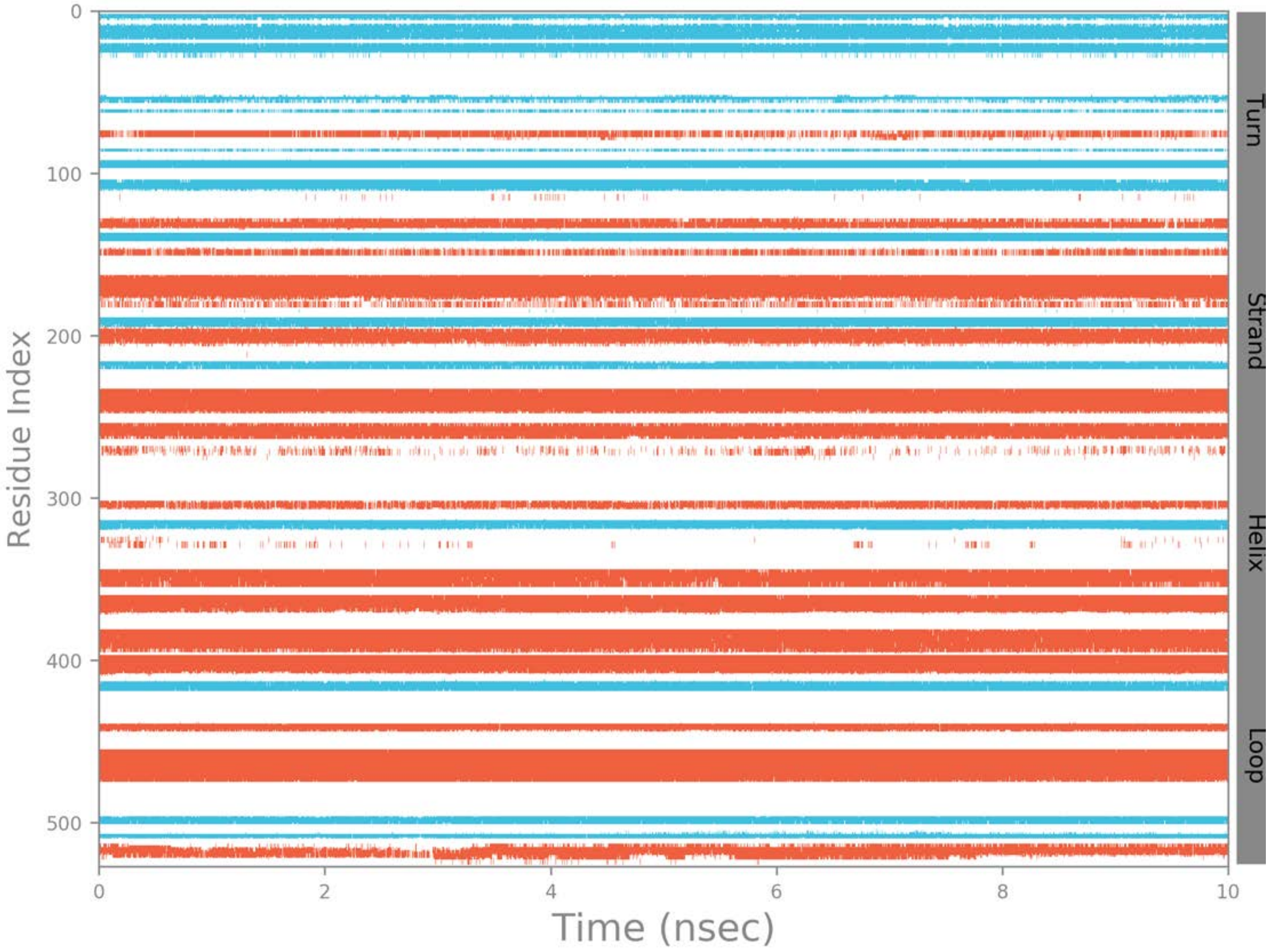

RMSF of *N-trans*-Feruloyltyramine Ligand

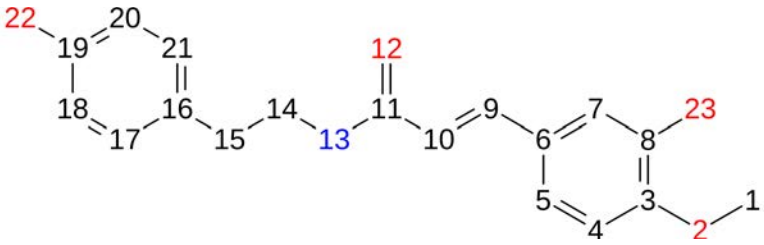

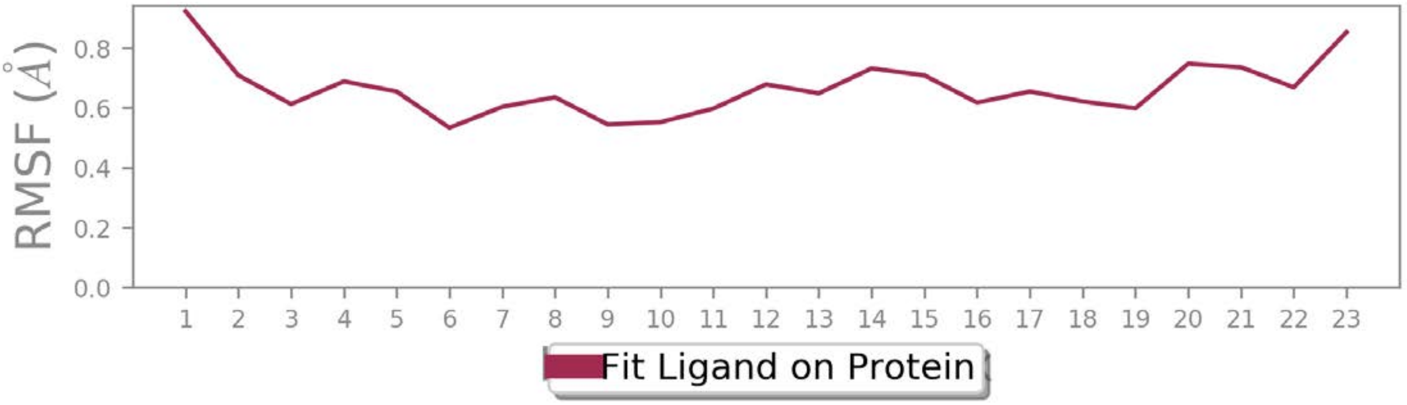

The Ligand Root Mean Square Fluctuation (L-RMSF) is useful for characterizing changes in the ligand atom positions. The RMSF for atom *i* is:

$$RMSF_i = \sqrt{\frac{1}{T}\sum_{t=1}^{T}(r'_i(t)) - r_i(t_{ref}))^2}$$

where *T* is the trajectory time over which the RMSF is calculated, t_{ref} is the reference time (usually for the first frame, and is regarded as the *zero* of time); *r* is the position of atom *i* in the reference at time t_{ref}, and *r'* is the position of atom *i* at time *t* after superposition on the reference frame.

Ligand RMSF shows the ligand's fluctuations broken down by atom, corresponding to the 2D structure in the top panel. The ligand RMSF may give you insights on how ligand fragments interact with the protein and their entropic role in the binding event. In the bottom panel, the 'Fit Ligand on Protein' line shows the ligand fluctuations, with respect to the protein. The protein-ligand complex is first aligned on the protein backbone and then the ligand RMSF is measured on the ligand heavy atoms.

Protein-Ligand Contacts

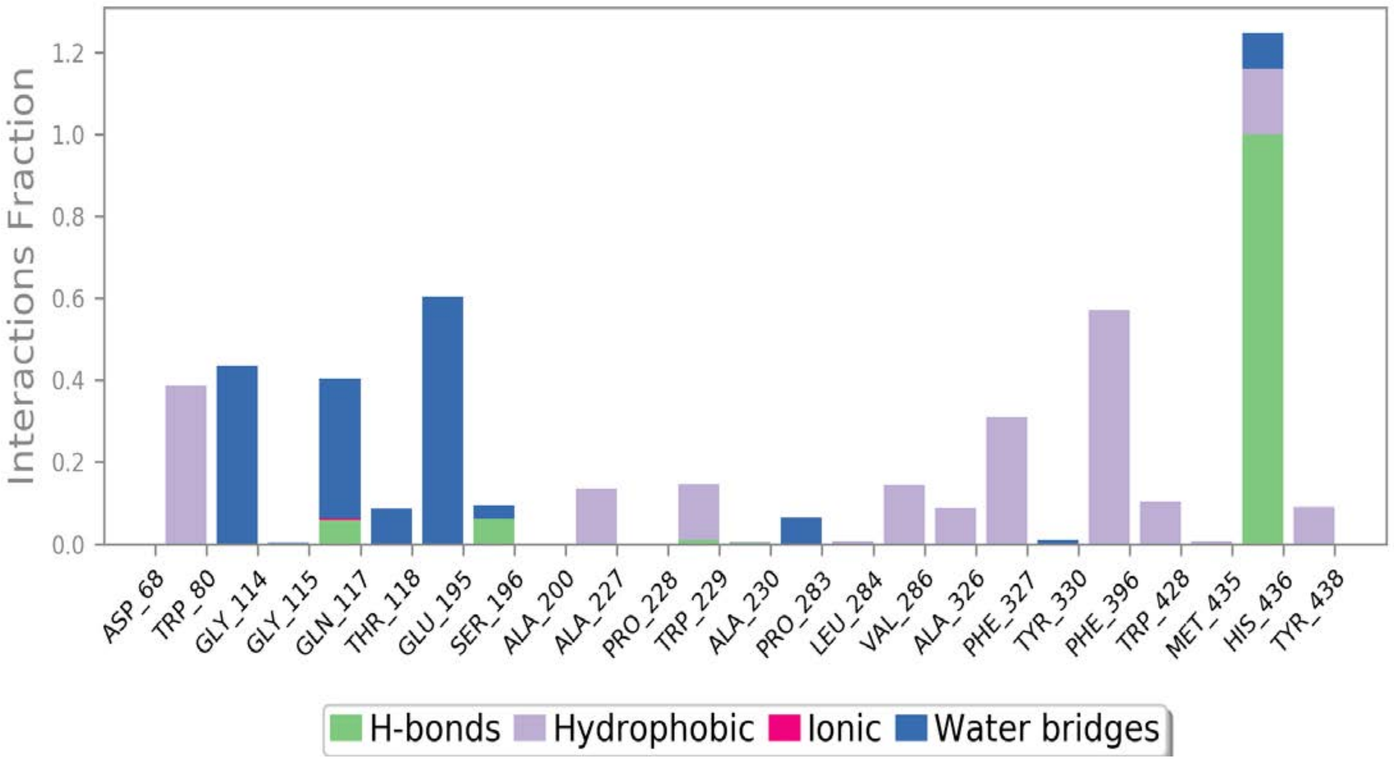

Protein interactions with the ligand can be monitored throughout the simulation. These interactions can be categorized by type and summarized, as shown in the plot above. Protein-ligand interactions (or 'contacts') are categorized into four types: Hydrogen Bonds, Hydrophobic, Ionic and Water Bridges. Each interaction type contains more specific subtypes, which can be explored through the 'Simulation Interactions Diagram' panel. The stacked bar charts are normalized over the course of the trajectory: for example, a value of 0.7 suggests that 70% of the simulation time the specific interaction is maintained. Values over 1.0 are possible as some protein residue may make multiple contacts of same subtype with the ligand.

Hydrogen Bonds: (H-bonds) play a significant role in ligand binding. Consideration of hydrogen-bonding properties in drug design is important because of their strong influence on drug specificity, metabolization and adsorption. Hydrogen bonds between a protein and a ligand can be further broken down into four subtypes: backbone acceptor; backbone donor; side-chain acceptor; side-chain donor.
The current geometric criteria for protein-ligand H-bond is: distance of 2.5 Å between the donor and acceptor atoms (D—H···A); a donor angle of ≥120° between the donor-hydrogen-acceptor atoms (D—H···A); and an acceptor angle of ≥90° between the hydrogen-acceptor-bonded_atom atoms (H···A—X).

Hydrophobic contacts: fall into three subtypes: π-Cation; π-π; and Other, non-specific interactions. Generally these type of interactions involve a hydrophobic amino acid and an aromatic or aliphatic group on the ligand, but we have extended this category to also include π-Cation interactions.
The current geometric criteria for hydrophobic interactions is as follows: π-Cation — Aromatic and charged groups within 4.5 Å; π-π — Two aromatic groups stacked face-to-face or face-to-edge; Other — A non-specific hydrophobic sidechain within 3.6 Å of a ligand's aromatic or aliphatic carbons.

Ionic interactions: or polar interactions, are between two oppositely charged atoms that are within 3.7 Å of each other and do not involve a hydrogen bond. We also monitor Protein-Metal-Ligand interactions, which are defined by a metal ion coordinated within 3.4 Å of protein's and ligand's heavy atoms (except carbon). All ionic interactions are broken down into two subtypes: those mediated by a protein backbone or side chains.

Water Bridges: are hydrogen-bonded protein-ligand interactions mediated by a water molecule. The hydrogen-bond geometry is slightly relaxed from the standard H-bond definition.
The current geometric criteria for a protein-water or water-ligand H-bond are: a distance of 2.8 Å between the donor and acceptor atoms (D—H···A); a donor angle of ≥110° between the donor-hydrogen-acceptor atoms (D—H···A); and an acceptor angle of ≥90° between the hydrogen-acceptor-bonded_atom atoms (H···A—X).

Protein-Ligand Contacts (cont.)

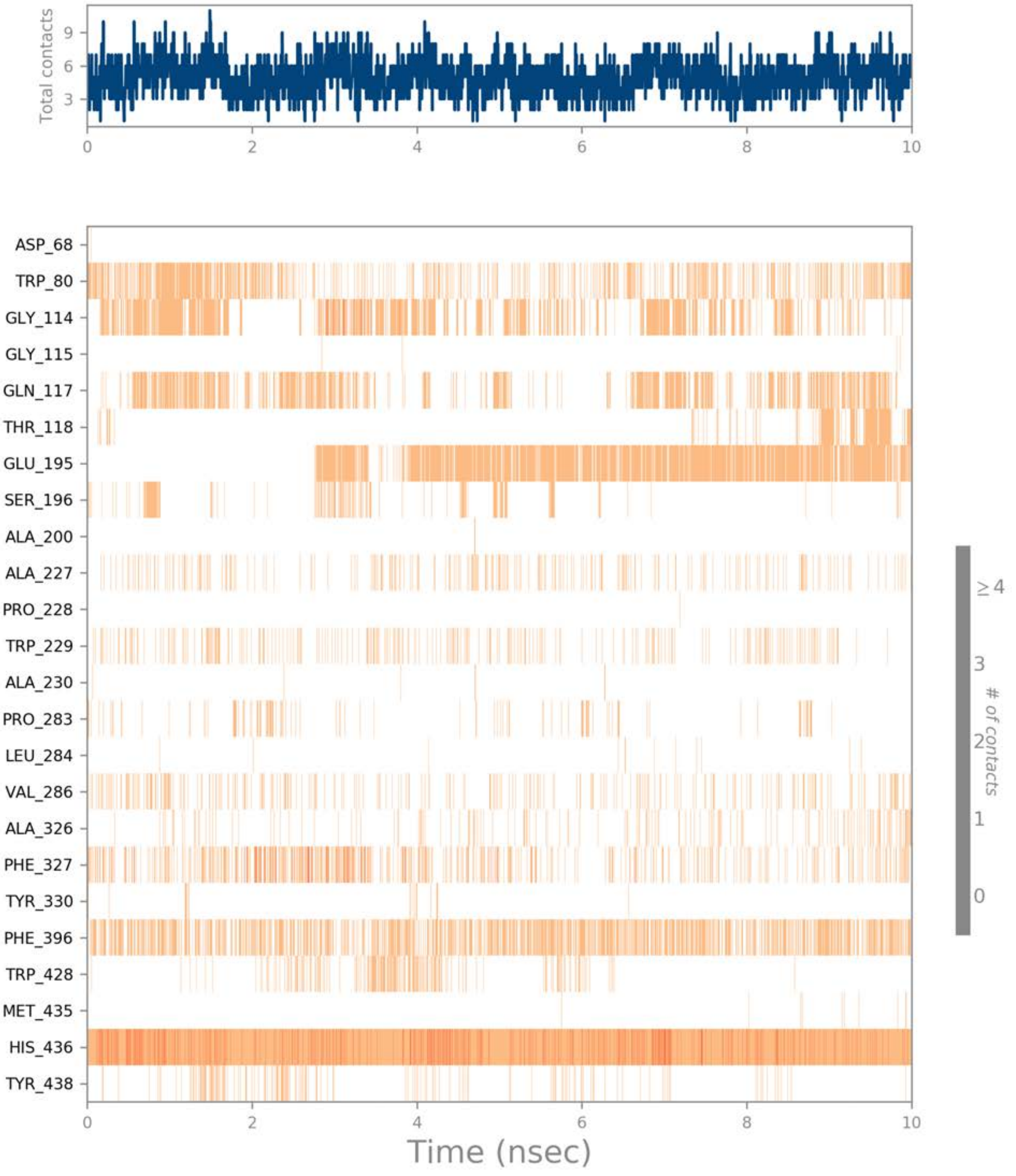

A timeline representation of the interactions and contacts (**H-bonds**, **Hydrophobic**, **Ionic**, **Water bridges**) summarized in the previous page. The top panel shows the total number of specific contacts the protein makes with the ligand over the course of the trajectory. The bottom panel shows which residues interact with the ligand in each trajectory frame. Some residues make more than one specific contact with the ligand, which is represented by a darker shade of orange, according to the scale to the right of the plot.

Ligand-Protein Contacts

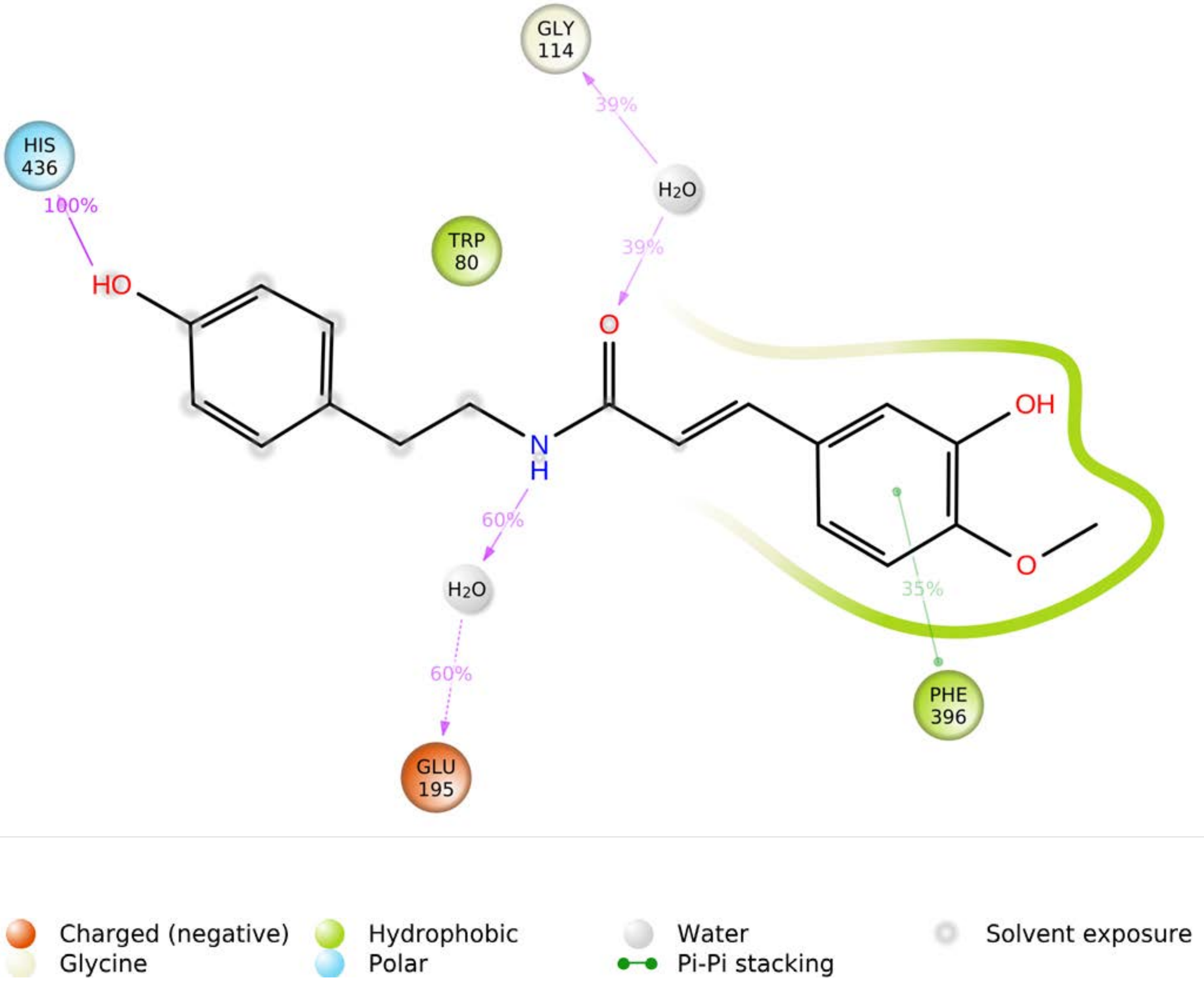

A schematic of detailed ligand atom interactions with the protein residues. Interactions that occur more than **30.0%** of the simulation time in the selected trajectory (0.00 through 10.00 nsec), are shown.
Note: it is possible to have interactions with >100% as some residues may have multiple interactions of a single type with the same ligand atom. For example, the ARG side chain has four H-bond donors that can all hydrogen-bond to a single H-bond acceptor.

Ligand Torsion Profile

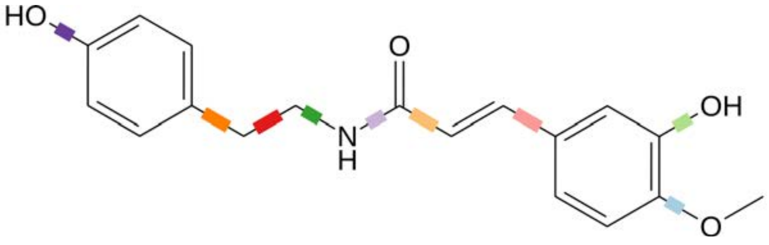

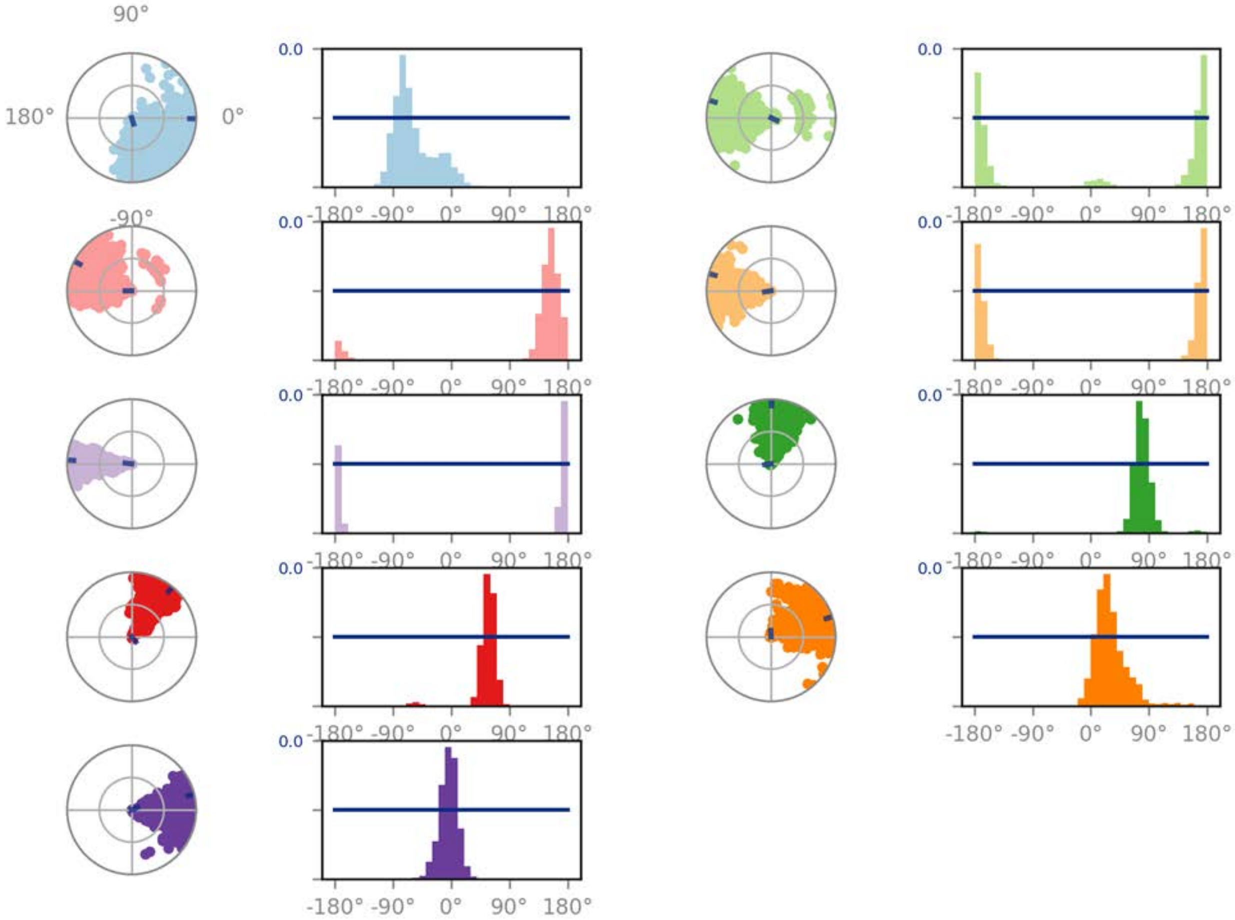

The ligand torsions plot summarizes the conformational evolution of every rotatable bond (RB) in the ligand throughout the simulation trajectory (0.00 through 10.00 nsec). The top panel shows the 2d schematic of a ligand with color-coded rotatable bonds. Each rotatable bond torsion is accompanied by a dial plot and bar plots of the same color.

Dial (or radial) plots describe the conformation of the torsion throughout the course of the simulation. The beginning of the simulation is in the center of the radial plot and the time evolution is plotted radially outwards.

The bar plots summarize the data on the dial plots, by showing the probability density of the torsion. If torsional potential information is available, the plot also shows the potential of the rotatable bond (by summing the potential of the related torsions). The values of the potential are on the left *Y*-axis of the chart, and are expressed in *kcal/mol*. Looking at the histogram and torsion potential relationships may give insights into the conformational strain the ligand undergoes to maintain a protein-bound conformation.

Ligand Properties

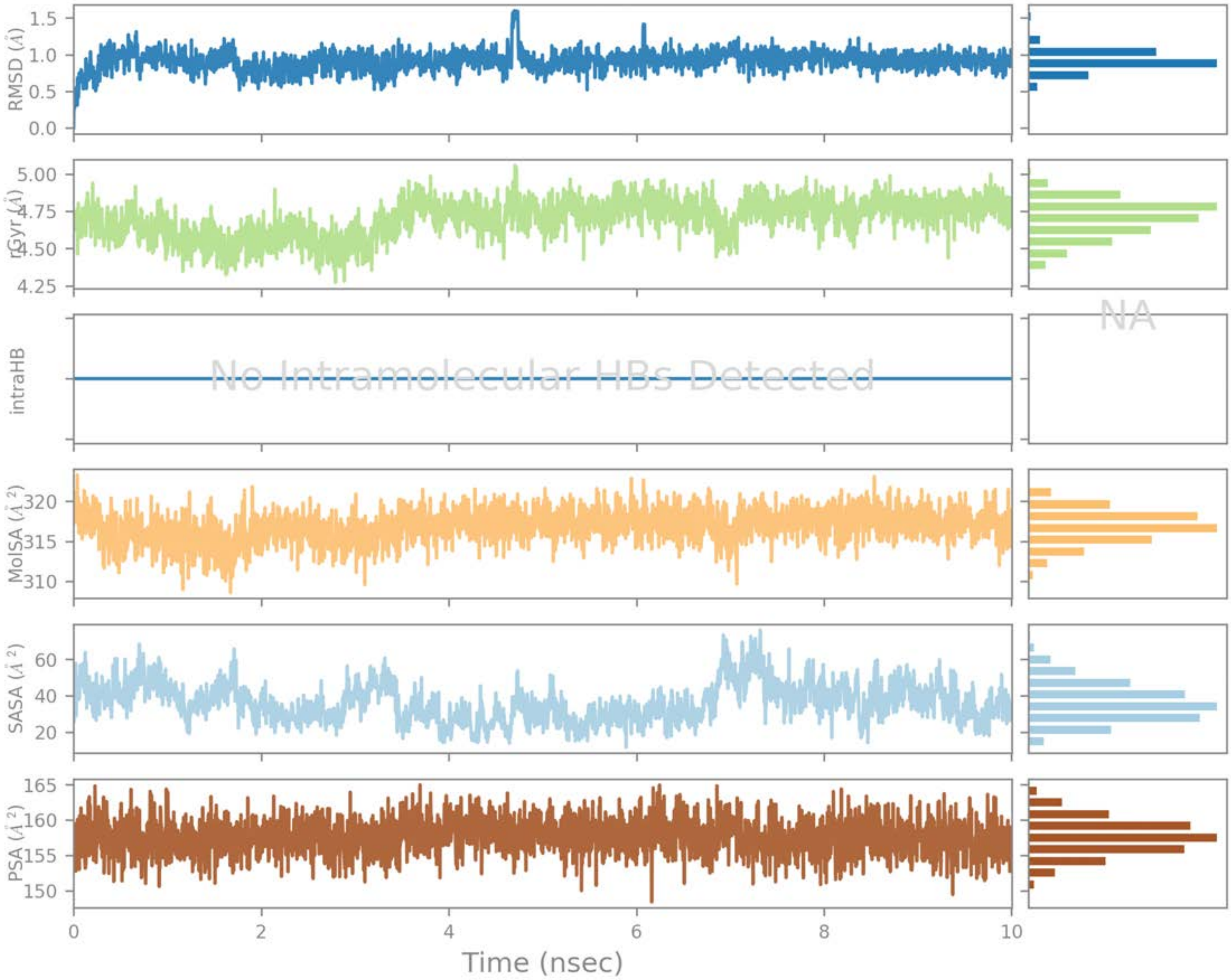

Ligand RMSD: Root mean square deviation of a ligand with respect to the reference conformation (typically the first frame is used as the reference and it is regarded as time *t*=0).

Radius of Gyration (rGyr): Measures the 'extendedness' of a ligand, and is equivalent to its principal moment of inertia.

Intramolecular Hydrogen Bonds (intraHB): Number of internal hydrogen bonds (HB) within a ligand molecule.

Molecular Surface Area (MolSA): Molecular surface calculation with 1.4 Å probe radius. This value is equivalent to a van der Waals surface area.

Solvent Accessible Surface Area (SASA): Surface area of a molecule accessible by a water molecule.

Polar Surface Area (PSA): Solvent accessible surface area in a molecule contributed only by oxygen and nitrogen atoms.
